# The genome of the golden apple snail *Pomacea canaliculata* provides insight into stress tolerance and invasive adaptation

**DOI:** 10.1093/gigascience/giy101

**Published:** 2018-08-09

**Authors:** Conghui Liu, Yan Zhang, Yuwei Ren, Hengchao Wang, Shuqu Li, Fan Jiang, Lijuan Yin, Xi Qiao, Guojie Zhang, Wanqiang Qian, Bo Liu, Wei Fan

**Affiliations:** 1 Agricultural Genomics Institute, Chinese Academy of Agricultural Sciences, Pengfei Road Shenzhen, Guangdong, 518120, China; 2 BGI-Shenzhen, Shenzhen, Guangdong, 518083, China

**Keywords:** golden apple snail, *Pomacea canaliculata*, genome, adaptive evolution, stress tolerance, P450, reproduction, perivitelline, metagenome

## Abstract

**Background:**

The golden apple snail (*Pomacea canaliculata*) is a freshwater snail listed among the top 100 worst invasive species worldwide and a noted agricultural and quarantine pest that causes great economic losses. It is characterized by fast growth, strong stress tolerance, a high reproduction rate, and adaptation to a broad range of environments.

**Results:**

Here, we used long-read sequencing to produce a 440-Mb high-quality, chromosome-level assembly of the *P. canaliculata* genome. In total, 50 Mb (11.4%) repeat sequences and 21,533 gene models were identified in the genome. The major findings of this study include the recent explosion of DNA/hAT-Charlie transposable elements, the expansion of the P450 gene family, and the constitution of the cellular homeostasis system, which contributes to ecological plasticity in stress adaptation. In addition, the high transcriptional levels of perivitelline genes in the ovary and albumen gland promote the function of nutrient supply and defense ability in eggs. Furthermore, the gut metagenome also contains diverse genes for food digestion and xenobiotic degradation.

**Conclusions:**

These findings collectively provide novel insights into the molecular mechanisms of the ecological plasticity and high invasiveness.

## Background

The golden apple snail *Pomacea canaliculata* (family Ampullariidae, order Architaenioglossa) is a freshwater snail listed among the world's top 100 worst invasive species [1] and is considered an agricultural and quarantine pest worldwide [2]. Native to tropical and subtropical South America, *P. canaliculata* gradually spread to nonindigenous regions, such as Southeast and East Asia [3], Africa [4], North America [5], Oceania [6], and even Europe [7]. Its successful biological invasion was closely related to its polyphagous feeding habits [8], voracious appetite [9], broad environmental adaptability [10], and rapid growth and high rate of reproduction [11]. In addition to its ecological impact, *P. canaliculata* ravages a wide range of crops, including grains, fruits, and vegetables [12], causing severe economic losses each year as a result of yield loss, replanting cost, and expenditures on control [13]. More seriously, *P. canaliculata* has been involved in the transmission of a fatal human disease, eosinophilic meningitis, which first appeared in East Asia where people frequently consume these snails [14]. During this pathophoresis, *P. canaliculata* acts as an important intermediate host of the pathogenic parasite *Angiostrongylus cantonensis*, and the range of infected regions is still expanding, creating a great challenge in terms of human health [15, 16].

Molluscs are a highly diverse group, second only to arthropods in species number [17], and their high biodiversity makes them an excellent model to address issues such as biogeography, adaptability, and evolutionary processes [18]. The worldwide invasive species *P. canaliculata* provides valuable potential in these fields [19]. As a primitive circumtropical species, *P. canaliculata* possesses strong ecological plasticity with many advantages, including low-temperature resistance [20] and drought tolerance [21], which has contributed to its competitive success in resource acquisition. *Pomaceacanaliculata* has been reported to establish populations at temperatures ranging from 10°C to 35°C [20, 22]. Additionally, *P. canaliculata* tolerates heavy metal contamination. When living in contaminated water, the gill is enriched with a high concentration of heavy metals, and histopathological changes in the digestive tract are detected; however, an extremely low mortality rate is observed [23]. The conspicuous coloration and neurotoxic lectin could confer a survival advantage on the eggs, defending the embryos against potential predators [24]. Moreover, an immune-neuroendocrine system can also be detected in *P. canaliculata*, as demonstrated by the existence of a specific immune memory after bacterial challenge [25, 26], broadening the study of invertebrate immunology.

The rich phenotypic and genetic diversity of molluscs makes them an excellent species group for addressing many important issues in evolution, ecology, and function. However, the genomic resources on Mollusca are still insufficient compared with those of other close phyla, such as Arthropoda and Nematoda, and few molluscs can be used as model organisms. *Pomacea canaliculata*, however, possesses the potential to be a model organism among molluscs because of several inherent characteristics. For example, *P. canaliculata* is easy to acquire because it has a broad global distribution originating from a primarily circumtropical environment. Moreover, its high adaptability, rapid growth, and efficient reproduction facilitate the cultivation of *P. canaliculata* in the laboratory.

In recent years, the genomic features of *P. canaliculata* have been increasingly studied. After the discovery of 14 pachytene bivalents in the karyotype [27], molecular markers were identified to investigate the genetic diversity of the *P. canaliculata* population, including 369 amplified fragment length polymorphism loci [28], 16,717 simple sequence repeats [29, 30], and 15,412 single-nucleotide polymorphisms [31]. In addition, multiple transcriptome analyses have been performed to investigate the adaptation, invasion, and immune mechanisms of *P. canaliculata*. For instance, Sun et al. reported 128,436 unigenes based on a *de novo* assembly of Illumina reads [31]; transcriptome changes in response to heat stress and starving incubation were used to characterize its invasive and adaptive abilities [32, 33]; a transcriptome analysis comparing invasive *P. canaliculata* and indigenous *Cipangopaludina cathayensis* provided insights into biological invasion [30]; and 402 immune-related differentially expressed genes (DEGs) in response to lipopolysaccharide challenge were used to explore the mechanisms of defense against pathogens [34]. Furthermore, proteomics tools such as isobaric tags for relative and absolute quantitation, and liquid chromatography-tandem mass spectrometry were also applied in the study of protein expression during estivation and oviposition [35, 36], together providing plentiful omics- data for the functional analysis of *P. canaliculata*.

However, research at the whole-genome level in *P. canaliculata* still lags far behind that in other mollusc species due to the lack of a high-quality reference genome. Multiple draft genomes of molluscs have been published, including the genomes of the California sea hare [37], Pacific oyster [38], pearl oyster [39, 40], owl limpet [41], California two-spot octopus [42], golden mussel [43], and *Biomphalaria* snails [44], greatly promoting research on mollusc genomics. In this study, we present a chromosome-level genome assembly of *P. canaliculata* with high-quality gene annotation, transcriptome data from several tissues and under various conditions, and metagenomic data from the intestinal tracts, all of which were then applied to study the species-specific environmental adaptation characteristics, such as the cellular homeostasis system underlying strong stress and the color and nutrient contents of the eggs. Our data will not only strengthen the understanding of the evolutionary mechanisms of molluscs and the molecular basis of biological invasion but also foster the development of approaches to control the invasion of *P. canaliculata* and provide a basis for interrupting the transmission of pathogenetic nematode parasites.

## Results

### Complete genome assembly at the chromosome level

**Figure 1:**
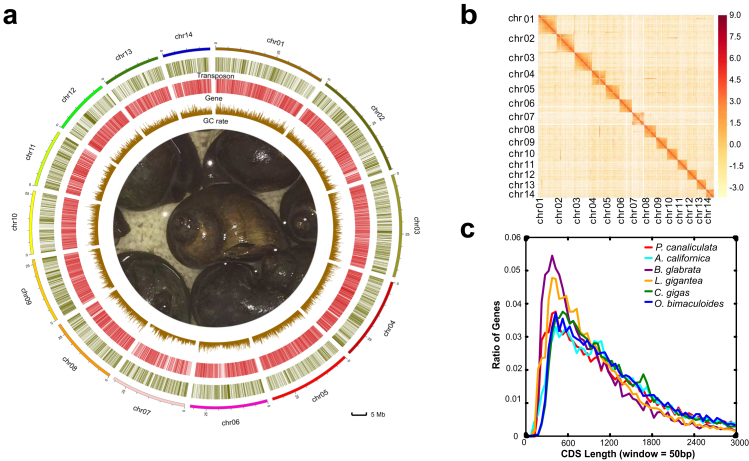
The genome characteristics of *P. canaliculata*. **(a)** Circos plot showing the genomic features. Track 1: 14 linkage groups of the genome; track 2: distribution of transposon elements in chromosomes; track 3: protein-coding genes located on chromosomes; track 4: distribution of Guanine and Cytosine (GC) contents. **(b)** A genome-wide contact matrix from Hi-C data between each pair of the 14 chromosomes using a 100-kb window size. The color value indicates the base 2 logarithm of the number of valid reads (log_2_[valid reads]). **(c)** Distribution of coding DNA sequence length in six closely related species.

We generated 26.6 Gb (60.1 X) of Pacific Biosciences (PacBio) single-molecule real-time (SMRT) raw reads with an average read length of 10.1 kb, and 291 Gb (652.4 X) of Illumina HiSeq paired-end reads with an average read length of 150–250 bp using DNA extracted from a single adult *P. canaliculata* (Supplementary Table S1). The 24.4 Gb (55.4 X) of clean PacBio SMRT reads that passed quality filtering were assembled using smartdenovo [45], resulting in an assembly of 1,234 raw contigs with a total length of 473.0 Mb and an N50 length of 1.0 Mb. After filtering of alternatively heterozygous contigs, the 745 resulting contigs with a total length of 440.1 Mb and an N50 length of 1.1 Mb were taken as the final contigs. Previous karyotype research has shown that the haploid *P. canaliculata* genome consists of 14 chromosomes [27]. Based on the Hi-C data, 439.5 Mb (99.9%) of final contigs were anchored and oriented into 14 large scaffolds, each corresponding to a natural chromosome (Fig. 1a and 1b), with the longest 45.4 Mb and the shortest 27.2 Mb. This assembly quality is much better than that of the other molluscan genomes published thus far (Table 1). In addition to the length and continuity of the assembled sequences, another important aspect for evaluating genome assembly is the ratio of genome coverage. With an estimated genome size of 446 Mb and genome heterozygosity between 1% and 2% based on the distribution of *k*-mer frequency [46] (Supplementary Fig. S1), ∼98.6% of the *P. canaliculata* genome has been assembled. To further confirm the accuracy and completeness of the assembly, we mapped the Illumina shotgun reads to the assembled reference genome. Significantly, 97% and 95% of the genome-derived and transcriptome-derived reads, respectively, could be aligned to the reference genome, suggesting no obvious bias in sequencing and assembly. Additionally, the mitochondrial genome of *P. canaliculata* was assembled as a single contig 15,707 bp in length, which has 99.9% sequence identity to the published mitochondrial genome (GenBank: KJ739609.1) (Supplementary Fig. S2). This high-quality reference genome provides a good foundation for gene annotation.

**Table 1:** Summary of assembly and annotation of mollusc genomes

Genome feature	*P. canaliculata*	*L. gigantea*	*A. californica*	*B. glabrata*	*C. gigas*	*O. bimaculoides*
Assembled sequences (bp)	440,071,717	359,505,668	927,310,431	916,377,450	557,735,934	2,381,887,882
Contig N50 size (bp)	1072,857	94,165	9,817	18,978	37,218	5,982
Contig N90 size (bp)	303,904	10,180	1,626	5,132	11,109	1,606
Scaffold N50 size (bp)	31,531,291	1870,055	917,541	48,059	401,685	475,182
Scaffold N90 size (bp)	23,662,357	74,480	207,390	817	68,181	79,088
GC content (%)	40.3	33.3	40.3	36.0	33.4	36
No. of gene models	21,533	23,824	19,909	14,224	28,402	15,814
Avg. coding DNA sequence length (bp)	1,497	1,136	1,568	1,066	1,472	1,535
Benchmarking Universal Single-Copy Ortholog (%)	98.9	98.4	98.7	72.8	99.4	98.7
Transposable elements (bp)	49,579,006	37,369,817	202,174,499	189,550,886	103,381,274	737,398,096
Tandem repeat (bp)	873,801	257,674	8263,822	2145,821	590,907	62,633,792

The protein-coding genes were predicted on the reference genome by EVM, integrating evidence from *de novo* prediction, transcriptome, and homology data. In total, 21,533 gene models were predicted as the reference gene set, with coding regions spanning ∼32.2 Mb (7.3%) of the genome (Table 1 and Supplementary Table S2). The distribution of coding DNA sequence Coding sequence (CDS) length in *P. canaliculata* is similar to that in closely related species (Fig. 1c). Overall, 97.5% of the reference genes were supported by transcriptome data, and 98.0% of eukaryote core genes from OrthoDB [47] were identified in the reference gene set by Benchmarking Universal Single-Copy Ortholog (BUSCO). These results were comparable to those in other published molluscan genomes (Table 1). In functional annotation, 19,815 (91.9%) reference genes were annotated by at least one functional database. Specifically, 15,662 (72.7%), 13,769 (63.4%), 17,081 (79.3%), 18,847 (87.5%), and 17,003 (79.9%) reference genes were annotated with the eggNOG, Kyoto Encyclopedia of Genes and Genomes (KEGG), NR, InterPro, and UniProt databases, respectively (Supplementary Fig. S3).

### Signs of adaptive evolution in *P. canaliculata* genome

**Figure 2:**
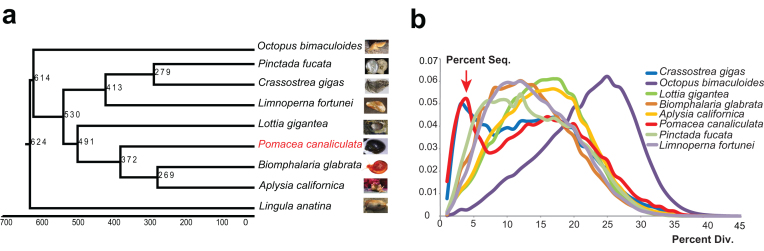
Evolutionary genomic analysis of *P. canaliculata*. **(a)** Phylogenetic placement of *P. canaliculata* within the dated tree of molluscs. The estimated divergence time is shown at each branching point, and *P. canaliculata* is shown in red. **(b)** Distribution of divergence rate for the class of DNA transposons in mollusc genomes. The divergence rate was calculated by comparing all transposable element (TE) sequences identified in the genome to the corresponding consensus sequence in each TE subfamily. The red arrow indicates that *P. canaliculata* and *C. gigas* had a recent explosion of TEs at a divergence rate of ∼4%.

To gain insight into the evolutionary perspective of *P. canaliculata*, a phylogenetic tree was built based on 306 high-confidence single-copy orthologous genes from nine related species (*P. canaliculata, Lottia gigantea, Aplysia californica, Biomphalaria glabrata, Crassostrea gigas, Octopus bimaculoides, Pinctada fucata, Lingula anatina*, and *Limnoperna fortunei*) by PhyML [48] and the divergence time was estimated using MCMCTree [49]. The results show that *P. canaliculata* diverged from the ancestor of *B. glabrata* and *A. californica* 372 million years ago (Mya) and from *L. gigantea* 491 Mya (Fig. 2a).

Then, the molluscan orthologous genes were investigated for adaptive evolution. Utilizing pairwise protein sequence similarities, gene family clustering was conducted using orthoFinder [50]. A total of 239,541 reference genes from the nine species were clustered into 69,582 orthologous groups, among which 14,766 orthologous groups contained at least two genes each. We identified 66 orthologous groups that underwent common expansion in both *P. canaliculata* and *L. fortunei* but not the other seven species. The functions of these orthologous groups are mainly related to signal transduction; replication and repair; translation, glycan biosynthesis, and metabolism; lipid metabolism; and the endocrine, immune, and nervous systems (Supplementary Fig. S4). These relations suggest that the gene families that underwent expansion may play important roles in adaptation to the environment as invasive species.

The high-coverage genome assembly enables a comprehensive analysis of the transposable elements (TEs), which play multiple roles in driving genome evolution in eukaryotes [51]. We identified 49.6 Mb TE sequences in the assembled *P. canaliculata* genome (Table 1), including 3.4 Mb long terminal repeats, 27.2 Mb long interspersed elements, 17.5 Mb DNA transposons, and 1.5 Mb short interspersed elements. Next, we analyzed the divergence rate of each class of TEs among the available sequenced mollusc genomes. Notably, the TE class of DNA transposons showed a specific peak at a divergence rate of ∼4% divergence rate for *P. canaliculata* and *C. gigas* (Fig. 2b), indicating a recent explosion of DNA transposons in these two species. We analyzed the expression of 709 genes, including DNA elements restricted to the 4% peak inside the gene region, compared with that of the other genes outside the 4% peak (Supplementary Fig. S5). DEGs were defined here by *P* values smaller than 0.05 for comparison of the treatment (heat, cold, heavy metal, and air exposure) and control data. The percentages of DEGs in the 4% peak were higher than those of genes outside the peak (10.2% higher for heat, 8.6% higher for cold, 8.6% higher for heavy metal, and 7.3% higher for air exposure). Among the DEGs in the 4% peak, approximately half were upregulated, and the other half were downregulated. Moreover, the DEGs in the 4% peak were mainly enriched in cellular metabolic process, response to stimulus, localization, and signaling according to Gene Ontology (GO) annotation. These results indicated that genes in the 4% peak were likely to be more active in the response to stimulus, promoting potential plasticity in stress adaptation. TEs are powerful facilitators of evolution that generate “evolutionary potential” to introduce small adaptive changes within a lineage, and the importance of TEs in stress responses and adaptation has been reported in numerous studies [52, 53]. The recent explosion of DNA TEs in *P. canaliculata* could also play an important role in promoting the potential plasticity in stress adaptation.

### Investigation of cellular homeostasis system underlying strong stress adaptation

**Figure 3:**
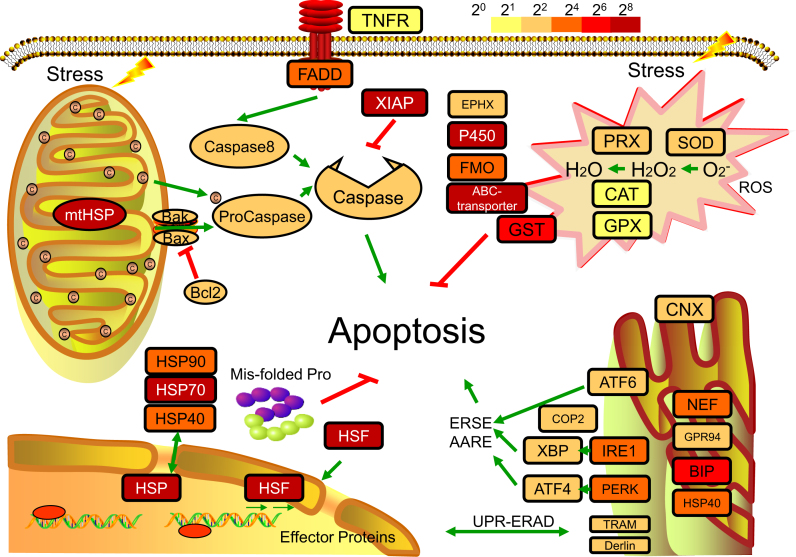
The cellular homeostasis system in *P. canaliculata*. The unfolded protein response (UPR) system includes HSPs and HSF in the heat shock response and CNX, NEF, GRP94, BIP, HSP40, ATF6, IRE1, PERK, COP2, XBP, ATF4, TRAM, and Derlin in the endoplasmic reticulum unfolded protein response (UPR-ERAD). Apoptotic pathways include XIAPs, Bcl2, caspases, TNFR, and FADD. The antioxidant systems include PRX, SOD, CAT, and GPX. The xenobiotic biotransformation system includes EPHX3, P450, FMO, and ABC transporter. The colors of the boxes for gene families represent the degree of upregulation (FPKM-stimulus/FPKM-control) as an overall result of stress, including heat, cold, heavy metal, and air exposure. Pathways and genes were obtained based on KEGG annotation.

The homeostasis system plays a crucial role in stress adaptability, providing the molecular basis for re-establishing dynamic equilibrium after challenges by various environmental stressors, including temperature, air exposure, anthropogenic pollution, and pathogens [54]. In this study, we addressed three constituent parts of the cellular homeostasis system that contribute to the successful ecological plasticity of *P. canaliculata* (Fig. 3). The transcriptomes of the hemocytes after different stimuli (cold, heat, heavy metal, and air exposure) were also sequenced and analyzed to address the potential roles of these genes in the cellular homeostasis system.

The unfolded protein response (UPR) system is the central component of protein homeostasis [55]. Heat shock proteins (HSPs) act as molecular chaperones to maintain correct folding, and heat shock transcription factor 1 (HSF1) is responsible for the transcriptional induction of HSPs [56]. In the *P. canaliculata* genome, 13 HSP70s, 6 HSP90s, 7 HSP40s, and 11 HSFs were identified (Supplementary Table S3), and the expression of HSP90s and HSFs was highly induced in response to heat, cold, heavy metal, and air exposure (Supplementary Table S4 and Fig. S6). Inositol-requiring enzyme 1 (IRE 1), protein kinase RNA-like ER kinase (PERK), and activating transcription factor 6 (ATF6) are three mediators recruited by the endoplasmic reticulum (ER) to regulate the UPR [57]. We found putative coding genes of the three core mediators, their respective downstream transcription factors, and the corresponding recognition chaperones in the *P. canaliculata* genome (Supplementary Table S3).

The xenobiotic biotransformation system helps the molluscs adapt to toxicants, especially pesticides in aquatic environments [58]. Manual annotation of this genome identified 157 cytochrome P450s (CYP450s), 15 flavin-containing monooxygenases (FMOs), 53 glutathione S-transferases, and 105 ATP binding cassette (ABC) transporters, most of which showed upregulated expression under stress (Supplementary Tables S3 and S4). These proteins have been shown to function in contaminant detection, conjugative modification, and expulsion for xenobiotic detoxification [59-61].

The massive production of reactive oxygen species (ROS) and reactive oxygen intermediates induced by stress leads to many pathological conditions, and antioxidant systems protect the organism from superoxide [62]. Four main antioxidant enzyme classes, namely, superoxide dismutase (SOD), catalase (CAT), peroxidase (PRX), and glutathione peroxidase (GPX), were found in *P. canaliculata* and showed elevated global expression in response to stress (Supplementary Tables S3 and S4).

Apoptosis is a process of cell death when sensing stress, and the regulation of apoptosis contributes to the dynamic homeostasis of the internal environment. In *P. canaliculata*, we propose the existence of both intrinsic and extrinsic apoptotic signaling pathways, evidenced by the presence of homologous genes involved in both pathways. These two pathways could be activated by cytochrome C and tumor necrosis factor receptor (TNFR), respectively (Supplementary Table S3). Inhibitors of apoptosis, such as XIAP, Bcl2, and Bak, are also detected and show increased expression in response to stress (Supplementary Table S4), which is expected to delay the process of apoptosis and cell death in the stress response.

### Expansion of the P450 gene family contributes to stress tolerance

Cytochrome P450 (CYP) enzymes are a monooxygenase family with highly diverse structures and functions that have been widely identified in all kingdoms of life [63]. P450s catalyze the reductive scission of molecular oxygen and are responsible for the synthesis and metabolism of various molecules, including drugs, hormones, antibiotics, pesticides, carcinogens, and toxins [64]. The hormones they synthesize, such as glucocorticoids, mineralocorticoids, progestins, and sex hormones, are critical to stress response, growth, and reproduction, and the endogenous and exogenous chemical metabolism participate in combatting toxic compounds [65].

**Figure 4:**
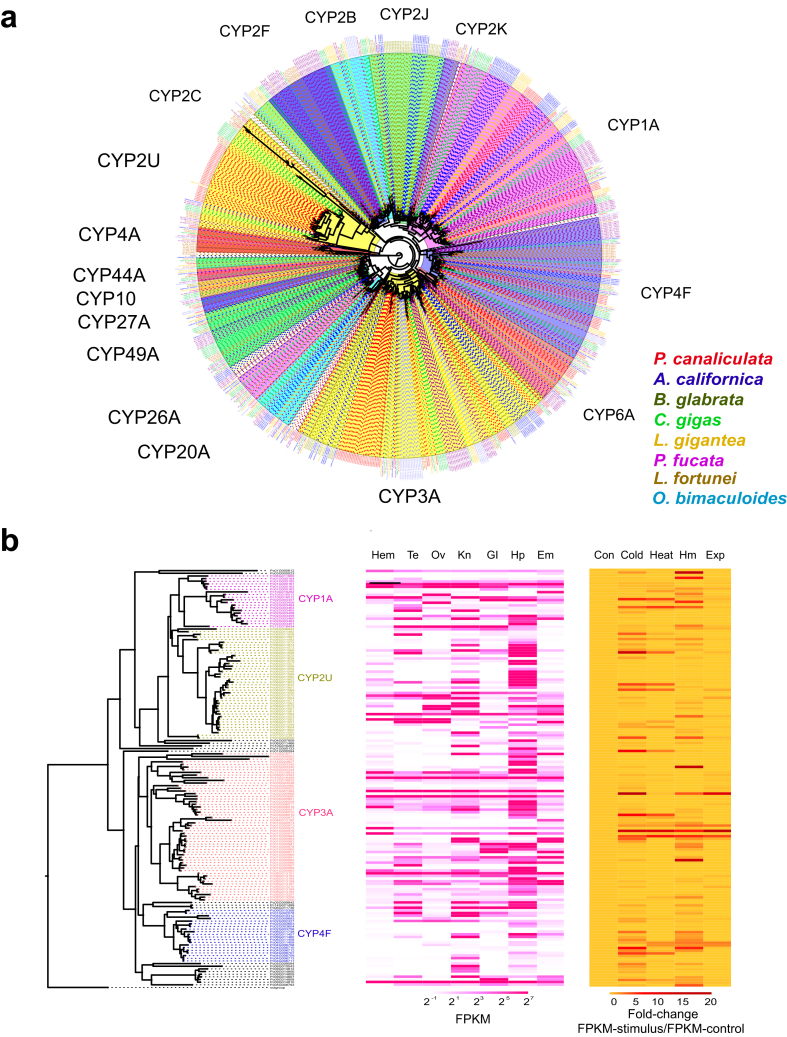
The expansion of the P450 gene family in *P. canaliculata*. **(a)** Phylogenetic tree demonstrating orthologous and paralogous relationships of all P450 genes from eight species including *P. canaliculata*, *A. californica*, *B. glabrata*, *C. gigas*, *L. fortunei, L. gigantea*, *O. bimaculoides*, and *P. fucata*. P450 genes from eight species were obtained based on Pfam annotation (Interpro) with an E-value of 10^−5^. Clades are labeled by P450 subfamily names. The tree was constructed using the maximum likelihood method in MEGA7, and the branch length scale indicates the average number of residue substitutions per site. **(b)** Phylogenetic tree of P450 genes in *P. canaliculata*, which is a subset of the phylogenetic tree for the species, and their heat map of expression (FPKM) in tissues (Hem, hemocytes; Te, testis; Ov, ovary and albumen gland; Kn, kidney; Gl, gill; Hp, hepatopancreas; Em, embryo) and heat map of induced expression (FPKM-stimulus/FPKM-control) under stress (Con: control; heat; cold; Hm: heavy metal; Exp: air exposure).

We found that the *P. canaliculata* CYP gene family had undergone an expansion compared to that in the other molluscs. We identified 157 genes in the genome of *P. canaliculata* and 128, 102, 135, 115, 78, 52, and 94 genes in *A. californica, B. glabrata, C. gigas, L. fortunei, L. gigantea, O. bimaculoides*, and *P. fucata*, respectively, using the same standard (Fig. 4a). An expansive trend was also observed in comparison with other model species, such as *Homo sapiens* (57), *Mus musculus* (102), *Danio rerio* (94), and *Drosophila melanogaster* (94) [66]. Gene expansion was mainly found in the CYP2U and CYP3A subfamilies, whereas fewer genes were expanded in CYP4F. In mammals, CYP2U participates in the metabolism of fatty acids to generate bioactive eicosanoid derivatives, potentially regulating the development of immune function [67]. In *P. canaliculata*, 40 genes formed the CYP2U clade, mainly expressed in the hepatopancreas (Fig. 4b and Supplementary Table S5a, S5b). CYP3A is a versatile enzyme that metabolizes a wide range of xenobiotics, and its production promotes the growth of various cell types [68]. The 56 CYP3A genes are comprehensively expressed in the hepatopancreas, gill, and kidney (Fig. 4b and Supplementary Table S5a, S5b). CYP4F possesses epoxygenase activity, metabolizing fatty acids to epoxides to suppress hypertension, pain perception, and inflammation [69]. Twenty genes were identified in CYP4F, and Pc06G011748, Pc06G011460, Pc06G011458, Pc06G011459, Pc04G006708, Pc04G006710, and Pc04G006707 exhibited highly induced expression levels under cold, heat, heavy metal, and air exposure stress, indicating their critical roles in the stress tolerance (Fig. 4b and Supplementary Table S5a and S5b).

### Identification of perivitelline genes and their high transcriptional levels in the ovary and albumen gland

**Figure 5:**
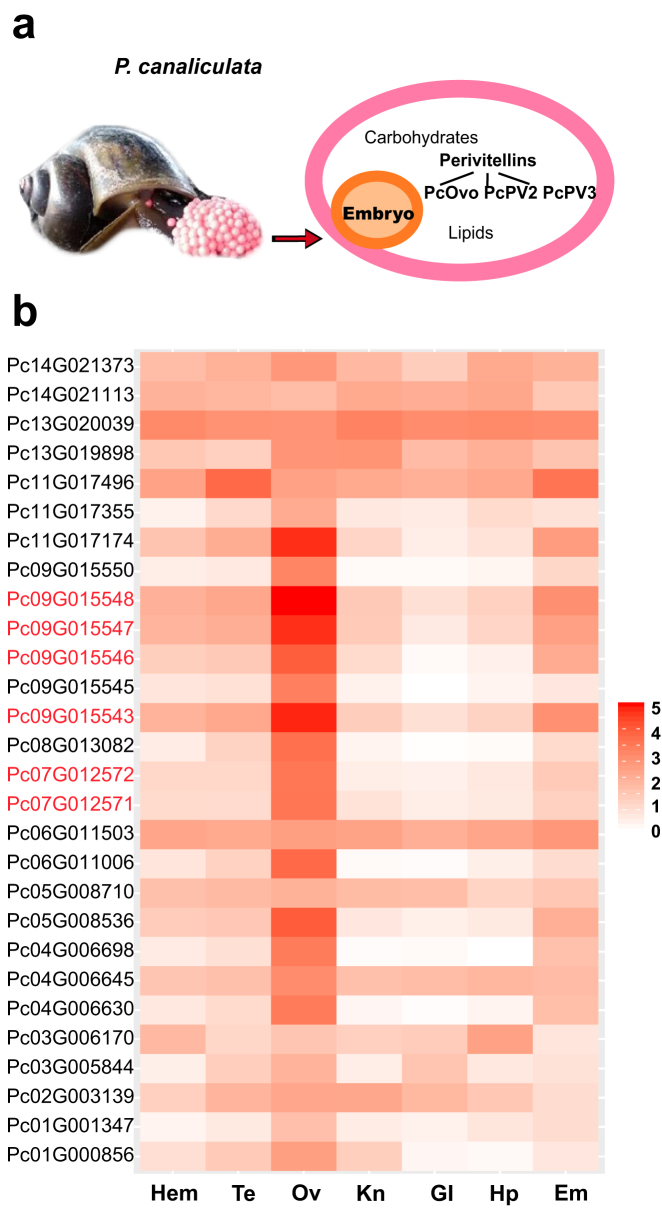
The composition and expression of the *P. canaliculata* perivitellines in different tissues. **(a)** Perivitelline fluid (PVF) lies under the eggshell and surrounds the embryo. It contains carbohydrates, lipids, and proteins. The proteins are also known as perivitellines and are classified into three categories, PcOvo, PcPV2, and PcPV3. **(b)** The displayed expression value of PVF proteins is the base 10 logarithm of FPKM (log_10_FPKM). The genes marked in red encode perivitellines. The tissues examined are abbreviated as follows: Hem, hemocytes; Te, testis; Ov, ovary and albumen gland; Kn, kidney; Gl, gill; Hp, hepatopancreas; Em, embryo.

*Pomacea canaliculata* has eggs characterized by abundant nutrients, reddish or pinkish color, aerial oviposition, and neurotoxicity [24, 70] due to the perivitelline fluid (PVF), which fills the space between the eggshell and the embryo and consists of carbohydrates, lipids, and proteins (Fig. 5a). The PVF proteins in *P. canaliculata* include three major components, PcOvo, PcPV2, and PcPV3 [71], collectively named perivitellines, which make up 90% of the total proteins, whereas most of the other dozens of low-abundance components each account for less than 1% of the total proteins [36]. The perivitellines are not only responsible for the major supply of materials and energy during embryogenesis but also provide warning pigments and deadly toxicants against predators [24, 72, 73].

We identified 28 candidate PVF genes in *P. canaliculata* by mapping each of the 59 fragmental PVF protein sequences derived from a previous proteomics study by Sun [36] to its best hit in the reference gene set of *P. canaliculata*, using Basic Local Alignment Search Tool for proteins (BLASTP) with requirements of over 85% identity and at least 50% alignment length (Supplementary Table S6). Then, the functional annotation of those fragmental proteins was also transferred to our identified PVF genes. The transcriptome data show that 22 (79%) of the 28 candidate PVF genes exhibit their highest expression in the ovary and albumen gland (PVF protein synthesis factory) among all seven tissues (Fig. 5b and Supplementary Table S7), confirming that most of them are genuine functional PVF genes. Six of these 28 candidate PVF genes are perivitelline genes, including two PcOvo genes, Pc09G015543 (PcOvo2) and Pc09G015548 (PcOvo3); two PcPV2 genes, Pc07G012572 (PcPV2-31) and Pc07G012571 (PcPV2-67); and two possible PcPV3 genes, Pc09G015546 and Pc09G015547. The expression levels of these six genes in the ovary and albumen gland are much higher than those of the other 22 candidate PVF genes.

By analyzing the orthoFinder gene families that include orthologous and paralogous genes from *P. canaliculata* and eight other sequenced mollusc species, we found that these 28 candidate PVF genes were classified into 20 multiple-gene families (two or more genes) and seven single-gene families (only one gene) (Supplementary Table S8). Notably, five of the six perivitelline genes were classified into single-gene families, except for Pc07G012571 (PcPV2-67), which not only has homologous genes in other mollusc species but also has three paralogous genes in *P. canaliculata* itself. However, none of these three PcPV2-67 paralogous genes in *P. canaliculata* showed higher expression in the ovary and albumen gland than in other tissues, indicating that they are likely not PVF-related genes, i.e., only Pc07G012571 plays a role in PVF. The nearly unique and single-copy nature of the six perivitelline genes in *P. canaliculata* may be explained by the long evolutionary distance, more than 200 Mya for *P. canaliculata* and its most closely related species, *A. californica*, as well as numerous differences in their living characteristics and egg structures. Another possible explanation is that these six major PVF genes may have experienced rapid evolution in their history to adapt to the changing environment.

### The gut microbiome plays important roles in stress resistance and food digestion

The gut microbiome is regarded as the “second genome” of the host animal due to the fact that gut microbiota contributes to the food digestion, immune system development, and many other processes important to the host. To investigate the relationship between the gut microbiome and the invasive lifestyle of *P. canaliculata*, we collected gut digesta samples from 70 *P. canaliculata* snails and generated 31 Gb of high-quality metagenomic data on the Illumina HiseqX10 platform. To our knowledge, this study is the first in-depth sequencing of the snail gut microbiome. A total of 1,142,095 nonredundant (NR) genes were obtained with an average ORF length of 604 bp (Supplementary Table S9). The taxonomic composition analysis showed that at the phylum level, Proteobacteria was predominant, followed by Verrucomicrobia, Bacteroidetes, Firmicutes, Spirochaetes, and Actinobacteria (Supplementary Table S10a). At the genus level, the most abundant genera included *Aeromonas*, *Enterobacter*, *Desulfovibrio*, *Citrobacter*, *Comamonas*, *Klebsiella*, and *Pseudomonas* (Supplementary Table S10b), most of which were also present in *Achatina fulica* [74, 75].

Interestingly, some of the most abundant genera, such as *Desulfovibrio*, *Citrobacter*, and *Pseudomonas*, were reported as having strong abilities to remove heavy metals by bioprecipitation and bioabsorption [76-78]. For example, the sulfur-reducing bacteria *Desulfovibrio* produce hydrogen sulfide, which precipitates metals and therefore reduces the toxic effects of dissolved metals [76]. Based on the KEGG pathway database, the complete sulfate reduction metabolism pathway was identified in the *P. canaliculata* gut microbiome. We suggested that these gut microbes might help *P. canaliculata* survive the environmental stress of heavy metals in harsh conditions. In addition, a large number of genes in xenobiotic biodegradation and metabolism pathways were annotated, corresponding to 288 KEGG orthologous groups (KOs) and 21 pathways (Supplementary Table S11). As many of the pathways, such as benzoate degradation, toluene degradation, xylene degradation, and steroid degradation, could not be identified in the host genome through KO analysis, we suggested that microbial detoxification abilities may contribute to the ability of *P. canaliculata* to resist stresses caused by xenobiotics such as pesticides and environmental pollutants.

In digestion, the gut microbes are directly involved in the breakdown of the cellulose portion of the diet, and previous studies have isolated cellulolytic bacteria and evaluated the cellulolytic enzyme activities [79]. in our work we found a broader range of carbohydrate active enzymes (CAZymes). Of the 208 annotated CAZyme families, 99 were glycoside hydrolase families (Supplementary Table S12). Enzymes that could be classified as cellulases, endohemicelluloses, debranching enzymes, and oligosaccharide-degrading enzymes were all identified. These findings indicate that the gut microbiome provides assistance in digesting a broad range of food sources, enabling *P. canaliculata* to grow rapidly and adapt to an invasive lifestyle.

## Conclusions

Given its environmental invasiveness, broad stress adaptability, and rapid reproduction, the golden apple snail *P. canaliculata* has received a vast amount of attention worldwide. However, the underlying genetic mechanisms of these properties have not been comprehensively uncovered. The chromosome-level genome of *P. canaliculata* presented in this study sheds the first light on the genomic basis of its ecological plasticity in response to various stressors. The major findings of this study include the recent explosion of DNA/hAT-Charlie TEs, the expansion of the P450 gene family, and the constitution of the cellular homeostasis system, all of which contribute to the plasticity of the organism in stress adaptation. Although the function of the recently originated TEs could not be confirmed, TEs are considered powerful facilitators in adaptive evolution, suggesting that their increased numbers play an important role in the stress resistance of *P. canaliculata*. The UPR system, xenobiotic biotransformation system, and ROS system are all major components of the cellular homeostasis system, and the P450s in particular underwent expansion with specific functions. In addition, exclusive perivitelline genes were identified in the *P. canaliculata* genome, and they are believed to contribute to the high reproductive rate and the expansion of habitats. Furthermore, the gut metagenome contains diverse genes for food digestion and xenobiotic degradation. These findings collectively provide novel insight into the molecular mechanisms of ecological plasticity and high invasiveness.

In this study, we report a fine reference genome of *P. canaliculata*, the first chromosome-level Mollusca genome published. With widespread distribution, rapid growth, and efficient reproduction, *P. canaliculata* possesses the potential to be a model organism of Mollusca. As its cellular complexity and conservation of pathways also make *P. canaliculata* a useful representative of Mollusca, the genome described in this study can be used to advance our understanding of the molecular mechanisms involved in various scientific questions regarding Mollusca.

## Methods

### Sample collection and sequencing

Adult *P. canaliculata* were collected from a local paddy field in Shenzhen, Guangdong province, China, and maintained in aerated freshwater at 15 ± 2°C for a week before processing. Genomic DNA was extracted from the foot muscles of a single *P. canaliculata* for constructing polymerase chain reaction-free Illumina 350-bp insert libraries and PacBio 20-kb insert library and sequenced on Illumina HiSeq 2500 and PacBio SMRT platforms, respectively. The Hi-C library was prepared using the muscle tissue of another single *P. canaliculata* by the following methods: nuclear DNA was cross-linked *in situ*, extracted, and then digested with a restriction enzyme. The sticky ends of the digested fragments were biotinylated, diluted, and then ligated to each other randomly. Biotinylated DNA fragments were enriched and sheared again for preparing the sequencing library, which was then sequenced on a HiSeq X Ten platform (Illumina).

Seven tissues including embryos (2 days post-fertilization), gill, hemocytes, hepatopancreas, kidney, ovary and albumen gland, and testis from six animals were collected as parallel samples. Next, animals were cultivated at 37°C and 10°C for 24-hour heat and cold tolerance; in Cr^3+^(2 mg/L), Cu^2+^(0.2 mg/L), and Pb^2+^(1 mg/L) for 24-hour heavy metal tolerance, and in a waterless tank for 7 days air exposure. Then, the hemocytes were harvested and stored, with three replicates for each group. Total RNAs were extracted from the stored tissues of *P. canaliculata* materials. Then, mRNAs were pulled out by beads with poly-T for constructing cDNA libraries (insert 350-bp) and sequenced on an Illumina HiSeq 2500 sequencer.

The intestinal digesta from 70 adult snails of *P. canaliculata* were collected, pooled into six samples, and stored at −20°C until microbial DNA was extracted. A combination of cell lysis treatments was applied, including five freeze-thaw cycles (alternating between 65°C and liquid nitrogen for 5 minutes), repeated beads-beating in A stool lysis (ASL) buffer (cat. no. 19 082; Qiagen Inc.), and incubated at 95°C for 15 minutes. DNA was isolated following the reported protocol [80]. Paired-end libraries of metagenomic DNA were prepared with an insert size of 350 bp following the manufacture's protocol (cat. no. E7645L; New England Biolabs). Sequencing was performed on Illumina HiSeq X10.

### Genome assembly and annotation

The Illumina raw reads were filtered by trimming the adapter sequence and low-quality regions [81], resulting in clean and high-quality reads with an average error rate of <0.001. For the PacBio raw data, the short subreads (<2 kb) and low-quality (error rate >0.2) subreads were filtered out, and only one representative subread was retained for each PacBio read. The clean PacBio reads were assembled by the software smartdenovo [82], after which Illumina reads were aligned to the contigs by BWA-MEM (BWA, RRID:SCR_010910), and single base errors in the contigs were corrected by Pilon v1.16 (Pilon, RRID:SCR_014731) with the parameters “-fix bases, -nonpf, -minqual 20.” The *P. canaliculata* genome is highly heterozygous, as illustrated by the double peaks on the distribution curve of *k*-mer frequency, and the current assembly algorithm tends to collapse homozygous regions and report heterozygous regions in alternative contigs. To obtain a haploid reference contig, we employed a whole-genome alignment strategy with MUMmer v3.23 to recognize and selectively remove alternative heterozygous contigs, which were characterized by shorter length (less than 200 kb) and the ability of most regions (more than 50%) to be aligned to another larger contig with confident identity (higher than 80%). Next, Hi-C sequencing data were aligned to the haploid reference contigs by BWA-MEM, and then these contigs were clustered into chromosomes with LACH-ESIS [83].

A *de novo* repeat library for *P. canaliculata* was constructed by RepeatModeler v. 1.0.4 (RepeatModeler, RRID:SCR_015027; [84]). TEs in the *P. canaliculata* genome were also identified by RepeatMasker v4.0.6 (RepeatMasker, RRID:SCR_012954; [85]) using both the Repbase library and the *de novo* library. Tandem repeats in the *P. canaliculata* genome were predicted using Tandem Repeats Finder v4.07b [86]. The divergence rates of TEs were calculated between the identified TE elements in the genome and their consensus sequence at the TE family level.

The gene models in the *P. canaliculata* genome were predicted by EVidence Modeler v1.1.1 [87], integrating evidence from *ab initio* predictions, homology-based searches, and RNA sequencing (RNA-seq) alignments. Then, these gene models were annotated by RNA-seq data, UniProt database, and InterProScan software (InterProScan, RRID:SCR_005829) [88]. Finally, the gene models were retained if they had at least one piece of supporting evidence from the UniProt database, InterProScan domain, and RNA-seq data. Gene functional annotation was performed by aligning the protein sequences to the National Center for Biotechnology Information (NCBI) NR, UniProt, COG, and KEGG databases with BLASTp v2.3.0+ under an E-value cutoff of 10^−5^ and choosing the best hit. Pathway analysis and functional classification were conducted based on the KEGG database [89]. InterProScan was used to assign preliminary GO terms, Pfam domains and IPR domains to the gene models.

### Evolutionary analysis

Orthologous and paralogous groups were assigned from nine species (*P. canaliculata, Lottia gigantea, Aplysia californica, Biomphalaria glabrata, Crassostrea gigas, Octopus bimaculoides, Pinctada fucata, Limnoperna fortunei*, and *Lingula anatina*) by OrthoFinder [50] with default parameters. Orthologous groups that contained only one gene for each species were selected to construct the phylogenetic tree. The protein sequences of each gene family were independently aligned by muscle v3.8.31 [90] and then concatenated into one super-sequence. The phylogenetic tree was constructed by maximum likelihood (ML) using PhyML v3.0 (PhyML, RRID:SCR_014629) [48] with the best-fit model (LG+I+G) estimated by ProtTest3 [91]. The Bayesian relaxed molecular clock approach was adopted to estimate the neutral evolutionary rate and species divergence time using the program MCMCTree, implemented in the PAML v4.9 package (PAML, RRID:SCR_014932) [49]. The tree was calibrated with the following time frames to constrain the age of the nodes between the species: minimum = 260 Mya and maximum = 290 Mya for *P. fucata* and *C. gigas* [92]; minimum = 450 Mya and maximum = 480 Mya for *A. californica* (or *B. glabrata*) and *L. gigantea* [93]. The calibration time (fossil record time) interval (550–610 Mya) of *O. bimaculoides* was adopted from previous results [94]. To identify the common expanded gene families, we compared the *P. canaliculata* and *L. fortunei* with the other seven species. The gene numbers of the orthologous group in *P. canaliculata* and *L. fortunei* were two or more times that in all of other species, respectively. Additionally, these gene families with *P* value less than 0.01 were considered as expansion by z-test.

### Transcriptome data analysis

Transcriptome reads were trimmed using the same method for genomic reads [81 and then mapped to the reference genome of *P. canaliculata* using TopHat v. 2.1.0 (TopHat, RRID:SCR_013035) with default settings. The expression level of each reference gene in terms of FPKM was computed by cufflinks v2.2.1 (cufflinks, RRID:SCR_014597). A gene was considered to be expressed if its FPKM was >0. Differential gene expression analysis was conducted using cuffdiff v2.2.1.

### Metagenome data analysis

The Illumina raw reads were filtered by trimming the adapter sequence and low-quality regions [81], resulting in high-quality reads with an average error rate of <0.001. Then, the reads mapped to the following genomes by BWA-MEM were filtered out [95] [96] to exclude the contaminated host, food, parasite, and human DNA sequences. The genomes include the *P. canaliculata* genome, the *Brassica rapa* genome, the *Oryza sativa* genome, two *Angiostrongylus cantonensis* genomes, the *Caenorhabditis elegans* genome, the *Schistosoma mansoni* genome, the *Clonorchis sinensis* genome, the *Fasciola hepatica* genome, the *Danio rerio* genome, and the *Homo sapiens hg38* genome. Finally, short reads (length < 75 bp) and unpaired reads were excluded to form a set of clean reads.

The clean reads were assembled by metaSPAdes (v3.11.1) [97] in paired-end mode for each sample. Then, gene prediction was performed on contigs longer than 500 bp by Prodigal v2.6.3 (Prodigal, RRID:SCR_011936) [98] with the parameter “-p meta,” and gene models with cds length less than 102 bp were filtered out. An NR gene set (539,344 genes) was constructed using the gene models predicted from each sample by cd-hit-est (v4.6.6) [99] with the parameter “-c 0.95 -n 10 -G 0 –a S 0.9,” which adopts a greedy incremental clustering algorithm and the criteria of identity >95% and overlap >90% of the shorter genes. Then, the clean reads were mapped onto this NR gene set by BWA-MEM with the criteria of alignment length ≥50 bp and identity >95%. The unmapped reads from all samples were assembled together, and the genes were predicted again. The newly predicted genes were combined with the previous gene set by cd-hit-est to obtain a new NR gene set (1,147,339 genes). After the taxonomic assignments to the new NR gene set, 5,244 genes classified as Eukaryota but not fungi were removed, and the final NR gene set (1,142,095 genes) was obtained.

The taxonomic assignments of the final NR genes were made on the basis of DIAMOND (DIAMOND, RRID:SCR_016071) [100] protein alignment against the NCBI NR database by CARMA3 [101]. Functional annotation was performed by aligning all the protein sequences to the KEGG [102] database (release 79) using DIAMOND and taking the best hit with the criteria of E-value <1e-5. CAZymes were annotated with dbCAN (release 5.0) [103] using Hmmer v3.0 hmmscan (Hmmer, RRID:SCR_005305) [104] by taking the best hit with an E-value <1e-18 and coverage > .35.

The clean reads from each sample were aligned against the gene catalogue (1,142,095 genes) by BWA-MEM with the criteria of alignment length ≥50 bp and identity >95%. Sequence-based gene abundance profiling was performed as previously described [105]. The taxonomic profiles of the samples were calculated by summing the gene abundance according to the taxonomic assignment result.

## Supplementary Material

GIGA-D-18-00030_(Original_Submission).pdf

GIGA-D-18-00030_Revision_1.pdf

GIGA-D-18-00030_Revision_2.pdf

Response_to_Reviewer_Comments_Original_Submission.pdf

Response_to_Reviewer_Comments_Revision_1.pdf

Reviewer_1_Report_(Original_Submission) -- Takeshi Takeuchi, PhD 3/12/2018 Reviewed

Reviewer_1_Report_(Revision_1) -- Takeshi Takeuchi, PhD 5/25/2018 Reviewed

Reviewer_2_Report_(Original_Submission) -- Mathew Jenny 3/17/2018 Reviewed

Reviewer_3_Original_Submission_(attachment).pdf

Reviewer_3_Report_(Original_Submission) -- Marcela Uliano da Silva, PhD 03/20/2018 Reviewed

Reviewer_3_Report_(Revision_1) -- Marcela Uliano da Silva, PhD 05/31/218 Reviewed

Reviewer_3_Revision_1_(attachment).pdf

Supplemental Tables and Figures

## Availability of supporting data

Supplementary Tables S1−S12 and Figs. S1− to S6 are available in the additional file. The raw sequencing data have been deposited in DDBJ/EMBL/GenBank under project accession PRJNA427478, SRR6425828 for genomic Illumina_PE125 sequencing data, SRR6425829 for genomic Illumina_PE150 sequencing data, SRR6425827 for genomic Pacific Biosciences sequencing data, SRR6429132∼SRR6429164 for transcriptome sequencing data, and SRR6472920∼SRR6472925 for gut microbiome data. Other supporting data, including genome assemblies, annotations, phylogenetic tree files, and Benchmarking Universal Single-Copy Ortholog results, are available via the *GigaScience* repository GigaDB [106].

## Abbreviations

ABC, ATP binding cassette; ATF6, activating transcription factor 6; BLASTp: Basic Local Alignment Search Tool for Proteins; CAT, catalase; CAZymes, carbohydrate active enzymes;CDS, coding sequences; CYP450s, cytochrome P450s; DEGs, differentially expressed genes; dRNA-seq: differential RNA sequencing; Em, embryo; ER, endoplasmic reticulum; FMOs, flavin-containing monooxygenases; GI, gill; GO: Gene Ontology; GPX, glutathione peroxidase; GSTs, glutathione S-transferases; Hem, hemocytes; Hp, hepatopancreas; HSF1, heat shock transcription factor 1; HSPs, heat shock proteins; Kn, kidney; KEGG, Kyoto Encyclopedia of Genes and Genomes; KOs, KEGG orthologous groups; LINE, long interspersed elements; Mya, million years ago; NCBI: National Center for Biotechnology Information; NR, nonredundant genes; ORF, open reading frame; Ov, ovary and albumen gland; Ovo, ovorubin; PERK, protein kinase RNA-like ER kinase; Prx, peroxidase; PS: processing site; PVF, pervitelline luid; RNA-seq: RNA sequencing; ROS, reactive oxygen species; SNP: single nucleotide polymorphism; SOD, superoxide dismutase; Te, testis; TEs, transposable elements; TNFR, tumor necrosis factor receptor; TSS, transcriptional start site; UPR, unfolded protein response; UTR, untranslated region: GC rate, guanine and cytocine rate; ASL buffer, a stool lysis buffer.
