# Supplementary material for: The genome of the golden apple snail Pomacea canaliculata provides insight into stress tolerance and invasive adaptation
Source: Gigascience. 2018 Aug 9;7(9):giy101. doi: 10.1093/gigascience/giy101 (PMC6129957; doi:10.1093/gigascience/giy101)
Supplement: GIGA-D-18-00030_(Original_Submission).pdf [file giy101_giga-d-18-00030_(original_submission).pdf]

GigaScience

The genome of golden apple snail Pomacea canaliculata provides insight into stress tolerance and invasive adaptation

--Manuscript Draft--

Manuscript Number:	GIGA-D-18-00030
Full Title:	The genome of golden apple snail Pomacea canaliculata provides insight into stress tolerance and invasive adaptation
Article Type:	Research
Funding Information:	National key research and development program of China (2016YFC1200600) — Dr Wei Fan<br>Shenzhen science and technology program (JCYJ20150630165133395) — Dr Wei Fan<br>Fund of Key Laboratory of Shenzhen (ZDSYS20141118170111640) — Dr Wei Fan<br>The Agricultural Science and Technology Innovation Program (ASTIP) of Chinese Academy of Agricultural Sciences(CAAS) & Elite Youth Program of Chinese Academy of Agricultural Sciences — Dr Wei Fan
Abstract:	Background: The golden apple snail (Pomacea canaliculata) is a worldwide fresh water snail listed in the top-100 worst invasive species, and a noted agricultural and quarantine pest causing huge economic loss, characterized with fast growth, strong stress tolerance, high reproduction rate, and adaptation to a broad range of environments.<br>Results: Here, we used long-read sequencing to produce a 440-Mb high-quality chromosome level assembly for P. canaliculata genome. In total, 50 Mb (11.4%) repeat sequences and 21,533 gene models were identified in the genome. Major findings of this study include the recent explosion of DNA/hAT-Charlie TEs, the expansion of P450 gene family and the constitution of cellular homeostasis system, contributing to the ecological plasticity in the stress adaptation. In addition, the perivitellin gene expansion and high transcriptional level in ovary promotes the function of nutrients supplying and defense ability in the eggs. Furthermore, the gut metagenome also encodes rich genes for food digestion and xenobiotics degradation.<br>Conclusions: These findings collectively provide novel insight into the molecular mechanisms of the ecological plasticity and high invasiveness. Our results not only strengthen the understanding of molluscs genomics and biological invasion, but also benefit preventing the invasion of apple snail and transmission of pathogenetic parasites.
Corresponding Author:	Wei Fan<br>Chinese Academy of Agricultural Sciences<br>CHINA
Corresponding Author Secondary Information:	
Corresponding Author's Institution:	Chinese Academy of Agricultural Sciences
Corresponding Author's Secondary Institution:	
First Author:	Conghui Liu
First Author Secondary Information:	
Order of Authors:	Conghui Liu<br>Bo Liu<br>Yuwei Ren<br>Yan Zhang

Powered by Editorial Manager® and ProduXion Manager® from Aries Systems Corporation

	Hengchao Wang
	Shuqu Li
	Fan Jiang
	Lijuan Yin
	Guojie Zhang
	Wanqiang Qian
	Wei Fan
**Order of Authors Secondary Information:**	
**Opposed Reviewers:**	
**Additional Information:**	
**Question**	**Response**
Are you submitting this manuscript to a special series or article collection?	No
**Experimental design and statistics**<br><br>Full details of the experimental design and statistical methods used should be given in the Methods section, as detailed in our Minimum Standards Reporting Checklist. Information essential to interpreting the data presented should be made available in the figure legends.<br><br>Have you included all the information requested in your manuscript?	Yes
**Resources**<br><br>A description of all resources used, including antibodies, cell lines, animals and software tools, with enough information to allow them to be uniquely identified, should be included in the Methods section. Authors are strongly encouraged to cite Research Resource Identifiers (RRIDs) for antibodies, model organisms and tools, where possible.<br><br>Have you included the information requested as detailed in our Minimum Standards Reporting Checklist?	Yes
**Availability of data and materials**<br><br>All datasets and code on which the conclusions of the paper rely must be either included in your submission or deposited in publicly available repositories	Yes

(where available and ethically appropriate), referencing such data using a unique identifier in the references and in the “Availability of Data and Materials” section of your manuscript.<br><br>Have you have met the above requirement as detailed in our Minimum Standards Reporting Checklist?	

Powered by Editorial Manager® and ProduXion Manager® from Aries Systems Corporation

Manuscript

Click here to download Manuscript PC_manuscript_submit_20180209.docx

The genome of golden apple snail *Pomacea canaliculata* provides insight into stress tolerance and invasive adaptation

Conghui Liu[1]*, Bo Liu[1]*, Yuwei Ren[1]*, Yan Zhang[1]*, Hengchao Wang[1], Shuqu Li[1], Fan Jiang[1], Lijuan Yin[1], Guojie Zhang[2], Wanqiang Qian[1†] and Wei Fan[1†]

[1]Agricultural Genomic Institute, Chinese Academy of Agricultural Sciences, Shenzhen, Guangdong, 518120, China.

[2]BGI-Shenzhen, Shenzhen, Guangdong, 518083, China

Conghui Liu: rapherlch@163.com; Bo Liu: lb_bobo@aliyun.com; Yuwei Ren: xiaoshudaxia@126.com; Yan Zhang: milrazhang@163.com; Hengchao Wang: wanghengchao000@qq.com; Shuqu Li: lishuqu1234@163.com; Fan Jiang: greatjf@163.com; Lijuan Yin: yinlijuan1005@163.com; Guojie Zhang: guojie.zhang@bio.ku.dk

*These authors contributed equally to this work.

[†]Correspondence should be addressed to Wanqiang Qian (qianwanqiang@caas.cn) or Wei Fan (fanwei@caas.cn).

Abstract

Background: The golden apple snail (*Pomacea canaliculata*) is a worldwide fresh water snail listed in the top-100 worst invasive species, and a noted agricultural and quarantine pest causing huge economic loss, characterized with fast growth, strong stress tolerance, high reproduction rate, and adaptation to a broad range of

environments.

Results: Here, we used long-read sequencing to produce a 440-Mb high-quality chromosome level assembly for *P. canaliculata* genome. In total, 50 Mb (11.4%) repeat sequences and 21,533 gene models were identified in the genome. Major findings of this study include the recent explosion of DNA/hAT-Charlie TEs, the expansion of P450 gene family and the constitution of cellular homeostasis system, contributing to the ecological plasticity in the stress adaptation. In addition, the perivitellin gene expansion and high transcriptional level in ovary promote the function of nutrients supplying and defense ability in the eggs. Furthermore, the gut metagenome also encodes rich genes for food digestion and xenobiotics degradation.

Conclusions: These findings collectively provide novel insight into the molecular mechanisms of the ecological plasticity and high invasiveness. Our results not only strengthen the understanding of molluscs genomics and biological invasion, but also benefit preventing the invasion of apple snail and transmission of pathogenetic parasites.

Keywords: golden apple snail, *Pomacea canaliculata*, genome, adaptive evolution, stress tolerance, P450, reproduction, perivitelline, metagenome

Background

The golden apple snail *Pomacea canaliculata* (family Ampullariidae; Order Architaenioglossa) is a fresh water snail listed in the 100 of the world's worst invasive species [1], and considered as a noted agricultural and quarantine pest worldwide [2].

Native to the tropical and subtropical South American, the *P. canaliculata* gradually spread to the non-indigenous region, such as Southeast and East Asia [3], Africa [4], North America [5], Oceania [6] and even Europe [7], and the successful biological invasion was due to polyphagous feeding habits [8], voracious appetite [9], broad environmental adaptability [10] and rapid growth and high rate of reproduction [11]. Besides the ecological impact, the *P. canaliculata* ravaged a wide range of crops including grain, fruit and vegetable [12], causing severe economic loss each year as a result of yield loss, replanting cost and the funds of control (https://www.cabi.org/isc/datasheet/68490). More seriously, *P. canaliculata* has involved in the transmission of a human fatal disease, Eosinophilic meningitis, that firstly appeared in East Asia where people take them as food frequently [13]. During this pathophoresis, *P. canaliculata* acts as an important intermediate host of pathogenic parasite *Angiostrongyulus cantonensis*, and the range of infectious regions is still expanding, causing great challenge to human health [14, 15].

Molluscs is a highly diverse group and second only to arthropods in species number [16], and the high biodiversity makes molluscs an excellent model to address the issues such as biogeography, adaptability and evolution process [17], and the worldwide invasive *P. canaliculata* provides valuable potential in these fields [18]. As a primitive circumtropical species, *P. canaliculata* possesses strong ecology plasticity to hold advantage on plenty of aspects, including low temperature resistance [19], drought tolerance [20], which contributes to succeed in resource acquisition over the competitive species. Additionally, *P. canaliculata* is tolerant with heavy metal

contamination. When living in contaminated water, its gill is enriched of high concentration of heavy metal and histopathological changes in digestive tract is detected, however, with extremely low mortality rate [21]. For protection of embryos, the conspicuous coloration and neurotoxic lectin could confer the eggs a survival advantage and defense against the potential predator [22]. Moreover, the immune-neuroendocrine system can also be detected in *P. canalicula*, demonstrates by the existence of a specific immune memory after the bacterial challenge [23, 24], broadening the studies of invertebrate immunology.

During the past years, the genomic features of *P. canalicula* have been increasingly studied. After the discovery of 14 pachytene bivalents in the karyotype [25], molecular markers were identified to investigate the genetic diversity of *P. canaliculata* population, including 369 amplified fragment length polymorphism (AFLP) locis [26], 16,717 simple sequence repeats (SSR) [27, 28] and 15,412 single-nucleotide polymorphisms SNPs [29]. In addition, multiple transcriptome analyses have been performed to investigate the adaptation, invasion and immune mechanisms. For instance, Sun et al. reported 128,436 unigenes based on a de novo assembly of Illumina reads [29], transcriptome changes in response to heat stress and starving incubation was used to characterize invasive and adaptive abilities [30, 31], a transcriptome analysis between invasive *P. canaliculata* and indigenous *Cipangopaludina cahayensis* provides insights into biological invasion [28], and 402 immune-related differentially expressed genes (DEGs) by Lipopolysaccharide (LPyS) challenge were used to explore the mechanisms against pathogens [32]. Furthermore,

proteomics tools such as Isobaric Tags For Relative, Absolute Quantitation (iTRAQ), and Liquid Chromatography-tandem Mass Spectrometry (LC-MS/MS) were also applied in the study of protein expression for the estivation and oviposition [33, 34], together providing plentiful omics-data for the functional analysis of *P. canalicula*. However, researches at whole genome level in *P. canaliculata* still lags far behind other molluscs species, due to the lack of a high-quality reference genome. By far, multiple draft genomes of molluscs have been published, such as Califonia sea hare [35], Pacific oyster [36], Pearl oyster [37], owl limpet [38], California two-spot octopus [39], deep-sea mussel [40], *Biomphalaria* snails [41], greatly promoting the research of molluscs genomics. In this study, we present a chromosome-level genome assembly of *P. canalicula* with high-quality gene annotation, transcriptome data from several tissues and under various conditions, as well as the metagenomic data from the intestinal tracts, all of which were then applied to study the species-specific invasive characters, such as cellular homeostasis system underlying strong stress, and color and nutrient of the eggs. Our data will not only strengthen the understanding of evolutionary mechanisms of molluscs and molecular basis of biological invasion, but also foster developments to control the invasion of *P. canalicula* and interrupt the transmission of pathogenetic nematode parasites.

RESULTS

Complete genome assembly at chromosome level

We generated 26.6 Gb (60.1 X) PacBio SMRT raw reads with average read length

10.1 Kb, and 291 Gb (652.4 X) Illumina HiSeq paired-end reads with read length 150-250 bp, using DNA extracted from one single adult *P. canaliculate* (Table S1). The 24.4 Gb (55.4 X) clean PacBio SMRT reads that passed quality filtering were assembled by smartdenovo (https://github.com/ruanjue/smartdenovo), giving rise to an assembly of 1234 raw contigs with total length 473.6 Mb and N50 length 1.0 Mb. After filtering of alternatively heterozygous contigs, 745 resulting contigs with total length 440.1 Mb and N50 length 1.1 Mb were taken as the final contigs. Previous karyotype research shown that haploid P. *canaliculate* genome consist of 14 chromosomes [25]. Based on Hi-C data, 439.5 Mb (99.9%) final contigs were anchored and oriented into 14 large scaffolds, each corresponding to a natural chromosome (Figure 1a and Figure 1b), with the longest 45.4 Mb and shortest 27.2 Mb. This assembly quality is much better than the other published mollucan genomes so far (Table 1). Besides the length and continuity of assembled sequences, another important aspect for evaluating genome assembly is the ratio of genome coverage. With an estimated genome size of 446 Mb based on distribution of k-mer frequency [42] (Figure S1), ~98.6 % of the genome has been assembled in *P. canaliculata*. To further confirm the accuracy and completeness of the assembly, we mapped the Illumina shotgun reads to the assembled reference genome. Significantly, 97% and 95% of the genome-derived and transcriptome-derived reads could be aligned to the reference genome, respectively, suggesting no obvious bias for sequencing and assembly. Additionally, the mitochondrial genome of *P. canaliculata* was also assembled as a single contig with 15,707 bp in length, which has 99.9 % sequence

identity to the published mitochondrial genome (GenBank: KJ739609.1) (Figure S2). The high-quality reference genome provides a good foundation for gene annotation. The protein-coding genes were predicted on the reference genome by EVM, integrating evidences from *de novo* prediction, transcriptome and homology data. In total, 21,533 gene models were predicted as the reference gene set, with coding regions spanning ~32.2 Mb (7.3 %) of the genome (Table 1 and Table S2). The distribution of CDS length in *P. canaliculata* is similar to the closely related species (Figure 1c). Overall, 97.5 % of the reference genes were supported by transcriptome data, and 98.0 % of eukaryote core genes from OrthoDB (http://www.orthodb.org/) were identified in the reference gene set by BUSCO, comparable to the other published mollucan genomes (Table 1). For the functional annotation, a total of 19,815 (91.9 %) reference genes were annotated by at least one functional database. Specifically, 15,662 (72.7 %), 13,769 (63.4 %), 17,081 (79.3 %), 18,847 (87.5 %) and 17,003 (79.9 %) reference genes were annotated with eggNOG, KEGG, NR, Interpro and Uniprot database, respectively (Figure S3).

Signs of Adaptive Evolution in P. canaliculata Genome

To gain insight into evolutionary perspective of *P. canaliculata*, the phylogenetic tree was built based on 471 high-confidence single-copy ortholog genes from seven related species (*P. canaliculata, L. gigantea, A. california, B. glabrata, C. gigas, O. bimaculoides and L. anatina*) by Phyml [43] and the divergence time was estimated using mcmctree [44]. The result shows that *P. canaliculata* diverged from the ancestor

of *B. glabrata* and *A. California* 290 million years ago (Mya), and from *L. gigantea* 415 Mya (Figure 2a).

Then, the molluscan ortholog genes were investigated for adaptive evolution. Utilizing pairwise protein sequence similarities, the gene family clustering was conducted by orthfinder [45]. A total of 152,878 reference genes from the seven species were clustered into 68,942 ortholog groups, amongst which 13,805 ortholog groups with at least two genes each. In *P. canaliculata*, we identified 9,626 ortholog groups, amongst which 117 and 5,462 ortholog groups undergone species-specific expansion, thus may play important roles in adaption to the environment as an invasive species. The functions of these orthologous groups are mainly related to glycan biosynthesis, digestive, endocrine, signal transduction, immune, or carbohydrate metabolism and so on (Figure S4).

The high-coverage genome assembly enables a comprehensive analysis of the transposable elements (TEs), which plays multiple roles in driving genome evolution in eukaryotes [46]. In total, we identified 49.6 Mb TE sequences in the assembled *P. canaliculata* genome (Table 1), including 3.4 Mb long terminal repeats (LTR), 27.2 Mb long interspersed elements (LINE), 17.5 Mb DNA transposons and 1.5 Mb short interspersed elements (SINE). Next, we analyzed the divergence rate of TEs for each class of TEs among the available sequenced mollusk genomes, interestingly, only the results of DNA transposons showed a unique peak at ~4% divergence rate for *P. canaliculata* and *C. gigas* (Figure 2b), indicating a recent explosion of DNA transposons in these two species. More than half of the DNA transposons belong to

the DNA/hAT-Charlie TE family, which is ~22.7% of total DNA/hAT-Charlie TEs in the genome. TEs are powerful facilitators of evolution by generating “evolutionary potential” to introduce small adaptive changes within a lineage, and the importance of TEs to stress responses and adaptation has been reported in numerous researches [47, 48]. The recent explosion of DNA/hAT-Charlie TEs in *P. canaliculata* could also play important roles to promote the potential plasticity in the stress adaptation.

Investigation of Cellular homeostasis system underlying strong stress adaptation

Homeostasis system plays a crucial role in the stress adaptability, providing the molecular basis in re-establishing the dynamic equilibrium after the challenge of various environmental stressors, including temperature, air exposure, anthropogenic pollution and pathogens [49]. In the present study, we addressed three constituent parts of the cellular homeostasis system, which contributes to the successful ecological plasticity of *P. canaliculata* (Figure 3). Transcriptome data of the hemocytes after stimulus (cold, heat, heavy and air exposure) was also sequenced and analyzed to address the potential roles of the genes in Cellular homeostasis system.

Unfolded protein response (UPR) system makes the central part of protein homeostasis [50]. Heat shock proteins (HSPs) acts as molecular chaperones to maintain the correct folding, and heat shock transcription factor 1 (HSF1) are responsible for the transcriptional induction of HSPs [51]. In *P. canaliculata* genome, 13 HSP70s，6 HSP90s, 7 HSP40s and 11 HSFs were identified (Table S3), and the expression of HSP90s and HSFs were highly induced in response to the stress of heat,

cold, heavy metal and air exposure (Table S4). Inositol-requiring protein 1 (IRE1), protein kinase RNA-like ER kinase (PERK), and activating transcription factor 6 (ATF6) are three mediators recruited by endoplasmic reticulum (ER) to regulated the UPR [52]. We found putative coding genes of the three core mediators, their respective downstream transcription factors, and the corresponding recognition chaperons in *P. canaliculata* genome (Table S3).

Xenobiotic biotransformation system helps the mollusc adapt to toxicants, especially the pesticide in aquatic environments [53]. Manual annotation on this genome identified 157 cytochrome P450s (CYP450s), 15 flavin-containing monooxygenases (FMOs), 53 glutathione S-transferases (GSTs) and 105 ATP binding cassette (ABC) transporters, most of which showed an up-regulation in expression under stress (Table S3, Table S4). These proteins are evidenced to function in contaminant detecting, conjugative modification and expulsion for xenobiotic detoxification [54-56].

Massive production of reactive oxygen species (ROS) and reactive oxygen intermediates (ROI) induced by stress lead to many pathological conditions, and antioxidant system protect the organism from superoxide [57]. Four main antioxidant enzyme classes, namely superoxide dismutase (SOD), catalase (CAT), peroxidase (Prx), and glutathione peroxidase (GPX), were found in the *P. canaliculata* with an elevating global expression in response to stress (Table S3, Table S4).

Apoptosis is a process of cell death when sensing stress and the regulation of apoptosis maintains the dynamic homeostasis of internal environment. In *P. canaliculata*, we propose the existence of both intrinsic and extrinsic apoptotic

signaling pathways, evidenced by the presence of homologous genes involve in both pathways. It seems these two pathways could be activated by cytochrome C and tumor necrosis factor receptor (TNFR), respectively (Table S3). The inhibitors of apoptosis, such as XIAP, Bcl2 and Bak, are also detected with an increased expression in response to the stress (Table S4), which are expected to delay the apoptosis process and the cell death in stress response.

The expansion of P450 gene family contribute to stress tolerance

Cytochromes P450 (CYP) enzymes are a monooxygenase family with highly diverse structures and functions, broadly identified in all kingdoms of life [58]. P450s catalyze the reductive scission of molecular oxygen, and are responsible for the synthesis and metabolism of various molecules, including drugs, hormones, antibiotics, pesticides, carcinogens and toxins [59]. The synthesized hormones, such as glucocorticoids, mineralocorticoids, progestins, and sex hormones, are critical to stress response, growth and reproduction, and the endogenous and exogenous chemical metabolism helps the host combat with the toxic compounds [60].

We found the *P. canaliculata* CYP gene family had greater level of expansion compared to the other molluscs. We identified 157 genes in the genome of *P. canaliculata*, and 128, 102, 135, 78, 52 and 94 genes from *A. California, B. glabrata, C. gigas, L. gigantean, O. bimaculoides* and *P. fucata* respectively under the same standard (Figure 4a). The expansive trend was also observed, compared with the model species, such as *Homo sapiens* (57), *Mus musculus* (102), *Dario rerio* (94) and

Drosophila melanogaster (94) [61]. The gene expansion was mainly found in CYP2U and CYP3A sub-families, and fewer genes expanded in CYP4F. In mammals, CYP2U plays a role in the metabolism of fatty acid to generate bioactive eicosanoid derivatives, potentially regulating the development of immune function [62]. In *P. canaliculata*, 40 genes forged into the CYP2U clade, mainly expressing in hepatopancreas (Figure 4b and Table S5_a, Table S5_b). CYP3A acts as a versatile enzyme metabolizing a wide range of xenobiotics, and the productions promote the growth of various cell types [63]. The 56 CYP3A genes have comprehensive expression in hepatopancreas, gill and kidney (Figure 4b and Table S5_a, Table S5_b). CYP4F possesses epoxygenase activity, metabolizing fatty acid to epoxides to suppress hypertension, pain perception and inflammation [64]. 20 genes were identified in CYP4F, and several CYP4F genes present highly induced expression levels under the stress of cold, heat, heavy metal and air exposure, indicating their critical roles in the stress tolerance (Figure 4b and Table S5_a, Table S5_b).

The perivitellin gene expansion and high transcriptional level in ovary enhance reproduction

To adapt to the fast invasion life, besides the strong ability to stress tolerance, the *P. canaliculata* possesses a high reproductive rate, and one important contributor is their distinct eggs characterized with abundant nutrients, reddish or pinkish color, aerial oviposition and neurotoxic [22, 34]. In most gastropod eggs, Pervitelline Fluid (PVF) with large amounts of nutrients filled in space between the eggshell and the embryo, is composed of carbohydrates, lipids and proteins termed perivitellins, which is not

only responsible for the major supply of material and energy during embryogenesis, but also provide warning pigment and deadly toxicant against the predators [65]. Perivitellins of *P. canaliculata* (Pc) have been verified by proteomics approach and was further divided into three categories called Pc Ovorubin (PcOvo), PcPV2, PcPV3, which are all high-density lipoprotein (HDL) [66] (Figure 5a). We totally identified 18 perivitellin genes from the *P. canaliculata* genome, compared to 2 and 1 perivitellin genes from *A. california* and *P. fucata* respectively, by aligning the seven reference perivitellin gene sequences (NCBI accession AFQ23940.1, AFQ23939.1, AFQ23938.1, AFQ23945.1, AFQ23937.1, P0C8G7.2, P0C8G6.2) to each genome sequences with the same method (blastn e-value 10^{-20}). It is apparent that the copy number of perivitellin genes was expanded in *P. canaliculata*, and our orthologous and paralogous gene family data by orthoFinder confirmed this. Among the 20 perivitellin genes in *P. canaliculate*, there are 2 PcOvo, 13 PcPV2, and 3 unclassified PVFs (Figure 5b and Table S6). The PcOvo carotenoprotein is responsible for the red coloration of the eggs and antioxidant to protect against sun radiation and desiccation [67, 68], while PcPV2 is reported to be neurotoxin implying lethal effect on rodents [22]. The expansion of these genes may enhance the underlying functions of nutrition and protection, offering the eggs an advantage of survival and improve the reproduction rate.

The expression of 18 *P. canaliculata* perivitelline genes were detected in 7 tissues, including embryo, testis, ovary, kidney, gill, hepatopancreas and hemocyte. The highest expression of each gene concentrated in embryo and two sexual gland testis

and ovary, especially in the ovary (Figure 5b and Table S7), suggesting that their decoding proteins might be of importance in germ cell production and embryo development. Taken together, *P. canaliculata* distinguish its embryo development from other seven species on the preponderance of perivitellin gene number and high expression level, that further promotes corresponding function of nutrients supplying and defense ability and eventually contribute to reproduction.

Gut microbiome plays important roles in stress resistance and food digestion

The gut microbiome is well known as the second genome of animals, which plays key roles in food digestion, immune defense, etc that are essential to the animals. To investigate whether the gut microbiome has influence on the invasive life style, we collected gut digesta samples from 70 adults of *P. canaliculata*, and generated 31 Gb high quality metagenomic data on Illumina HiseqX10 platform. To our knowledge, this is the first high-depth sequencing of snail gut microbiome. A total of 1,142,095 non-redundant genes were obtained, with an average open reading frame (ORF) length of 604 bp (Table S8). The taxonomic composition analysis showed that, at the phylum level, Proteobacteria was the predominant, followed by Verrucomicrobia, Bacteroidetes, Firmicutes, Spirochaetes, Actinobacteria, etc. (Table S9_a). At the genus level, the most abundant genera include *Aeromonas*, *Enterobacter*, *Desulfovibrio*, *Citrobacter*, *Comamonas*, *Klebsiella* and *Pseudomonas*. (Table S9_b), most of which were also presented in the snails of *Achatina fulica* [69, 70].

It is interesting that some of the most abundant genera such as *Desulfovibrio*,

Citrobacter and *Pseudomonas* were reported to have strong abilities of removing heavy metals, by mechanisms of bioprecipitation and bioabsorption [71-73]. For example, the sulfur-reducing bacteria *Desulfovibrio* produced H_2S that precipitate metals, and therefore reduced the toxic effects of dissolving metals [71]. Based on the KEGG pathway database, the complete sulfate reduction metabolism pathway was identified in the *P. canaliculata* gut microbiome. We suggested that the gut microbes might help *P. canaliculata* to confront with the environmental stress of heavy metals in hash conditions. In addition, a large number of genes in pathways of xenobiotics biodegradation and metabolism were annotated, corresponding to 288 KEGG orthologous groups (KOs) and 21 pathways (Table S10). As many of the pathways such as benzoate degradation, toluene degradation, xylene degradation and steroid degradation could not be identified in the host genome through KO analysis, we suggested that the microbial detoxification abilities may contribute the *P. canaliculata* to resist stresses caused by xenobiotics such as pesticides and environmental pollutants.

In view of dietary digestion, the gut microbes were directly involved in breakdown of the cellulose portion, and previous studies have isolated some cellulolytic bacteria and evaluated the cellulolytic enzyme activities [74]. In our work, a broader range of carbohydrate active enzymes (CAZymes) were found. Of the 208 annotated CAZyme families, 99 were Glycoside Hydrolase (GH) families (Table S11). Enzymes that could be classified as cellulases, endohemicelluloses, debranching enzymes, oligosaccharide-degrading enzymes were all presented. These findings indicate that

the gut microbiome give assistance to digest a broad range of food sources, making *P. canaliculata* grow fast to adapt to an invasive life style.

Conclusion and discussion

Given its environmental invasiveness, broad stress adaptability and rapid reproduction, the golden apple snail *P. canaliculata* has received a vast of attention worldwide. However, the underlying genetic mechanism has not been comprehensively uncovered. The chromosome level genome of *P. canaliculata* presented in this study sheds first lights into the genomic basis of the ecological plasticity to various stressors. Major findings of this study include the recent explosion of DNA/hAT-Charlie TEs, the expansion of P450 gene family and the constitution of Cellular homeostasis system, contributing to the plasticity in the stress adaptation. Although the defined function of the recently originated TEs could not be confirmed, the explosion of TEs is deemed as powerful facilitators in adaptive evolution, indicating its important role in *P. canaliculata*'s stress resistance. UPR system, Xenobiotic biotransformation system and ROS system are major components of the Cellular homeostasis system, and especially P450s expands with specific functions. In addition, exclusive perivitellin genes are characterized from the *P. canaliculata* genome, contributing to the high reproductive rate and the expansion of habitats. Furthermore, the gut metagenome encodes rich genes for food digestion and xenobiotics degradation. These findings collectively provide novel insight into the molecular mechanisms of the ecological plasticity and high invasiveness.

The rich phenotypic and genetic diversity of molluscs make them an excellent species group to address many valuable issues about evolution, ecology and function. However, the genomic resource of Mollusca is still insufficient compared with other close phylums, such as Arthropoda and Nematoda, and few molluscs could be employed as model organism. *P. canaliculata* possesses potential to be a model organism of molluscs because of several inherent characters. For example, *P. canaliculata* is easy to acquire, for it has a broad global distribution originated from a primarily circumtropical environment. Due to the high adaptability, rapid growth and efficient reproduction, *P. canaliculata* also facilitate the cultivation in laboratory. We report a fine reference genome of *P. canaliculata* in the present study, which is the first chromosome level genome published in Mollusca. As the cellular complexity and the conservation of pathways, *P. canaliculata* could be a representative of Mollusca, so the genome described in this study can be used to advance our understanding of the molecular mechanisms for various scientific issues in Mollusca.

Methods

Samples collection and sequencing

Adults of *P. canaliculata* were collected from a local paddy field in Shenzhen, Guangdong province, China, and maintained in aerated freshwater at 15 ± 2 °C for a week before processing. Genomic DNA was extracted from the foot muscles of a single *P. canaliculata* for constructing PCR free Illumina 350-bp insert libraries and

PacBio 20-kb insert library, and sequenced on Illumina HiSeq 2500 and PacBio SMRT platforms, respectively. The Hi-C library was prepared using the muscle tissue of another single *P. canaliculate* by following methods: Nuclear DNA was cross-linked in situ, extracted, and then digested with a restriction enzyme. The sticky ends of the digested fragments were biotinylated, diluted, and then ligated to each other randomly. Biotinylated DNA fragments were enriched and sheared again for preparing the sequencing library, which was then sequenced on a HiSeq X Ten platform (Illumina).

Seven tissues including embryos (2 days post fertilization), gill, hemocytes, hepatopancreas, kidney, ovary and testis from six animals were collected as parallel samples. Next, animals were cultivated in 37 °C and 10 °C for 24 hours heat and cold tolerance, in Cr^{3+}(2mg L^{-1}), Cu^{2+}(0.2mg L^{-1}) and Pb^{2+}(1mg L^{-1}) for 24 hours heavy metal tolerance, and in waterless tank for 7 days air exposure. Then the hemocytes were harvested and stored, with three replicates for each group. In final, total messenger RNAs (mRNA) were extracted from the stored tissues of *P. canaliculata* materials for constructing cDNA libraries (insert 350-bp), and sequenced on an Illumina HiSeq 2500 sequencer.

The intestinal digesta from 70 adult snails of *P. canaliculata* were collected, pooled into 6 samples and stored at −20 °C until microbial DNA was extracted. A combination of cell lysis treatments was applied, including five freeze-thaw cycles (alternating between 65 °C and liquid nitrogen for 5 min), repeated beads-beating in ASL buffer (cat. no. 19082; Qiagen Inc.), and incubated at 95 °C for 15 min. DNA

was isolated following the protocol reported protocol [75]. Paired-end libraries of metagenomic DNA were prepared with an insert size of 350 base pairs (bp) following the manufacture's protocol (cat. no. E7645L; New England Biolabs). Sequencing was performed on Illumina HiSeq X10.

Genome assembly and annotation

The Illumina raw reads were filtered by trimming the adapter sequence and low-quality part, resulting in a clean and high-quality reads data with average error rate < 0.001. For the PacBio raw data, the short subreads (< 2 kb) and low-quality (error rate > 0.2) subreads were filtered out, and only one representative subread was retained for each PacBio read. The clean PacBio reads were assembled by the software samrtdenovo (https://github.com/ruanjue/smartdenovo), then Illumina reads were aligned to the contigs by BWA-MEM, and single base errors in the contigs were corrected by Pilon (v1.16) with parameters "-fix bases, -nonpf, -minqual 20". The P. *canaliculata* genome is highly heterozygous illustrated by the double peaks on the distribution curve of K-mer frequency, and current assembly algorithm tends to collapse homozygous regions and report heterozygous regions in alternative contigs. To get a haploid reference contigs, we employed a whole-genome alignment (WGA) strategy by MUMmer v3.23 to recognize and selectively remove alternative heterozygous contigs, which were characterized by shorter length (less than 200 kb) and most regions (larger than 50%) can be aligned to another larger contig with

confident identity (higher than 80%). Next, Hi-C sequencing data were aligned to the haploid reference contigs by BWA-MEM, and then these contigs were clustered into chromosomes with LACH-ESIS (http://shendurelab.github.io/LACHESIS/).

The gene models in *P. canaliculata* genome were predicted by EVidence Modeler v1.1.1 [76], integrating evidences from ab initio predictions, homology-based searches and RNA-seq alignments. Then, the protein-coding sequences were mapped by RNA-seq data and functionally annotated using UniProt and InterProScan (5.16-55.0) databases [77]. Finally, the gene models were retained if they had at least one supporting evidence from UniProt database, InterProScan domain and RNA-seq data. Gene functional annotation was performed by aligning the protein sequences to NCBI NR, UniProt, COG and KEGG databases with BLASTP v2.3.0+ under E-value cutoff of 10^{-5} and choosing the best hit. The pathway analysis and functional classification were conducted based on KEGG database [78]. InterProScan was used to assign preliminary GO terms, Pfam domains and IPR domains to the gene models.

A de novo repeat library for *P. canaliculata* was constructed by RepeatModeler (v1.0.4; http://www.repeatmasker.org/RepeatModeler.html). TEs in the *P. canaliculata* genome were also identified by RepeatMasker (v4.0.6; http://www.repeatmasker.org/) using both Repbase library and the de novo library. Tandem repeats in the *P. canaliculata* genome were predicted using Tandem Repeats Finder v4.07b [79]. The divergence rates of TEs were calculated between the identified TE elements in the genome and their consensus sequence at the TE family level.

Evolutionary analysis

Orthologous and paralogous groups were assigned from seven species (*P. canaliculata, Lottia gigantea, Aplysia california, Biomphalaria glabrata, Crassostrea gigas, Octopus bimaculoides* and *Lingula anatina*) by OrthoFinder [45] with default parameters. Orthologous groups that contain only one gene for each species were selected to construct the phylogenetic tree. The protein sequences of each gene family was independently aligned by muscle v3.8.31 [80] and then concatenated into one super-sequence. The phylogenetic tree was constructed by maximum likelihood (ML) using PhyML v3.0 [43] with best-fit model (LG+I+G) that was estimated by ProtTest3 [81]. The Bayesian Relaxed Molecular Clock (BRMC) approach was adopted to estimate the neutral evolutionary rate and species divergence time using the program MCMCTree, implemented in PAML v4.9 package [44]. The calibration time (fossil record time) interval (173-398 Mya) of *Octopus bimaculoides* was adopted from previous results.

Transcriptome data analysis

Transcriptome reads were mapped to the reference genome of *P. canaliculata* using TopHat (v. 2.1.0) with default settings. The expression level of each reference gene in terms of FPKM was computed by cufflinks v2.2.1. A gene was considered to be expressed if its FPKM >0. Differential gene expression analysis was conducted using cuffdiff v2.2.1.

Metagenome data analysis

Raw reads were cleaned to exclude adapter sequences, low quality sequence, as well as contaminated DNA. The adapter sequence in reads were identified and trimmed by an ungapped dynamic programming algorithm; the low-quality part (head or tail) of reads were trimmed off to ensure that the average error rate of the left reads is lower than 0.001; the reads that mapped to the contaminated DNA by BWA-MEM [82] were filtered out; finally, shorter reads (length < 75-bp) and unpaired reads were excluded to form a clean reads data. The BWA database built for cleaning contamination included genomes of 10 species: *P. canaliculata* genome, *Brassica rapa* genome, *Oryza sativa* genome, 2 *Angiostrongylus cantonensis* genomes, *Caenorhabditis elegans* genome, *schistosoma mansoni* genome, *clonorchis sinensis* genome, *fasciola hepatica* genome, *Danio rerio* genome, and *human hg38* genome.

The clean reads were assembled by metaSPAdes (v3.11.1) [83] under pair-end mode for each sample, then gene prediction was performed on contigs longer than 500 bp by Prodigal (v2.6.3) [84] with parameter "-p meta", and gene models with cds length less than 102 bp were filtered out. A non-redundant (NR) gene set (539,344 genes) was constructed using the gene models predicted from each samples by cd-hit-est (v4.6.6) [85] with parameter "-c 0.95 -n 10 -G 0 –a S 0.9", which adopts a greedy incremental clustering algorithm and the criteria of identity > 95% and overlap > 90% of the shorter genes. Then, the clean reads were mapped onto this NR gene set by

BWA-MEM with the criteria of alignment length ≥ 50bp and identity > 95%. The unmapped reads from all samples were assembled together, and genes were predicted again. The newly predicted genes were combined with the previous gene set by cd-hit-est to get a new NR gene set (1,147,339 genes). After the taxonomic assignments to the new NR gene set, 5244 genes classified as Eukaryota but not fungi were removed, and the final NR gene set (1,142,095 genes) was obtained.

Taxonomic assignments for the final NR genes were made on the basis of DIAMOND [86] protein alignment against the NCBI-NR database by CARMA3 [87]. Functional annotation was performed by aligning all the protein sequences to the KEGG [88] database (release 79) using DIAMOND and taking the best hit with the criteria of E-value < 1e-5. CAZymes were annotated with dbCAN (release 5.0) [89] using HMMER (v3.0) hmmscan [90] by taking the best hit with E-value < 1e-18 and coverage > 0.35.

The clean reads from each sample were aligned against the gene catalog (1,142,095 genes) by BWA-MEM with the criteria of alignment length ≥ 50bp and identity > 95%. Sequence-based gene abundance profiling was performed as previously described [91]. Taxonomic profiles of the samples were calculated by adding the gene abundance together according to the taxonomic assignment result.

Abbreviations

P. Canaliculata, Pomacea canaliculata; L. gigantean, Lottia gigantean;

A. California, Aplysia California; B. glabrata, Biomphalaria glabrata; C. gigas, Crassostrea gigas; O. bimaculoides, Octopus bimaculoides; L. anatine, Lingula anatine; P. fucata, Pinctada fucata; Hem, hemocyte; Te, testis; Ov, ovary; Kn, kidney; GI, gill; Hp, hepatopancreas, Em, embryo; SSR, simple sequence repeats; mya, million years ago*; BLAST, basic local alignment search tool;* SNP, single nucleotide polymorphism; PVF, Pervitelline Fluid; Ovo, ovorubin; AFLP, amplified fragment length polymorphism; DEGs, differentially expressed genes; LPyS, Lipopolysaccharide; iTRAQ, Isobaric Tags For Relative, Absolute Quantitation; LC-MS/MS, Liquid Chromatography-tandem Mass Spectrometry; TEs, transposable elements; LTR, long terminal repeats; LINE, long interspersed elements; SINE, short interspersed elements; UPR, Unfolded protein response; HSPs, heat shock proteins; HSF1, heat shock transcription factor 1; PERK, protein kinase RNA-like ER kinase; ATF6,activating transcription factor 6; ER, endoplasmic reticulum; CYP450s, cytochrome P450s; FMOs, flavin-containing monooxygenases; GSTs, glutathione S-transferases; ABC, ATP binding cassette; ROS, reactive oxygen species; ROI, reactive oxygen intermediates; SOD, superoxide dismutase; CAT, catalase; Prx, peroxidase; GPX, glutathione peroxidase; TNFR, tumor necrosis factor receptor; NR, non-redundant genes; ORF, open reading frame; Kos, orthologous groups; CAZymes, carbohydrate active enzymes; GH, Glycoside Hydrolase.

Availability of data and materials

Tables S1 to S11 and Figures S1 to S4 are available in the supplementary information file. The raw sequencing data has been deposited in DDBJ/EMBL/GenBank under project accession PRJNA427478, SRR6425828 for genomic Illumina_PE125 sequencing data, SRR6425829 for genomic Illumina_PE150 sequencing data, SRR6425827 for genomic Pacbio sequencing data, SRR6429132~SRR6429164 for transcriptome sequencing data, and SRR6472920~SRR6472925 for gut microbiome data. All the analysis data have also been released for public use and can be freely accessed at AGIS ftpsite: ftp://ftp.agis.org.cn/~fanwei/Pomacea_canaliculata_Genome/ .

Authors' contributions

WF and WQ conceived the study and designed the experiments. CL and YZ performed the genome sequencing and assembly, BL performed annotation and evolutionary analysis. CL performed the stress tolerance analysis, YR performed the reproduction analysis, YZ performed the metagenome analysis. HW, SL, FJ, LY provide suggestions and help checking. WF, CL, BL, YR, YZ wrote the manuscript, and GZ help revise the manuscript. All authors read and approved the final manuscript.

Competing interests

The authors declare that they have no competing interests.

Acknowledgements

This project is supported by the National key research and development program of China (2016YFC1200600), Shenzhen science and technology program (JCYJ20150630165133395), Fund of Key Laboratory of Shenzhen (ZDSYS20141118170111640), and The Agricultural Science and Technology Innovation Program (ASTIP) of Chinese Academy of Agricultural Sciences(CAAS) & Elite Youth Program of Chinese Academy of Agricultural Sciences. We thank Fanghao Wan, Jue Ruan, Yutao Xiao for providing constructive suggestions to this project.

Legends of Tables and Figures

Tables

Table 1. Summary of assembly and annotation of mollusk genomes

Genome feature	*P. canaliculata*	*L. gigantea*	*A. californica*	*B. glabrata*	*C. gigas*	*O. bimaculoides*
Assembled sequences (bp)	440,071,717	359,505,668	927,310,431	916,377,450	557,735,934	2,3381,887,882
Contig N50 size (bp)	1,072,857	94,165	9,817	18,978	37,218	5,982
Contig N90 size (bp)	303,904	10,180	1,626	5,132	11,109	1,606
Scaffold N50 size (bp)	31,531,291	1,870,055	917,541	48,059	401,685	475,182
Scaffold N90 size (bp)	23,662,357	74,480	207,390	817	68,181	79,088
GC content (%)	40.3	33.3	40.3	36.0	33.4	36
No. of gene models	21,533	23,824	19,909	14,224	28,402	15,814
Avg. CDS length (bp)	1,497	1,136	1,568	1,066	1,472	1,535
BUSCO (%)	98.9	98.4	98.7	72.8	99.4	98.7
Transposable elements (bp)	49,579,006	37,369,817	202,174,499	189,550,886	103,381,274	737,398,096
Tandem repeat (bp)	873,801	257,674	8,263,822	2,145,821	590,907	62,633,792

Figures

Figure 1. The genome characteristics of *P. canaliculata*. (a) Circos plot showing the genomic features**.** Track 1: 14 linkage groups of the genome; Track 2: distribution of transposon elements in chromosomes; Track 3: protein-coding genes located on chromosomes; Track 4: distribution of GC contents. **(b)** A genome-wide contacting matrix from Hi-C data between each pair of the 14 chromosomes, using 100 kb window size. The color value means the logarithm of valid reads to base 2 (log_2(valid reads)). **(c)** Distribution of CDS length in six closely related species.

Figure 2. Evolutionary genomic analysis between *P. canaliculata* and other molluscs. (a) Phylogenetic placement of *P. canaliculata* within the molluscs dated tree. The estimated divergence time were shown on each branching point, the species marked with red color was *P. canaliculata*. **(b)** Distribution of divergence rate for the class of DNA transposons in molluscs genomes. The divergence rate was calculated by comparing all TE sequences identified in the genome to its corresponding

consensus sequence in each TE subfamily. The red arrow indicates the P. canaliculata and C. gigas had a recent explosion of TEs at ~4% divergence rate.

Figure 3. The cellular homeostasis system in *P. canaliculata*. Unfolded protein response (UPR) system included HSPs and HSF in the heat shock response and CNX, NEF, GRP94, BIP, HSP40, ATF6, IRE1, PERK, COP2, XBP, ATF4, TRAM and Derlin in the endoplasmic reticulum unfolded-protein response (UPR-ERAD). Apoptotic pathways included XIAPs, Bcl2, caspases, TNFR, and FADD. The antioxidant systems included PRX, SOD, CAT and GPX. The xenobiotic biotransformation system included EPHX3, P450, FMO and ABC transpoter. Gene boxes for gene families with the filled colors represent the degree of upregulation (FPKM-stimulus/FPKM-control) by an ovreall result of stress including heat, cold, heavy metal and air exposure. Pathways and genes were obtained based on KEGG annotation.

Figure 4. The expansion of P450 gene family in *P. canaliculata*. (a) Phylogenetic tree demonstrating orthologous and paralogous relationships of all P450 genes from 7 species including *P. canaliculata*, *A. california*, *B. glabrata*, *C. gigas*, *L. gigantea*, *O.bimaculoides* and *P. fucata*. P450 genes from seven species were obtained based Pfam annotation (Interpro) with the E-value 10^{-5}. Clades are labeled by P450 subfamily names. The tree was constructed using the Maximum likelihood method in MEGA7, and branch length scale indicates average residue substitutions per site. (b) Phylogenetic tree of P450 genes in *P. canaliculata*, which is a subset of the

phylogenetic tree for the 7 species, and their heat map of expression (FPKM) in seven tissues (Hem, hemocyte; Te, testis; Ov, Ovary; Kn, kidney; Gl, gill; Hp, hepatopancreas; Em, Embryo), and heat map of induced expression (FPKM-stimulus/FPKM-control) under stress (Con: control; heat; cold; Hm: heavy metal; Exp: air exposure).

Figure 5. The *P. canaliculata* perivitellins composition and expression in different tissues. (a) Pervitelline Fluid (PVF) is under the eggshell and surrounds the embryo, it contains carbohydrates, lipids, proteins, and the proteins is also known as perivitellins and classified into three categories of PcOvo, PcPV2, PcPV3. (b) The shown expression value is the logarithm of FPKM to base 2 (log_2FPKM). The first 3 letters in each gene ID refer to three classes of perivitellins, uPV means unclassified perivitellins, PV2 means PcPV2, Ovo means PcOvo. Abbreviations were used for 7 tissues (Hem, hemocyte; Te, testis; Ov, Ovary; Kn, kidney; Gl, gill; Hp, hepatopancreas; Em, Embryo).

References

1. Lowe S, Browne M, Boudjelas S, de Poorter M. 100 of the World's Worst Invasive Alien Species: A selection fromthe Global Invasive Species Database. Auckland, New Zeland: World Conservation Union (IUCN); 2000.

2. Ranamukhaarachchi SL, Wickramasinghe S. Golden apple snails in the world:

introduction, impact, and control measures. Global advances in ecology and management of golden apple snails. 2006:133-52.

3. Naylor R. Invasions in Agriculture: Assessing the Cost of the Golden Apple Snail in Asia. Royal Swedish Academy of Sciences. 1996;25:443-8.

4. Berthold T. Vergleichende Anatomie, Phylogenie und historische Biogeographie der Ampullariidae: (Mollusca, Gastropoda). 1991.

5. Howells RG, Burlakova LE, Karatayev AY, Marfurt RK, Burks RL. Native and introduced Ampullaridae in North America: History, status, and ecology. 2006:73-112.

6. Halwart M, Bartley DM. International mechanisms for the control and responsible use of alien species in aquatic ecosystems, with special reference to the golden apple snail. Los Baños, Philippines: Philippine Rice Research Institute (PhilRice); 2006.

7. López MA, Altaba CR, Andree KB, López V. First invasion of the Apple snail Pomacea insularum in Europe. Tentacle. 2010;18:26-8.

8. Estebenet AL, Martín PR. Pomacea canaliculata (Gastropoda: Ampullariidae): life-history traits and their plasticity. Biocell 2002;26:83-9.

9. Lach L. The spread of the introduced freshwater apple snail *Pomacea canaliculata* (Lamarck) (Gastropoda Ampullariidae) on Oahu, Hawaii. Bishop Museum Occasional Papers. 1999;58:66-71.

10. Yusa Y，Sugiura N, Wada T. Predatory Potential of Freshwater Animals on an Invasive Agricultural Pest, the Apple Snail *Pomacea canaliculata* (Gastropoda: Ampullariidae), in Southern Japan. Biol Invasions. 2006;8:137-47.

11. Lach L, Britton DK, Rundell RJ, Cowie RH. Food Preference and Reproductive Plasticity

in an Invasive Freshwater Snail. Biol Invasions. 2000;2:279-88.

12. Mochida O. Spread of freshwater *Pomacea* snails (Pilidae, Mollusca) from Argentina to Asia. Micronesica. 1991;3 51-62.

13. Shan L, Zhang Y, Steinmann P, Zhou X. Emerging Angiostrongyliasis in Mainland China. Emerg Infect Dis. 2008;14:161-4.

14. Caldeira RL, Mendonca CL, Goveia CO, Lenzi HL, Graeff-TeixeiraC Lima WS, et al. First record of molluscs naturally infected with *Angiostrongylus cantonensis* (Chen, 1935) (Nematoda: Metastrongylidae) in Brazil. Memórias do Instituto Oswaldo Cruz. 2007;102:887-9.

15. McMichael AJ, Beaglehole R. The changing global context of public health. Lancet (London, England). 2000;356:495-9.

16. Chapman A. Numbers of Living Species in Australia and the World. Australian Biological Resources Study; 2009.

17. Lindberg DR, Ponder WF, Haszprunar G. The Mollusca: relationships and patterns from their first half-billion years. Oxford University Press, Oxford; 2004.

18. Hayes KA, Cowie RH, Thiengo SC. A global phylogeny of apple snails: Gondwanan origin, generic relationships, and the influence of outgroup choice (Caenogastropoda: Ampullariidae). Biol J Linn Soc Lond. 2009;98:61-76.

19. Matsukura K, Tsumuki H,Izumi Y, Wada T. Physiological response to low temperature in the freshwater apple snail, *Pomacea canaliculata* (Gastropoda: Ampullariidae). J Exp Biol. 2009;212:2558-63.

20. Yusa Y, Wada T, Takahashi S. Effects of dormant duration, body size, self-burial and

water condition on the long-term survival of the apple snail, *Pomacea canaliculata* (Gastropoda: Ampullariidae). Appl Entomol Zool. 2006;41:627-32.

21. Kruatrachue M, Sumritdee C, Pokethitiyook P, Singhakaew S. Histopathological effects of contaminated sediments on golden apple snail (*Pomacea canaliculata,* Lamarck 1822). Bull Environ Contam Toxicol. 2011;86:610-4.

22. Dreon MS, Frassa MV, Ceolín M, Ituarte S, Qiu JW, Sun J, et al. Novel animal defenses against predation: a snail egg neurotoxin combining lectin and pore-forming chains that resembles plant defense and bacteria attack toxins. PLoS One. 2013;8:e63782. doi:10.1371/journal.pone.0063782.

23. Ottaviani E, Caselgrandi E, Fontanili P, Franceschi C. Evolution, immune responses and stress: studies on molluscan cells. Acta Biol Hung. 1992;43:293-8.

24. Ottaviani E, Accorsi A, Rigillo G, Malagoli D, Blom JM, Tascedda F. Epigenetic modification in neurons of the mollusc *Pomacea canaliculata* after immune challenge. Brain Res. 2013;1537:18-26.

25. Mercado Laczkó AC, Lopretto EC. Estudio cromosómico y cariotípico de p*omacea canaliculata* (Lamarck, 1801) (Gastropoda, Ampullariidae). Revista del Museo Argentino de Ciencias Naturales "Bernardino Rivadavia" Hidrobiología. 1998;8:15-20.

26. Xu J, Han X, Li N, Yu J, Qian C, Bao Z. Analysis of genetic diversity of three geographic populations of *Pomacea canaliculata* by AFLP. Acta Ecol Sin. 2009;29:4119- 26.

27. Chen L, Xu H, Li H, Wu J, Ding H, Liu Y. Isolation and characterization of sixteen polymorphic microsatellite loci in the golden apple snail *Pomacea canaliculata*. Int J Mol Sci. 2011;12:5993-8.

28. Mu X, Hou G, Song H, Xu P, Luo D, Gu D, et al. Transcriptome analysis between invasive *Pomacea canaliculata* and indigenous *Cipangopaludina cahayensis* reveals genomic divergence and diagnostic microsatellite/SSR markers. BMC Genet. 2015;16:12.

29. Sun J, Wang M, Wang H, Zhang H, Zhang X, Thiyagarajan V, et al. De novo assembly of the transcriptome of an invasive snail and its multiple ecological applications. Mol Ecol Resour. 2012;12:1133-44.

30. Mu H, Sun J , Fang L, Luan T, Williams GA, Cheung SG, et al. Genetic Basis of Differential Heat Resistance between Two Species of Congeneric Freshwater Snails: Insights from Quantitative Proteomics and Base Substitution Rate Analysis. J Proteome Res. 2015;14:4296-308.

31. Yang L, Cheng TY, Zhao FY. Comparative profiling of hepatopancreas transcriptomes in satiated and starving *Pomacea canaliculata*. BMC Genet. 2017;18:18.

32. Xiong YM, Yan ZH, Zhang JE, Li HY. Analysis of albumen gland proteins suggests survival strategies of developing embryos of *Pomacea canaliculata*. Molluscan Res. 2017:1-6.

33. Sun J, Mu H , Zhang H, Chandramouli KH, Qian PY, Wong CK, et al. Understanding the regulation of estivation in a freshwater snail through iTRAQ-based comparative proteomics. J Proteome res. 2013;12:5271-80.

34. Sun J, Zhang H, Wang H, Heras H, Dreon MS, Ituarte S, et al. First proteome of the egg perivitelline fluid of a freshwater gastropod with aerial oviposition. J Proteome Res. 2012;11:4240-8.

35. Aplysia Genome Project. Broad Institute. Vertebrate Biology Group. 2009.

https://www.broadinstitute.org/aplysia/aplysia-genome-project

36. Zhang G, Fang X, Guo X, Li L, Luo R, Xu F, et al. The oyster genome reveals stress adaptation and complexity of shell formation. Nature. 2012;490:49-54.

37. Takeuchi T, Kawashima T, Koyanagi R, Gyoja F, Tanaka M, Ikuta T, et al. Draft genome of the pearl oyster *Pinctada fucata*: a platform for understanding bivalve biology. DNA Res. 2012;19:117-30.

38. Simakov O, Marletaz F, Cho SJ, Edsinger-Gonzales E, Havlak P, Hellsten U, et al. Insights into bilaterian evolution from three spiralian genomes. Nature. 2013;493:526-31.

39. Albertin CB, Simakov O, Mitros T, Wang ZY, Pungor JR, Edsinger-Gonzales E, et al. The octopus genome and the evolution of cephalopod neural and morphological novelties. Nature. 2015;524:220-4.

40. Sun J, Zhang Y, Xu T, Zhang Y, Mu H, Zhang Y, et al. Adaptation to deep-sea chemosynthetic environments as revealed by mussel genomes. Nat Ecol Evol. 2017;1:121. doi:10.1038/s41559-017-0121.

41. Adema CM, Hillier LW, Jones CS, Loker ES, Knight M, Minx P, et al. Corrigendum: Whole genome analysis of a schistosomiasis-transmitting freshwater snail. Nat Commun. 2017;8:16153.

42. Liu B, Shi Y, Yuan J, Hu X, Zhang H, Li N, et al. Estimation of genomic characteristics by analyzing k-mer frequency in de novo genome projects. Quantitative Biology 2013:arXiv:1308.2012 [q-bio.GN].

43. Guindon S, Dufayard JF, Lefort V, Anisimova M, Hordijk W, Gascuel O. New algorithms and methods to estimate maximum-likelihood phylogenies: assessing the performance of

PhyML 3.0. Syst Biol 2010;59:307-21. doi:10.1093/sysbio/syq010.

44. Yang Z. PAML 4: phylogenetic analysis by maximum likelihood. Mol Biol Evol. 2007;24:1586-91. doi:10.1093/molbev/msm088.

45. Emms DM, Kelly S. OrthoFinder: solving fundamental biases in whole genome comparisons dramatically improves orthogroup inference accuracy. Genome Biol. 2015;16:157. doi:10.1186/s13059-015-0721-2.

46. Feschotte C, Wessler SR. Mariner-like transposases are widespread and diverse in flowering plants. Proc Natl Acad Sci U S A 2002;99:280-5.

47. Hua-Van A, Le Rouzic A, Boutin TS, Filée J, Capy P. The struggle for life of the genome's selfish architects. Biol Direct. 2011;6:19.

48. Werren JH. Selfish genetic elements, genetic conflict, and evolutionary innovation. Proc Natl Acad Sci U S A. 2011;108 Suppl 2:10863-70.

49. Chrousos GP. Stress and disorders of the stress system. Nat Rev Endocrinol. 2009;5:374-81.

50. Vabulas RM, Raychaudhuri S, Hayer-Hartl M. Protein folding in the cytoplasm and the heat shock response. Cold Spring Harbor perspectives in biology. 2010;2:a004390.

51. Chen B，Retzlaff M，Roos T，Frydman J. Cellular Strategies of Protein Quality Control. Cold Spring Harbor Perspectives in Biology. 2011;3:a004374.

52. Korennykh A and Walter P. Structural basis of the unfolded protein response. Annu Rev Cell Dev Biol. 2012;28:251-77.

53. Chambers JE and Yarbrough JD. Xenobiotic biotransformation systems in fishes. Comp Biochem Physiol C. 1976;55:77-84.

54. Mello DF, de Oliveira ES, Vieira RC, Simoes E, Trevisan R, Dafre AL, et al. Cellular and Transcriptional Responses of *Crassostrea gigas* Hemocytes Exposed in Vitro to Brevetoxin (PbTx-2) Mar Drugs. 2012;10: 583-97.

55. Boutet I, Tanguy A, Moraga D. Characterisation and expression of four mRNA sequences encoding glutathione S-transferases pi, mu, omega and sigma classes in the Pacific oyster *Crassostrea gigas* exposed to hydrocarbons and pesticides. Mar Biol 2004;146:53-64.

56. Deeley RG, Westlake C, Cole SP. Transmembrane transport of endo- and xenobiotics by mammalian ATP-binding cassette multidrug resistance proteins. Physiol Rev. 2006;86:849-99.

57. Liu C, Zhang T, Wang L, Wang M, Wang W, Jia Z, et al. The modulation of extracellular superoxide dismutase in the specifically enhanced cellular immune response against secondary challenge of Vibrio splendidus in Pacific oyster (*Crassostrea gigas*). Dev Comp Immunol. 2016;63:163-70.

58. Lamb DC, Lei L, Warrilow AG, Lepesheva GI, Mullins JG, Waterman MR, et al. The first virally encoded cytochrome p450. J Virol. 2009;83:8266-9.

59. Urlacher VB, Girhard M. Cytochrome P450 monooxygenases: an update on perspectives for synthetic application. Trends Biotechnol. 2012;30:26-36.

60. Sanderson T, van den Berg M. Topic 3.1: Interactions of xenobiotics with the steroid hormone biosynthesis pathway. Pure Appl Chem. 2003;75:1957-71.

61. Goldstone JV, McArthur AG, Kubota A, Zanette J, Parente T, Jönsson ME, et al. Identification and developmental expression of the full complement of Cytochrome P450 genes in Zebrafish. BMC Genomics. 2010;11:643.

62. Chuang SS, Helvig C, Taimi M, Ramshaw HA, Collop AH, Amad M, et al. CYP2U1, a novel human thymus- and brain-specific cytochrome P450, catalyzes omega- and (omega-1)-hydroxylation of fatty acids. J Biol Chem. 2004;279:6305-14.

63. Fleming I. The pharmacology of the cytochrome P450 epoxygenase/soluble epoxide hydrolase axis in the vasculature and cardiovascular disease. Pharmacol Rev. 2014;66:1106-40.

64. Zhang G, Kodani S, Hammock BD. Stabilized epoxygenated fatty acids regulate inflammation, pain, angiogenesis and cancer. Prog Lipid Res. 2014;53:108-23.

65. de Jong-Brink M, Boer HH, Joosse J. Mollusca. In: Adiyodi, K.G., Adiyodi, R.G. (Eds.), Reproductive Biology of invertebrates. Oogenesis oviposition and oosorption, vol. 1. John Wiley & Sons Ltd., New York, 1983; pp. 297-355.

66. Garin CF, Heras H, Pollero RJ. Lipoproteins of the egg perivitelline fluid of *Pomacea canaliculata* snails (Mollusca: Gastropoda). J Exp Zool. 1996;276:307-14.

67. Dreon MS, Schinella G, Heras H, Pollero RJ. Antioxidant defense system in the apple snail eggs, the role of ovorubin. Arch Biochem Biophys. 2004;422:1-8.

68. Dreon MS, Ituarte S, Heras H. The role of the proteinase inhibitor ovorubin in apple snail eggs resembles plant embryo defense against predation. PLoS One. 2010;5:e15059. doi:10.1371/journal.pone.0015059.

69. Cardoso AM, Cavalcante JJV, Vieira RP, Lima JL, Grieco MAB, Clementino MM, et al. Gut Bacterial Communities in the Giant Land Snail *Achatina fulica* and Their Modification by Sugarcane-Based Diet. Plos One. 2012;7 doi:ARTN e3344010.1371/journal.pone.0033440.

70. Cardoso AM, Cavalcante JJV, Cantão ME, Thompson CE, Flatschart RB, Glogauer A, et al. Metagenomic Analysis of the Microbiota from the Crop of an Invasive Snail Reveals a Rich Reservoir of Novel Genes. Plos One. 2012;7 doi:ARTN e4850510.1371/journal.pone.0048505.

71. Cabrera G, Pérez R, Gómez JM, Ábalos A, Cantero D. Toxic effects of dissolved heavy metals on Desulfovibrio vulgaris and Desulfovibrio sp strains. J Hazard Mater 2006;135:40-6. doi:10.1016/j.jhazmat.2005.11.058.

72. Finlay JA, Allan VJ, Conner A, Callow ME, Basnakova G, Macaskie LE. Phosphate release and heavy metal accumulation by biofilm-immobilized and chemically-coupled cells of a Citrobacter sp. pre-grown in continuous culture. Biotechnol Bioeng. 1999;63:87-97.

73. Valls M, de Lorenzo V, Gonzalez-Duarte R, Atrian S. Engineering outer-membrane proteins in *Pseudomonas putida* for enhanced heavy-metal bioadsorption. J Inorg Biochem. 2000;79:219-23.

74. Pinheiro GL, Correa RF, Cunha RS, Cardoso AM, Chaia C, Clementino MM, et al. Isolation of aerobic cultivable cellulolytic bacteria from different regions of the gastrointestinal tract of giant land snail *Achatina fulica*. Front Microbiol. 2015;6 doi:Artn 86010.3389/Fmicb.2015.00860.

75. Zoetendal EG, Heilig HG, Klaassens ES, Booijink CC, Kleerebezem M, Smidt H, et al. Isolation of DNA from bacterial samples of the human gastrointestinal tract. Nature protocols 2006, 1(2): 870-873

76. Haas BJ, Salzberg SL, Zhu W, Pertea M, Allen JE, Orvis J, et al. Automated eukaryotic

gene structure annotation using EVidenceModeler and the Program to Assemble Spliced Alignments. Genome Biol. 2008;9:R7.

77. Quevillon E, Silventoinen V, Pillai S, Harte N, Mulder N, Apweiler R, et al. InterProScan: protein domains identifier. Nucleic Acids Res. 2005;33:W116-20.

78. Kanehisa M, Goto S, Sato Y, Furumichi M, Tanabe M. KEGG for integration and interpretation of large-scale molecular data sets. Nucleic Acids Res. 2012;40:D109-D14.

79. Benson G. Tandem repeats finder: a program to analyze DNA sequences. Nucleic Acids Res. 1999;27:573-80.

80. Edgar RC. MUSCLE: multiple sequence alignment with high accuracy and high throughput. Nucleic Acids Res. 2004;32:1792-7.

81. Darriba D, Taboada GL, Doallo R, Posada D. ProtTest 3: fast selection of best-fit models of protein evolution. Bioinformatics. 2011;27:1164-5. doi:10.1093/bioinformatics/btr088.

82. Li H and Durbin R. Fast and accurate short read alignment with Burrows-Wheeler transform. Bioinformatics. 2009;25:1754-60.

83. Nurk S, Meleshko D, Korobeynikov A, Pevzner PA. metaSPAdes: a new versatile metagenomic assembler. Genome Res. 2017;27:824-34.

84. Hyatt D, LoCascio PF, Hauser LJ, Uberbacher EC. Gene and translation initiation site prediction in metagenomic sequences. Bioinformatics. 2012;28:2223-30.

85. Fu L, Niu B, Zhu Z, Wu S, Li W. CD-HIT: accelerated for clustering the next-generation sequencing data. Bioinformatics. 2012;28:3150-2.

86. Buchfink B, Chao X, Huson DH. Fast and sensitive protein alignment using DIAMOND. Nat Methods. 2015;12:59-60.

87. Gerlach W and Stoye J. Taxonomic classification of metagenomic shotgun sequences with CARMA3. Nucleic Acids Res. 2011;39 doi:Artn E9110.1093/Nar/Gkr225.

88. Kanehisa M, Goto S, Kawashima S, Okuno Y, Hattori M. The KEGG resource for deciphering the genome. Nucleic Acids Res. 2004;32:D277-80.

89. Yin Y, Mao X, Yang J, Chen X, Mao F, Xu Y. dbCAN: a web resource for automated carbohydrate-active enzyme annotation. Nucleic Acids Res. 2012;40:W445-51.

90. Eddy SR. Accelerated Profile HMM Searches. Plos Comput Biol. 2011;7 doi:ARTN e100219510.1371/journal.pcbi.1002195.

91. Qin JJ, Li YR, Cai ZM, Li SH, Zhu JF, Zhang F, et al. A metagenome-wide association study of gut microbiota in type 2 diabetes. Nature. 2012;490:55-60.

Figure

Click here to download Figure Fig.1.pdf

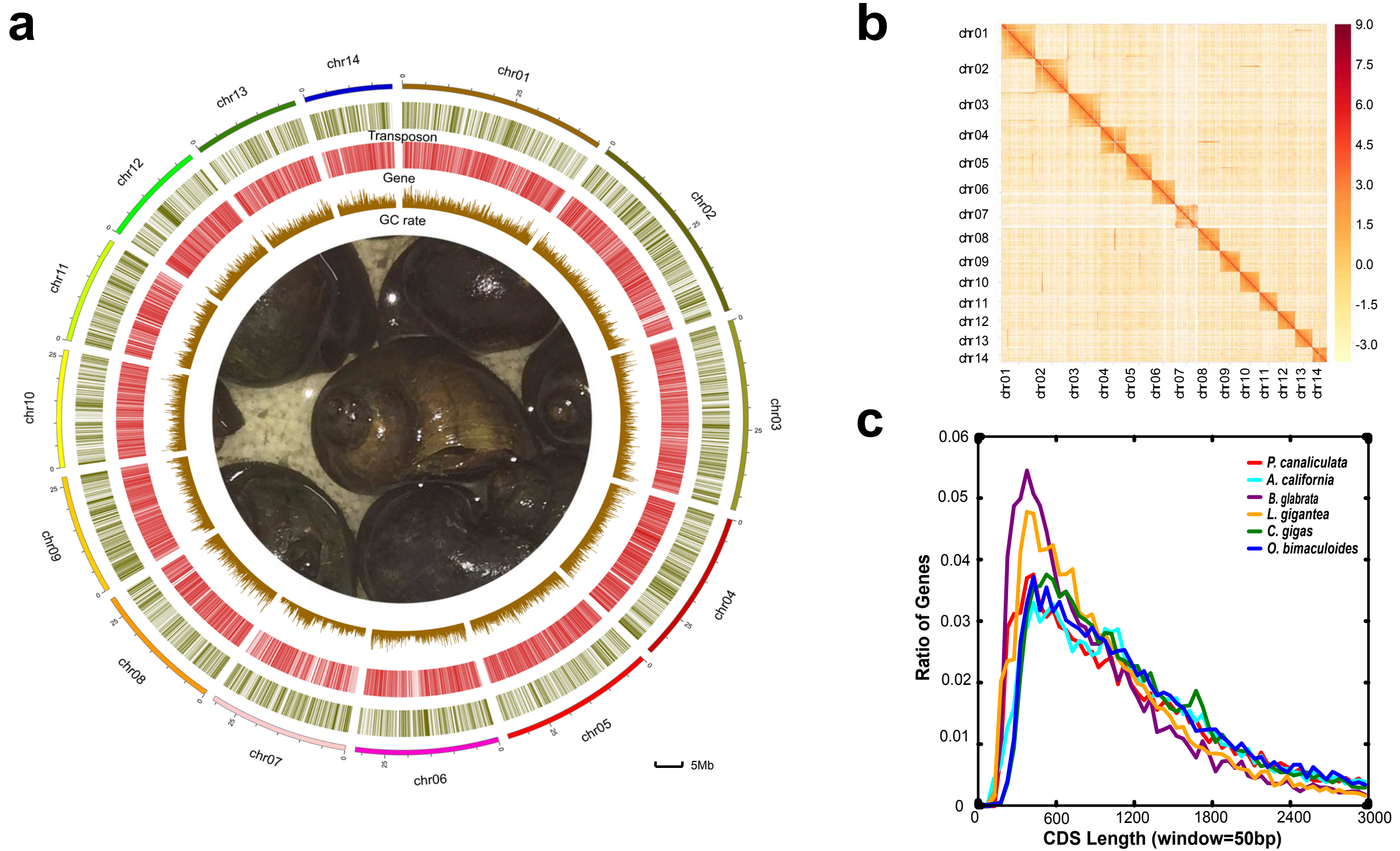

Figure

Click here to download Figure Fig.2.pdf

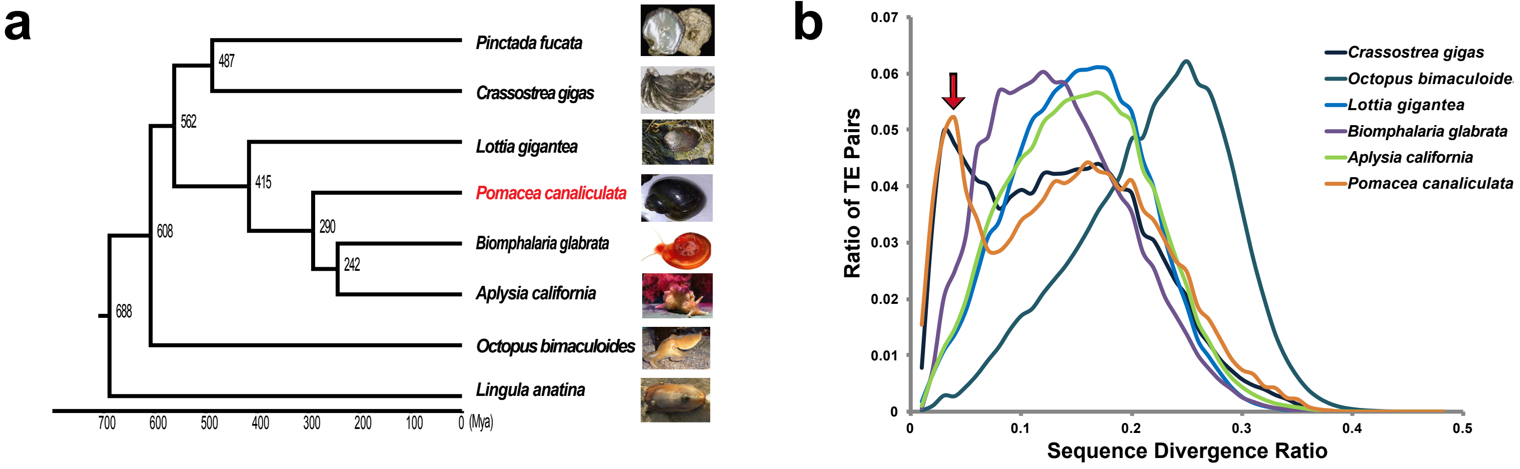

Figure

Click here to download Figure Fig.3.pdf

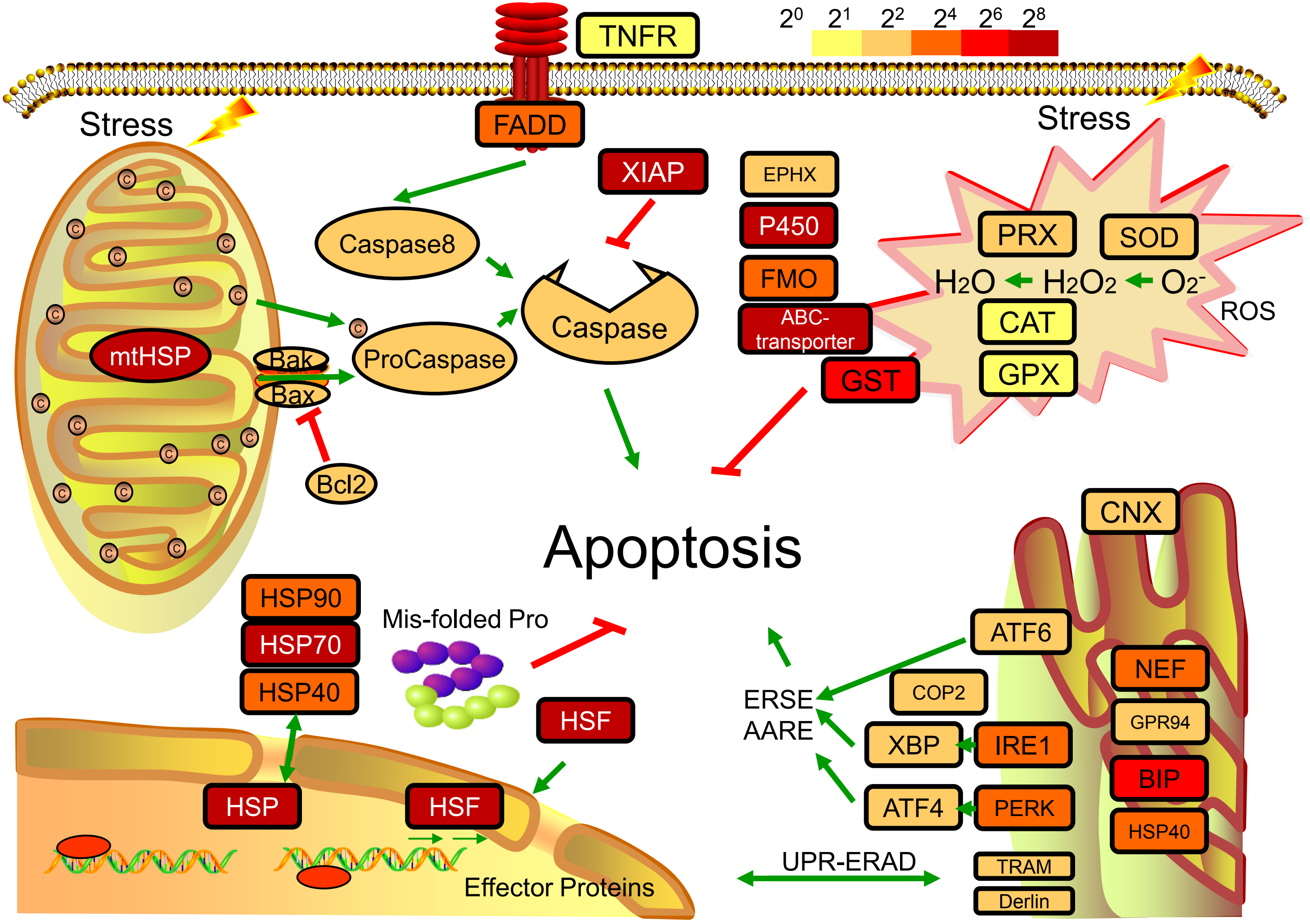

Click here to download Figure Fig.4.pdf

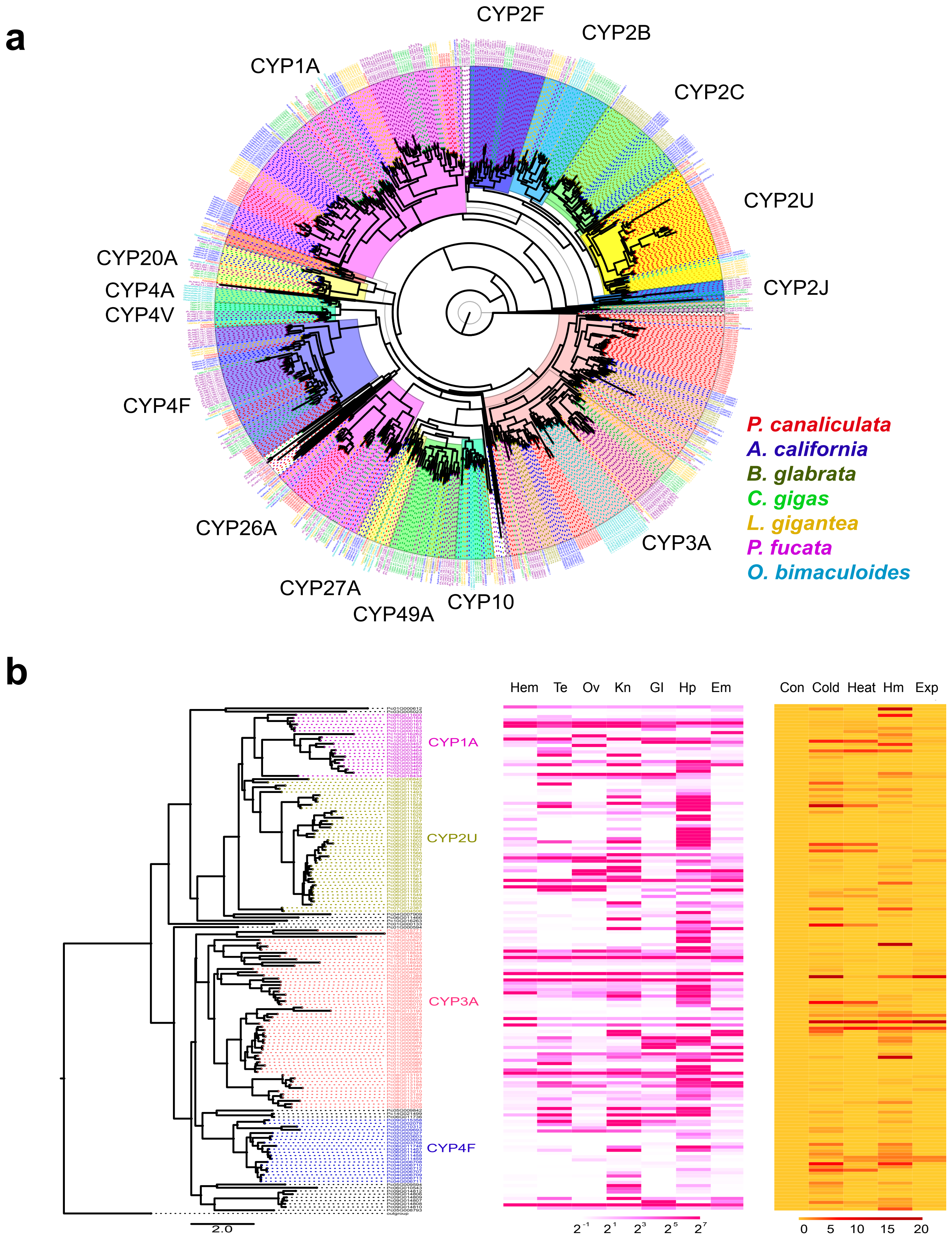

Figure

Click here to download Figure Fig.5.pdf

a

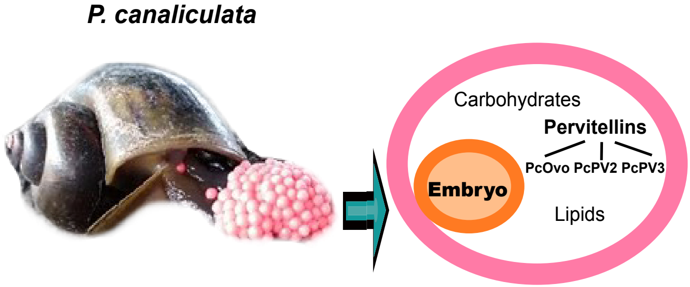

b

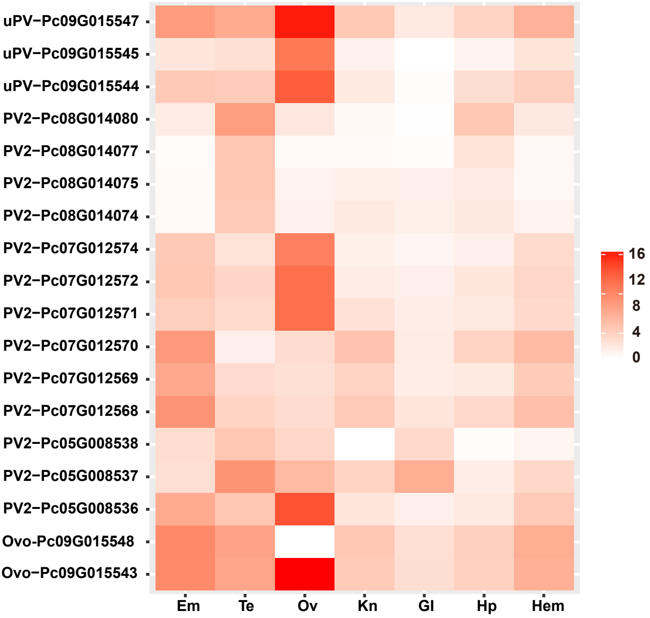

Click here to access/download
Supplementary Material
Supplemental Information.doc

 Click here to download Personal Cover Cover letter.doc

Dear Laurie and Scott,

We are delighted to submit our genome paper of golden apple snail to GigaScience. We appreciate any of your advices.

It was 8 years ago that I worked on the panda genome project in BGI, and I have always been grateful for Laurie's kind revision of that manuscript published in *Nature*. It was 6 years ago that I wrote a review paper on the sequence assembly algorithm, and I was in debt to Scott for helping me revise that manuscript later published on Briefings in Functional Genomics. Now I am working at Agricultural Genomics Institute, Chinese Academy of Agricultural Sciences, being a PI researcher in agricultural genomics, and focusing mainly in pest animals and microbiome.

The golden apple snail is an important worldwide invasive animal, listed in the top-100 worst invasive species. It has become a major pest in the rice field, causing huge economic loss each year but lack of efficient preventing approaches. By PacBio sequencing and Hi-C technology, we have assembled the genome into 14 chromosomes, which is the best available genome sequence in Molluscs. Key findings include the recent explosion of DNA/hAT-Charlie TEs, the expansion of P450 gene family and the constitution of cellular homeostasis system, contributing to the ecological plasticity in the stress adaptation, as well as the perivitellin gene expansion and high transcriptional level in ovary that promotes the function of nutrients supplying and defense ability in the eggs. We also analyzed the gut metagenome and found rich genes for food digestion and xenobiotics degradation. The golden apple snail possesses potential to be a model organism of molluscs, and we believe that with a high-quality reference genome, it will become more important in molluscs researches.

Thank you for your consideration. We would really appreciate if you could accelerate the processing of our manuscript given a highly competitive situation.

Best wishes,

Wei Fan
