# Supplementary material for: The genome of the golden apple snail Pomacea canaliculata provides insight into stress tolerance and invasive adaptation
Source: Gigascience. 2018 Aug 9;7(9):giy101. doi: 10.1093/gigascience/giy101 (PMC6129957; doi:10.1093/gigascience/giy101)
Supplement: GIGA-D-18-00030_Revision_1.pdf [file giy101_giga-d-18-00030_revision_1.pdf]

GigaScience

# The genome of golden apple snail Pomacea canaliculata provides insight into stress tolerance and invasive adaptation

--Manuscript Draft--

| | |
|---|---|
| **Manuscript Number:** | GIGA-D-18-00030R1 |
| **Full Title:** | The genome of golden apple snail Pomacea canaliculata provides insight into stress tolerance and invasive adaptation |
| **Article Type:** | Research |
| **Funding Information:** | National key research and development program of China (2016YFC1200600) — Dr Wei Fan<br>Shenzhen science and technology program (JCYJ20150630165133395) — Dr Wei Fan<br>Fund of Key Laboratory of Shenzhen (ZDSYS20141118170111640) — Dr Wei Fan<br>The Agricultural Science and Technology Innovation Program (ASTIP) of Chinese Academy of Agricultural Sciences(CAAS) & Elite Youth Program of Chinese Academy of Agricultural Sciences — Dr Wei Fan |


**Abstract:**

Background: The golden apple snail (Pomacea canaliculata) is a fresh water snail listed among the top-100 worst invasive species, worldwide and a noted agricultural and quarantine pest that causes great economic losses. It is characterized by fast growth, strong stress tolerance, a high reproduction rate, and adaptation to a broad range of environments.
Results: Here, we used long-read sequencing to produce a 440-Mb high-quality chromosome-level assembly for the P. canaliculata genome. In total, 50 Mb (11.4%) repeat sequences and 21,533 gene models were identified in the genome. The major findings of this study include the recent explosion of DNA/hAT-Charlie transposable elements (TEs), the expansion of the P450 gene family and the constitution of the cellular homeostasis system, which contributes to ecological plasticity in stress adaptation. In addition, the high transcriptional levels of perivitellin genes in the ovary and albumen gland promote the function of nutrient supply and defence ability in eggs. Furthermore, the gut metagenome also contains diverse genes for food digestion and xenobiotic degradation.
Conclusions: These findings collectively provide novel insight into the molecular mechanisms of the ecological plasticity and high invasiveness.



| | |
|---|---|
| **Corresponding Author:** | Wei Fan<br>Chinese Academy of Agricultural Sciences<br>CHINA |
| **Corresponding Author Secondary Information:** | |
| **Corresponding Author's Institution:** | Chinese Academy of Agricultural Sciences |
| **Corresponding Author's Secondary Institution:** | |
| **First Author:** | Conghui Liu |
| **First Author Secondary Information:** | |
| **Order of Authors:** | Conghui Liu<br>Yan Zhang<br>Yuwei Ren<br>Hengchao Wang<br>Shuqu Li |


Powered by Editorial Manager® and ProduXion Manager® from Aries Systems Corporation

| | |
|---|---|
| | Fan Jiang |
| | Lijuan Yin |
| | Guojie Zhang |
| | Wanqiang Qian |
| | Bo Liu |
| | Wei Fan |
| **Order of Authors Secondary Information:** | |
| **Response to Reviewers:** | GIGA-D-18-00030<br>The genome of golden apple snail Pomacea canaliculata provides insight into stress tolerance and invasive adaptation<br>Conghui Liu; Bo Liu; Yuwei Ren; Yan Zhang; Hengchao Wang; Shuqu Li; Fan Jiang; Lijuan Yin; Guojie Zhang; Wanqiang Qian; Wei Fan<br>GigaScience<br><br>Dear Dr Fan,<br><br>Your manuscript "The genome of golden apple snail Pomacea canaliculata provides insight into stress tolerance and invasive adaptation" (GIGA-D-18-00030) has been assessed by our reviewers. Although it is of interest, we are unable to consider it for publication in its current form. The reviewers have raised a number of points which we believe would improve the manuscript and may allow a revised version to be published in GigaScience.<br>Reply: we have made revisions according to the reviewer's suggestions.<br><br>Their reports, together with any other comments, are below. Please also take a moment to check our website at https://giga.editorialmanager.com/ for any additional comments that were saved as attachments.<br><br>Please consider including more recent data on other molluscs in your analyses - see the report of reviewer 3 below.<br>Reply: We have added a new mollusc species "golden mussel Limnoperna fortunei", and replaced the data for "pearl oyster Pinctada fucata" with the latest version.<br><br>Please carefully revise the manuscript for language use and grammar, ideally with the help of a native speaker. Please note the attached file of one of the reviewers (available via Editorial Manager), which contains some suggestions for improvements<br>Reply: We have revised the language and grammar, and asked a native speaker for polishing. We also adopted the suggestions from the attached file of one of the reviewers.<br><br>Author roles: I note that you indicate four "equally contributing" first authors. Please note that we cannot indicate more than three "equally contributing" co-first authors, and please be aware that shared first authorship is reserved for exceptional cases where the contribution of two or three authors is indeed exactly equal.<br>Reply: We have reduced the co-first authors to three.<br><br>If you are able to fully address these points, we would encourage you to submit a revised manuscript to GigaScience. Once you have made the necessary corrections, please submit online at:<br><br>https://giga.editorialmanager.com/<br><br>Reply: After fully addressed all the points, we re-submitted the manuscript to GigaScience.<br><br>If you have forgotten your username or password please use the "Send Login Details" link to get your login information. For security reasons, your password will be reset.<br><br>Please include a point-by-point within the 'Response to Reviewers' box in the submission system. Please ensure you describe additional experiments that were |

carried out and include a detailed rebuttal of any criticisms or requested revisions that you disagreed with. Please also ensure that your revised manuscript conforms to the journal style, which can be found in the Instructions for Authors on the journal homepage.

The due date for submitting the revised version of your article is 21 Jun 2018.

I look forward to receiving your revised manuscript soon.

Best wishes,

Hans Zauner
GigaScience
www.gigasciencejournal.com

Reviewer reports:

Reviewer #1: In their manuscript Liu et al. reported the genome sequence of the golden apple snail Pomacea canaliculata. They constructed chromosomal-level genome assembly using HiSeq, PacBio, and Hi-C sequencing technologies. They also tested differential gene expression under various environmental stress, showing many genes are responded to maintain homeostasis. In addition, they sequenced gut metagenome of the snail for the first time, implying that microorganisms contribute to digestion and resistance to xenobiotics of the host animal.

I think the massive data provides fundamental information to understand the biology of the animal as well as molluscs, therefore the study is valuable to be published in the journal GigaScience after some corrections.

Overall, the methods are appropriate, but description and interpretation of the results look not sufficient in some points as shown below.

P. 5, lines 94-96
"such as Califonia sea hare, Pacific oyster, Pearl oyster,..."
should be "such as the Califonia sea hare, the Pacific oyster, the pearl oyster,..."
There are many mistakes like this. I won't mention all of them. Please consult professional English editor before submitting the revision.
Reply: We have corrected this mistake in the new submitted manuscript.

P. 7, lines 148-150
"genes from seven related species..."
In fact eight species including Pinctada fucata were analyzed in figures 2a and 4a. Takeuchi et al.(2016, Zoological Letters, 2:3) and Luo et al.(2015, Nature Communications, 6, 8301) should be referred for P. fucata and Lingula anatina genome data, respectively.
Reply: We have corrected the species number. Because a new species is added into analysis, now the total species number is nine.  The reference paper of the new species “Uliano-Silva M, Dondero F, Dan Otto T, Costa I, Lima NCB, Americo JA, et al. A hybrid-hierarchical genome assembly strategy to sequence the invasive golden mussel Limnoperna fortunei. Gigascience. 2017. doi: 10.1093/gigascience/gix128. ” were also added at line 104 in the new submitted manuscript.

In addition, please carefully correct scientific names in Abbreviations and figures.
"Lottia gigantean" should be "Lottia giganta"
"Aplysia california" should be "Aplysia californica."
"Lingula anatine" should be "Lingula anatina"
Reply: we have corrected all the mistakes on scientific names in Abbreviations and figures.

P. 9 178-179
From the results I could not understand how the idea that the "DNA/hAT-Charlie TEs... promote the potential plasticity in the stress adaptation" came. This hypothesis can be tested using the present RNA-seq data, by checking whether the TEs are up-regulated under the stresses.

Reply: Transposons can insert into any genomic regions, which may change the gene regulations, or modify the gene structure thus form new functions. If a genome has high transposon activity, then it has high ability to adapt to the changing environment, so the recent explosion of DNA TEs may benefit the fast evolution of P. canaliculata in the recent history. There were several previous studies (Hua-Van A, Le Rouzic A, Boutin TS, Filée J, Capy P. The struggle for life of the genome's selfish architects. Biol Direct. 2011;6:19; Werren JH. Selfish genetic elements, genetic conflict, and evolutionary innovation. Proc Natl Acad Sci U S A. 2011;108:10863-70) on this issue that provides evidences that TEs can introduce small adaptive changes for a species. Using the RNA-seq data to resolve this question is good idea. In our understanding, TEs can't be transcribed and translated as an independent element, except for some low and random transcriptions which are likely to be no functions. So we analyzed the expression of 709 genes including DNA elements that restricted to the 4% peak inside the gene region, compared with the other genes that outside the 4% peak. Differentially expressed genes (DEG) were defined here by P-value smaller than 0.05 for comparison of treatments (heat, cold, heavy metal and air exposure) and control data. The percent of DEGs in the 4% peak were higher than those of genes outside the peak (10.2% higher for heat, 8.6% higher for cold, 8.6% higher for heavy metal, and 7.3% higher for air exposure). Among the DEGs in the 4% peak, about half are up-regulated and the other half are down-regulated. Moreover, the DEGs in the 4% peak were mainly enriched in cellular metabolic process, response to stimulus, localization and signaling by GO annotation. These results indicated that genes in the 4% peak were likely to be more active in the response of stimulus, promoting the potential plasticity in the stress adaptation.
The figure and related context was added in the new manuscript.

P.11 lines 232-236
The authors claimed that the P. canaliculata CYP gene family expanded compare to other molluscs. But the gene expansion of CYP looks common among molluscs. The number of the gene in P. canaliculata didn't significantly stand out from other molluscs (for example P. canaliculata has 157 genes and the Pacific oyster has 135). A molecular phylogeny in Fig 4a shows that lineage-specific gene expansion of CYP occurs not only in P. canaliculata but also other molluscs.
Reply: We appreciate the reviewer's comments. We claimed CYP450 family as expansion for two reasons. 1) Although the gene number of CYP450 in P. canaliculata was close to C.gigas (135) and A. californica (128), the gene ratio of "CYP450 genes/total genes in genome" was distinct, namely P. canaliculata (157/21553,~0.0073), C.gigas (135/46748, ~0.0029) and A. californica (128/27591, ~0.0046). 2) The expansion was more obvious in special subfamily, such as CYP3A (P.c 56, A.c 24, Bg 21, C.g 12, L.g 10, P.f 23 and O.b 20 ) and CYP 2U (P.c 42 A.c 5, Bg 2, C.g 0, L.g 8, P.f 0 and O.b 2) (figure 4). However, we weaken the mood when refer to expansion and withdraw the adjective "great".

P. 17 lines 346-354
"The rich phenotypic... in laboratory."
These sentences should be move to Introduction.
Reply: We have moved "The rich phenotypic... in the laboratory." into introduction part between line 71 and 80.

P. 18 lines 380-381
"total messenger RNAs"
Total RNA or messenger RNA?
Reply: Here we mean "messenger RNA". The sentence was revised to be "In final, total RNAs were extracted from the stored tissues of P. canaliculata materials, and then mRNAs were pulled out by beads with poly-T for constructing cDNA libraries."

P. 21 line 445
Please cite the literature of "previous results."
Reply: We have added the literature "Sun J, Zhang Y, Xu T, Zhang Y, Mu H, Zhang Y, et al. Adaptation to deep-sea chemosynthetic environments as revealed by mussel genomes. Nature Ecology & Evolution. 2017.1(5), 121; Benton MJ, Donoghue, PCJ,

Powered by Editorial Manager® and ProduXion Manager® from Aries Systems Corporation

Asher RJ. in The Timetree of Life:Calibrating and Constraining Molecular Clocks (eds Hedges, S. B. & Kumar, S.)35–86 Oxford Univ. Press, 2009; Zapata F, Wilson NG, Howison M, Andrade SC, Jörger KM, Schrödl M, Goetz FE, Giribet G, Dunn CW. Phylogenomic analyses of deep gastropod relationships reject Orthogastropoda. Proc Biol Sci. 2014 281:20141739. doi: 10.1098/rspb.2014.1739" in revised version.

Figure 2
The title "Evolutionary genomic analysis between P. canaliculata and other molluscs" is not appropriate because Lingula is a brachiopod.
Reply: The title of Figure 2 is changed to "Evolutionary genomic analysis of P. canaliculata", because our focus is the species of P. canaliculata, other species were used for comparison.

Figure 4
Method for molecular phylogeny construction of CYP genes should be described.
Reply: The method was described in Figure 4 legend "The tree was constructed using the maximum likelihood method in MEGA7, and the branch length scale indicates the average number of residue substitutions per site".

Figure S1
Which K-mer size used?
Reply: here we used 17-mer, the K-mer size is 17.

Table S4, S5, and S7
It is not reader-friendly to show the huge data in a table. I couldn't recognize what is the message of the data. Why not visualize the data in a heat map like Fig4b.
Reply: A supplemental figure S6 corresponding to Table S4 was added.
Data in Table S5 was corresponding to the color in the heatmap of figure 4b, Data in Table S7 was corresponding to the color in the heatmap of figure 5b, so there is no need to add other heat map figures.

Table S9
What "Mean" and "SD" indicate? E-value of blast results? Please describe.
Reply: "Mean" and "SD" indicate the mean and standard deviation of relative abundance of a phylum or a genus from the 6 gut microbiota samples. We also added a note under the Table.

Reviewer 2: - [the reviewer has no specific comments to the authors at this point, but recommends careful improvements of language and grammar]
Reply: We have improved the language and grammar, and polished the text by native speakers.

Reviewer #3: This manuscript presents a high-quality genome assembly for the snail P. canaiculata. Such genome and further analysis presented will contribute deeply for future studies of the molecular evolution and adaptation of molluscs, as well as to the study of the molecular mechanisms leading to - or involved with - invasive species success. I also point out the relevance of a first qualitative description of a high-depth gut microbiome for a snail. For such reasons, I recommend the publication of this manuscript. Nevertheless, I would like to recommend some essential revision prior publication.

First, the English has to be revised. I'll give a few examples bellow, and authors will find major marks in purple concerning specifically the need of English revision in the revised pdf attached. However, the entire manuscript would benefit from a native English speaker revision.
Reply: we have revised the descriptions highlighted in the attached pdf, and also asked a native speaker to help polish the language.

Examples of sentences needing English revision:

Lines 50-51: "causing severe economic loss each year as a result of yield loss,

replanting cost and the funds of control." - rephrasing necessary.
Reply: This sentence is modified to “causing severe economic losses each year as a result of yield loss, replanting cost and expenditures on control.”

Line 52: "More seriously, P. canaliculata has involved in the transmission of a human fatal disease."
Reply: This sentence is modified to “More seriously, P. canaliculata has been involved in the transmission of a fatal human disease,”

Line 57: "causing great challenge to human health"
- rephrasing advised.
Reply: This sentence is modified to “creating a great challenge in terms of human health.”

Line 58: "Molluscs is …" - English correction necessary.
Reply: This sentence is modified to “Molluscs are a highly diverse group, second only to arthropods in species number, and their high biodiversity makes them an excellent model to address issues such as biogeography, adaptability and evolutionary processes.”

Lines 92-94: "However, researches at whole genome level in P. canaliculata still lags far behind other mollusks species, due to the lack of a high-quality reference genome. By far, multiple draft…" - rewriting necessary.
Reply: This sentence is modified to “However, research at the whole-genome level in P. canaliculata still lags far behind that in other mollusc species due to the lack of a high-quality reference genome. Multiple draft genomes of molluscs have been published, including the genomes of the California sea hare, Pacific oyster, pearl oyster, owl limpet, California two-spot octopus, golden mussel, and Biomphalaria snails, greatly promoting research on mollusc genomics.”

Line 263: "was" should be "were".
Reply: Corrected in the new manuscript.

Data and analysis related comments:

Lines 36-37: The description of the genome and the several molecular expression data are great contributions for the further understanding of molluscan and invasive biology. Nevertheless, we should avoid direct jumps to conclusions such as in lines 36 and 37, as the results in the manuscript don't present tools or direct ways to prevent invasions or pathogen transmission. I advise the withdraw of such sentence.
Reply: We agree to the suggestion, and have removed that sentence “Our results not only strengthen the understanding of molluscs genomics and biological invasion, but also benefit preventing the invasion of apple snail and transmission of pathogenetic parasites.”

Line 47: I would rephrase the sentence here in line 47. Even though the biology of the species may positively influence its invasive capacity, such characteristics are not exclusive of invasive mollusks. For that reason, I would exclude the "was due to" (line 47) which implies causality.
Reply: In the revised manuscript, we rephrased “was due to” to be “is closely related to”.

Line 63: Please present and refer to the lower temperature the species can establish populations in.
Reply: We added a sentence “P. canaliculata has been reported to establish populations at temperatures ranged from 10 °C to 35 °C” in the new manuscript, as well as two reference papers (Seuffert ME, Burela S, Martín PR. Influence of water temperature on the activity of the freshwater snail Pomacea canaliculata (Caenogastropoda: Ampullariidae) at its southernmost limit (Southern Pampas, Argentina). Journal of Thermal Biology. 2010; 35:77-84; Matsukura K, Tsumuki H,Izumi Y, Wada T. Physiological response to low temperature in the freshwater apple snail, Pomacea canaliculata (Gastropoda: Ampullariidae). J Exp Biol. 2009;212:2558-63).

| | |
|---|---|
| | Line 95: I would cite here also the draft genome of the invasive Limnoperna fortunei mussel.<br>Reply: The golden mussel “Limnoperna fortunei” and the related article were added in the new manuscript.<br><br>Line 95: There is a new version of the Pearl oyster published. If analysis were performed with data cited in line 95, I would advise for updating the analysis with proteins from the new genome (Du X, Fan G, Jiao Y et al. The pearl oyster Pinctada fucata martensii genome and multi-omic analyses provide insights into biomineralization. Gigascience 2017;6(8):1-12).<br>Reply: We have replaced the proteins data of Pinctada fucata to the latest version, and updated all the analysis in the new manuscript.<br><br>Line 100-101: Rephrasing is necessary as cellular homeostasis, color and nutrient of the eggs are not species-specific invasive characteristics.<br>Reply: We revised “invasive characters” to “environmental adaptation characteristics”.<br><br>Line 104-105: same argument as for lines 36-37. Some rephrasing starting from "interrupt transmission…" is necessary.<br>Reply: We agree with the suggestion, and weakened the mood. The sentence is modified to “and provide a basis for interrupting the transmission of pathogenetic nematode parasites”.<br><br>Table S1: Table S1 would benefit of having 2 columns: one with (i) number of reads generated and (ii) total bp produced for each library, instead of having a column 'Data size' (and what G bp means?).<br>Reply: We have made 2 columns in Table S1 according to the suggestions. One column refers to number of sequenced reads, the other column refers to number of sequenced bases.<br><br>Line 122: The ratio of genome coverage by reads used as input in the assembly? Rephrase it together with the sentences in lines 126-127, please.<br>Reply: In this sentence “another important aspect for evaluating genome assembly is the ratio of genome coverage.” (between line 132 and 133), we want to explain that the ratio of assembly coverage is important. In P. canaliculata, the genome size of 446 Mb was estimated by the distribution of k-mer frequency. In this assembly genome, ~98.6 % sequence has been assembled.<br>In the sentence “we mapped the Illumina shotgun reads to the assembled reference genome. Significantly, 97% and 95% of the genome-derived and transcriptome-derived reads, respectively, could be aligned to the reference genome,” (between line 136 and 137), we want to confirm the accuracy and no obvious bias for sequencing and assembly.<br><br>Line 123-124 and line 403: Please estimate and present the levels of heterozygosity using the illumina reads.<br>Reply: We used K-mer with K-size 17 to estimate the genome heterozygosity based on algorithm from reference (Liu B, et al. Quantitative Biology 2013:arXiv:1308.2012 [q-bio.GN]). The estimated heterozygosity of P. canaliculata range from 1% to 2%. In addition, we also used FIndError (Gnerre S et al., 2011) in the Allpath-LG package to estimate the heterozygosity, the result is 1.75%, consistent with the first method.<br>We have added it in revised manuscript. “With an estimated genome size of 446 Mb and genome heterozygosity between 1% and 2% based on the distribution of k-mer frequency.”<br><br>Line 415-416: "Then, the protein-coding sequences were mapped by RNA-seq data." - please explain this sentence.<br>Reply: To determine whether the predicted genes are expressed or not, we used the transcriptome data to map to the CDS of genes. The gene models were retained if they had at least one supporting evidence from UniProt database, InterProScan domain and RNA-seq data.<br>To be more clear, we have revised this sentence in the new manuscript: “Then, these gene models were annotated by RNA-seq data, UniProt database and InterProScan software”. |

Powered by Editorial Manager® and ProduXion Manager® from Aries Systems Corporation

Line 163: Withdraw "and so on".
Reply： "and so on" is removed.

Lines 146-163: To start understanding if the genome composition itself - and not only regulation of gene expression - can play a major role in the success of invasive species, I would advise to compare gene family expansions and contractions between the genomes of two invasive mollusks, which is now possible once the draft genome of L. fortunei is available (GigaScience doi: 10.1093/gigascience/gix128.). Further discussion about the presence - or lack thereof - of common expansions and contractions of gene families would be a great contribution. Such gene families could be further investigated for their roles in the expression of phenotypes related to invasive ecology and behaviour. I would strongly suggest for a comparative analysis of P. canaliculata and L. fortunei protein sets leading to a new Figure S4 and brief discussion on the findings.
Reply: We agree with the reviewer's comments. In the revised version, we added the genome data of L. fortune to re-construct the orthoFinder ortholog and paralog gene families. Then, we identified the common expanded gene families both in P. canaliculata and L. fortune. The functions of these gene families are mainly enriched in signal transduction, replication and repair, Translation, glycan biosynthesis and metabolism, Lipid metabolism, endocrine, immune and nervous system. And we have revised the results in Figure S4.

Line 171-172: "interestingly, only the results of DNA transposons showed a unique peak at ~4% divergence rate for P. canaliculata and C. gigas" - rewrite this sentence.
Reply: We rephrased it as "Notably, the TE class of DNA transposons showed a specific peak at a divergence rate of ~4% divergence rate for P. canaliculata and C. gigas".

Line 249: Please indicate how many and which genes were highly induced to facilitate further investigation by other groups in the future.
Reply: Gene IDs"Pc06G011748, Pc06G011460, Pc06G011458, Pc06G011459, Pc04G006708, Pc04G006710 and Pc04G006707"were added.

Line 254 -257: This direct link between phenotype and molecular characteristics cannot be supported by your data. Please rephrase it.
Reply: We revised these sentences in the new manuscript:
"P. canaliculata has eggs characterized by abundant nutrients, reddish or pinkish colour, aerial oviposition and neurotoxicity due to the perivitelline Fluid (PVF), which fills the space between the eggshell and the embryo and consists of carbohydrates, lipids and proteins (Figure 5a)."

Line 264- 269: Please clarify what was performed here. In any case, blast alone is not the best tool to predict orthology. I would use RBBH methods.
Reply: In the revised manuscript, we used the reported 59 PVF protein fragments as query to identify the PVF genes from P. canaliculata reference genes by blastp (e-value 10-5), and further used the requirements of more than 85% sequence identity and over half alignment length for the query to get 36 best hits, corresponding to 28 candidate P. canaliculata PVF genes, and then confirmed 6 perivitellin genes which encode the subunits of PcOvo, PcPV2, and PcPV3, according to their high RNA expression in ovary and albumen gland tissue.
As OrthoFinder could analyze the orthology and paralogy of more than two species at the same time, we utilized the OrthoFinder results to investigate the ortholog and paralog relationships of these P. canaliculata PVF genes compared with other 8 sequenced mollusc species. Notably, 5 of the 6 perivitellin genes fall into single-gene families. In other words, it is hard to detect any homologs for most of these perivitellin genes in other sequenced mollusc species. One reason may be that the divergence time is too long (>200 Mya), another reason may be that these major PVF genes may have experienced fast evolution in the history, in order to adapt to the changing environment. At last, we used the RBBH method (reciprocal best hit) to identify ortholog genes between each species pair, and the result is consistent with that of OrthoFinder.

Line 327: Conclusion and discussion? At this point, only conclusions should be stated.

Powered by Editorial Manager® and ProduXion Manager® from Aries Systems Corporation

| | |
|---|---|
| | Please eliminate sentences from 346 to 359. [condense and add into introduction]<br>Reply: We have condensed this paragraph, and moved it to the introduction part between line 376 and line 382. The revised paragraph is:<br>"In this study, we report a fine reference genome of P. canaliculata, first chromosome-level Mollusca genome published. With its easy acquisition, rapid growth and efficient reproduction, P. canaliculata possesses the potential to be a model organism of Mollusca. As its the cellular complexity and conservation of pathways also make P. canaliculata a useful representative of Mollusca, the genome described in this study can be used to advance our understanding of the molecular mechanisms involved in various scientific questions regarding Mollusca."<br><br>Line 395: Please indicate software used for trimming.<br>Reply: We used an in-house software for trimming (clean_dapter, clean_lowqual, filter_unpaired_reads.pl), which is freely available at Github " https://github.com/fanagislab/common_use".<br><br>Line 424-431: I would state the masking before stating the gene prediction. Rewrite.<br>Reply: We have moved the repeat paragraph before the gene prediction paragraph.<br><br>Line 448: Any trimming performed for the transcriptome?<br>Reply: Yes, we use the same method. This sentence is revised to:<br>"Transcriptome reads were trimmed with the same method for genomic reads (https://github.com/fanagislab/common_use), and then mapped to the reference genome of P. canaliculata using TopHat (v. 2.1.0) with default settings".<br><br>Line 591: Please make available a supplementary material with the IDs of all sequences presented in Figure 4b. Please explain the scale in the heat maps of figure 4b.<br>Reply: The IDs are listed in Supplemental table S5.<br>We explain the scale meaning in the legend of figure 4b. The scale for the left heat map represent FPKM value, showing by gradually changing colors; The scale for the right heat map represent fold change (FPKM-stimulus/FPKM-control), showing by gradually changing colors. To be more clear, we also add two marks "FPKM" and "Fold-change" alongside the scale on the figure.<br>--<br>Please also take a moment to check our website at https://giga.editorialmanager.com/l.asp?i=38889&l=8DS0D5CA for any additional comments that were saved as attachments. Please note that as GigaScience has a policy of open peer review, you will be able to see the names of the reviewers. |
| **Additional Information:** | |
| **Question** | **Response** |
| Are you submitting this manuscript to a special series or article collection? | No |
| **Experimental design and statistics**<br><br>Full details of the experimental design and statistical methods used should be given in the Methods section, as detailed in our Minimum Standards Reporting Checklist. Information essential to interpreting the data presented should be made available in the figure legends.<br><br>Have you included all the information requested in your manuscript? | Yes |

| | |
|---|---|
| **Resources**<br><br>A description of all resources used, including antibodies, cell lines, animals and software tools, with enough information to allow them to be uniquely identified, should be included in the Methods section. Authors are strongly encouraged to cite Research Resource Identifiers (RRIDs) for antibodies, model organisms and tools, where possible.<br><br>Have you included the information requested as detailed in our Minimum Standards Reporting Checklist? | Yes |
| **Availability of data and materials**<br><br>All datasets and code on which the conclusions of the paper rely must be either included in your submission or deposited in publicly available repositories (where available and ethically appropriate), referencing such data using a unique identifier in the references and in the “Availability of Data and Materials” section of your manuscript.<br><br>Have you have met the above requirement as detailed in our Minimum Standards Reporting Checklist? | Yes |

Powered by Editorial Manager® and ProduXion Manager® from Aries Systems Corporation

Manuscript

Click here to download Manuscript PC_manuscript_revised-final.docx

# The genome of the golden apple snail *Pomacea canaliculata* provides insight into stress tolerance and invasive adaptation

Conghui Liu[1]*, Yan Zhang[1]*, Yuwei Ren[1]*, Hengchao Wang[1], Shuqu Li[1], Fan Jiang[1], Lijuan Yin[1], Guojie Zhang[2], Wanqiang Qian[1]†, Bo Liu[1]†, Wei Fan[1]†

[1]Agricultural Genomic Institute, Chinese Academy of Agricultural Sciences, Shenzhen, Guangdong, 518120, China.

[2]BGI-Shenzhen, Shenzhen, Guangdong, 518083, China

Conghui Liu: rapherlch@163.com; Yan Zhang: milrazhang@163.com; Yuwei Ren: xiaoshudaxia@126.com; Hengchao Wang: wanghengchao000@qq.com; Shuqu Li: lishuqu1234@163.com; Fan Jiang: greatjf@163.com; Lijuan Yin: yinlijuan1005@163.com; Guojie Zhang: guojie.zhang@bio.ku.dk

*These authors contributed equally to this work.

†Correspondence should be addressed to Wanqiang Qian (qianwanqiang@caas.cn), Bo Liu (lb_bobo@aliyun.com) or Wei Fan (fanwei@caas.cn).

## Abstract

**Background:** The golden apple snail (*Pomacea canaliculata*) is a fresh water snail listed among the top-100 worst invasive species, worldwide and a noted agricultural and quarantine pest that causes great economic losses. It is characterized by fast growth, strong stress tolerance, a high reproduction rate, and adaptation to a broad range of environments.

**Results:** Here, we used long-read sequencing to produce a 440-Mb high-quality chromosome-level assembly for the *P. canaliculata* genome. In total, 50 Mb (11.4%)

repeat sequences and 21,533 gene models were identified in the genome. The major findings of this study include the recent explosion of DNA/hAT-Charlie transposable elements (TEs), the expansion of the P450 gene family and the constitution of the cellular homeostasis system, which contributes to ecological plasticity in stress adaptation. In addition, the high transcriptional levels of perivitellin genes in the ovary and albumen gland promote the function of nutrient supply and defence ability in eggs. Furthermore, the gut metagenome also contains diverse genes for food digestion and xenobiotic degradation.

**Conclusions:** These findings collectively provide novel insight into the molecular mechanisms of the ecological plasticity and high invasiveness.

**Keywords:** golden apple snail, *Pomacea canaliculata*, genome, adaptive evolution, stress tolerance, P450, reproduction, perivitelline, metagenome

## Background

The golden apple snail *Pomacea canaliculata* (family Ampullariidae, order Architaenioglossa) is a fresh water snail listed among the world's top 100 worst invasive species [1] and is considered an agricultural and quarantine pest worldwide [2]. Native to tropical and subtropical South America, *P. canaliculata* gradually spread to non-indigenous regions, such as Southeast and East Asia [3], Africa [4], North America [5], Oceania [6] and even Europe [7]. Its successful biological invasion was closely related to its polyphagous feeding habits [8], voracious appetite [9], broad environmental adaptability [10] and rapid growth and

high rate of reproduction [11]. In addition to its ecological impact, *P. canaliculata* ravages a wide range of crops, including grains, fruits and vegetables [12], causing severe economic losses each year as a result of yield loss, replanting cost and expenditures on control (https://www.cabi.org/isc/datasheet/68490). More seriously, *P. canaliculata* has been involved in the transmission of a fatal human disease, eosinophilic meningitis, that first appeared in East Asia where people frequently consume these snails [13]. During this pathophoresis, *P. canaliculata* acts as an important intermediate host of the pathogenic parasite *Angiostrongylus cantonensis*, and the range of infected regions is still expanding, creating a great challenge in terms of human health [14, 15].

Molluscs are a highly diverse group, second only to arthropods in species number [16], and their high biodiversity makes them an excellent model to address issues such as biogeography, adaptability and evolutionary processes [17]. The worldwide invasive species *P. canaliculata* provides valuable potential in these fields [18]. As a primitive circumtropical species, *P. canaliculata* possesses strong ecological plasticity with many advantages, including low-temperature resistance [19] and drought tolerance [20], which has contributed to its competitive success in resource acquisition. *P. canaliculata* has been reported to establish populations at temperatures ranging from 10 °C to 35 °C [19, 21]. Additionally, *P. canaliculata* tolerates heavy metal contamination. When living in contaminated water, the gill is enriched with a high concentration of heavy metals, and histopathological changes in the digestive tract are detected; however, an extremely low mortality rate is observed [22]. The conspicuous

colouration and neurotoxic lectin could confer a survival advantage on the eggs, defending the embryos against potential predators [23]. Moreover, an immune-neuroendocrine system can also be detected in *P. canaliculata*, as demonstrated by the existence of a specific immune memory after bacterial challenge [24, 25], broadening the study of invertebrate immunology.

The rich phenotypic and genetic diversity of molluscs makes them an excellent species group for addressing many important issues in evolution, ecology and function. However, the genomic resources on Mollusca are still insufficient compared with those of other close phyla, such as Arthropoda and Nematoda, and few molluscs can be employed as model organisms. *P. canaliculata*, however, possesses the potential to be a model organism among molluscs because of several inherent characteristics. For example, *P. canaliculata* is easy to acquire because it has a broad global distribution originating from a primarily circumtropical environment. Moreover, its high adaptability, rapid growth and efficient reproduction facilitate the cultivation of *P. canaliculata* in the laboratory.

In recent years, the genomic features of *P. canaliculata* have been increasingly studied. After the discovery of 14 pachytene bivalents in the karyotype [26], molecular markers were identified to investigate the genetic diversity of the *P. canaliculata* population, including 369 amplified fragment length polymorphism (AFLP) loci [27], 16,717 simple sequence repeats (SSR) [28, 29] and 15,412 single-nucleotide polymorphisms SNPs [30]. In addition, multiple transcriptome analyses have been performed to investigate the adaptation, invasion and immune mechanisms of *P.*

*canaliculata*. For instance, Sun et al. reported 128,436 unigenes based on a de novo assembly of Illumina reads [30]; transcriptome changes in response to heat stress and starving incubation were used to characterize its invasive and adaptive abilities [31, 32]; a transcriptome analysis comparing invasive *P. canaliculata* and indigenous *Cipangopaludina cathayensis* provided insights into biological invasion [29]; and 402 immune-related differentially expressed genes (DEGs) in response to lipopolysaccharide (LPyS) challenge were used to explore the mechanisms of defence against pathogens [33]. Furthermore, proteomics tools such as isobaric tags for relative and absolute quantitation (iTRAQ), and liquid chromatography-tandem mass spectrometry (LC-MS/MS) were also applied in the study of protein expression during estivation and oviposition [34,35], together providing plentiful omics- data for the functional analysis of *P. canaliculata*.

However, research at the whole-genome level in *P. canaliculata* still lags far behind that in other mollusc species due to the lack of a high-quality reference genome. Multiple draft genomes of molluscs have been published, including the genomes of the California sea hare [36], Pacific oyster [37], pearl oyster [38], owl limpet [39], California two-spot octopus [40], golden mussel [41], and *Biomphalaria* snails [42], greatly promoting research on mollusc genomics. In this study, we present a chromosome-level genome assembly of *P. canaliculata* with high-quality gene annotation, transcriptome data from several tissues and under various conditions, and metagenomic data from the intestinal tracts, all of which were then applied to study the species-specific environmental adaptation characteristics, such as the cellular

homeostasis system underlying strong stress and the colour and nutrient contents of the eggs. Our data will not only strengthen the understanding of the evolutionary mechanisms of molluscs and the molecular basis of biological invasion but also foster the development of approaches to control the invasion of *P. canaliculata* and provide a basis for interrupting the transmission of pathogenetic nematode parasites.

## RESULTS

### Complete genome assembly at the chromosome level

We generated 26.6 Gb (60.1 X) of PacBio SMRT raw reads with an average read length of 10.1 kb, and 291 Gb (652.4 X) of Illumina HiSeq paired-end reads with an average read length of 150-250 bp using DNA extracted from a single adult *P. canaliculata* (Table S1). The 24.4 Gb (55.4 X) of clean PacBio SMRT reads that passed quality filtering were assembled by smartdenovo (https://github.com/ruanjue/smartdenovo), resulting in an assembly of 1,234 raw contigs with a total length of 473.6 Mb and an N50 length of 1.0 Mb. After filtering of alternatively heterozygous contigs, the 745 resulting contigs with a total length of 440.1 Mb and an N50 length of 1.1 Mb were taken as the final contigs. Previous karyotype research has shown that the haploid P. *canaliculata* genome consists of 14 chromosomes [26]. Based on the Hi-C data, 439.5 Mb (99.9%) of final contigs were anchored and oriented into 14 large scaffolds, each corresponding to a natural chromosome (Figure 1a and Figure 1b), with the longest 45.4 Mb and the shortest 27.2 Mb. This assembly quality is much better than that of the other molluscan

genomes published thus far (Table 1). In addition to the length and continuity of the assembled sequences, another important aspect for evaluating genome assembly is the ratio of genome coverage. With an estimated genome size of 446 Mb and genome heterozygosity between 1% and 2% based on the distribution of k-mer frequency [43] (Figure S1), ~98.6 % of the *P. canaliculata* genome has been assembled. To further confirm the accuracy and completeness of the assembly, we mapped the Illumina shotgun reads to the assembled reference genome. Significantly, 97% and 95% of the genome-derived and transcriptome-derived reads, respectively, could be aligned to the reference genome, suggesting no obvious bias in sequencing and assembly. Additionally, the mitochondrial genome of *P. canaliculata* was assembled as a single contig 15,707 bp in length, which has 99.9% sequence identity to the published mitochondrial genome (GenBank: KJ739609.1) (Figure S2). This high-quality reference genome provides a good foundation for gene annotation.

The protein-coding genes were predicted on the reference genome by EVM, integrating evidence from *de novo* prediction, transcriptome and homology data. In total, 21,533 gene models were predicted as the reference gene set, with coding regions spanning ~32.2 Mb (7.3 %) of the genome (Table 1 and Table S2). The distribution of CDS length in *P. canaliculata* is similar to that in closely related species (Figure 1c). Overall, 97.5% of the reference genes were supported by transcriptome data, and 98.0% of eukaryote core genes from OrthoDB (http://www.orthodb.org/) were identified in the reference gene set by BUSCO. These results were comparable to those in other published molluscan genomes (Table 1). In

functional annotation, a total of 19,815 (91.9 %) reference genes were annotated by at least one functional database. Specifically, 15,662 (72.7%), 13,769 (63.4%), 17,081 (79.3%), 18,847 (87.5%) and 17,003 (79.9%) reference genes were annotated with the eggNOG, KEGG, NR, InterPro and UniProt databases, respectively (Figure S3).

**Signs of adaptive evolution in *P. canaliculata* genome**

To gain insight into the evolutionary perspective of *P. canaliculata*, a phylogenetic tree was built based on 306 high-confidence single-copy orthologous genes from nine related species (*P. canaliculata, Lottia giganta, Aplysia californica, Biomphalaria glabrata, Crassostrea gigas, Octopus bimaculoides, Pintada fucata, Lingula anatina* and *Limnoperna fortunei*) by PhyML [44] and the divergence time was estimated using MCMCTree [45]. The results show that *P. canaliculata* diverged from the ancestor of *B. glabrata* and *A. californica* 372 million years ago (Mya) and from *L. giganta* 491 Mya (Figure 2a).

Then, the molluscan orthologous genes were investigated for adaptive evolution. Utilizing pairwise protein sequence similarities, gene family clustering was conducted by orthfinder [46]. A total of 239,541 reference genes from the nine species were clustered into 69,582 orthologous groups, among which 14,766 orthologous groups contained at least two genes each. We identified 66 orthologue groups that underwent common expansion in both *P. canaliculata* and *L. fortune* but not the other seven species. The functions of these orthologous groups are mainly related to signal transduction; replication and repair; translation, glycan biosynthesis and metabolism;

lipid metabolism; and the endocrine, immune and nervous systems (Figure S4). These relations suggests that the gene families that underwent expansion may play important roles in adaptation to the environment as invasive species.

The high-coverage genome assembly enables a comprehensive analysis of the transposable elements (TEs), which play multiple roles in driving genome evolution in eukaryotes [47]. In total, we identified 49.6 Mb TE sequences in the assembled *P. canaliculata* genome (Table 1), including 3.4 Mb long terminal repeats (LTRs), 27.2 Mb long interspersed elements (LINEs), 17.5 Mb DNA transposons and 1.5 Mb short interspersed elements (SINEs). Next, we analysed the divergence rate of each class of TEs among the available sequenced mollusc genomes. Notably, the TE class of DNA transposons showed a specific peak at a divergence rate of ~4% divergence rate for *P. canaliculata* and *C. gigas* (Figure 2b), indicating a recent explosion of DNA transposons in these two species. We analysed the expression of 709 genes, including DNA elements restricted to the 4% peak inside the gene region, compared with that of the other genes outside the 4% peak (Figure S5). DEGs were defined here by P-values smaller than 0.05 for comparison of the treatment (heat, cold, heavy metal and air exposure) and control data. The percentages of DEGs in the 4% peak were higher than those of genes outside the peak (10.2% higher for heat, 8.6% higher for cold, 8.6% higher for heavy metal, and 7.3% higher for air exposure). Among the DEGs in the 4% peak, approximately half were up-regulated, and the other half were down-regulated. Moreover, the DEGs in the 4% peak were mainly enriched in cellular metabolic process, response to stimulus, localization and signaling according to GO annotation.

These results indicated that genes in the 4% peak were likely to be more active in the response to stimulus, promoting potential plasticity in stress adaptation. TEs are powerful facilitators of evolution that generate "evolutionary potential" to introduce small adaptive changes within a lineage, and the importance of TEs in stress responses and adaptation has been reported in numerous studies [48,49]. The recent explosion of DNA TEs in *P. canaliculata* could also play an important role in promoting the potential plasticity in stress adaptation.

**Investigation of cellular homeostasis system underlying strong stress adaptation**

The homeostasis system plays a crucial role in stress adaptability, providing the molecular basis for re-establishing dynamic equilibrium after challenges by various environmental stressors, including temperature, air exposure, anthropogenic pollution and pathogens [50]. In this study, we addressed three constituent parts of the cellular homeostasis system, which contributes to the successful ecological plasticity of *P. canaliculata* (Figure 3). The transcriptomes of the hemocytes after different stimuli (cold, heat, heavy metal and air exposure) were also sequenced and analysed to address the potential roles of these genes in the cellular homeostasis system.

The unfolded protein response (UPR) system is the central component of protein homeostasis [51]. Heat shock proteins (HSPs) act as molecular chaperones to maintain correct folding, and heat shock transcription factor 1 (HSF1) is responsible for the transcriptional induction of HSPs [52]. In the *P. canaliculata* genome, 13 HSP70s, 6 HSP90s, 7 HSP40s and 11 HSFs were identified (Table S3), and the

expression of HSP90s and HSFs was highly induced in response to heat, cold, heavy metal and air exposure (Table S4 and Figure S6). Inositol-requiring protein 1 (IRE1), protein kinase RNA-like ER kinase (PERK), and activating transcription factor 6 (ATF6) are three mediators recruited by the endoplasmic reticulum (ER) to regulate the UPR [53]. We found putative coding genes of the three core mediators, their respective downstream transcription factors, and the corresponding recognition chaperones in the *P. canaliculata* genome (Table S3).

The xenobiotic biotransformation system helps the molluscs adapt to toxicants, especially pesticides in aquatic environments [54]. Manual annotation of this genome identified 157 cytochrome P450s (CYP450s), 15 flavin-containing monooxygenases (FMOs), 53 glutathione S-transferases (GSTs) and 105 ATP binding cassette (ABC) transporters, most of which showed up-regulated expression under stress (Table S3 and Table S4). These proteins have been shown to function in contaminant detection, conjugative modification and expulsion for xenobiotic detoxification [55-57].

The massive production of reactive oxygen species (ROS) and reactive oxygen intermediates (ROIs) induced by stress leads to many pathological conditions, and antioxidant systems protect the organism from superoxide [58]. Four main antioxidant enzyme classes, namely, superoxide dismutase (SOD), catalase (CAT), peroxidase (Prx), and glutathione peroxidase (GPX), were found in *P. canaliculata* and showed elevated global expression in response to stress (Table S3 and Table S4).

Apoptosis is a process of cell death when sensing stress and the regulation of apoptosis maintains the dynamic homeostasis of the internal environment. In *P.*

*canaliculata*, we propose the existence of both intrinsic and extrinsic apoptotic signaling pathways, evidenced by the presence of homologous genes involved in both pathways. These two pathways could be activated by cytochrome C and tumour necrosis factor receptor (TNFR), respectively (Table S3). Inhibitors of apoptosis, such as XIAP, Bcl2 and Bak, are also detected and show increased expression in response to stress (Table S4), which is expected to delay the process of apoptosis and cell death in the stress response.

**The expansion of the P450 gene family contribute to stress tolerance**

Cytochrome P450 (CYP) enzymes are a monooxygenase family with highly diverse structures and functions that have been widely identified in all kingdoms of life [59]. P450s catalyse the reductive scission of molecular oxygen and are responsible for the synthesis and metabolism of various molecules, including drugs, hormones, antibiotics, pesticides, carcinogens and toxins [60]. The hormones they synthesize, such as glucocorticoids, mineralocorticoids, progestins, and sex hormones, are critical to stress response, growth and reproduction, and the endogenous and exogenous chemical metabolism participate in combatting toxic compounds [61].

We found that the *P. canaliculata* CYP gene family had undergone an expansion compared to that in the other molluscs. We identified 157 genes in the genome of *P. canaliculata* and 128, 102, 135, 78, 52 and 94 genes in *A. californica, B. glabrata, C. gigas, L. giganta, O. bimaculoides* and *P. fucata* respectively, using the same standard (Figure 4a). An expansive trend was also observed in comparison with other model

species, such as *Homo sapiens* (57), *Mus musculus* (102), *Danio rerio* (94) and *Drosophila melanogaster* (94) [62]. Gene expansion was mainly found in the CYP2U and CYP3A sub-families, whereas fewer genes were expanded in CYP4F. In mammals, CYP2U participates in the metabolism of fatty acids to generate bioactive eicosanoid derivatives, potentially regulating the development of immune function [63]. In *P. canaliculata*, 40 genes formed the CYP2U clade, mainly expressed in the hepatopancreas (Figure 4b and Table S5_a, Table S5_b). CYP3A is a versatile enzyme that metabolizes a wide range of xenobiotics, and its production promotes the growth of various cell types [64]. The 56 CYP3A genes are comprehensively expressed in the hepatopancreas, gill and kidney (Figure 4b and Table S5_a, Table S5_b). CYP4F possesses epoxygenase activity, metabolizing fatty acids to epoxides to suppress hypertension, pain perception and inflammation [65]. Twenty genes were identified in CYP4F, and Pc06G011748, Pc06G011460, Pc06G011458, Pc06G011459, Pc04G006708, Pc04G006710 and Pc04G006707 exhibited highly induced expression levels under cold, heat, heavy metal and air exposure stress, indicating their critical roles in the stress tolerance (Figure 4b, Table S5_a and Table S5_b).

**The identification of perivitellin genes and their high transcriptional levels in the ovary and albumen gland**

*P. canaliculata* has eggs characterized by abundant nutrients, reddish or pinkish colour, aerial oviposition and neurotoxicity [23, 66] due to the perivitelline Fluid (PVF), which fills the space between the eggshell and the embryo and consists of carbohydrates, lipids and proteins (Figure 5a). The PVF proteins in *P. canaliculata*,

include three major components, PcOvo, PcPV2, and PcPV3 [67], collectively named perivitellins, which make up 90% of the total proteins, whereas most of the other dozens of low-abundance components each account for less than 1% of the total proteins [35]. The perivitellins are not only responsible for the major supply of materials and energy during embryogenesis but also provide warning pigments and deadly toxicants against predators [23, 68, 69].

We identified 28 candidate PVF genes in *P. canaliculata* by mapping each of the 59 fragmental PVF protein sequences derived from a previous proteomics study by Sun [35] to its best hit in the reference gene set of *P. canaliculata*, using BLASTP with requirements of over 85% identity and at least 50% alignment length (Table S6). Then, the functional annotation of those fragmental proteins was also transferred to our identified PVF genes. The transcriptome data show that 22 (79%) of the 28 candidate PVF genes exhibit their highest expression in the ovary and albumen gland (PVF protein synthesis factory) among all 7 tissues (Figure 5b and Table S7), confirming that most of them are genuine functional PVF genes. Six of these 28 candidate PVF genes are perivitellin genes, including two PcOvo genes, Pc09G015543 (PcOvo2) and Pc09G015548 (PcOvo3); two PcPV2 genes, Pc07G012572 (PcPV2-31) and Pc07G012571 (PcPV2-67); and two possible PcPV3 genes, Pc09G015546 and Pc09G015547. The expression levels of these 6 genes in the ovary and albumen gland are much higher than those of the other 22 candidate PVF genes.

By analysing the orthoFinder gene families that include orthologous and paralogous genes from *P. canaliculata* and 8 other sequenced mollusc species, we found that

these 28 candidate PVF genes were classified into 20 multiple-gene families (⩾2 genes) and 7 single-gene families (only one gene) (Table S8). Notably, 5 of the 6 perivitellin genes were classified into single-gene families, except for Pc07G012571 (PcPV2-67), which not only has homologous genes in other mollusc species but also has three paralogous genes in *P. canaliculata* itself. However, none of these three PcPV2-67 paralogous genes in *P. canaliculata* showed higher expression in the ovary and albumen gland than in other tissues, indicating that they are likely not PVF-related genes, i.e., only Pc07G012571 plays a role in PVF. The nearly unique and single-copy nature of the 6 perivitellin genes in *P. canaliculata*, may be explained by the long evolutionary distance, over 200 Mya for *P. canaliculata* and its most closely related species, *A. californica*, as well as numerous differences in their living characteristics and egg structures. Another possible explanation is that these 6 major PVF genes may have experienced rapid evolution in their history to adapt to the changing environment.

**The gut microbiome plays important roles in stress resistance and food digestion**

The gut microbiome is well known as the second genome of animals and plays important roles in food digestion, immune defence, and other processes that are essential to the animal host. To investigate whether the gut microbiome influences the invasive lifestyle, we collected gut digesta samples from 70 *P. canaliculata* snails and generated 31 Gb of high-quality metagenomic data on the Illumina HiseqX10 platform. To our knowledge, this study is the first in-depth sequencing of the snail gut

microbiome. A total of 1,142,095 non-redundant genes were obtained with an average open reading frame (ORF) length of 604 bp (Table S9). The taxonomic composition analysis showed that, at the phylum level, Proteobacteria was predominant, followed by Verrucomicrobia, Bacteroidetes, Firmicutes, Spirochaetes, Actinobacteria, etc. (Table S10_a). At the genus level, the most abundant genera included *Aeromonas*, *Enterobacter*, *Desulfovibrio*, *Citrobacter*, *Comamonas*, *Klebsiella* and *Pseudomonas* (Table S10_b), most of which were also present in *Achatina fulica* [70,71].

Interestingly, some of the most abundant genera, such as *Desulfovibrio, Citrobacter* and *Pseudomonas*, were reported as having strong abilities to remove heavy metals by bioprecipitation and bioabsorption [72-74]. For example, the sulfur-reducing bacteria *Desulfovibrio* produces $H_2S$, which precipitates metals and therefore reduces the toxic effects of dissolved metals [72]. Based on the KEGG pathway database, the complete sulfate reduction metabolism pathway was identified in the *P. canaliculata* gut microbiome. We suggested that these gut microbes might help *P. canaliculata* survive the environmental stress of heavy metals in harsh conditions. In addition, a large number of genes in xenobiotic biodegradation and metabolism pathways were annotated, corresponding to 288 KEGG orthologous groups (KOs) and 21 pathways (Table S11). As many of the pathways, such as benzoate degradation, toluene degradation, xylene degradation and steroid degradation, could not be identified in the host genome through KO analysis, we suggested that microbial detoxification abilities may contribute to the ability *P. canaliculata* to resist stresses caused by xenobiotics such as pesticides and environmental pollutants.

In digestion, the gut microbes are directly involved in the breakdown of the cellulose portion of the diet, and previous studies have isolated cellulolytic bacteria and evaluated the cellulolytic enzyme activities [75]. Our work found a broader range of carbohydrate active enzymes (CAZymes). Of the 208 annotated CAZyme families, 99 were glycoside hydrolase (GH) families (Table S12). Enzymes that could be classified as cellulases, endohemicelluloses, debranching enzymes, and oligosaccharide-degrading enzymes were all identified. These findings indicate that the gut microbiome provides assistance in digesting a broad range of food sources, enabling *P. canaliculata* to grow rapidly and adapt to an invasive lifestyle.

## Conclusion and discussion

Given its environmental invasiveness, broad stress adaptability and rapid reproduction, the golden apple snail *P. canaliculata* has received a vast amount of attention worldwide. However, the underlying genetic mechanisms of these properties have not been comprehensively uncovered. The chromosome-level genome of *P. canaliculata* presented in this study sheds the first light on into the genomic basis of its ecological plasticity in response to various stressors. The major findings of this study include the recent explosion of DNA/hAT-Charlie TEs, the expansion of the P450 gene family and the constitution of the cellular homeostasis system, all of which contribute to the plasticity of the organism in stress adaptation. Although the function of the recently originated TEs could not be confirmed, TEs are considered powerful facilitators in adaptive evolution, suggesting that their increased number plays an important role in

the stress resistance of *P. canaliculata*. The UPR system, xenobiotic biotransformation system and ROS system are all major components of the cellular homeostasis system, and the P450s in particular underwent expansion with specific functions. In addition, exclusive perivitellin genes were identified in the *P. canaliculata* genome, and they are believed to contribute to the high reproductive rate and the expansion of habitats. Furthermore, the gut metagenome contains diverse genes for food digestion and xenobiotic degradation. These findings collectively provide novel insight into the molecular mechanisms of ecological plasticity and high invasiveness.

In this study, we report a fine reference genome of *P. canaliculata*, first chromosome-level Mollusca genome published. With its easy acquisition, rapid growth and efficient reproduction, *P. canaliculata* possesses the potential to be a model organism of Mollusca. As its cellular complexity and conservation of pathways also make *P. canaliculata* a useful representative of Mollusca, the genome described in this study can be used to advance our understanding of the molecular mechanisms involved in various scientific questions regarding Mollusca.

## Methods

### Samples collection and sequencing

Adults of *P. canaliculata* were collected from a local paddy field in Shenzhen, Guangdong province, China, and maintained in aerated freshwater at 15 ± 2 °C for a week before processing. Genomic DNA was extracted from the foot muscles of a single *P. canaliculata* for constructing PCR free Illumina 350-bp insert libraries and

PacBio 20-kb insert library, and sequenced on Illumina HiSeq 2500 and PacBio SMRT platforms, respectively. The Hi-C library was prepared using the muscle tissue of another single *P. canaliculata* by following methods: Nuclear DNA was cross-linked in situ, extracted, and then digested with a restriction enzyme. The sticky ends of the digested fragments were biotinylated, diluted, and then ligated to each other randomly. Biotinylated DNA fragments were enriched and sheared again for preparing the sequencing library, which was then sequenced on a HiSeq X Ten platform (Illumina).

Seven tissues including embryos (2 days post fertilization), gill, hemocytes, hepatopancreas, kidney, ovary and albumen gland and testis from six animals were collected as parallel samples. Next, animals were cultivated in 37 °C and 10 °C for 24 hours heat and cold tolerance, in $Cr^{3+}$(2mg $L^{-1}$), $Cu^{2+}$(0.2mg $L^{-1}$) and $Pb^{2+}$(1mg $L^{-1}$) for 24 hours heavy metal tolerance, and in waterless tank for 7 days air exposure. Then the hemocytes were harvested and stored, with three replicates for each group. In final, total RNAs were extracted from the stored tissues of *P. canaliculata* materials, and then mRNAs were pulled out by beads with poly-T for constructing cDNA libraries (insert 350-bp), and sequenced on an Illumina HiSeq 2500 sequencer.

The intestinal digesta from 70 adult snails of *P. canaliculata* were collected, pooled into 6 samples and stored at −20 °C until microbial DNA was extracted. A combination of cell lysis treatments was applied, including five freeze-thaw cycles (alternating between 65 °C and liquid nitrogen for 5 min), repeated beads-beating in ASL buffer (cat. no. 19082; Qiagen Inc.), and incubated at 95 °C for 15 min. DNA

was isolated following the protocol reported protocol [76]. Paired-end libraries of metagenomic DNA were prepared with an insert size of 350 base pairs (bp) following the manufacture’s protocol (cat. no. E7645L; New England Biolabs). Sequencing was performed on Illumina HiSeq X10.

**Genome assembly and annotation**

The Illumina raw reads were filtered by trimming the adapter sequence and low-quality regions (https://github.com/fanagislab/common_use), resulting in clean and high-quality reads with an average error rate < 0.001. For the PacBio raw data, the short subreads (< 2 kb) and low-quality (error rate > 0.2) subreads were filtered out, and only one representative subread was retained for each PacBio read. The clean PacBio reads were assembled by the software smartdenovo (https://github.com/ruanjue/smartdenovo), after which Illumina reads were aligned to the contigs by BWA-MEM, and single base errors in the contigs were corrected by Pilon (v1.16) with the parameters “-fix bases, -nonpf, -minqual 20”. The *P. canaliculata* genome is highly heterozygous, as illustrated by the double peaks on the distribution curve of k-mer frequency, and the current assembly algorithm tends to collapse homozygous regions and report heterozygous regions in alternative contigs. To obtain a haploid reference contigs, we employed a whole-genome alignment (WGA) strategy with MUMmer v3.23 to recognize and selectively remove alternative heterozygous contigs, which were characterized by shorter length (less than 200 kb) and the ability of most regions (more than 50%) to be aligned to another larger contig

with confident identity (higher than 80%). Next, Hi-C sequencing data were aligned to the haploid reference contigs by BWA-MEM, and then these contigs were clustered into chromosomes with LACH-ESIS (http://shendurelab.github.io/LACHESIS/).

A de novo repeat library for *P. canaliculata* was constructed by RepeatModeler (v1.0.4; http://www.repeatmasker.org/RepeatModeler.html). TEs in the *P. canaliculata* genome were also identified by RepeatMasker (v4.0.6; http://www.repeatmasker.org/) using both the Repbase library and the de novo library. Tandem repeats in the *P. canaliculata* genome were predicted using Tandem Repeats Finder v4.07b [77]. The divergence rates of TEs were calculated between the identified TE elements in the genome and their consensus sequence at the TE family level.

The gene models in the *P. canaliculata* genome were predicted by EVidence Modeler v1.1.1 [78], integrating evidence from ab initio predictions, homology-based searches and RNA-seq alignments. Then, these gene models were annotated by RNA-seq data, UniProt database and InterProScan software [79]. Finally, the gene models were retained if they had at least one piece of supporting evidence from the UniProt database, InterProScan domain and RNA-seq data. Gene functional annotation was performed by aligning the protein sequences to the NCBI NR, UniProt, COG and KEGG databases with BLASTP v2.3.0+ under an E-value cutoff of $10^{-5}$ and choosing the best hit. Pathway analysis and functional classification were conducted based on the KEGG database [80]. InterProScan was used to assign preliminary GO terms, Pfam domains and IPR domains to the gene models.

## Evolutionary analysis

Orthologous and paralogous groups were assigned from seven species (*P. canaliculata, Lottia giganta, Aplysia californica, Biomphalaria glabrata, Crassostrea gigas, Octopus bimaculoides, Pintada fucata, Limnoperna fortunei* and *Lingula anatina*) by OrthoFinder [46] with default parameters. Orthologous groups that contained only one gene for each species were selected to construct the phylogenetic tree. The protein sequences of each gene family were independently aligned by muscle v3.8.31 [81] and then concatenated into one super-sequence. The phylogenetic tree was constructed by maximum likelihood (ML) using PhyML v3.0 [44] with the best-fit model (LG+I+G) estimated by ProtTest3 [82]. The Bayesian relaxed molecular clock (BRMC) approach was adopted to estimate the neutral evolutionary rate and species divergence time using the program MCMCTree, implemented in the PAML v4.9 package [45]. The tree was calibrated with the following time frames to constrain the age of the nodes between the species: minimum = 260 Ma and maximum = 290 Ma for *P. fucata* and *C. gigas* [83]; minimum = 450 Ma and maximum = 480 Ma for *A. californica* (or *B. glabrata*) and *L. giganta* [84]. The calibration time (fossil record time) interval (550-610 Mya) of *O. bimaculoides* was adopted from previous results [85].

## Transcriptome data analysis

Transcriptome reads were trimmed with the same method for genomic reads (https://github.com/fanagislab/common_use), and then mapped to the reference

genome of *P. canaliculata* using TopHat (v. 2.1.0) with default settings. The expression level of each reference gene in terms of FPKM was computed by cufflinks v2.2.1. A gene was considered to be expressed if its FPKM >0. Differential gene expression analysis was conducted using cuffdiff v2.2.1.

**Metagenome data analysis**

Raw reads were cleaned to exclude adapter sequences, low-quality sequences, and contaminated DNA. The adapter sequence was identified and trimmed from the reads by an ungapped dynamic programming algorithm; the low-quality part (head or tail) of the reads was trimmed off to ensure that the average error rate of the remaining reads was lower than 0.001; the reads that were mapped to contaminated DNA by BWA-MEM [86] were filtered out; and finally, shorter reads (length < 75 bp) and unpaired reads were excluded to form a set of clean reads. The BWA database built for cleaning contamination included genomes of 10 species: the *P. canaliculata* genome, the *Brassica rapa* genome, the *Oryza sativa* genome, 2 *Angiostrongylus cantonensis* genomes, the *Caenorhabditis elegans* genome, the *Schistosoma mansoni* genome, the *Clonorchis sinensis* genome, the *Fasciola hepatica* genome, the *Danio rerio* genome, and the *human hg38* genome.

The clean reads were assembled by metaSPAdes (v3.11.1) [87] in paired-end mode for each sample. Then, gene prediction was performed on contigs longer than 500 bp by Prodigal (v2.6.3) [88] with the parameter "-p meta", and gene models with cds length less than 102 bp were filtered out. A non-redundant (NR) gene set (539,344

genes) was constructed using the gene models predicted from each sample by cd-hit-est (v4.6.6) [89] with the parameter "-c 0.95 -n 10 -G 0 –a S 0.9", which adopts a greedy incremental clustering algorithm and the criteria of identity > 95% and overlap > 90% of the shorter genes. Then, the clean reads were mapped onto this NR gene set by BWA-MEM with the criteria of alignment length ⩾ 50bp and identity > 95%. The unmapped reads from all samples were assembled together, and the genes were predicted again. The newly predicted genes were combined with the previous gene set by cd-hit-est to obtain a new NR gene set (1,147,339 genes). After the taxonomic assignments to the new NR gene set, 5244 genes classified as Eukaryota but not fungi were removed, and the final NR gene set (1,142,095 genes) was obtained.

The taxonomic assignments of the final NR genes were made on the basis of DIAMOND [90] protein alignment against the NCBI -NR database by CARMA3 [91]. Functional annotation was performed by aligning all the protein sequences to the KEGG [92] database (release 79) using DIAMOND and taking the best hit with the criteria of E-value < 1e-5. CAZymes were annotated with dbCAN (release 5.0) [93] using HMMER (v3.0) hmmscan [94] by taking the best hit with an E-value < 1e-18 and coverage > 0.35.

The clean reads from each sample were aligned against the gene catalogue (1,142,095 genes) by BWA-MEM with the criteria of alignment length ⩾ 50bp and identity > 95%. Sequence-based gene abundance profiling was performed as previously described [95]. The taxonomic profiles of the samples were calculated by summing

the gene abundance according to the taxonomic assignment result.

## Abbreviations

*A. californica, Aplysia californica; B. glabrata, Biomphalaria glabrata; C. gigas, Crassostrea gigas; O. bimaculoides, Octopus bimaculoides; L. anatina, Lingula anatina; L. fortune, Limnoperna fortune; L. giganta, Lottia giganta; P. canaliculata, Pomacea canaliculata; P. fucata, Pinctada fucata;* Hem, hemocyte; Te, testis; Ov, ovary and albumen gland; Kn, kidney; GI, gill; Hp, hepatopancreas, Em, embryo; SSR, simple sequence repeats; mya, million years ago*; BLAST, basic local alignment search tool;* SNP, single nucleotide polymorphism; PVF, Pervitelline Fluid; Ovo, ovorubin; AFLP, amplified fragment length polymorphism; DEGs, differentially expressed genes; LPyS, Lipopolysaccharide; iTRAQ, Isobaric Tags For Relative, Absolute Quantitation; LC-MS/MS, Liquid Chromatography-tandem Mass Spectrometry; TEs, transposable elements; LTR, long terminal repeats; LINE, long interspersed elements; SINE, short interspersed elements; UPR, Unfolded protein response; HSPs, heat shock proteins; HSF1, heat shock transcription factor 1; PERK, protein kinase RNA-like ER kinase; ATF6,activating transcription factor 6; ER, endoplasmic reticulum; CYP450s, cytochrome P450s; FMOs, flavin-containing monooxygenases; GSTs, glutathione S-transferases; ABC, ATP binding cassette; ROS, reactive oxygen species; ROI, reactive oxygen intermediates; SOD, superoxide dismutase; CAT, catalase; Prx, peroxidase; GPX, glutathione peroxidase; TNFR, tumor necrosis factor receptor; NR, non-redundant genes; ORF, open reading frame;

Kos, orthologous groups; CAZymes, carbohydrate active enzymes; GH, Glycoside Hydrolase.

## Availability of data and materials

Tables S1 to S12 and Figures S1 to S6 are available in the supplementary information file. The raw sequencing data has been deposited in DDBJ/EMBL/GenBank under project accession PRJNA427478, SRR6425828 for genomic Illumina_PE125 sequencing data, SRR6425829 for genomic Illumina_PE150 sequencing data, SRR6425827 for genomic PacBio sequencing data, SRR6429132~SRR6429164 for transcriptome sequencing data, and SRR6472920~SRR6472925 for gut microbiome data. All the analysis data have also been released for public use and can be freely accessed at AGIS ftpsite: ftp://ftp.agis.org.cn/~fanwei/Pomacea_canaliculata_Genome/ .

## Authors' contributions

WF and WQ conceived the study and designed the experiments. CL and YZ performed the genome sequencing and assembly, BL performed annotation and evolutionary analysis. CL performed the stress tolerance analysis, YR performed the reproduction analysis, YZ performed the metagenome analysis. HW, SL, FJ, LY provide suggestions and help checking. WF, CL, BL, YR, YZ wrote the manuscript, and GZ help revise the manuscript. All authors read and approved the final manuscript.

## Competing interests

The authors declare that they have no competing interests.

## Acknowledgements

This project is supported by the National key research and development program of China (2016YFC1200600), Shenzhen science and technology program (JCYJ20150630165133395), Fund of Key Laboratory of Shenzhen (ZDSYS20141118170111640), and The Agricultural Science and Technology Innovation Program (ASTIP) of Chinese Academy of Agricultural Sciences(CAAS) & Elite Youth Program of Chinese Academy of Agricultural Sciences. We thank Fanghao Wan, Jue Ruan, Yutao Xiao for providing constructive suggestions to this project.

# Legends of tables and figures

## Tables

**Table 1. Summary of assembly and annotation of mollusc genomes**

| **Genome feature** | ***P. canaliculata*** | ***L. giganta*** | ***A. californica*** | ***B. glabrata*** | ***C. gigas*** | ***O. bimaculoides*** |
|---|---|---|---|---|---|---|
| Assembled sequences (bp) | 440,071,717 | 359,505,668 | 927,310,431 | 916,377,450 | 557,735,934 | 23,381,887,882 |
| Contig N50 size (bp) | 1,072,857 | 94,165 | 9,817 | 18,978 | 37,218 | 5,982 |
| Contig N90 size (bp) | 303,904 | 10,180 | 1,626 | 5,132 | 11,109 | 1,606 |
| Scaffold N50 size (bp) | 31,531,291 | 1,870,055 | 917,541 | 48,059 | 401,685 | 475,182 |
| Scaffold N90 size (bp) | 23,662,357 | 74,480 | 207,390 | 817 | 68,181 | 79,088 |
| GC content (%) | 40.3 | 33.3 | 40.3 | 36.0 | 33.4 | 36 |
| No. of gene models | 21,533 | 23,824 | 19,909 | 14,224 | 28,402 | 15,814 |
| Avg. CDS length (bp) | 1,497 | 1,136 | 1,568 | 1,066 | 1,472 | 1,535 |

| | | | | | | |
|---|---|---|---|---|---|---|
| BUSCO (%) | 98.9 | 98.4 | 98.7 | 72.8 | 99.4 | 98.7 |
| Transposable elements (bp) | 49,579,006 | 37,369,817 | 202,174,499 | 189,550,886 | 103,381,274 | 737,398,096 |
| Tandem repeat (bp) | 873,801 | 257,674 | 8,263,822 | 2,145,821 | 590,907 | 62,633,792 |

## Figures

**Figure 1. The genome characteristics of *P. canaliculata*. (a)** Circos plot showing the genomic features**.** Track 1: 14 linkage groups of the genome; Track 2: distribution of transposon elements in chromosomes; Track 3: protein-coding genes located on chromosomes; Track 4: distribution of GC contents. **(b)** A genome-wide contact matrix from Hi-C data between each pair of the 14 chromosomes using a 100 kb window size. The colour value indicates the base 2 logarithm of the number of valid reads ($\log_2$(valid reads)). **(c)** Distribution of CDS length in six closely related species.

**Figure 2. Evolutionary genomic analysis of *P. canaliculata*. (a)** Phylogenetic placement of *P. canaliculata* within the dated tree of molluscs. The estimated divergence time is shown at each branching point, and *P. canaliculata* is shown in red. **(b)** Distribution of divergence rate for the class of DNA transposons in molluscs genomes. The divergence rate was calculated by comparing all TE sequences identified in the genome to the corresponding consensus sequence in each TE subfamily. The red arrow indicates that *P. canaliculata* and *C. gigas* had a recent explosion of TEs at a divergence rate of ~4%.

**Figure 3. The cellular homeostasis system in *P. canaliculata***. The unfolded protein response (UPR) system includes HSPs and HSF in the heat shock response and CNX, NEF, GRP94, BIP, HSP40, ATF6, IRE1, PERK, COP2, XBP, ATF4, TRAM and Derlin in the endoplasmic reticulum unfolded protein response (UPR-ERAD). Apoptotic pathways include XIAPs, Bcl2, caspases, TNFR, and FADD. The antioxidant systems include PRX, SOD, CAT and GPX. The xenobiotic biotransformation system includes EPHX3, P450, FMO and ABC transporter. The colours of the boxes for gene families represent the degree of upregulation

(FPKM-stimulus/FPKM-control) as an overall result of stress, including heat, cold, heavy metal and air exposure. Pathways and genes were obtained based on KEGG annotation.

**Figure 4. The expansion of the P450 gene family in *P. canaliculata*.** (a) Phylogenetic tree demonstrating orthologous and paralogous relationships of all P450 genes from 7 species including *P. canaliculata*, *A. californica*, *B. glabrata*, *C. gigas*, *L. giganta*, *O. bimaculoides* and *P. fucata*. P450 genes from seven species were obtained based on Pfam annotation (Interpro) with an E-value of $10^{-5}$. Clades are labelled by P450 subfamily names. The tree was constructed using the maximum likelihood method in MEGA7, and the branch length scale indicates the average number of residue substitutions per site. (b) Phylogenetic tree of P450 genes in *P. canaliculata*, which is a subset of the phylogenetic tree for the 7 species, and their heat map of expression (FPKM) in seven tissues (Hem, hemocyte; Te, testis; Ov, ovary and albumen gland; Kn, kidney; Gl, gill; Hp, hepatopancreas; Em, embryo) and heat map of induced expression (FPKM-stimulus/FPKM-control) under stress (Con: control; heat; cold; Hm: heavy metal; Exp: air exposure).

**Figure 5. The composition and expression of the *P. canaliculata* perivitellins in different tissues.** (a) Perivitelline fluid (PVF) lies under the eggshell and surrounds the embryo. It contains carbohydrates, lipids, and proteins. The proteins are also known as perivitellins and are classified into three categories, PcOvo, PcPV2, and PcPV3. (b) The displayed expression value of PVF proteins is the base 10 logarithm of FPKM ($log_{10}$FPKM). The genes marked in red encode perivitellins. The 7 tissues

examined are abbreviated as follows: Hem, hemocyte; Te, testis; Ov, ovary and albumen gland; Kn, kidney; Gl, gill; Hp, hepatopancreas; Em, embryo.

## References


1. Lowe S, Browne M, Boudjelas S, de Poorter M. 100 of the World's Worst Invasive Alien Species: A selection fromthe Global Invasive Species Database. Auckland, New Zeland: World Conservation Union (IUCN); 2000.
2. Ranamukhaarachchi SL, Wickramasinghe S. Golden apple snails in the world: introduction, impact, and control measures. Global advances in ecology and management of golden apple snails. 2006:133-52.
3. Naylor R. Invasions in Agriculture: Assessing the Cost of the Golden Apple Snail in Asia. Royal Swedish Academy of Sciences. 1996;25:443-8.
4. Berthold T. Vergleichende Anatomie, Phylogenie und historische Biogeographie der Ampullariidae: (Mollusca, Gastropoda). 1991.
5. Howells RG, Burlakova LE, Karatayev AY, Marfurt RK, Burks RL. Native and introduced Ampullaridae in North America: History, status, and ecology. 2006:73-112.
6. Halwart M, Bartley DM. International mechanisms for the control and responsible use of alien species in aquatic ecosystems, with special reference to the golden apple snail. Los Baños, Philippines: Philippine Rice Research Institute (PhilRice); 2006.
7. López MA, Altaba CR, Andree KB, López V. First invasion of the Apple snail Pomacea insularum in Europe. Tentacle. 2010;18:26-8.
8. Estebenet AL, Martín PR. Pomacea canaliculata (Gastropoda: Ampullariidae): life-history

traits and their plasticity. Biocell 2002;26:83-9.

9. Lach L. The spread of the introduced freshwater apple snail *Pomacea canaliculata* (Lamarck) (Gastropoda Ampullariidae) on Oahu, Hawaii. Bishop Museum Occasional Papers. 1999;58:66-71.

10. Yusa Y，Sugiura N, Wada T. Predatory Potential of Freshwater Animals on an Invasive Agricultural Pest, the Apple Snail *Pomacea canaliculata* (Gastropoda: Ampullariidae), in Southern Japan. Biol Invasions. 2006;8:137-47.

11. Lach L, Britton DK, Rundell RJ, Cowie RH. Food Preference and Reproductive Plasticity in an Invasive Freshwater Snail. Biol Invasions. 2000;2:279-88.

12. Mochida O. Spread of freshwater *Pomacea* snails (Pilidae, Mollusca) from Argentina to Asia. Micronesica. 1991;3 51-62.

13. Shan L, Zhang Y, Steinmann P, Zhou X. Emerging Angiostrongyliasis in Mainland China. Emerg Infect Dis. 2008;14:161-4.

14. Caldeira RL, Mendonca CL, Goveia CO, Lenzi HL, Graeff-TeixeiraC Lima WS, et al. First record of molluscs naturally infected with *Angiostrongylus cantonensis* (Chen, 1935) (Nematoda: Metastrongylidae) in Brazil. Memórias do Instituto Oswaldo Cruz. 2007;102:887-9.

15. McMichael AJ, Beaglehole R. The changing global context of public health. Lancet (London, England). 2000;356:495-9.

16. Chapman A. Numbers of Living Species in Australia and the World. Australian Biological Resources Study; 2009.

17. Lindberg DR, Ponder WF, Haszprunar G. The Mollusca: relationships and patterns from

their first half-billion years. Oxford University Press, Oxford; 2004.

18. Hayes KA, Cowie RH, Thiengo SC. A global phylogeny of apple snails: Gondwanan origin, generic relationships, and the influence of outgroup choice (Caenogastropoda: Ampullariidae). Biol J Linn Soc Lond. 2009;98:61-76.

19. Matsukura K, Tsumuki H,Izumi Y, Wada T. Physiological response to low temperature in the freshwater apple snail, *Pomacea canaliculata* (Gastropoda: Ampullariidae). J Exp Biol. 2009;212:2558-63.

20. Yusa Y, Wada T, Takahashi S. Effects of dormant duration, body size, self-burial and water condition on the long-term survival of the apple snail, *Pomacea canaliculata* (Gastropoda: Ampullariidae). Appl Entomol Zool. 2006;41:627-32.

21. Seuffert ME, Burela S, Martín PR. Influence of water temperature on the activity of the freshwater snail Pomacea canaliculata (Caenogastropoda: Ampullariidae) at its southernmost limit (Southern Pampas, Argentina). Journal of Thermal Biology. 2010; 35:77-84.

22. Kruatrachue M, Sumritdee C, Pokethitiyook P, Singhakaew S. Histopathological effects of contaminated sediments on golden apple snail (*Pomacea canaliculata*, Lamarck 1822). Bull Environ Contam Toxicol. 2011;86:610-4.

23. Dreon MS, Frassa MV, Ceolín M, Ituarte S, Qiu JW, Sun J, et al. Novel animal defenses against predation: a snail egg neurotoxin combining lectin and pore-forming chains that resembles plant defense and bacteria attack toxins. PLoS One. 2013;8:e63782. doi:10.1371/journal.pone.0063782.

24. Ottaviani E, Caselgrandi E, Fontanili P, Franceschi C. Evolution, immune responses and

stress: studies on molluscan cells. Acta Biol Hung. 1992;43:293-8.

25. Ottaviani E, Accorsi A, Rigillo G, Malagoli D, Blom JM, Tascedda F. Epigenetic modification in neurons of the mollusc *Pomacea canaliculata* after immune challenge. Brain Res. 2013;1537:18-26.

26. Mercado Laczkó AC, Lopretto EC. Estudio cromosómico y cariotípico de p*omacea canaliculata* (Lamarck, 1801) (Gastropoda, Ampullariidae). Revista del Museo Argentino de Ciencias Naturales "Bernardino Rivadavia" Hidrobiología. 1998;8:15-20.

27. Xu J, Han X, Li N, Yu J, Qian C, Bao Z. Analysis of genetic diversity of three geographic populations of *Pomacea canaliculata* by AFLP. Acta Ecol Sin. 2009;29:4119- 26.

28. Chen L, Xu H, Li H, Wu J, Ding H, Liu Y. Isolation and characterization of sixteen polymorphic microsatellite loci in the golden apple snail *Pomacea canaliculata*. Int J Mol Sci. 2011;12:5993-8.

29. Mu X, Hou G, Song H, Xu P, Luo D, Gu D, et al. Transcriptome analysis between invasive *Pomacea canaliculata* and indigenous *Cipangopaludina cahayensis* reveals genomic divergence and diagnostic microsatellite/SSR markers. BMC Genet. 2015;16:12.

30. Sun J, Wang M, Wang H, Zhang H, Zhang X, Thiyagarajan V, et al. De novo assembly of the transcriptome of an invasive snail and its multiple ecological applications. Mol Ecol Resour. 2012;12:1133-44.

31. Mu H, Sun J , Fang L, Luan T, Williams GA, Cheung SG, et al. Genetic Basis of Differential Heat Resistance between Two Species of Congeneric Freshwater Snails: Insights from Quantitative Proteomics and Base Substitution Rate Analysis. J Proteome Res. 2015;14:4296-308.

32. Yang L, Cheng TY, Zhao FY. Comparative profiling of hepatopancreas transcriptomes in satiated and starving *Pomacea canaliculata*. BMC Genet. 2017;18:18.

33. Xiong YM, Yan ZH, Zhang JE, Li HY. Analysis of albumen gland proteins suggests survival strategies of developing embryos of *Pomacea canaliculata*. Molluscan Res. 2017:1-6.

34. Sun J, Mu H , Zhang H, Chandramouli KH, Qian PY, Wong CK, et al. Understanding the regulation of estivation in a freshwater snail through iTRAQ-based comparative proteomics. J Proteome res. 2013;12:5271-80.

35. Sun J, Zhang H, Wang H, Heras H, Dreon MS, Ituarte S, et al. First proteome of the egg perivitelline fluid of a freshwater gastropod with aerial oviposition. J Proteome Res. 2012;11:4240-8.

36. Aplysia Genome Project. Broad Institute. Vertebrate Biology Group. 2009. https://www.broadinstitute.org/aplysia/aplysia-genome-project

37. Zhang G, Fang X, Guo X, Li L, Luo R, Xu F, et al. The oyster genome reveals stress adaptation and complexity of shell formation. Nature. 2012;490:49-54.

38. Du X, Fan G, Jiao Y, Zhang H, Guo X, Huang R, et al. The pearl oyster Pinctada fucata martensii genome and multi-omic analyses provide insights into biomineralization. Gigascience. 2017;6:1-12.

39. Simakov O, Marletaz F, Cho SJ, Edsinger-Gonzales E, Havlak P, Hellsten U, et al. Insights into bilaterian evolution from three spiralian genomes. Nature. 2013;493:526-31.

40. Albertin CB, Simakov O, Mitros T, Wang ZY, Pungor JR, Edsinger-Gonzales E, et al. The octopus genome and the evolution of cephalopod neural and morphological novelties.

Nature. 2015;524:220-4.

41. Uliano-Silva M, Dondero F, Dan Otto T, Costa I, Lima NCB, Americo JA, et al. A hybrid-hierarchical genome assembly strategy to sequence the invasive golden mussel Limnoperna fortunei. Gigascience. 2017. doi: 10.1093/gigascience/gix128

42. Adema CM, Hillier LW, Jones CS, Loker ES, Knight M, Minx P, et al. Corrigendum: Whole genome analysis of a schistosomiasis-transmitting freshwater snail. Nat Commun. 2017;8:16153.

43. Liu B, Shi Y, Yuan J, Hu X, Zhang H, Li N, et al. Estimation of genomic characteristics by analyzing k-mer frequency in de novo genome projects. Quantitative Biology 2013:arXiv:1308.2012 [q-bio.GN].

44. Guindon S, Dufayard JF, Lefort V, Anisimova M, Hordijk W, Gascuel O. New algorithms and methods to estimate maximum-likelihood phylogenies: assessing the performance of PhyML 3.0. Syst Biol 2010;59:307-21. doi:10.1093/sysbio/syq010.

45. Yang Z. PAML 4: phylogenetic analysis by maximum likelihood. Mol Biol Evol. 2007;24:1586-91. doi:10.1093/molbev/msm088.

46. Emms DM, Kelly S. OrthoFinder: solving fundamental biases in whole genome comparisons dramatically improves orthogroup inference accuracy. Genome Biol. 2015;16:157. doi:10.1186/s13059-015-0721-2.

47. Feschotte C, Wessler SR. Mariner-like transposases are widespread and diverse in flowering plants. Proc Natl Acad Sci U S A 2002;99:280-5.

48. Hua-Van A, Le Rouzic A, Boutin TS, Filée J, Capy P. The struggle for life of the genome's selfish architects. Biol Direct. 2011;6:19.

49. Werren JH. Selfish genetic elements, genetic conflict, and evolutionary innovation. Proc Natl Acad Sci U S A. 2011;108:10863-70.

50. Chrousos GP. Stress and disorders of the stress system. Nat Rev Endocrinol. 2009;5:374-81.

51. Vabulas RM, Raychaudhuri S, Hayer-Hartl M. Protein folding in the cytoplasm and the heat shock response. Cold Spring Harbor perspectives in biology. 2010;2:a004390.

52. Chen B，Retzlaff M，Roos T，Frydman J. Cellular Strategies of Protein Quality Control. Cold Spring Harbor Perspectives in Biology. 2011;3:a004374.

53. Korennykh A and Walter P. Structural basis of the unfolded protein response. Annu Rev Cell Dev Biol. 2012;28:251-77.

54. Chambers JE and Yarbrough JD. Xenobiotic biotransformation systems in fishes. Comp Biochem Physiol C. 1976;55:77-84.

55. Mello DF, de Oliveira ES, Vieira RC, Simoes E, Trevisan R, Dafre AL, et al. Cellular and Transcriptional Responses of *Crassostrea gigas* Hemocytes Exposed in Vitro to Brevetoxin (PbTx-2) Mar Drugs. 2012;10: 583-97.

56. Boutet I, Tanguy A, Moraga D. Characterisation and expression of four mRNA sequences encoding glutathione S-transferases pi, mu, omega and sigma classes in the Pacific oyster *Crassostrea gigas* exposed to hydrocarbons and pesticides. Mar Biol 2004;146:53-64.

57. Deeley RG, Westlake C, Cole SP. Transmembrane transport of endo- and xenobiotics by mammalian ATP-binding cassette multidrug resistance proteins. Physiol Rev. 2006;86:849-99.

58. Liu C, Zhang T, Wang L, Wang M, Wang W, Jia Z, et al. The modulation of extracellular

superoxide dismutase in the specifically enhanced cellular immune response against secondary challenge of Vibrio splendidus in Pacific oyster (*Crassostrea gigas*). Dev Comp Immunol. 2016;63:163-70.

59. Lamb DC, Lei L, Warrilow AG, Lepesheva GI, Mullins JG, Waterman MR, et al. The first virally encoded cytochrome p450. J Virol. 2009;83:8266-9.

60. Urlacher VB, Girhard M. Cytochrome P450 monooxygenases: an update on perspectives for synthetic application. Trends Biotechnol. 2012;30:26-36.

61. Sanderson T, van den Berg M. Topic 3.1: Interactions of xenobiotics with the steroid hormone biosynthesis pathway. Pure Appl Chem. 2003;75:1957-71.

62. Goldstone JV, McArthur AG, Kubota A, Zanette J, Parente T, Jönsson ME, et al. Identification and developmental expression of the full complement of Cytochrome P450 genes in Zebrafish. BMC Genomics. 2010;11:643.

63. Chuang SS, Helvig C, Taimi M, Ramshaw HA, Collop AH, Amad M, et al. CYP2U1, a novel human thymus- and brain-specific cytochrome P450, catalyzes omega- and (omega-1)-hydroxylation of fatty acids. J Biol Chem. 2004;279:6305-14.

64. Fleming I. The pharmacology of the cytochrome P450 epoxygenase/soluble epoxide hydrolase axis in the vasculature and cardiovascular disease. Pharmacol Rev. 2014;66:1106-40.

65. Zhang G, Kodani S, Hammock BD. Stabilized epoxygenated fatty acids regulate inflammation, pain, angiogenesis and cancer. Prog Lipid Res. 2014;53:108-23.

66. de Jong-Brink M, Boer HH, Joosse J. Mollusca. In: Adiyodi, K.G., Adiyodi, R.G. (Eds.), Reproductive Biology of invertebrates. Oogenesis oviposition and

oosorption, vol. 1. John Wiley & Sons Ltd., New York, 1983; pp. 297-355.

67. Garin CF, Heras H, Pollero RJ. Lipoproteins of the egg perivitelline fluid of *Pomacea canaliculata* snails (Mollusca: Gastropoda). J Exp Zool. 1996;276:307-14.

68. Dreon MS, Schinella G, Heras H, Pollero RJ. Antioxidant defense system in the apple snail eggs, the role of ovorubin. Arch Biochem Biophys. 2004;422:1-8.

69. Dreon MS, Ituarte S, Heras H. The role of the proteinase inhibitor ovorubin in apple snail eggs resembles plant embryo defense against predation. PLoS One. 2010;5:e15059. doi:10.1371/journal.pone.0015059.

70. Cardoso AM, Cavalcante JJV, Vieira RP, Lima JL, Grieco MAB, Clementino MM, et al. Gut Bacterial Communities in the Giant Land Snail *Achatina fulica* and Their Modification by Sugarcane-Based Diet. Plos One. 2012;7 doi:ARTN e3344010.1371/journal.pone.0033440.

71. Cardoso AM, Cavalcante JJV, Cantão ME, Thompson CE, Flatschart RB, Glogauer A, et al. Metagenomic Analysis of the Microbiota from the Crop of an Invasive Snail Reveals a Rich Reservoir of Novel Genes. Plos One. 2012;7 doi:ARTN e4850510.1371/journal.pone.0048505.

72. Cabrera G, Pérez R, Gómez JM, Ábalos A, Cantero D. Toxic effects of dissolved heavy metals on Desulfovibrio vulgaris and Desulfovibrio sp strains. J Hazard Mater 2006;135:40-6. doi:10.1016/j.jhazmat.2005.11.058.

73. Finlay JA, Allan VJ, Conner A, Callow ME, Basnakova G, Macaskie LE. Phosphate release and heavy metal accumulation by biofilm-immobilized and chemically-coupled cells of a Citrobacter sp. pre-grown in continuous culture. Biotechnol Bioeng.

1999;63:87-97.

74. Valls M, de Lorenzo V, Gonzalez-Duarte R, Atrian S. Engineering outer-membrane proteins in *Pseudomonas putida* for enhanced heavy-metal bioadsorption. J Inorg Biochem. 2000;79:219-23.

75. Pinheiro GL, Correa RF, Cunha RS, Cardoso AM, Chaia C, Clementino MM, et al. Isolation of aerobic cultivable cellulolytic bacteria from different regions of the gastrointestinal tract of giant land snail *Achatina fulica*. Front Microbiol. 2015;6 doi:Artn 86010.3389/Fmicb.2015.00860.

76. Zoetendal EG, Heilig HG, Klaassens ES, Booijink CC, Kleerebezem M, Smidt H, et al. Isolation of DNA from bacterial samples of the human gastrointestinal tract. Nature protocols 2006, 1(2): 870-873.

77. Benson G. Tandem repeats finder: a program to analyze DNA sequences. Nucleic Acids Res. 1999;27:573-80.

78. Haas BJ, Salzberg SL, Zhu W, Pertea M, Allen JE, Orvis J, et al. Automated eukaryotic gene structure annotation using EVidenceModeler and the Program to Assemble Spliced Alignments. Genome Biol. 2008;9:R7.

79. Quevillon E, Silventoinen V, Pillai S, Harte N, Mulder N, Apweiler R, et al. InterProScan: protein domains identifier. Nucleic Acids Res. 2005;33:W116-20.

80. Kanehisa M, Goto S, Sato Y, Furumichi M, Tanabe M. KEGG for integration and interpretation of large-scale molecular data sets. Nucleic Acids Res. 2012;40:D109-D14.

81. Edgar RC. MUSCLE: multiple sequence alignment with high accuracy and high throughput. Nucleic Acids Res. 2004;32:1792-7.

82. Darriba D, Taboada GL, Doallo R, Posada D. ProtTest 3: fast selection of best-fit models of protein evolution. Bioinformatics. 2011;27:1164-5. doi:10.1093/bioinformatics/btr088.

83. Sun J, Zhang Y, Xu T, Zhang Y, Mu H, Zhang Y, et al. Adaptation to deep-sea chemosynthetic environments as revealed by mussel genomes. Nature Ecology & Evolution. 2017; 1: 121.

84. Benton MJ, Donoghue PCJ, Asher RJ. in The Timetree of Life:Calibrating and Constraining Molecular Clocks (eds Hedges, S. B. & Kumar, S.)35–86 (Oxford Univ. Press, 2009.

85. Zapata F, Wilson NG, Howison M, Andrade SC, Jörger KM, Schrödl M, et al. Phylogenomic analyses of deep gastropod relationships reject Orthogastropoda. Proc Biol Sci. 2014;281(1794):20141739. doi: 10.1098/rspb.2014.1739.

86. Li H and Durbin R. Fast and accurate short read alignment with Burrows-Wheeler transform. Bioinformatics. 2009;25:1754-60.

87. Nurk S, Meleshko D, Korobeynikov A, Pevzner PA. metaSPAdes: a new versatile metagenomic assembler. Genome Res. 2017;27:824-34.

88. Hyatt D, LoCascio PF, Hauser LJ, Uberbacher EC. Gene and translation initiation site prediction in metagenomic sequences. Bioinformatics. 2012;28:2223-30.

89. Fu L, Niu B, Zhu Z, Wu S, Li W. CD-HIT: accelerated for clustering the next-generation sequencing data. Bioinformatics. 2012;28:3150-2.

90. Buchfink B, Chao X, Huson DH. Fast and sensitive protein alignment using DIAMOND. Nat Methods. 2015;12:59-60.

91. Gerlach W and Stoye J. Taxonomic classification of metagenomic shotgun sequences

with CARMA3. Nucleic Acids Res. 2011;39 doi:Artn E9110.1093/Nar/Gkr225.

92. Kanehisa M, Goto S, Kawashima S, Okuno Y, Hattori M. The KEGG resource for deciphering the genome. Nucleic Acids Res. 2004;32:D277-80.

93. Yin Y, Mao X, Yang J, Chen X, Mao F, Xu Y. dbCAN: a web resource for automated carbohydrate-active enzyme annotation. Nucleic Acids Res. 2012;40:W445-51.

94. Eddy SR. Accelerated Profile HMM Searches. Plos Comput Biol. 2011;7 doi:ARTN e100219510.1371/journal.pcbi.1002195.

95. Qin JJ, Li YR, Cai ZM, Li SH, Zhu JF, Zhang F, et al. A metagenome-wide association study of gut microbiota in type 2 diabetes. Nature. 2012;490:55-60.

Manuscript-with-tracks

Click here to download Manuscript PC_manuscript-with-tracks.docx

# The genome of the golden apple snail *Pomacea canaliculata* provides insight into stress tolerance and invasive adaptation

~~Conghui Liu[1]*, Bo Liu[1]*, Yuwei Ren[1]*, Yan Zhang[1]*, Hengchao Wang[1], Shuqu Li[1], Fan Jiang[1], Lijuan Yin[1], Guojie Zhang[2], Wanqiang Qian[1‡] and Wei Fan[1‡]~~

Conghui Liu[1]*, Yan Zhang[1]*, Yuwei Ren[1]*, Hengchao Wang[1], Shuqu Li[1], Fan Jiang[1], Lijuan Yin[1], Guojie Zhang[2], Wanqiang Qian[1]† , Bo Liu[1]†, Wei Fan[1]†

[1]Agricultural Genomic Institute, Chinese Academy of Agricultural Sciences, Shenzhen, Guangdong, 518120, China.

[2]BGI-Shenzhen, Shenzhen, Guangdong, 518083, China

Conghui Liu: rapherlch@163.com; Yan Zhang: milrazhang@163.com; ~~Bo Liu: lb_bobo@aliyun.com;~~ Yuwei Ren: xiaoshudaxia@126.com; ~~Yan Zhang: milrazhang@163.com;~~ Hengchao Wang: wanghengchao000@qq.com; Shuqu Li: lishuqu1234@163.com; Fan Jiang: greatjf@163.com; Lijuan Yin: yinlijuan1005@163.com; Guojie Zhang: guojie.zhang@bio.ku.dk

*These authors contributed equally to this work.

†Correspondence should be addressed to Wanqiang Qian (qianwanqiang@caas.cn), Bo Liu (lb_bobo@aliyun.com) or Wei Fan (fanwei@caas.cn).

## Abstract

**Background:** The golden apple snail (*Pomacea canaliculata*) is a ~~worldwide~~ fresh water snail listed among~~in~~ the top-100 worst invasive species, worldwide and a noted

agricultural and quarantine pest that causes ~~causing huge~~ great economic losses.~~,~~ It is characterized by ~~with~~ fast growth, strong stress tolerance, a high reproduction rate, and adaptation to a broad range of environments.

**Results:** Here, we used long-read sequencing to produce a 440-Mb high-quality chromosome-~~-~~level assembly for the *P. canaliculata* genome. In total, 50 Mb (11.4%) repeat sequences and 21,533 gene models were identified in the genome. The major ~~Major~~ findings of this study include the recent explosion of DNA/hAT-Charlie transposable elements (TEs), the expansion of the P450 gene family and the constitution of the cellular homeostasis system, which contributes~~ing~~ to ~~the~~ ecological plasticity in ~~the~~ stress adaptation. In addition, the ~~perivitellin gene expansion and~~ high transcriptional levels of perivitellin genes in the ovary and albumen gland promote the function of nutrient~~s~~ supply~~ing~~ and ~~defense~~ defence ability in ~~the~~ eggs. Furthermore, the gut metagenome also contains diverse ~~encodes~~ a ~~rich array of~~ genes for food digestion and xenobiotic~~s~~ degradation.

**Conclusions:** These findings collectively provide novel insight into the molecular mechanisms of the ecological plasticity and high invasiveness. ~~Our results not only strengthen the understanding of molluscs genomics and biological invasion, but also benefit preventing the invasion of apple snail and transmission of pathogenetic parasites.~~

**Keywords:** golden apple snail, *Pomacea canaliculata*, genome, adaptive evolution, stress tolerance, P450, reproduction, perivitelline, metagenome

**Background**

The golden apple snail *Pomacea canaliculata* (family Ampullariidae,~~;~~ o~~O~~rder Architaenioglossa) is a fresh water snail listed ~~in~~among the world’s top 100 ~~of the world's~~ worst invasive species [1]~~,~~ and is considered ~~as a noted~~ an agricultural and quarantine pest worldwide [2]. Native to ~~the~~ tropical and subtropical South America~~n~~, ~~the~~ *P. canaliculata* gradually spread to ~~the~~ non-indigenous regions, such as Southeast and East Asia [3], Africa [4], North America [5], Oceania [6] and even Europe [7].~~,~~ ~~and the~~ Its successful biological invasion ~~was due~~ was closely related to its polyphagous feeding habits [8], voracious appetite [9], broad environmental adaptability [10] and rapid growth and high rate of reproduction [11]. In addition to its ~~Besides the~~ ecological impact, ~~the~~ *P. canaliculata* ravaged a wide range of crops, including grains, fruits and vegetables [12], causing severe economic losses each year as a result of yield loss, replanting cost and ~~the funds of~~ expenditures on control (https://www.cabi.org/isc/datasheet/68490). More seriously, *P. canaliculata* has been involved in the transmission of a ~~human~~ fatal human disease, e~~E~~osinophilic meningitis, that first~~ly~~ appeared in East Asia where people ~~take them as food~~ frequently consume these snails [13]. During this pathophoresis, *P. canaliculata* acts as an important intermediate host of the pathogenic parasite *Angiostrongy~~u~~lus cantonensis*, and the range of ~~infectious~~ infected regions is still expanding, creating a ~~causing~~ great challenge in terms of ~~to~~ human health [14, 15].

Molluscs are~~is~~ a highly diverse group, ~~and~~ second only to arthropods in species

number [16], and their high biodiversity makes them ~~molluscs~~ an excellent model to address ~~the~~ issues such as biogeography, adaptability and evolutionary processes [17].~~,~~ T~~and t~~he worldwide invasive species *P. canaliculata* provides valuable potential in these fields [18]. As a primitive circumtropical species, *P. canaliculata* possesses strong ecologicaly~~y~~ plasticity with many advantages ~~to hold advantage on plenty of aspects~~, including low-~~–~~temperature resistance [19]~~,~~ and drought tolerance [20], which has contributed~~s~~ to its competitive success ~~succeed~~ in resource acquisition ~~over the competitive species~~. ~~It was reported~~ *P. canaliculata* has been reported to establish populations ~~could set population in the distribution of~~ at temperatures ranging from 10 °C to 35 °C [19, 21]. Additionally, *P. canaliculata* ~~is~~ tolerates~~tolerant with~~ heavy metal contamination. When living in contaminated water, the~~its~~ gill is enriched with a~~of~~ high concentration of heavy metals and histopathological changes in the digestive tract ~~is~~ are detected;~~,~~ however, an~~with~~ extremely low mortality rate is observed [~~21~~22]. ~~For protection of embryos, t~~The conspicuous colouration and neurotoxic lectin could confer ~~the eggs~~ a survival advantage on the eggs, ~~and~~ defending~~se~~ the embryos against ~~the~~ potential predators [~~22~~23]. Moreover, ~~the~~an immune-neuroendocrine system can also be detected in *P. canaliculata*, as demonstrated~~s~~ by the existence of a specific immune memory after ~~the~~ bacterial challenge [24~~23~~, –25~~24~~], broadening the study~~ies~~ of invertebrate immunology.

The rich phenotypic and genetic diversity of molluscs makes them an excellent species group ~~to~~for addressing many important issues in ~~about~~ evolution, ecology and function. However, the genomic resources on~~f~~ Mollusca ~~is~~are still insufficient

**Formatted:** Not Highlight

**Formatted:** Not Highlight

**Formatted:** Indent: First line: 0"

compared with those of other close phylaums, such as Arthropoda and Nematoda, and few molluscs cancould be employed as model organisms. *P. canaliculata*, however, possesses the potential to be a model organism amongof molluscs because of several inherent characteristicss. For example, *P. canaliculata* is easy to acquire , forbecause it has a broad global distribution originatinged from a primarily circumtropical environment. Due to the Moreover, its high adaptability, rapid growth and efficient reproduction, facilitate the cultivation of *P. canaliculata* also facilitate the cultivation in the laboratory.

**Formatted:** Font: Not Italic

In recentDuring the past years, the genomic features of *P. canaliculata* have been increasingly studied. After the discovery of 14 pachytene bivalents in the karyotype [2526], molecular markers were identified to investigate the genetic diversity of the *P. canaliculata* population, including 369 amplified fragment length polymorphism (AFLP) locis [2627], 16,717 simple sequence repeats (SSR) [27, 2828, 29] and 15,412 single-nucleotide polymorphisms SNPs [2930]. In addition, multiple transcriptome analyses have been performed to investigate the adaptation, invasion and immune mechanisms of *P. canaliculata*. For instance, Sun et al. reported 128,436 unigenes based on a de novo assembly of Illumina reads [2930];, transcriptome changes in response to heat stress and starving incubation werewas used to characterize its invasive and adaptive abilities [30, 3131, 32];, a transcriptome analysis between comparing invasive *P. canaliculata* and indigenous *Cipangopaludina cathayensis* provideds insights into biological invasion [2829];, and 402 immune-related differentially expressed genes (DEGs) in response to by

Llipopolysaccharide (LPyS) challenge were used to explore the mechanisms of defence against pathogens [3332]. Furthermore, proteomics tools such as Iisobaric Tags tags For for Relativerelative, and aAbsolute Quantitationquantitation (iTRAQ), and Liquid liquid Chromatographychromatography-tandem Mass mass Spectrometry spectrometry (LC-MS/MS) were also applied in the study of protein expression during for the estivation and oviposition [34, 3533, 34], together providing plentiful omics- data for the functional analysis of *P. canaliculata*.

However, researches at the whole--genome level in *P. canaliculata* still lags far behind that in other molluscs species, due to the lack of a high-quality reference genome. By far, Mmultiple draft genomes of molluscs have been published, such as including the genomes of the California sea hare [3635], Pacific oyster [3736], Pearl oyster [37] pearl oyster [38], owl limpet [3938], California two-spot octopus [3940], deep-sea golden mussel [4140](), and *Biomphalaria* snails [4142], greatly promoting the research onf molluscs genomics. In this study, we present a chromosome-level genome assembly of *P. canaliculata* with high-quality gene annotation, transcriptome data from several tissues and under various conditions, as well as the and metagenomic data from the intestinal tracts, all of which were then applied to study the species-specific environmental adaptationinvasive characteristicss, such as the cellular homeostasis system underlying strong stress, and the colour and nutrient contents of the eggs. Our data will not only strengthen the understanding of the evolutionary mechanisms of molluscs and the molecular basis of biological invasion, but also foster the developments of approaches to control the invasion of *P.*

**Formatted:** Not Highlight

**Formatted:** Not Highlight

**Formatted:** Not Highlight

**Formatted:** Not Highlight

**Formatted:** Not Highlight

**Formatted:** Not Highlight

**Formatted:** Not Highlight

*canaliculata* and provide a basis for interrupting~~interrupt~~ the transmission of pathogenetic nematode parasites.

## RESULTS

### Complete genome assembly at the chromosome level

We generated 26.6 Gb (60.1 X) of PacBio SMRT raw reads with an average read length of 10.1 ~~Kb~~kb, and 291 Gb (652.4 X) of Illumina HiSeq paired-end reads with an average read length of 150-250 bp~~,~~ using DNA extracted from a~~one~~ single adult *P.* ~~*canaliculate*~~ *canaliculata* (Table S1). The 24.4 Gb (55.4 X) of clean PacBio SMRT reads that passed quality filtering were assembled by smartdenovo (https://github.com/ruanjue/smartdenovo), ~~giving rise to~~ resulting in an assembly of 1,234 raw contigs with a total length of 473.6 Mb and an N50 length of 1.0 Mb. After filtering of alternatively heterozygous contigs, the 745 resulting contigs with a total length of 440.1 Mb and an N50 length of 1.1 Mb were taken as the final contigs. Previous karyotype research has shown that the haploid P. ~~*canaliculate*~~ *canaliculata* genome consists of 14 chromosomes [~~25~~26]. Based on the Hi-C data, 439.5 Mb (99.9%) of final contigs were anchored and oriented into 14 large scaffolds, each corresponding to a natural chromosome (Figure 1a and Figure 1b), with the longest 45.4 Mb and the shortest 27.2 Mb. This assembly quality is much better than that of the other ~~published~~ molluscan genomes published thus~~so~~ far (Table 1). ~~Besides~~ In addition to the length and continuity of the assembled sequences, another important aspect for evaluating genome assembly is the ratio of genome coverage. With an

estimated genome size of 446 Mb and genome heterozygosity between 1% and 2% based on the distribution of k-mer frequency [~~42~~43] (Figure S1), ~98.6 % of the *P. canaliculata* genome has been assembled~~ in *P. canaliculata*~~. To further confirm the accuracy and completeness of the assembly, we mapped the Illumina shotgun reads to the assembled reference genome. Significantly, 97% and 95% of the genome-derived and transcriptome-derived reads, respectively, could be aligned to the reference genome, ~~respectively,~~ suggesting no obvious bias ~~for~~in sequencing and assembly. Additionally, the mitochondrial genome of *P. canaliculata* was ~~also~~ assembled as a single contig ~~with~~ 15,707 bp in length, which has 99.9 % sequence identity to the published mitochondrial genome (GenBank: KJ739609.1) (Figure S2). Thise high-quality reference genome provides a good foundation for gene annotation.

The protein-coding genes were predicted on the reference genome by EVM, integrating evidence~~s~~ from *de novo* prediction, transcriptome and homology data. In total, 21,533 gene models were predicted as the reference gene set, with coding regions spanning ~32.2 Mb (7.3 %) of the genome (Table 1 and Table S2). The distribution of CDS length in *P. canaliculata* is similar to that in ~~the~~ closely related species (Figure 1c). Overall, 97.5 % of the reference genes were supported by transcriptome data, and 98.0 % of eukaryote core genes from OrthoDB (http://www.orthodb.org/) were identified in the reference gene set by BUSCO.~~,~~ These results were comparable to those in ~~the~~ other published molluscan genomes (Table 1). ~~For the~~ In functional annotation, a total of 19,815 (91.9 %) reference genes were annotated by at least one functional database. Specifically, 15,662 (72.7 %), 13,769

(63.4%), 17,081 (79.3%), 18,847 (87.5%) and 17,003 (79.9%) reference genes were annotated with the eggNOG, KEGG, NR, InterP~~p~~ro and UniP~~p~~rot databases, respectively (Figure S3).

## Signs of ~~Adaptive~~ adaptive ~~Evolution~~ evolution in *P. canaliculata* ~~Genome~~genome

Formatted: Font: Italic

To gain insight into the evolutionary perspective of *P. canaliculata*, ~~the~~ a phylogenetic tree was built based on 306~~471~~ high-confidence single-copy orthologous genes from nine~~seven~~ related species (*P. canaliculata*, *Lottia~~.~~ gigantea*, *Aplysia~~.~~ californica*, *Biomphalaria~~.~~ glabrata*, *Crassostrea~~.~~ gigas*, *Octopus~~.~~ bimaculoides*, *Pintada~~.~~ fucata*, *Lingula~~.~~ anatina* and *Limnoperna~~.~~ fortunei*) ~~*and L. anatina*)~~ by PhyML~~ml~~ [~~43~~44] and the divergence time was estimated using MCMCTree~~mcmctree~~ [~~44~~45]. The results show~~s~~ that *P. canaliculata* diverged from the ancestor of *B. glabrata* and *A. c~~C~~alifornica* 372~~290~~ million years ago (Mya)~~,~~ and from *L. gigantea* 491~~415~~ Mya (Figure 2a).

Then, the molluscan orthologous genes were investigated for adaptive evolution. Utilizing pairwise protein sequence similarities, ~~the~~ gene family clustering was conducted by orthfinder [~~45~~46]. A total of 239,541~~152,878~~ reference genes from the ~~seven~~ nine species were clustered into 69,582~~68,942~~ orthologous groups, among~~st~~ which 14,766~~13,805~~ orthologous groups ~~with~~ contained at least two genes each. ~~Compared to other seven species, w~~We identified 66 orthologue groups ~~undergone~~that underwent ~~–~~common expansion in both ~~in~~ *P. canaliculata* and *L. ~~fortunei~~fortune* but not the other seven species. ~~In *P. canaliculata*, we identified 9,626 ortholog groups,~~

~~amongst which 117 and 5,462 ortholog groups undergone species-specific expansion, thus may play important roles in adaption to the environment as an invasive species.~~ The functions of these orthologous groups are mainly related to signal transduction;~~,~~ replication and repair~~,~~; translation, glycan biosynthesis~~,~~ and metabolism~~,~~; ~~L~~lipid metabolism;~~,~~ and the endocrine, immune and nervous systems ~~digestive, endocrine, signal transduction, immune, or carbohydrate metabolism and so on~~ (Figure S4). These relations~~It~~ suggests~~ed~~ that the ~~expansion~~ gene families that underwent expansion may play important roles in adaptation to the environment as invasive species.~~.~~

The high-coverage genome assembly enables a comprehensive analysis of the transposable elements (TEs), which play~~s~~ multiple roles in driving genome evolution in eukaryotes [~~46~~47]. In total, we identified 49.6 Mb TE sequences in the assembled *P. canaliculata* genome (Table 1), including 3.4 Mb long terminal repeats (LTRs), 27.2 Mb long interspersed elements (LINEs), 17.5 Mb DNA transposons and 1.5 Mb short interspersed elements (SINEs). Next, we ~~analyzed~~ analysed the divergence rate of ~~TEs for~~ each class of TEs among the available sequenced mollus~~c~~k genomes. Not~~e~~ably~~worthy~~, the TE class of DNA transposons showed a specific~~, interestingly, only the results of DNA transposons showed a unique~~ peak at a divergence rate of ~4% divergence rate for *P. canaliculata* and *C. gigas* (Figure 2b), indicating a recent explosion of DNA transposons in these two species. We analy~~z~~sed the expression of 709 genes, including DNA elements ~~that~~ restricted to the 4% peak inside the gene region, compared with that of the other genes ~~that~~ outside the 4% peak (Figure S5).

~~Differentially expressed genes (~~DEGs~~)~~ were defined here by P-values smaller than 0.05 for comparison of the treatment~~s~~ (heat, cold, heavy metal and air exposure) and control data. The percentages of DEGs in the 4% peak were higher than those of genes outside the peak (10.2% higher for heat, 8.6% higher for cold, 8.6% higher for heavy metal, and 7.3% higher for air exposure). Among the DEGs in the 4% peak, approximately ~~about~~ half ~~are~~were up-regulated, and the other half were ~~are~~ down-regulated. Moreover, the DEGs in the 4% peak were mainly enriched in cellular metabolic process, response to stimulus, localization and signaling ~~signaling~~ according to ~~by~~ GO annotation. These results indicated that genes in the 4% peak were likely to be more active in the response to ~~of~~ stimulus, promoting ~~the~~ potential plasticity in ~~the~~ stress adaptation.~~More than half of the DNA transposons belong to the DNA/hAT-Charlie TE family, which is ~22.7% of total DNA/hAT-Charlie TEs in the genome.~~ TEs are powerful facilitators of evolution that generate ~~by generating~~ “evolutionary potential” to introduce small adaptive changes within a lineage, and the importance of TEs in ~~to~~ stress responses and adaptation has been reported in numerous studies~~researches~~ [48, 49~~47, 48~~]. The recent explosion of DNA~~/hAT-Charlie~~ TEs in *P. canaliculata* could also play an important role~~s~~ in~~to~~ promoting~~e~~ the potential plasticity in ~~the~~ stress adaptation.

**Investigation of ~~Cellular~~ cellular homeostasis system underlying strong stress adaptation**

The h~~H~~omeostasis system plays a crucial role in ~~the~~ stress adaptability, providing the molecular basis for~~in~~ re-establishing ~~the~~ dynamic equilibrium after ~~the~~ challenges by

~~of~~ various environmental stressors, including temperature, air exposure, anthropogenic pollution and pathogens [50~~49~~]. In this ~~the present~~ study, we addressed three constituent parts of the cellular homeostasis system, which contributes to the successful ecological plasticity of *P. canaliculata* (Figure 3). The transcriptomes ~~data~~ of the hemocytes after different stimuli~~ius~~ (cold, heat, heavy metal and air exposure) were~~as~~ also sequenced and ~~analyzed~~ analysed to address the potential roles of these genes in the c~~C~~ellular homeostasis system.

The u~~U~~nfolded protein response (UPR) system is ~~makes~~ the central component ~~part~~ of protein homeostasis [51~~50~~]. Heat shock proteins (HSPs) act~~s~~ as molecular chaperones to maintain ~~the~~ correct folding, and heat shock transcription factor 1 (HSF1) ~~are~~ is responsible for the transcriptional induction of HSPs [52~~51~~]. In the *P. canaliculata* genome, 13 HSP70s, ~~,~~ 6 HSP90s, 7 HSP40s and 11 HSFs were identified (Table S3), and the expression of HSP90s and HSFs ~~were~~ was highly induced in response to ~~the stress of~~ heat, cold, heavy metal and air exposure (Table S4 and Figure S6). Inositol-requiring protein 1 (IRE1), protein kinase RNA-like ER kinase (PERK), and activating transcription factor 6 (ATF6) are three mediators recruited by the endoplasmic reticulum (ER) to regulate~~d~~ the UPR [53~~52~~]. We found putative coding genes of the three core mediators, their respective downstream transcription factors, and the corresponding recognition chaperones in the *P. canaliculata* genome (Table S3).

The x~~X~~enobiotic biotransformation system helps the molluscs adapt to toxicants, especially ~~the~~ pesticides in aquatic environments [54~~53~~]. Manual annotation ~~on~~ of this

genome identified 157 cytochrome P450s (CYP450s), 15 flavin-containing monooxygenases (FMOs), 53 glutathione S-transferases (GSTs) and 105 ATP binding cassette (ABC) transporters, most of which showed ~~an~~ up-~~regulation~~ regulated~~in~~ expression under stress (Table S3 and~~,~~ Table S4). These proteins ~~are evidenced~~ have been shown to function in contaminant ~~detecting~~detection, conjugative modification and expulsion for xenobiotic detoxification [55-57~~54-56~~].

The m~~M~~assive production of reactive oxygen species (ROS) and reactive oxygen intermediates (ROIs) induced by stress leads to many pathological conditions, and antioxidant systems protect the organism from superoxide [58~~57~~]. Four main antioxidant enzyme classes, namely, superoxide dismutase (SOD), catalase (CAT), peroxidase (Prx), and glutathione peroxidase (GPX), were found in ~~the~~ *P. canaliculata* and showed ~~with an elevating~~ elevated global expression in response to stress (Table S3 and~~,~~ Table S4).

Apoptosis is a process of cell death when sensing stress and the regulation of apoptosis maintains the dynamic homeostasis of the internal environment. In *P. canaliculata*, we propose the existence of both intrinsic and extrinsic apoptotic signaling ~~signaling~~ pathways, evidenced by the presence of homologous genes involved in both pathways. ~~It seems t~~These two pathways could be activated by cytochrome C and tumour necrosis factor receptor (TNFR), respectively (Table S3). ~~The i~~Inhibitors of apoptosis, such as XIAP, Bcl2 and Bak, are also detected and show ~~with an~~ increased expression in response to ~~the~~ stress (Table S4), which ~~are~~ is expected to delay the process of apoptosis ~~process~~ and ~~the~~ cell death in the stress

response.

**The expansion of the P450 gene family contribute to stress tolerance**

Cytochrome~~s~~ P450 (CYP) enzymes are a monooxygenase family with highly diverse structures and functions~~,~~ that have been widely ~~broadly~~ identified in all kingdoms of life [~~58~~59]. P450s ~~catalyze~~ catalyse the reductive scission of molecular oxygen~~,~~ and are responsible for the synthesis and metabolism of various molecules, including drugs, hormones, antibiotics, pesticides, carcinogens and toxins [~~59~~60]. The hormones they synthesize~~d hormones~~, such as glucocorticoids, mineralocorticoids, progestins, and sex hormones, are critical to stress response, growth and reproduction, and the endogenous and exogenous chemical metabolism participate in ~~helps the host~~ combat~~ting~~ ~~with the~~ toxic compounds [~~60~~61].

We found that the *P. canaliculata* CYP gene family had ~~greater level of~~ undergone an expansion compared to that in the other molluscs. We identified 157 genes in the genome of *P. canaliculata*~~,~~ and 128, 102, 135, 78, 52 and 94 genes in ~~from~~ *A. ~~California~~californica*, *B. glabrata*, *C. gigas*, *L. gigant~~aean~~*, *O. bimaculoides* and *P. fucata* respectively, using ~~under~~ the same standard (Figure 4a). An ~~The~~ expansive trend was also observed~~,~~ in comparison ~~compared~~ with other ~~the~~ model species, such as *Homo sapiens* (57), *Mus musculus* (102), *Dan~~r~~io rerio* (94) and *Drosophila melanogaster* (94) [62~~61~~]. G~~The g~~ene expansion was mainly found in the CYP2U and CYP3A sub-families, whereas ~~and~~ fewer genes were expanded in CYP4F. In mammals, CYP2U participates ~~plays a role~~ in the metabolism of fatty acids to

generate bioactive eicosanoid derivatives, potentially regulating the development of immune function [63~~62~~]. In *P. canaliculata*, 40 genes formed ~~forged into~~ the CYP2U clade, mainly ~~expressing~~ expressed in the hepatopancreas (Figure 4b and Table S5_a, Table S5_b). CYP3A is ~~acts as~~ a versatile enzyme ~~metabolizing~~ that metabolizes a wide range of xenobiotics, and its production ~~the productions~~ promotes the growth of various cell types [64~~63~~]. The 56 CYP3A genes are ~~have~~ comprehensively ~~expression~~ expressed in the hepatopancreas, gill and kidney (Figure 4b and Table S5_a, Table S5_b). CYP4F possesses epoxygenase activity, metabolizing fatty acids to epoxides to suppress hypertension, pain perception and inflammation [65~~64~~]. Twenty~~20~~ genes were identified in CYP4F, and Pc06G011748, Pc06G011460, Pc06G011458, Pc06G011459, Pc04G006708, Pc04G006710 and Pc04G006707~~several CYP4F genes~~ exhibited ~~present~~ highly induced expression levels under ~~the stress of~~ cold, heat, heavy metal and air exposure stress, indicating their critical roles in the stress tolerance (Figure 4b, ~~and~~ Table S5_a~~,~~ and Table S5_b).

**The identification of perivitellin genes and their high transcriptional levels in the ovary and albumen gland~~perivitellin gene expansion and high transcriptional level in ovary enhance reproduction~~**

~~To adapt to the fast invasion life, besides the strong ability to stress tolerance, t~~The *P. canaliculata* ~~possesses a high reproductive rate, and one important contributor is their distinct~~ has eggs characterized ~~with~~ by abundant nutrients, reddish or pinkish colour, aerial oviposition and neurotoxicity [~~22~~23, ~~34~~66],~~. In most gastropod eggs,~~ ~~and these characteristics are contributed by~~ due to the perivitelline ~~Perivitelline~~ Fluid (PVF),

with large amounts of nutrients which fills filled in the space between the eggshell and the embryo, and consists which is composed of carbohydrates, lipids and proteins (Figure 5a). termed perivitellins, which is not only responsible for the major supply of material and energy during embryogenesis, but also provide warning pigment and deadly toxicant against the predators [65]. In *P. canaliculata*, tThe PVF proteins iIn *P. canaliculata*, tinclude three major Perivitellins components, of PcOvo, PcPV2, and PcPV3 [67], collectively named perivitellins, which mtake up 90% of the total proteins, whereas most of the other dozens of low-abundance components each only account for less than 1% of the total proteins [35]. The perivitellins isare not only responsible for the major supply of materials and energy during embryogenesis, but also provide warning pigments and deadly toxicants against the predators [23, 68, 69]. of *P. canaliculata* (Pc) have been verified by proteomics approach and was further divided into three categories called Pc Ovorubin (PcOvo), PcPV2, PcPV3, which are all high-density lipoprotein (HDL) [66] (Figure 5a).

We identified 28 candidate PVF genes in *P. canaliculata*, by mapping each of the 59 fragmental PVF protein sequences derived from a previous proteomics study by Sun [35] to theirits best hit in the reference gene set of *P. canaliculata*, using BLASTPblastp with requirements of over 85% identity and at least 50% alignment length (Table S6). Then, the functional annotation of those fragmental proteins was also transferred to our identified PVF genes. We totally identified 18 perivitellin genes from the *P. canaliculata* genome, compared to 2 and 1 perivitellin genes from *A. californica* and *P. fucata* respectively, by aligning the seven reference perivitellin gene

~~sequences (NCBI accession AFQ23940.1, AFQ23939.1, AFQ23938.1, AFQ23945.1, AFQ23937.1, P0C8G7.2, P0C8G6.2) to each genome sequences with the same method (blastn e-value $10^{-20}$). It is apparent that the copy number of perivitellin genes was expanded in *P. canaliculata*, and our orthologous and paralogous gene family data by orthoFinder confirmed this. Among the 20 perivitellin genes in *P. canaliculate*, there are 2 PcOvo, 13 PcPV2, and 3 unclassified PVFs (Figure 5b and Table S6). The PcOvo carotenoprotein is responsible for the red coloration of the eggs and antioxidant to protect against sun radiation and desiccation [67, 68], while PcPV2 is reported to be neurotoxin implying lethal effect on rodents [22]. The expansion of these genes may enhance the underlying functions of nutrition and protection, offering the eggs an advantage of survival and improve the reproduction rate.~~

The transcriptome data show~~s~~ that 22 (79%) of the 28 candidate PVF genes exhibit their highest expression in the ovary and albumen gland (PVF protein synthesis factory) among all ~~the~~ 7 tissues (Figure 5b and Table S7), confirming that most of them are genuine ~~real~~ functional PVF genes. Six of ~~In~~ these 28 candidate PVF genes are~~, there are 6~~ perivitellin genes, including two PcOvo genes, Pc09G015543 (PcOvo2) and Pc09G015548 (PcOvo3)~~,~~; two PcPV2 genes, Pc07G012572 (PcPV2-31) and Pc07G012571 (PcPV2-67)~~,~~; and two possible PcPV3 genes, Pc09G015546 and Pc09G015547. The expression levels of these 6 genes in the ovary and albumen gland ~~for these 6 genes~~ are much higher than those of the other 22 candidate PVF genes.

By analy~~z~~sing the orthoFinder gene families that include orthologous and paralogous genes from *P. canaliculata* and 8 other sequenced mollusc species, we found that

these 28 candidate PVF genes were classified into 20 multiple-gene families (≥~~≥>=~~ 2 genes) and 7 single-gene families (only one gene) (Table S8). Notably~~eworthy~~, 5 of the 6 perivitellin genes were classified ~~fall~~ into single-gene families, except for Pc07G012571 (PcPV2-67), which ~~that~~ not only has homologous genes in other mollusc species but also has three paralogous genes in *P. canaliculata* itself. However, none of these three PcPV2-67 paralogous genes ~~of~~in *P. canaliculata* showed higher expression~~ed higher~~ ~~ly~~ in the ovary and albumen gland than in other tissues, indicating that they are~~were~~ likely not PVF~~-~~related genes, i.e.~~,~~ only Pc07G012571 plays a role in PVF. The nearly unique and single-copy nature of the 6 perivitellin genes in *P. canaliculata*, may be explained by the long evolutionary distance, over 200 Mya for *P. canaliculata* and its most closely relat~~ive~~ed species, *A. ~~C~~californica*, as well as numerous ~~plenty of~~ differences in their living characteristics and egg structures. ~~s.On the other hand, it also indicates~~ Another possible explanation is that these 6 major PVF genes may have experienced ~~fast~~rapid evolution in their~~the~~ history~~,~~ ~~in order~~ to adapt to the changing environment.

~~The expression of 18 *P. canaliculata* perivitelline genes were detected in 7 tissues, including embryo, testis, ovary, kidney, gill, hepatopancreas and hemocyte. The highest expression of each gene concentrated in embryo and two sexual gland testis and ovary, especially in the ovary (Figure 5b and Table S7), suggesting that their decoding proteins might be of importance in germ cell production and embryo development. Taken together, *P. canaliculata* distinguish its embryo development from other seven species on the preponderance of perivitellin gene number and high~~

**Formatted:** Font: Not Italic

~~expression level, that further promotes corresponding function of nutrients supplying and defense ability and eventually contribute to reproduction.~~

**The g~~G~~ut microbiome plays important roles in stress resistance and food digestion**

The gut microbiome is well known as the second genome of animals~~, which~~ and plays important~~key~~ roles in food digestion, immune ~~defense~~defence, ~~etc~~ and other processes that are essential to the animal hosts~~s~~. To investigate whether the gut microbiome ~~has influence on~~ influences the invasive life~~-~~style, we collected gut digesta samples from 70 ~~adults of~~ *P. canaliculata*~~,~~ ~~adults~~snails and generated 31 Gb of high-quality metagenomic data on the Illumina HiseqX10 platform. To our knowledge, this study is the first in~~high~~-depth sequencing of the snail gut microbiome. A total of 1,142,095 non-redundant genes were obtained~~,~~ with an average open reading frame (ORF) length of 604 bp (Table ~~S8~~S9). The taxonomic composition analysis showed that, at the phylum level, Proteobacteria was ~~the~~ predominant, followed by Verrucomicrobia, Bacteroidetes, Firmicutes, Spirochaetes, Actinobacteria, etc. (Table ~~S9~~S10_a). At the genus level, the most abundant genera included *Aeromonas*, *Enterobacter*, *Desulfovibrio*, *Citrobacter*, *Comamonas*, *Klebsiella* and *Pseudomonas*~~.~~ (Table ~~S9~~S10_b), most of which were also present~~ed~~ in ~~the snails of~~ *Achatina fulica* [70,71~~69, 70~~].

~~It is interesting that~~ Interestingly, some of the most abundant genera, such as *Desulfovibrio*, *Citrobacter* and Pseudomonas, were reported as having ~~to have~~ strong abilities to ~~of removing~~ remove heavy metals~~,~~ , by ~~mechanisms of~~ bioprecipitation

**Formatted:** Font: Not Italic

and bioabsorption [72-74~~71-73~~]. For example, the sulfur-reducing bacteria *Desulfovibrio* produces~~d~~ $H_2S$, which ~~that~~ precipitates metals~~,~~ and therefore ~~reduced~~ reduces the toxic effects of ~~dissolving~~ dissolved metals [~~71~~72]. Based on the KEGG pathway database, the complete sulfate reduction metabolism pathway was identified in the *P. canaliculata* gut microbiome. We suggested that these gut microbes might help *P. canaliculata* ~~to confront with~~ survive the environmental stress of heavy metals in harsh conditions. In addition, a large number of genes in ~~pathways related to of~~ xenobiotic~~s~~ biodegradation and metabolism pathways were annotated, corresponding to 288 KEGG orthologous groups (KOs) and 21 pathways (Table ~~S10~~S11). As many of the pathways, such as benzoate degradation, toluene degradation, xylene degradation and steroid degradation, could not be identified in the host genome through KO analysis, we suggested that ~~the~~ microbial detoxification abilities may contribute to the ability *P. canaliculata* to resist stresses caused by xenobiotics such as pesticides and environmental pollutants.

In ~~view of dietary~~ digestion, the gut microbes are ~~were~~ directly involved in the breakdown of the cellulose portion of the diet, and previous studies have isolated ~~some~~ cellulolytic bacteria and evaluated the cellulolytic enzyme activities [75~~74~~]. O~~In o~~ur work found~~,~~ a broader range of carbohydrate active enzymes (CAZymes) ~~were found~~. Of the 208 annotated CAZyme families, 99 were ~~Glycoside~~ glycoside ~~Hydrolase~~ hydrolase (GH) families (Table ~~S11~~S12). Enzymes that could be classified as cellulases, endohemicelluloses, debranching enzymes, and oligosaccharide-degrading enzymes were all ~~–~~identified~~presented~~. These findings

indicate that the gut microbiome provides ~~give~~ assistance ~~to~~ in digesting a broad range of food sources, enabling ~~making~~ *P. canaliculata* to grow rapidly and ~~in order fast to~~ adapt to an invasive life~~-~~style.

## Conclusion and discussion

Given its environmental invasiveness, broad stress adaptability and rapid reproduction, the golden apple snail *P. canaliculata* has received a vast amount of attention worldwide. However, the underlying genetic mechanisms of these properties haves not been comprehensively uncovered. The chromosome-~~—~~level genome of *P. canaliculata* presented in this study sheds the first light~~s~~ on into the genomic basis of its ~~the~~ ecological plasticity in response to various stressors. ~~M~~The major findings of this study include the recent explosion of DNA/hAT-Charlie TEs, the expansion of the P450 gene family and the constitution of the c~~C~~ellular homeostasis system, all of which ~~contributing~~ contribute to the plasticity ~~in~~ of the organism in ~~the~~ stress adaptation. Although the ~~defined~~ function of the recently originated TEs could not be confirmed, ~~the explosion of~~ TEs are considered ~~is deemed as~~ powerful facilitators in adaptive evolution, suggesting that ~~indicating its~~ their increased number plays an important role in the stress resistance of *P. canaliculata*~~'s stress resistance~~. The UPR system, ~~Xenobiotic~~ xenobiotic biotransformation system and ROS system are all major components of the ~~Cellular~~ cellular homeostasis system, and the ~~especially~~ P450s in particular underwent expansion ~~expands~~ with specific functions. In addition, exclusive perivitellin genes ~~are~~ were identified in ~~characterized from~~ the *P.*

*canaliculata* genome, and they are believed to ~~contributing~~ contribute to the high reproductive rate and the expansion of habitats. Furthermore, the gut metagenome ~~encodes~~ contains diverse ~~a rich~~ ~~array of~~ genes for food digestion and xenobiotic~~s~~ degradation. These findings collectively provide novel insight into the molecular mechanisms of ~~the~~ ecological plasticity and high invasiveness.

~~The rich phenotypic and genetic diversity of molluscs make them an excellent species group to address many valuable issues about evolution, ecology and function. However, the genomic resource of Mollusca is still insufficient compared with other close phylums, such as Arthropoda and Nematoda, and few molluscs could be employed as model organism. *P. canaliculata* possesses potential to be a model organism of molluscs because of several inherent characters. For example, *P. canaliculata* is easy to acquire, for it has a broad global distribution originated from a primarily circumtropical environment. Due to the high adaptability, rapid growth and efficient reproduction, *P. canaliculata* also facilitate the cultivation in laboratory.~~ In this study, w~~W~~e report a fine reference genome of *P. canaliculata* ~~in the present study~~, ~~which is the~~ first chromosome-~~-~~level Mollusca genome published ~~in Mollusca~~. ~~Together with the~~ With its easy acquisition~~ring~~, rapid growth and efficient reproduction, *P. canaliculata* possesses the potential to be a model organism of Mollusca. As its ~~the~~ cellular complexity and ~~the~~ conservation of pathways also make~~,~~ *P. canaliculata* ~~could be~~ a useful representative of Mollusca, ~~so~~ the genome described in this study can be used to advance our understanding of the molecular mechanisms involved in ~~for~~ various scientific questions regarding ~~issues in~~ Mollusca.

## Methods

### Samples collection and sequencing

Adults of *P. canaliculata* were collected from a local paddy field in Shenzhen, Guangdong province, China, and maintained in aerated freshwater at 15 ± 2 °C for a week before processing. Genomic DNA was extracted from the foot muscles of a single *P. canaliculata* for constructing PCR free Illumina 350-bp insert libraries and PacBio 20-kb insert library, and sequenced on Illumina HiSeq 2500 and PacBio SMRT platforms, respectively. The Hi-C library was prepared using the muscle tissue of another single *P.* *canaliculata* ~~*canaliculate*~~ by following methods: Nuclear DNA was cross-linked in situ, extracted, and then digested with a restriction enzyme. The sticky ends of the digested fragments were biotinylated, diluted, and then ligated to each other randomly. Biotinylated DNA fragments were enriched and sheared again for preparing the sequencing library, which was then sequenced on a HiSeq X Ten platform (Illumina).

Seven tissues including embryos (2 days post fertilization), gill, hemocytes, hepatopancreas, kidney, ovary and albumen gland and testis from six animals were collected as parallel samples. Next, animals were cultivated in 37 °C and 10 °C for 24 hours heat and cold tolerance, in $Cr^{3+}$(2mg $L^{-1}$), $Cu^{2+}$(0.2mg $L^{-1}$) and $Pb^{2+}$(1mg $L^{-1}$) for 24 hours heavy metal tolerance, and in waterless tank for 7 days air exposure. Then the hemocytes were harvested and stored, with three replicates for each group.

In final, total RNAs were extracted from the stored tissues of *P. canaliculata* materials, and then mRNAs were pulled out by beads with poly-T for constructing cDNA libraries~~total messenger RNAs (mRNA) were extracted from the stored tissues of *P. canaliculata* materials for constructing cDNA libraries~~ (insert 350-bp), and sequenced on an Illumina HiSeq 2500 sequencer.

The intestinal digesta from 70 adult snails of *P. canaliculata* were collected, pooled into 6 samples and stored at −20 °C until microbial DNA was extracted. A combination of cell lysis treatments was applied, including five freeze-thaw cycles (alternating between 65 °C and liquid nitrogen for 5 min), repeated beads-beating in ASL buffer (cat. no. 19082; Qiagen Inc.), and incubated at 95 °C for 15 min. DNA was isolated following the protocol reported protocol [76~~75~~]. Paired-end libraries of metagenomic DNA were prepared with an insert size of 350 base pairs (bp) following the manufacture's protocol (cat. no. E7645L; New England Biolabs). Sequencing was performed on Illumina HiSeq X10.

**Genome assembly and annotation**

The Illumina raw reads were filtered by trimming the adapter sequence and low-quality– regions~~part~~ (https://github.com/fanagislab/common_use), resulting in ~~a~~ clean and high-quality reads ~~data~~ with an average error rate < 0.001. For the PacBio raw data, the short subreads (< 2 kb) and low-quality (error rate > 0.2) subreads were filtered out, and only one representative subread was retained for each PacBio read.

**Formatted:** Font: Not Bold

The clean PacBio reads were assembled by the software ~~samrtdenovo~~ smartdenovo (https://github.com/ruanjue/smartdenovo), ~~then~~ after which Illumina reads were aligned to the contigs by BWA-MEM, and single base errors in the contigs were corrected by Pilon (v1.16) –with the parameters "-fix bases, -nonpf, -minqual 20". The *P. canaliculata* genome is highly heterozygous, as illustrated by the double peaks on the distribution curve of k~~K~~-mer frequency, and the current assembly algorithm tends to collapse homozygous regions and report heterozygous regions in alternative contigs. To obtain ~~get~~ a haploid reference contigs, we employed a whole-genome alignment (WGA) strategy with ~~by~~ MUMmer v3.23 to recognize and selectively remove alternative heterozygous contigs, which were characterized by shorter length (less than 200 kb) and the ability of most regions (more~~larger~~ than 50%) to~~can~~ be aligned to another larger contig with confident identity (higher than 80%). Next, Hi-C sequencing data were aligned to the haploid reference contigs by BWA-MEM, and then these contigs were clustered into chromosomes with LACH-ESIS (http://shendurelab.github.io/LACHESIS/~~http://shendurelab.github.io/LACHESIS/~~).

A de novo repeat library for *P. canaliculata* was constructed by RepeatModeler (v1.0.4; http://www.repeatmasker.org/RepeatModeler.html). TEs in the *P. canaliculata* genome were also identified by RepeatMasker (v4.0.6; http://www.repeatmasker.org/) using both the Repbase library and the de novo library. Tandem repeats in the *P. canaliculata* genome were predicted using Tandem Repeats Finder v4.07b [77]. The divergence rates of TEs were calculated between the identified TE elements in the genome and their consensus sequence at the TE family level.

**Formatted:** Font: Italic

**Field Code Changed**

The gene models in the *P. canaliculata* genome were predicted by EVidence Modeler v1.1.1 [7876], integrating evidences from ab initio predictions, homology-based searches and RNA-seq alignments. Then, these gene models were annotated by RNA-seq data, UniProt database and InterProScan software the protein-coding sequences were mapped by RNA-seq data and functionally annotated using UniProt and InterProScan (5.16-55.0) databases [7779]. Finally, the gene models were retained if they had at least one piece of supporting evidence from the UniProt database, InterProScan domain and RNA-seq data. Gene functional annotation was performed by aligning the protein sequences to the NCBI NR, UniProt, COG and KEGG databases with BLASTP v2.3.0+ under an E-value cutoff of $10^{-5}$ and choosing the best hit. The pPathway analysis and functional classification were conducted based on the KEGG database [8078]. InterProScan was used to assign preliminary GO terms, Pfam domains and IPR domains to the gene models.

A de novo repeat library for *P. canaliculata* was constructed by RepeatModeler (v1.0.4; http://www.repeatmasker.org/RepeatModeler.html). TEs in the *P. canaliculata* genome were also identified by RepeatMasker (v4.0.6; http://www.repeatmasker.org/) using both Repbase library and the de novo library. Tandem repeats in the *P. canaliculata* genome were predicted using Tandem Repeats Finder v4.07b [79]. The divergence rates of TEs were calculated between the identified TE elements in the genome and their consensus sequence at the TE family level.

**Formatted:** Font: (Default) Times New Roman, Font color: Black

**Evolutionary analysis**

Orthologous and paralogous groups were assigned from seven species (*P. canaliculata*, *Lottia gigantea*, *Aplysia californica*, *Biomphalaria glabrata*, *Crassostrea gigas*, *Octopus bimaculoides*, *Pintada fucata*, *Limnoperna fortunei* and *Lingula anatina*) by OrthoFinder [~~45~~46] with default parameters. Orthologous groups that contained only one gene for each species were selected to construct the phylogenetic tree. The protein sequences of each gene family were~~was~~ independently aligned by muscle v3.8.31 [~~80~~81] and then concatenated into one super-sequence. The phylogenetic tree was constructed by maximum likelihood (ML) using PhyML v3.0 [44~~43~~] with the best-fit model (LG+I+G) ~~that was~~ estimated by ProtTest3 [82~~81~~]. The Bayesian ~~Relaxed~~ relaxed ~~Molecular~~ molecular ~~Clock~~ clock (BRMC) approach was adopted to estimate the neutral evolutionary rate and species divergence time using the program MCMCTree, implemented in the PAML v4.9 package [45~~44~~]. ~~The calibration time (fossil record time) interval (173-398 Mya) of *Octopus bimaculoides* was adopted from previous results.~~

The tree was calibrated with the following time frames to constrain the age of the nodes between the species: minimum = 260 Ma and maximum = 290 Ma for *P. fucata* and *C. gigas* [83]; minimum = 450 Ma and maximum = 480 Ma for *A. californica* (or *B. glabrata*) and *L. giganta* [84]. The calibration time (fossil record time) interval (550-610 Mya) of *O. bimaculoides* was adopted from previous results [85].



**Formatted:** Font: Italic

**Formatted:** Font: Not Italic

**Formatted:** Font: Not Italic

**Transcriptome data analysis**

~~Transcriptome reads were mapped to the reference genome of *P. canaliculata* using TopHat (v. 2.1.0) with default settings.~~ Transcriptome reads were trimmed with the same method for genomic reads (https://github.com/fanagislab/common_use), and then mapped to the reference genome of *P. canaliculata* using TopHat (v. 2.1.0) with default settings. The expression level of each reference gene in terms of FPKM was computed by cufflinks v2.2.1. A gene was considered to be expressed if its FPKM >0. Differential gene expression analysis was conducted using cuffdiff v2.2.1.

Formatted: Font: Not Bold

**Metagenome data analysis**

Raw reads were cleaned to exclude adapter sequences, low~~-~~quality sequences, and ~~as well as~~ contaminated DNA. The adapter sequence was identified and trimmed from the reads ~~in reads were identified and trimmed~~ by an ungapped dynamic programming algorithm; the low-quality part (head or tail) of the reads ~~were~~ was trimmed off to ensure that the average error rate of the ~~left~~ remaing reads ~~is~~ was lower than 0.001; the reads that were mapped to ~~the~~ contaminated DNA by BWA-MEM [~~82~~86] were filtered out; and finally, shorter reads (length < 75~~-~~ bp) and unpaired reads were excluded to form a set of clean reads ~~data~~. The BWA database built for cleaning contamination included genomes of 10 species: the *P. canaliculata* genome, the *Brassica rapa* genome, the *Oryza sativa* genome, 2 *Angiostrongylus cantonensis* genomes, the *Caenorhabditis elegans* genome, the *~~S~~schistosoma mansoni* genome, the

Formatted: Font: Not Italic

*C~~c~~lonorchis sinensis* genome, the *F~~f~~asciola hepatica* genome, the *Danio rerio* genome, and the *human hg38* genome.

The clean reads were assembled by metaSPAdes (v3.11.1) [~~83~~87] in ~~under~~ paired-end mode for each sample.~~,~~ T~~t~~hen, gene prediction was performed on contigs longer than 500 bp by Prodigal (v2.6.3) [~~84~~88] with the parameter “-p meta”, and gene models with cds length less than 102 bp were filtered out. A non-redundant (NR) gene set (539,344 genes) was constructed using the gene models predicted from each sample~~s~~ by cd-hit-est (v4.6.6) [~~85~~89] with the parameter “-c 0.95 -n 10 -G 0 –a S 0.9”, which adopts a greedy incremental clustering algorithm and the criteria of identity > 95% and overlap > 90% of the shorter genes. Then, the clean reads were mapped onto this NR gene set by BWA-MEM with the criteria of alignment length ≥~~≥~~ 50bp and identity > 95%. The unmapped reads from all samples were assembled together, and the genes were predicted again. The newly predicted genes were combined with the previous gene set by cd-hit-est to obtain ~~get~~ a new NR gene set (1,147,339 genes). After the taxonomic assignments to the new NR gene set, 5244 genes classified as Eukaryota but not fungi were removed, and the final NR gene set (1,142,095 genes) was obtained.

The taxonomic assignments ~~for~~ of the final NR genes were made on the basis of DIAMOND [~~86~~90] protein alignment against the NCBI -NR database by CARMA3 [~~87~~91]. Functional annotation was performed by aligning all the protein sequences to the KEGG [~~88~~92] database (release 79) using DIAMOND and taking the best hit with the criteria of E-value < 1e-5. CAZymes were annotated with dbCAN (release 5.0)

[~~89~~93] using HMMER (v3.0) hmmscan [~~90~~94] by taking the best hit with an E-value < 1e-18 and coverage > 0.35.

The clean reads from each sample were aligned against the gene catalogue (1,142,095 genes) by BWA-MEM with the criteria of alignment length ≥~~≥~~ 50bp and identity > 95%. Sequence-based gene abundance profiling was performed as previously described [~~91~~95]. The taxonomic profiles of the samples were calculated by summing ~~adding~~ the gene abundance ~~together~~ according to the taxonomic assignment result.

**Abbreviations**

~~*P. Canaliculata, Pomacea canaliculata; L. gigantean, Lottia gigantean;*~~

*A. c~~C~~alifornica, Aplysia ~~California~~californica; B. glabrata, Biomphalaria glabrata; C. gigas, Crassostrea gigas; O. bimaculoides, Octopus bimaculoides;~~–~~ L. anatinae, Lingula anatinae; L. fortune, Limnoperna fortune; L. giganta, Lottia giganta; P. ~~C~~canaliculata, Pomacea canaliculata; P. fucata, Pinctada fucata;* Hem, hemocyte; Te, testis; Ov, ovary and albumen gland; Kn, kidney; GI, gill; Hp, hepatopancreas, Em, embryo; SSR, simple sequence repeats; mya, million years ago*; BLAST, basic local alignment search tool;* SNP, single nucleotide polymorphism; PVF, Pervitelline Fluid; Ovo, ovorubin; AFLP, amplified fragment length polymorphism; DEGs, differentially expressed genes; LPyS, Lipopolysaccharide; iTRAQ, Isobaric Tags For Relative, Absolute Quantitation; LC-MS/MS, Liquid Chromatography-tandem Mass

**Formatted:** Font: (Default) Times New Roman, 12 pt, Italic

Spectrometry; TEs, transposable elements; LTR, long terminal repeats; LINE, long interspersed elements; SINE, short interspersed elements; UPR, Unfolded protein response; HSPs, heat shock proteins; HSF1, heat shock transcription factor 1; PERK, protein kinase RNA-like ER kinase; ATF6,activating transcription factor 6; ER, endoplasmic reticulum; CYP450s, cytochrome P450s; FMOs, flavin-containing monooxygenases; GSTs, glutathione S-transferases; ABC, ATP binding cassette; ROS, reactive oxygen species; ROI, reactive oxygen intermediates; SOD, superoxide dismutase; CAT, catalase; Prx, peroxidase; GPX, glutathione peroxidase; TNFR, tumor necrosis factor receptor; NR, non-redundant genes; ORF, open reading frame; Kos, orthologous groups; CAZymes, carbohydrate active enzymes; GH, Glycoside Hydrolase.

**Availability of data and materials**

Tables S1 to ~~S11~~ S12 and Figures S1 to ~~S4~~ S6 are available in the supplementary information file. The raw sequencing data has been deposited in DDBJ/EMBL/GenBank under project accession PRJNA427478, SRR6425828 for genomic Illumina_PE125 sequencing data, SRR6425829 for genomic Illumina_PE150 sequencing data, SRR6425827 for genomic ~~Pacbio~~ PacBio sequencing data, SRR6429132~SRR6429164 for transcriptome sequencing data, and SRR6472920~SRR6472925 for gut microbiome data. All the analysis data have also been released for public use and can be freely accessed at AGIS

ftpsite: ftp://ftp.agis.org.cn/~fanwei/Pomacea_canaliculata_Genome/ .

## Authors' contributions

WF and WQ conceived the study and designed the experiments. CL and YZ performed the genome sequencing and assembly, BL performed annotation and evolutionary analysis. CL performed the stress tolerance analysis, YR performed the reproduction analysis, YZ performed the metagenome analysis. HW, SL, FJ, LY provide suggestions and help checking. WF, CL, BL, YR, YZ wrote the manuscript, and GZ help revise the manuscript. All authors read and approved the final manuscript.

## Competing interests

The authors declare that they have no competing interests.

## Acknowledgements

This project is supported by the National key research and development program of China (2016YFC1200600), Shenzhen science and technology program (JCYJ20150630165133395), Fund of Key Laboratory of Shenzhen (ZDSYS20141118170111640), and The Agricultural Science and Technology Innovation Program (ASTIP) of Chinese Academy of Agricultural Sciences(CAAS) &

Elite Youth Program of Chinese Academy of Agricultural Sciences. We thank Fanghao Wan, Jue Ruan, Yutao Xiao for providing constructive suggestions to this project.

## Legends of t~~T~~ables and f~~F~~igures

### Tables

**Table 1. Summary of assembly and annotation of mollusc~~k~~ genomes**

| Genome feature | *P. canaliculata* | *L. gigantea* | *A. californica* | *B. glabrata* | *C. gigas* | *O. bimaculoides* |
|---|---|---|---|---|---|---|
| Assembled sequences (bp) | 440,071,717 | 359,505,668 | 927,310,431 | 916,377,450 | 557,735,934 | 2~~,~~3,381,887,882 |
| Contig N50 size (bp) | 1,072,857 | 94,165 | 9,817 | 18,978 | 37,218 | 5,982 |
| Contig N90 size (bp) | 303,904 | 10,180 | 1,626 | 5,132 | 11,109 | 1,606 |
| Scaffold N50 size (bp) | 31,531,291 | 1,870,055 | 917,541 | 48,059 | 401,685 | 475,182 |
| Scaffold N90 size (bp) | 23,662,357 | 74,480 | 207,390 | 817 | 68,181 | 79,088 |
| GC content (%) | 40.3 | 33.3 | 40.3 | 36.0 | 33.4 | 36 |
| No. of gene models | 21,533 | 23,824 | 19,909 | 14,224 | 28,402 | 15,814 |
| Avg. CDS length (bp) | 1,497 | 1,136 | 1,568 | 1,066 | 1,472 | 1,535 |
| BUSCO (%) | 98.9 | 98.4 | 98.7 | 72.8 | 99.4 | 98.7 |
| Transposable elements (bp) | 49,579,006 | 37,369,817 | 202,174,499 | 189,550,886 | 103,381,274 | 737,398,096 |
| Tandem repeat (bp) | 873,801 | 257,674 | 8,263,822 | 2,145,821 | 590,907 | 62,633,792 |

Formatted Table

### Figures

**Figure 1. The genome characteristics of *P. canaliculata*. (a)** Circos plot showing the genomic features**.** Track 1: 14 linkage groups of the genome; Track 2: distribution of transposon elements in chromosomes; Track 3: protein-coding genes located on chromosomes; Track 4: distribution of GC contents. **(b)** A genome-wide contact~~ing~~ matrix from Hi-C data between each pair of the 14 chromosomes~~,~~ using a 100 kb

window size. The colour value indicates ~~means~~ the base 2 logarithm of the number of valid reads ~~to base 2~~ ($log_2$(valid reads)). **(c)** Distribution of CDS length in six closely related species.

**Figure 2. Evolutionary genomic analysis of ~~between~~ *P. canaliculata* ~~and other molluscs~~.** **(a)** Phylogenetic placement of *P. canaliculata* within the ~~molluscs~~ dated tree of molluscs. The estimated divergence time ~~were~~ is shown ~~on~~ at each branching point, and *P. canaliculata* is shown ~~the species marked with~~ in red ~~color was *P. canaliculata*~~. **(b)** Distribution of divergence rate for the class of DNA transposons in molluscs genomes. The divergence rate was calculated by comparing all TE sequences identified in the genome to the ~~its~~ corresponding consensus sequence in each TE subfamily. The red arrow indicates ~~the~~ that *P. canaliculata* and *C. gigas* had a recent explosion of TEs at a divergence rate of ~4% ~~divergence rate~~.

**Formatted:** Font: Italic

**Formatted:** Font: Italic

**Figure 3. The cellular homeostasis system in *P. canaliculata*.** The u~~U~~nfolded protein response (UPR) system ~~included~~ includes HSPs and HSF in the heat shock response and CNX, NEF, GRP94, BIP, HSP40, ATF6, IRE1, PERK, COP2, XBP, ATF4, TRAM and Derlin in the endoplasmic reticulum unfolded protein response (UPR-ERAD). Apoptotic pathways include~~d~~ XIAPs, Bcl2, caspases, TNFR, and FADD. The antioxidant systems include~~d~~ PRX, SOD, CAT and GPX. The xenobiotic biotransformation system ~~included~~ includes EPHX3, P450, FMO and ABC transporter. The colours of the ~~Gene~~ boxes for gene families ~~with the filled colors~~ represent the degree of upregulation (FPKM-stimulus/FPKM-control) ~~by~~ as an over~~e~~all result of stress, including heat, cold, heavy metal and air exposure. Pathways and genes were obtained based on KEGG annotation.

**Figure 4. The expansion of the P450 gene family in *P. canaliculata*.** (a) Phylogenetic tree demonstrating orthologous and paralogous relationships of all P450 genes from 7 species including *P. canaliculata*, *A. californica*, *B. glabrata*, *C. gigas*, *L. gigant~~e~~a*, *O. bimaculoides* and *P. fucata*. P450 genes from seven species were obtained based on Pfam annotation (Interpro) with an ~~the~~ E-value of $10^{-5}$. Clades are labelled by P450 subfamily names. The tree was constructed using the ~~Maximum~~ maximum likelihood method in MEGA7, and the branch length scale indicates the average number of residue substitutions per site. (b) Phylogenetic tree of P450 genes in *P. canaliculata*, which is a subset of the phylogenetic tree for the 7 species, and their heat map of expression (FPKM) in seven tissues (Hem, hemocyte; Te, testis; Ov, ~~Ovary~~ovary and albumen gland; Kn, kidney; Gl, gill; Hp, hepatopancreas; Em, ~~Embryo~~embryo)~~,~~ and heat map of induced expression (FPKM-stimulus/FPKM-control) under stress (Con: control; heat; cold; Hm: heavy metal; Exp: air exposure).

**Figure 5. The composition and expression of the *P. canaliculata* perivitellins ~~composition and expression~~ in different tissues.** (a) Perivitelline ~~Fluid~~ fluid (PVF) ~~is~~ lies under the eggshell and surrounds the embryo.~~,~~ ~~I~~It contains carbohydrates, lipids, and proteins.~~,~~ T~~and t~~he proteins ~~is~~ are also known as perivitellins and are classified into three categories, ~~of~~ PcOvo, PcPV2, and PcPV3. (b) The displayed ~~shown~~ expression value of PVF proteins is the base 10 logarithm of FPKM ~~to base 2~~

(~~$log_2$FPKM~~$log_{10}$FPKM). The ~~first 3 letters in each gene ID~~ genes marked in red encode ~~refer to three classes of~~ perivitellins~~, uPV means unclassified perivitellins, PV2 means PcPV2, Ovo means PcOvo~~. ~~Abbreviations were used for~~ The 7 tissues examined are abbreviated as follows: ~~(~~Hem, hemocyte; Te, testis; Ov, ~~Ovary~~ovary and albumen gland; Kn, kidney; Gl, gill; Hp, hepatopancreas; Em, ~~Embryo~~embryo~~)~~.

## References


1. Lowe S, Browne M, Boudjelas S, de Poorter M. 100 of the World's Worst Invasive Alien Species: A selection fromthe Global Invasive Species Database. Auckland, New Zeland: World Conservation Union (IUCN); 2000.
2. Ranamukhaarachchi SL, Wickramasinghe S. Golden apple snails in the world: introduction, impact, and control measures. Global advances in ecology and management of golden apple snails. 2006:133-52.
3. Naylor R. Invasions in Agriculture: Assessing the Cost of the Golden Apple Snail in Asia. Royal Swedish Academy of Sciences. 1996;25:443-8.
4. Berthold T. Vergleichende Anatomie, Phylogenie und historische Biogeographie der Ampullariidae: (Mollusca, Gastropoda). 1991.
5. Howells RG, Burlakova LE, Karatayev AY, Marfurt RK, Burks RL. Native and introduced Ampullaridae in North America: History, status, and ecology. 2006:73-112.
6. Halwart M, Bartley DM. International mechanisms for the control and responsible use of alien species in aquatic ecosystems, with special reference to the golden apple snail. Los

Baños, Philippines: Philippine Rice Research Institute (PhilRice); 2006.

7. López MA, Altaba CR, Andree KB, López V. First invasion of the Apple snail Pomacea insularum in Europe. Tentacle. 2010;18:26-8.

8. Estebenet AL, Martín PR. Pomacea canaliculata (Gastropoda: Ampullariidae): life-history traits and their plasticity. Biocell 2002;26:83-9.

9. Lach L. The spread of the introduced freshwater apple snail *Pomacea canaliculata* (Lamarck) (Gastropoda Ampullariidae) on Oahu, Hawaii. Bishop Museum Occasional Papers. 1999;58:66-71.

10. Yusa Y，Sugiura N, Wada T. Predatory Potential of Freshwater Animals on an Invasive Agricultural Pest, the Apple Snail *Pomacea canaliculata* (Gastropoda: Ampullariidae), in Southern Japan. Biol Invasions. 2006;8:137-47.

11. Lach L, Britton DK, Rundell RJ, Cowie RH. Food Preference and Reproductive Plasticity in an Invasive Freshwater Snail. Biol Invasions. 2000;2:279-88.

12. Mochida O. Spread of freshwater *Pomacea* snails (Pilidae, Mollusca) from Argentina to Asia. Micronesica. 1991;3 51-62.

13. Shan L, Zhang Y, Steinmann P, Zhou X. Emerging Angiostrongyliasis in Mainland China. Emerg Infect Dis. 2008;14:161-4.

14. Caldeira RL, Mendonca CL, Goveia CO, Lenzi HL, Graeff-TeixeiraC Lima WS, et al. First record of molluscs naturally infected with *Angiostrongylus cantonensis* (Chen, 1935) (Nematoda: Metastrongylidae) in Brazil. Memórias do Instituto Oswaldo Cruz. 2007;102:887-9.

15. McMichael AJ, Beaglehole R. The changing global context of public health. Lancet

(London, England). 2000;356:495-9.

16. Chapman A. Numbers of Living Species in Australia and the World. Australian Biological Resources Study; 2009.

17. Lindberg DR, Ponder WF, Haszprunar G. The Mollusca: relationships and patterns from their first half-billion years. Oxford University Press, Oxford; 2004.

18. Hayes KA, Cowie RH, Thiengo SC. A global phylogeny of apple snails: Gondwanan origin, generic relationships, and the influence of outgroup choice (Caenogastropoda: Ampullariidae). Biol J Linn Soc Lond. 2009;98:61-76.

19. Matsukura K, Tsumuki H,Izumi Y, Wada T. Physiological response to low temperature in the freshwater apple snail, *Pomacea canaliculata* (Gastropoda: Ampullariidae). J Exp Biol. 2009;212:2558-63.

20. Yusa Y, Wada T, Takahashi S. Effects of dormant duration, body size, self-burial and water condition on the long-term survival of the apple snail, *Pomacea canaliculata* (Gastropoda: Ampullariidae). Appl Entomol Zool. 2006;41:627-32.

21. Seuffert ME, Burela S, Martín PR. Influence of water temperature on the activity of the freshwater snail Pomacea canaliculata (Caenogastropoda: Ampullariidae) at its southernmost limit (Southern Pampas, Argentina). Journal of Thermal Biology. 2010; 35:77-84.

~~21~~22. Kruatrachue M, Sumritdee C, Pokethitiyook P, Singhakaew S. Histopathological effects of contaminated sediments on golden apple snail (*Pomacea canaliculata*, Lamarck 1822). Bull Environ Contam Toxicol. 2011;86:610-4.

~~22~~23. Dreon MS, Frassa MV, Ceolín M, Ituarte S, Qiu JW, Sun J, et al. Novel animal defenses

against predation: a snail egg neurotoxin combining lectin and pore-forming chains that resembles plant defense and bacteria attack toxins. PLoS One. 2013;8:e63782. doi:10.1371/journal.pone.0063782.

~~23~~24. Ottaviani E, Caselgrandi E, Fontanili P, Franceschi C. Evolution, immune responses and stress: studies on molluscan cells. Acta Biol Hung. 1992;43:293-8.

~~24~~25. Ottaviani E, Accorsi A, Rigillo G, Malagoli D, Blom JM, Tascedda F. Epigenetic modification in neurons of the mollusc *Pomacea canaliculata* after immune challenge. Brain Res. 2013;1537:18-26.

~~25~~26. Mercado Laczkó AC, Lopretto EC. Estudio cromosómico y cariotípico de p*omacea canaliculata* (Lamarck, 1801) (Gastropoda, Ampullariidae). Revista del Museo Argentino de Ciencias Naturales "Bernardino Rivadavia" Hidrobiología. 1998;8:15-20.

~~26~~27. Xu J, Han X, Li N, Yu J, Qian C, Bao Z. Analysis of genetic diversity of three geographic populations of *Pomacea canaliculata* by AFLP. Acta Ecol Sin. 2009;29:4119- 26.

~~27~~28. Chen L, Xu H, Li H, Wu J, Ding H, Liu Y. Isolation and characterization of sixteen polymorphic microsatellite loci in the golden apple snail *Pomacea canaliculata*. Int J Mol Sci. 2011;12:5993-8.

~~28~~29. Mu X, Hou G, Song H, Xu P, Luo D, Gu D, et al. Transcriptome analysis between invasive *Pomacea canaliculata* and indigenous *Cipangopaludina cahayensis* reveals genomic divergence and diagnostic microsatellite/SSR markers. BMC Genet. 2015;16:12.

~~29~~30. Sun J, Wang M, Wang H, Zhang H, Zhang X, Thiyagarajan V, et al. De novo assembly of the transcriptome of an invasive snail and its multiple ecological applications. Mol Ecol Resour. 2012;12:1133-44.

~~30~~31. Mu H, Sun J , Fang L, Luan T, Williams GA, Cheung SG, et al. Genetic Basis of Differential Heat Resistance between Two Species of Congeneric Freshwater Snails: Insights from Quantitative Proteomics and Base Substitution Rate Analysis. J Proteome Res. 2015;14:4296-308.

~~31~~32. Yang L, Cheng TY, Zhao FY. Comparative profiling of hepatopancreas transcriptomes in satiated and starving *Pomacea canaliculata*. BMC Genet. 2017;18:18.

~~32~~33. Xiong YM, Yan ZH, Zhang JE, Li HY. Analysis of albumen gland proteins suggests survival strategies of developing embryos of *Pomacea canaliculata*. Molluscan Res. 2017:1-6.

~~33~~34. Sun J, Mu H , Zhang H, Chandramouli KH, Qian PY, Wong CK, et al. Understanding the regulation of estivation in a freshwater snail through iTRAQ-based comparative proteomics. J Proteome res. 2013;12:5271-80.

~~34~~35. Sun J, Zhang H, Wang H, Heras H, Dreon MS, Ituarte S, et al. First proteome of the egg perivitelline fluid of a freshwater gastropod with aerial oviposition. J Proteome Res. 2012;11:4240-8.

~~35~~36. Aplysia Genome Project. Broad Institute. Vertebrate Biology Group. 2009. https://www.broadinstitute.org/aplysia/aplysia-genome-project

~~36~~37. Zhang G, Fang X, Guo X, Li L, Luo R, Xu F, et al. The oyster genome reveals stress adaptation and complexity of shell formation. Nature. 2012;490:49-54.

~~37~~38. ~~Takeuchi T, Kawashima T, Koyanagi R, Gyoja F, Tanaka M, Ikuta T, et al. Draft genome of the pearl oyster *Pinctada fucata*: a platform for understanding bivalve biology. DNA Res. 2012;19:117-30.~~

Du X, Fan G, Jiao Y, Zhang H, Guo X, Huang R, et al. The pearl oyster Pinctada fucata martensii genome and multi-omic analyses provide insights into biomineralization. Gigascience. 2017;6:1-12.

3839. Simakov O, Marletaz F, Cho SJ, Edsinger-Gonzales E, Havlak P, Hellsten U, et al. Insights into bilaterian evolution from three spiralian genomes. Nature. 2013;493:526-31.

3940. Albertin CB, Simakov O, Mitros T, Wang ZY, Pungor JR, Edsinger-Gonzales E, et al. The octopus genome and the evolution of cephalopod neural and morphological novelties. Nature. 2015;524:220-4.

4041. Sun J, Zhang Y, Xu T, Zhang Y, Mu H, Zhang Y, et al. Adaptation to deep-sea chemosynthetic environments as revealed by mussel genomes. Nat Ecol Evol. 2017;1:121. doi:10.1038/s41559-017-0121.

Uliano-Silva M, Dondero F, Dan Otto T, Costa I, Lima NCB, Americo JA, et al. A hybrid-hierarchical genome assembly strategy to sequence the invasive golden mussel Limnoperna fortunei. Gigascience. 2017. doi: 10.1093/gigascience/gix128

4142. Adema CM, Hillier LW, Jones CS, Loker ES, Knight M, Minx P, et al. Corrigendum: Whole genome analysis of a schistosomiasis-transmitting freshwater snail. Nat Commun. 2017;8:16153.

4243. Liu B, Shi Y, Yuan J, Hu X, Zhang H, Li N, et al. Estimation of genomic characteristics by analyzing k-mer frequency in de novo genome projects. Quantitative Biology 2013:arXiv:1308.2012 [q-bio.GN].

4344. Guindon S, Dufayard JF, Lefort V, Anisimova M, Hordijk W, Gascuel O. New algorithms and methods to estimate maximum-likelihood phylogenies: assessing the performance of

**Formatted:** Font: (Default) Times New Roman, 10.5 pt
**Formatted** ...
**Formatted** ...
**Formatted** ...
**Formatted** ...
**Formatted** ...
**Formatted** ...
**Formatted:** Font: (Default) Times New Roman, 10.5 pt

**Formatted:** Font: (Default) Times New Roman, 10.5 pt
**Formatted:** Indent: First line: 0", Left 3.5 ch
**Formatted** ...
**Formatted** ...
**Formatted** ...
**Formatted** ...
**Formatted** ...
**Formatted** ...
**Formatted:** Font: (Default) Times New Roman, 10.5 pt

PhyML 3.0. Syst Biol 2010;59:307-21. doi:10.1093/sysbio/syq010.

~~44~~45. Yang Z. PAML 4: phylogenetic analysis by maximum likelihood. Mol Biol Evol. 2007;24:1586-91. doi:10.1093/molbev/msm088.

~~45~~46. Emms DM, Kelly S. OrthoFinder: solving fundamental biases in whole genome comparisons dramatically improves orthogroup inference accuracy. Genome Biol. 2015;16:157. doi:10.1186/s13059-015-0721-2.

~~46~~47. Feschotte C, Wessler SR. Mariner-like transposases are widespread and diverse in flowering plants. Proc Natl Acad Sci U S A 2002;99:280-5.

~~47~~48. Hua-Van A, Le Rouzic A, Boutin TS, Filée J, Capy P. The struggle for life of the genome's selfish architects. Biol Direct. 2011;6:19.

~~48~~49. Werren JH. Selfish genetic elements, genetic conflict, and evolutionary innovation. Proc Natl Acad Sci U S A. 2011;108~~ Suppl 2~~:10863-70.

~~49~~50. Chrousos GP. Stress and disorders of the stress system. Nat Rev Endocrinol. 2009;5:374-81.

~~50~~51. Vabulas RM, Raychaudhuri S, Hayer-Hartl M. Protein folding in the cytoplasm and the heat shock response. Cold Spring Harbor perspectives in biology. 2010;2:a004390.

~~51~~52. Chen B，Retzlaff M，Roos T，Frydman J. Cellular Strategies of Protein Quality Control. Cold Spring Harbor Perspectives in Biology. 2011;3:a004374.

~~52~~53. Korennykh A and Walter P. Structural basis of the unfolded protein response. Annu Rev Cell Dev Biol. 2012;28:251-77.

~~53~~54. Chambers JE and Yarbrough JD. Xenobiotic biotransformation systems in fishes. Comp Biochem Physiol C. 1976;55:77-84.

~~54~~55. Mello DF, de Oliveira ES, Vieira RC, Simoes E, Trevisan R, Dafre AL, et al. Cellular and Transcriptional Responses of *Crassostrea gigas* Hemocytes Exposed in Vitro to Brevetoxin (PbTx-2) Mar Drugs. 2012;10: 583-97.

~~55~~56. Boutet I, Tanguy A, Moraga D. Characterisation and expression of four mRNA sequences encoding glutathione S-transferases pi, mu, omega and sigma classes in the Pacific oyster *Crassostrea gigas* exposed to hydrocarbons and pesticides. Mar Biol 2004;146:53-64.

~~56~~57. Deeley RG, Westlake C, Cole SP. Transmembrane transport of endo- and xenobiotics by mammalian ATP-binding cassette multidrug resistance proteins. Physiol Rev. 2006;86:849-99.

~~57~~58. Liu C, Zhang T, Wang L, Wang M, Wang W, Jia Z, et al. The modulation of extracellular superoxide dismutase in the specifically enhanced cellular immune response against secondary challenge of Vibrio splendidus in Pacific oyster (*Crassostrea gigas*). Dev Comp Immunol. 2016;63:163-70.

~~58~~59. Lamb DC, Lei L, Warrilow AG, Lepesheva GI, Mullins JG, Waterman MR, et al. The first virally encoded cytochrome p450. J Virol. 2009;83:8266-9.

~~59~~60. Urlacher VB, Girhard M. Cytochrome P450 monooxygenases: an update on perspectives for synthetic application. Trends Biotechnol. 2012;30:26-36.

~~60~~61. Sanderson T, van den Berg M. Topic 3.1: Interactions of xenobiotics with the steroid hormone biosynthesis pathway. Pure Appl Chem. 2003;75:1957-71.

~~61~~62. Goldstone JV, McArthur AG, Kubota A, Zanette J, Parente T, Jönsson ME, et al. Identification and developmental expression of the full complement of Cytochrome P450 genes in Zebrafish. BMC Genomics. 2010;11:643.

~~62~~63. Chuang SS, Helvig C, Taimi M, Ramshaw HA, Collop AH, Amad M, et al. CYP2U1, a novel human thymus- and brain-specific cytochrome P450, catalyzes omega- and (omega-1)-hydroxylation of fatty acids. J Biol Chem. 2004;279:6305-14.

~~63~~64. Fleming I. The pharmacology of the cytochrome P450 epoxygenase/soluble epoxide hydrolase axis in the vasculature and cardiovascular disease. Pharmacol Rev. 2014;66:1106-40.

~~64~~65. Zhang G, Kodani S, Hammock BD. Stabilized epoxygenated fatty acids regulate inflammation, pain, angiogenesis and cancer. Prog Lipid Res. 2014;53:108-23.

~~65~~66. de Jong-Brink M, Boer HH, Joosse J. Mollusca. In: Adiyodi, K.G., Adiyodi, R.G. (Eds.), Reproductive Biology of invertebrates. Oogenesis oviposition and oosorption, vol. 1. John Wiley & Sons Ltd., New York, 1983; pp. 297-355.

~~66~~67. Garin CF, Heras H, Pollero RJ. Lipoproteins of the egg perivitelline fluid of *Pomacea canaliculata* snails (Mollusca: Gastropoda). J Exp Zool. 1996;276:307-14.

~~67~~68. Dreon MS, Schinella G, Heras H, Pollero RJ. Antioxidant defense system in the apple snail eggs, the role of ovorubin. Arch Biochem Biophys. 2004;422:1-8.

~~68~~69. Dreon MS, Ituarte S, Heras H. The role of the proteinase inhibitor ovorubin in apple snail eggs resembles plant embryo defense against predation. PLoS One. 2010;5:e15059. doi:10.1371/journal.pone.0015059.

~~69~~70. Cardoso AM, Cavalcante JJV, Vieira RP, Lima JL, Grieco MAB, Clementino MM, et al. Gut Bacterial Communities in the Giant Land Snail *Achatina fulica* and Their Modification by Sugarcane-Based Diet. Plos One. 2012;7 doi:ARTN e3344010.1371/journal.pone.0033440.

7071. Cardoso AM, Cavalcante JJV, Cantão ME, Thompson CE, Flatschart RB, Glogauer A, et al. Metagenomic Analysis of the Microbiota from the Crop of an Invasive Snail Reveals a Rich Reservoir of Novel Genes. Plos One. 2012;7 doi:ARTN e4850510.1371/journal.pone.0048505.

7172. Cabrera G, Pérez R, Gómez JM, Ábalos A, Cantero D. Toxic effects of dissolved heavy metals on Desulfovibrio vulgaris and Desulfovibrio sp strains. J Hazard Mater 2006;135:40-6. doi:10.1016/j.jhazmat.2005.11.058.

7273. Finlay JA, Allan VJ, Conner A, Callow ME, Basnakova G, Macaskie LE. Phosphate release and heavy metal accumulation by biofilm-immobilized and chemically-coupled cells of a Citrobacter sp. pre-grown in continuous culture. Biotechnol Bioeng. 1999;63:87-97.

7374. Valls M, de Lorenzo V, Gonzalez-Duarte R, Atrian S. Engineering outer-membrane proteins in *Pseudomonas putida* for enhanced heavy-metal bioadsorption. J Inorg Biochem. 2000;79:219-23.

7475. Pinheiro GL, Correa RF, Cunha RS, Cardoso AM, Chaia C, Clementino MM, et al. Isolation of aerobic cultivable cellulolytic bacteria from different regions of the gastrointestinal tract of giant land snail *Achatina fulica*. Front Microbiol. 2015;6 doi:Artn 86010.3389/Fmicb.2015.00860.

7576. Zoetendal EG, Heilig HG, Klaassens ES, Booijink CC, Kleerebezem M, Smidt H, et al. Isolation of DNA from bacterial samples of the human gastrointestinal tract. Nature protocols 2006, 1(2): 870-873.

77. Benson G. Tandem repeats finder: a program to analyze DNA sequences. Nucleic Acids

Res. 1999;27:573-80.

78. Haas BJ, Salzberg SL, Zhu W, Pertea M, Allen JE, Orvis J, et al. Automated eukaryotic gene structure annotation using EVidenceModeler and the Program to Assemble Spliced Alignments. Genome Biol. 2008;9:R7.

~~77~~79. Quevillon E, Silventoinen V, Pillai S, Harte N, Mulder N, Apweiler R, et al. InterProScan: protein domains identifier. Nucleic Acids Res. 2005;33:W116-20.

~~78~~80. Kanehisa M, Goto S, Sato Y, Furumichi M, Tanabe M. KEGG for integration and interpretation of large-scale molecular data sets. Nucleic Acids Res. 2012;40:D109-D14.

~~79~~80. ~~Benson G. Tandem repeats finder: a program to analyze DNA sequences. Nucleic Acids Res. 1999;27:573-80.~~

~~80~~81. Edgar RC. MUSCLE: multiple sequence alignment with high accuracy and high throughput. Nucleic Acids Res. 2004;32:1792-7.

~~81~~82. Darriba D, Taboada GL, Doallo R, Posada D. ProtTest 3: fast selection of best-fit models of protein evolution. Bioinformatics. 2011;27:1164-5. doi:10.1093/bioinformatics/btr088.

83. Sun J, Zhang Y, Xu T, Zhang Y, Mu H, Zhang Y, et al. Adaptation to deep-sea chemosynthetic environments as revealed by mussel genomes. Nature Ecology & Evolution. 2017; 1; 121.

84. Benton MJ, Donoghue PCJ, Asher RJ. in The Timetree of Life:Calibrating and Constraining Molecular Clocks (eds Hedges, S. B. & Kumar, S.)35–86 (Oxford Univ. Press, 2009.

85. Zapata F, Wilson NG, Howison M, Andrade SC, Jörger KM, Schrödl M, et al. Phylogenomic analyses of deep gastropod relationships reject Orthogastropoda. Proc Biol

**Formatted:** Font: 10.5 pt, Not Highlight

**Formatted:** Font: 10.5 pt, Not Highlight

**Formatted:** Font: 10.5 pt, Not Highlight

**Formatted:** Font: 10.5 pt, Not Highlight

**Formatted:** Font: 10.5 pt, Not Highlight

**Formatted:** Font: 10.5 pt

**Formatted:** Font: 10.5 pt, Not Highlight

**Formatted:** Font: 10.5 pt, Not Highlight

**Formatted:** Font: 10.5 pt, Not Highlight

**Formatted:** Font: 10.5 pt, Not Highlight

**Formatted:** Font: 10.5 pt, Not Highlight

**Formatted:** Font: 10.5 pt, Not Highlight

Sci. 2014;281(1794):20141739. doi: 10.1098/rspb.2014.1739.

~~82~~86. Li H and Durbin R. Fast and accurate short read alignment with Burrows-Wheeler transform. Bioinformatics. 2009;25:1754-60.

~~83~~87. Nurk S, Meleshko D, Korobeynikov A, Pevzner PA. metaSPAdes: a new versatile metagenomic assembler. Genome Res. 2017;27:824-34.

~~84~~88. Hyatt D, LoCascio PF, Hauser LJ, Uberbacher EC. Gene and translation initiation site prediction in metagenomic sequences. Bioinformatics. 2012;28:2223-30.

~~85~~89. Fu L, Niu B, Zhu Z, Wu S, Li W. CD-HIT: accelerated for clustering the next-generation sequencing data. Bioinformatics. 2012;28:3150-2.

~~86~~90. Buchfink B, Chao X, Huson DH. Fast and sensitive protein alignment using DIAMOND. Nat Methods. 2015;12:59-60.

~~87~~91. Gerlach W and Stoye J. Taxonomic classification of metagenomic shotgun sequences with CARMA3. Nucleic Acids Res. 2011;39 doi:Artn E9110.1093/Nar/Gkr225.

~~88~~92. Kanehisa M, Goto S, Kawashima S, Okuno Y, Hattori M. The KEGG resource for deciphering the genome. Nucleic Acids Res. 2004;32:D277-80.

~~89~~93. Yin Y, Mao X, Yang J, Chen X, Mao F, Xu Y. dbCAN: a web resource for automated carbohydrate-active enzyme annotation. Nucleic Acids Res. 2012;40:W445-51.

~~90~~94. Eddy SR. Accelerated Profile HMM Searches. Plos Comput Biol. 2011;7 doi:ARTN e100219510.1371/journal.pcbi.1002195.

~~91~~95. Qin JJ, Li YR, Cai ZM, Li SH, Zhu JF, Zhang F, et al. A metagenome-wide association study of gut microbiota in type 2 diabetes. Nature. 2012;490:55-60.

Figure

Click here to download Figure Fig.1.pdf

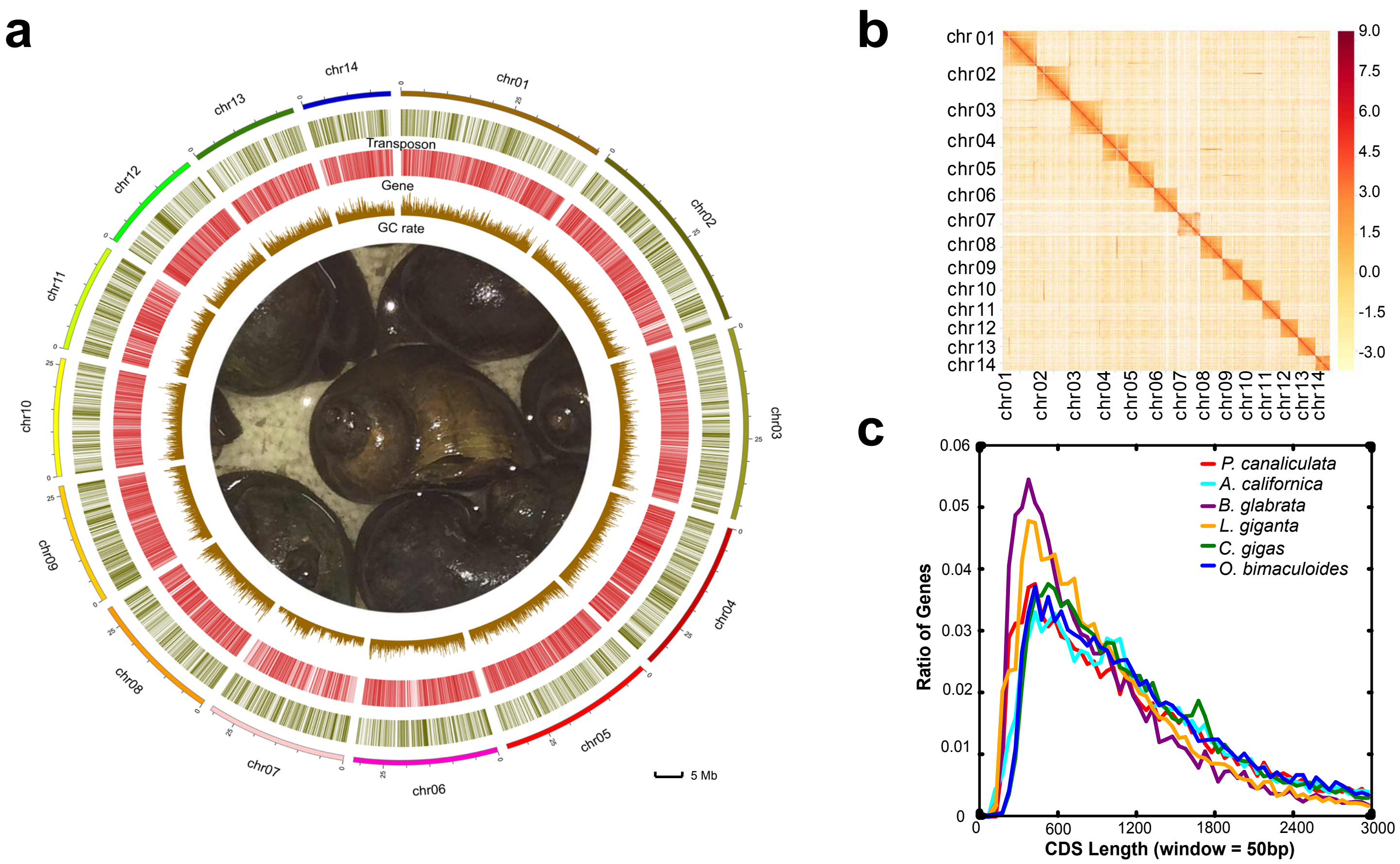

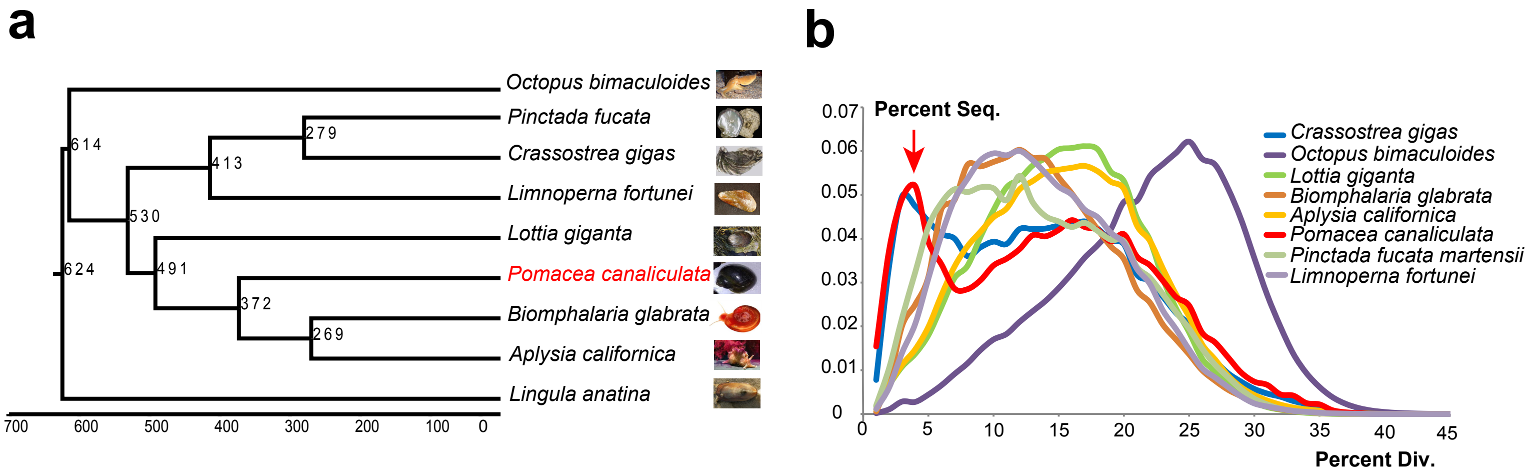
a
Octopus bimaculoides
Pinctada fucata
Crassostrea gigas
Limnoperna fortunei
Lottia giganta
Pomacea canaliculata
Biomphalaria glabrata
Aplysia californica
Lingula anatina
614
624
530
413
279
491
372
269
700
600
500
400
300
200
100
0
b
Percent Seq.
0.07
0.06
0.05
0.04
0.03
0.02
0.01
0
0
5
10
15
20
25
30
35
40
45
Percent Div.
Crassostrea gigas
Octopus bimaculoides
Lottia giganta
Biomphalaria glabrata
Aplysia californica
Pomacea canaliculata
Pinctada fucata martensii
Limnoperna fortunei

 Click here to download Figure Fig.3.pdf

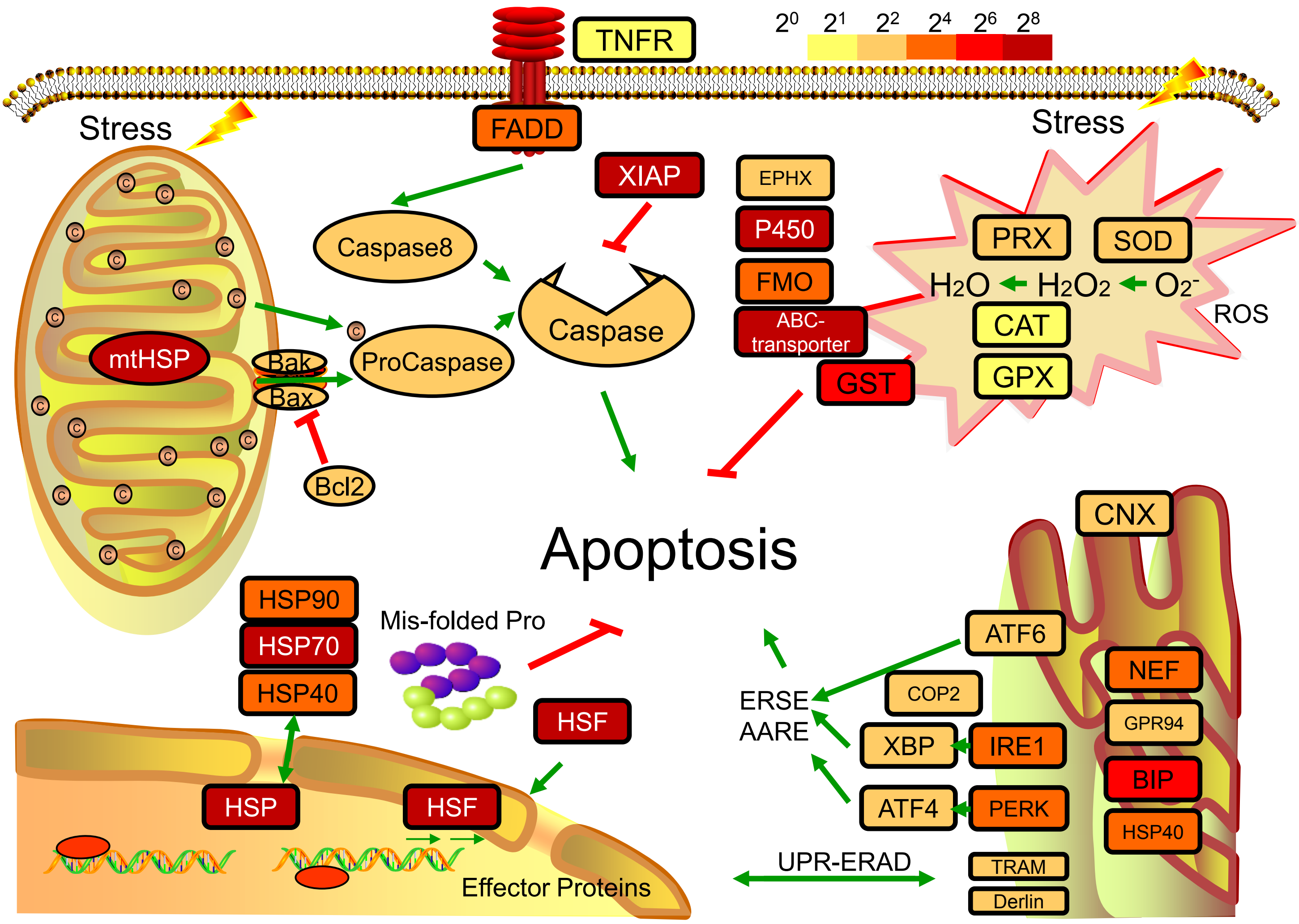

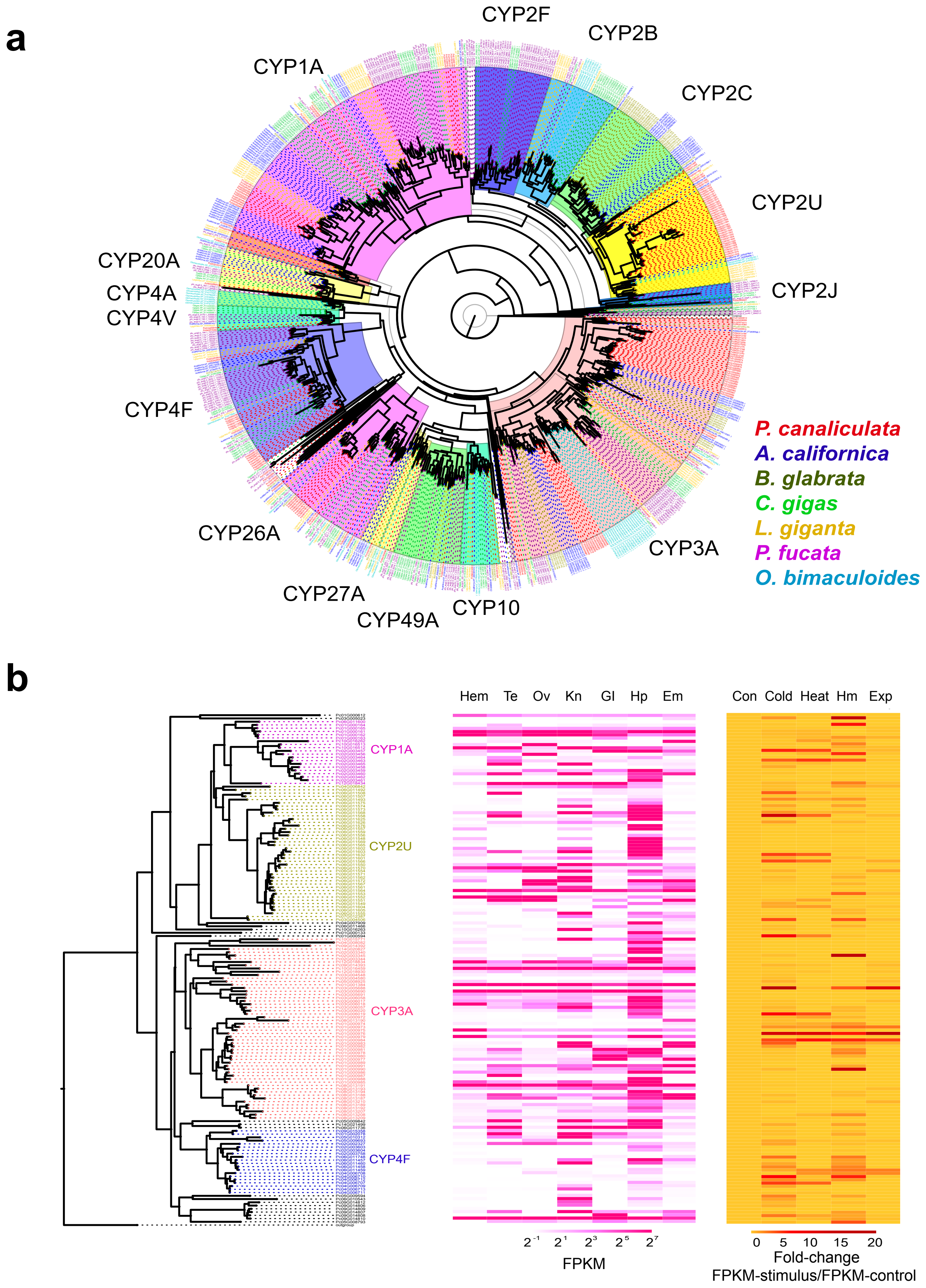
a
CYP2F
CYP2B
CYP1A
CYP2C
CYP2U
CYP20A
CYP2J
CYP4A
CYP4V
CYP4F
P. canaliculata
A. californica
B. glabrata
C. gigas
L. giganta
P. fucata
O. bimaculoides
CYP26A
CYP3A
CYP27A
CYP10
CYP49A
b
Hem Te Ov Kn Gl Hp Em
Con Cold Heat Hm Exp
CYP1A
CYP2U
CYP3A
CYP4F
2^-1 2^1 2^3 2^5 2^7
FPKM
0 5 10 15 20
Fold-change
FPKM-stimulus/FPKM-control

Figure Click here to download Figure Fig.5.pdf

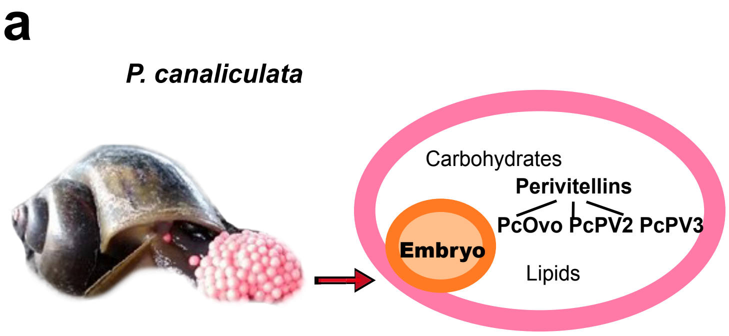


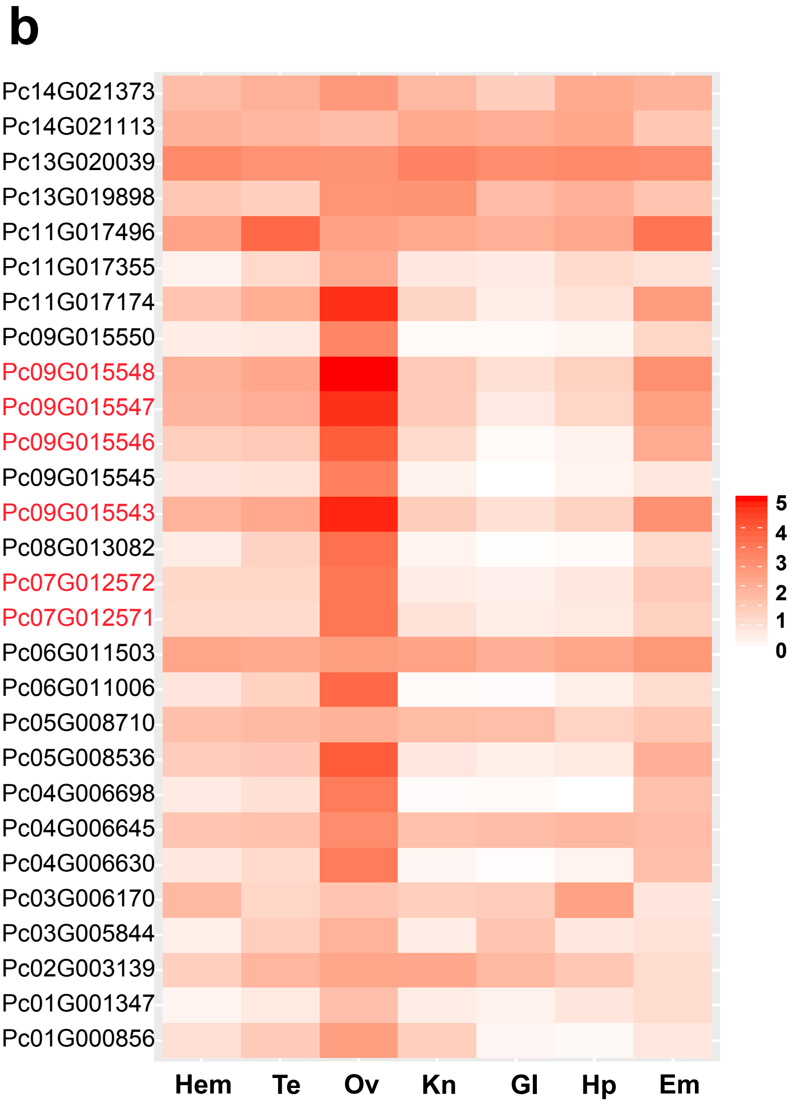

Click here to access/download
Supplementary Material
Supplemental_Information-final.doc

Click here to access/download
**Supplementary Material**
Supplemental_Information_with-tracks.doc
