# Supplementary material for: The genome of the golden apple snail Pomacea canaliculata provides insight into stress tolerance and invasive adaptation
Source: Gigascience. 2018 Aug 9;7(9):giy101. doi: 10.1093/gigascience/giy101 (PMC6129957; doi:10.1093/gigascience/giy101)
Supplement: GIGA-D-18-00030_Revision_2.pdf [file giy101_giga-d-18-00030_revision_2.pdf]

GigaScience

The genome of golden apple snail Pomacea canaliculata provides insight into stress tolerance and invasive adaptation

--Manuscript Draft--

Manuscript Number:	GIGA-D-18-00030R2
Full Title:	The genome of golden apple snail Pomacea canaliculata provides insight into stress tolerance and invasive adaptation
Article Type:	Research
Funding Information:	National key research and development program of China (2016YFC1200600) — Dr Wei Fan<br>Shenzhen science and technology program (JCYJ20150630165133395) — Dr Wei Fan<br>Fund of Key Laboratory of Shenzhen (ZDSYS20141118170111640) — Dr Wei Fan<br>The Agricultural Science and Technology Innovation Program (ASTIP) of Chinese Academy of Agricultural Sciences(CAAS) & Elite Youth Program of Chinese Academy of Agricultural Sciences — Dr Wei Fan
Abstract:	Background: The golden apple snail (Pomacea canaliculata) is a fresh water snail listed among the top-100 worst invasive species, worldwide and a noted agricultural and quarantine pest that causes great economic losses. It is characterized by fast growth, strong stress tolerance, a high reproduction rate, and adaptation to a broad range of environments.<br>Results: Here, we used long-read sequencing to produce a 440-Mb high-quality chromosome-level assembly for the P. canaliculata genome. In total, 50 Mb (11.4%) repeat sequences and 21,533 gene models were identified in the genome. The major findings of this study include the recent explosion of DNA/hAT-Charlie transposable elements (TEs), the expansion of the P450 gene family and the constitution of the cellular homeostasis system, which contributes to ecological plasticity in stress adaptation. In addition, the high transcriptional levels of perivitellin genes in the ovary and albumen gland promote the function of nutrient supply and defence ability in eggs. Furthermore, the gut metagenome also contains diverse genes for food digestion and xenobiotic degradation.<br>Conclusions: These findings collectively provide novel insights into the molecular mechanisms of the ecological plasticity and high invasiveness.
Corresponding Author:	Wei Fan<br>Chinese Academy of Agricultural Sciences<br>CHINA
Corresponding Author Secondary Information:	
Corresponding Author's Institution:	Chinese Academy of Agricultural Sciences
Corresponding Author's Secondary Institution:	
First Author:	Conghui Liu
First Author Secondary Information:	
Order of Authors:	Conghui Liu
	Yan Zhang
	Yuwei Ren
	Hengchao Wang
	Shuqu Li

Powered by Editorial Manager® and ProduXion Manager® from Aries Systems Corporation

	Fan Jiang
	Lijuan Yin
	Xi Qiao
	Guojie Zhang
	Wanqiang Qian
	Bo Liu
	Wei Fan
**Order of Authors Secondary Information:**	
**Response to Reviewers:**	Reviewer reports:<br>Reviewer #1: Still there are some typos in the revised manuscript.<br>For example, in line 181 "Pintada" must be "Pinctada".<br>Please carefully check the manuscript again before submission.<br>Reply: We have revised all the typos in the manuscript. We revised “Lottia giganta” to “Lottia gigantea” in line 161, “Pintada fucata” to “Pinctada fucata” in line 162, “giganta” to “gigantea” in line 166, “orthfinder” to “orthoFinder” in line 169, “L. fortune” to “L. fortunei” in line 172, “L. giganta” to “L. gigantea” in line 259, “Lottia giganta” to “Lottia gigantea” in line 462, “Pintada fucata” to “Pinctada fucata” in line 463, “giganta” to “gigantea” in line 476, “L. fortune” to “L. fortunei” in line 543, “L. giganta, Lottia giganta” to “L. gigantea, Lottia gigantea” in line 543, “L. giganta” to “L. gigantea” in Table 1, “L. giganta” to “L. gigantea” in the legend of Figure 4.<br><br>Reviewer #3: Dear authors,<br><br>Thank you for providing a revised version of the manuscript and for addressing my suggestions. I think this manuscript will be a great contribution for the genomic studies of mollusks and invasive species. I, however, still have a few comments.<br><br>1-) The written English is much improved, but there are still a few persistent mistakes. Such as "L. giganta" where it should be 'L. gigantea' and the same with "L. fortune" which is actually 'L. fortunei'.<br>Reply: We have revised “L. giganta” to “L. gigantea”, and “L. fortune” to “L. fortunei” in the manuscript. We revised “Lottia giganta” to “Lottia gigantea” in line 161, “giganta” to “gigantea” in line 166, “L. fortune” to “L. fortunei” in line 172, “L. giganta” to “L. gigantea” in line 259, “Lottia giganta” to “Lottia gigantea” in line 462, “giganta” to “gigantea” in line 476, “L. fortune” to “L. fortunei” in line 543, “L. giganta, Lottia giganta” to “L. gigantea, Lottia gigantea” in line 543, “L. giganta” to “L. gigantea” in Table 1, “L. giganta” to “L. gigantea” in the legend of Figure 4.<br><br>I've attached again a manuscript with some purple highlights of critical pieces of text that should be revised. For example, the sentence between lines 479-484 is too long and non-technical. The same for "With its easy acquisition" in line 377. The improvement of such sentences would greatly benefit the manuscript readers.<br>Reply: (1) The long and non-technical sentence between lines 479-484 “Raw reads were cleaned to exclude adapter sequences, low-quality sequences, and contaminated DNA. The adapter sequence was identified and trimmed from the reads by an ungapped dynamic programming algorithm; the low-quality part (head or tail) of the reads was trimmed off to ensure that the average error rate of the remaining reads was lower than 0.001; the reads that were mapped to contaminated DNA by BWA-MEM were filtered out…” has been revised to short sentences, with the non-technical description removed and the applied in-house program cited:<br>“The Illumina raw reads were filtered by trimming the adapter sequence and low-quality regions (https://github.com/fanagislab/common_use), resulting in high-quality reads with an average error rate < 0.001. Then, the reads mapped to the following genomes by BWA-MEM were filtered out (https://github.com/fanagislab/metagenome_analysis.git), to exclude the contaminated host, food, parasite, and human DNA sequences …”<br>(2) The “With its easy acquisition” in line 377 has been revised to “With wide distribution”, and the whole sentence became: “With wide distribution, rapid growth and efficient reproduction, P. canaliculata possesses the potential to be a model organism

Powered by Editorial Manager® and ProduXion Manager® from Aries Systems Corporation

of Mollusca.”

(3) The “orthologue groups” in line 170 has been revised to “orthologous groups”.

(4) The “maintains” in line 238 has been revised to “contributes to”, and the whole sentence became: “Apoptosis is a process of cell death when sensing stress, and the regulation of apoptosis contributes to the dynamic homeostasis of the internal environment.”

(5) The sentence between lines 319-322 “The gut microbiome is well known as the second genome of animals and plays important roles in food digestion, immune defence, and other processes that are essential to the animal host. To investigate whether the gut microbiome influences the invasive lifestyle” has been improved to: “The gut microbiome is regarded as the “second genome” of the host animal, due to the fact that the gut microbiota contributes to the food digestion, immune system development, and many other processes important to the host. To investigate the relationship between the gut microbiome and the invasive lifestyle of P. canaliculata.”

Also, the final subtopic should not be "Conclusion and Discussion", at that point, I would say, its time to just conclude.
Reply: We have deleted “and Discussion” in the subtopic.

In the results sections, however, many paragraphs start with a discussion of the literature instead of presenting the results: I would advice to revise those, present results first in the paragraphs and then discuss them. Again, coherence benefit readers a great deal.
Reply: Yes, we agree that results should be presented in front of discussion. To make it easier to understand for the readers, the sentences in the head of these paragraphs are brief background information, not discussion on the results. Real discussions are put after the results, in the end of the paragraphs.

2-) The amount of data generated is one of the strongest points of the work presented. And specially because of that, a great deal of analysis can be performed. For example, as you have 60x coverage of PacBio data for the snail, I would suggest running the Falcon and Falcon-Unzip pipeline to actually phase the genome: separate the haplotypes, instead of trying to merge or just through away the variation, as described in lines 424-432. The high heterozygosity described for the species actually helps in the phasing of haplotypes: there are several manuscripts describing methods to do so. I would run FALCON and FALCON-unzip, then I would polish with Illumina and try filling gaps with it in the different haplotypes and then would use the Hi-C data. I know its a great deal of analysis and highly experimental, so I'll leave it as a suggestion. But I would be interested in having a supplementary material with the imperfect alternate contigs generated by the phasing. This is the kind of information that were almost impossible to obtain with the generation of short reads, but now the long-reads technologies allow us to phase some long genome portions, and this is a very valuable information to some of us. With that, we can start understanding how much variation there are - and what are their evolutionary implications - in coding and non-coding regions within a genome.

Reply: Assembly the two haplotype chromosomes with long-reads is a very good suggestion, and we agree that the phased chromosomal sequences have greater value than the current mosaic reference genome sequence. In fact, we have run both SMARTdenovo and Falcon/Falcon-unzip, and polished by Pilon with illumina reads. The biggest difference of SMARTdenovo from Falcon is that SMRTdenovo does not need to correct sequencing errors in the first step, but instead perform an overlap-layout-consensus algorithm directly. With algorithms improved in many aspects, SMARTdenovo can achieve good assembly results with moderate sequencing coverage (50 X), in contrast, Falcon usually needs higher sequencing coverage (100 X) to get a good assembly. In this study, using the 60 X apple snail Pacbio data, SMARTdenovo generates contigs with N50 length over 1 megabases, which is 4 times of that of Falcon/Falcon-unzip (240 Kb).

Powered by Editorial Manager® and ProduXion Manager® from Aries Systems Corporation

	The comparison between SMARTdenovo and Falcon/Falcon-unzip assemblies showed that contigs assembled by SMARTdenovo had the assembly size of 473.04 Mb, N50 size of 1010.40 Kb and N90 size of 172.34 Kb; primary contigs assembled by Falcon had the assembly size of 475.28 Mb, N50 size of 241.14 Kb and N90 size of 54.29 Kb; alternate contigs assembled by Falcon had the assembly size of 54.10 Mb, N50 size of 43.88 Kb and N90 size of 22.68 Kb; primary contigs assembled by Falcon-unzip had the assembly size of 474.23 Mb, N50 size of 246.62 Kb and N90 size of 58.36 Kb; haplotigs assembled by Falcon-unzip had the assembly size of 173.15 Mb, N50 size of 48.98 Kb and N90 size of 17.44 Kb.<br><br>Considering that Hi-C contains extremely long-range linkage information, the larger contig length is an import factor for the success application of Hi-C data for scaffolding. Therefore, we adopted the SMARTdenovo contigs and then applied Hi-C to get a chromosomal-scale scaffold sequence.<br>To make the phasing information available to the public, we also uploaded the SMARTdenovo alternate sequences excluded from the reference haploid genome sequence, as well as the Falcon-unzip assembly of the apple snail, to the GigaDB and our institution's ftp-site, respectively.<br><br><br>3-) About the expansions found between the snail and L. fortunei, could you please describe the methodology used to consider genes expanded in these two groups? Was this done in a comparative manner with other species? Which ones? What was the criteria to consider gene families expanded?<br>Reply: we added the sentence at method part "To identify the common expanded gene families, we compared the P. canaliculata and L. fortunei with other seven species. The gene number of orthologous group in P. canaliculata and L. fortunei were two or more times than that in all of other species, respectively. Additionally, these gene families with P-value less than 0.01 were considered as expansion by z-test."<br><br><br>3a-) Have you identified CPYs expanded in both invasive species? I would suggest that L. fortunei should be included in figure 4.<br>Reply: We have identified the CYP genes in the L. fortunei in the revised manuscript, which were included in Figure 4a. There were 115 CYP genes found in L. fortunei, with no obvious expansion.
**Additional Information:**	
**Question**	**Response**
Are you submitting this manuscript to a special series or article collection?	No
**Experimental design and statistics**<br><br><br>Full details of the experimental design and statistical methods used should be given in the Methods section, as detailed in our Minimum Standards Reporting Checklist. Information essential to interpreting the data presented should be made available in the figure legends.<br><br><br>Have you included all the information requested in your manuscript?	Yes
**Resources**	Yes

A description of all resources used, including antibodies, cell lines, animals and software tools, with enough information to allow them to be uniquely identified, should be included in the Methods section. Authors are strongly encouraged to cite Research Resource Identifiers (RRIDs) for antibodies, model organisms and tools, where possible.<br><br>Have you included the information requested as detailed in our Minimum Standards Reporting Checklist?	
**Availability of data and materials**<br><br>All datasets and code on which the conclusions of the paper rely must be either included in your submission or deposited in publicly available repositories (where available and ethically appropriate), referencing such data using a unique identifier in the references and in the "Availability of Data and Materials" section of your manuscript.<br><br>Have you have met the above requirement as detailed in our Minimum Standards Reporting Checklist?	Yes

Manuscript

Click here to access/download;Manuscript;Snail_manuscript_revised-

Click here to view linked References

The genome of the golden apple snail *Pomacea canaliculata* provides insight into stress tolerance and invasive adaptation

Conghui Liu[1]*, Yan Zhang[1]*, Yuwei Ren[1]*, Hengchao Wang[1], Shuqu Li[1], Fan Jiang[1], Lijuan Yin[1], Xi Qiao[1], Guojie Zhang[2], Wanqiang Qian[1], Bo Liu[1]†, Wei Fan[1]†

[1]Agricultural Genomic Institute, Chinese Academy of Agricultural Sciences, Shenzhen, Guangdong, 518120, China.

[2]BGI-Shenzhen, Shenzhen, Guangdong, 518083, China

Conghui Liu: rapherlch@163.com; Yan Zhang: milrazhang@163.com; Yuwei Ren: xiaoshudaxia@126.com; Hengchao Wang: wanghengchao000@qq.com; Shuqu Li: lishuqu1234@163.com; Fan Jiang: greatjf@163.com; Lijuan Yin: yinlijuan1005@163.com; Xi Qiao: qiaoxi@caas.cn; Guojie Zhang: guojie.zhang@bio.ku.dk

*These authors contributed equally to this work.

†Correspondence should be addressed to Bo Liu (lb_bobo@aliyun.com) or Wei Fan (fanwei@caas.cn).

Abstract

Background: The golden apple snail (*Pomacea canaliculata*) is a fresh water snail listed among the top-100 worst invasive species worldwide and a noted agricultural and quarantine pest that causes great economic losses. It is characterized by fast growth, strong stress tolerance, a high reproduction rate, and adaptation to a broad range of environments.

Results: Here, we used long-read sequencing to produce a 440-Mb high-quality,

chromosome-level assembly of the *P. canaliculata* genome. In total, 50 Mb (11.4%) repeat sequences and 21,533 gene models were identified in the genome. The major findings of this study include the recent explosion of DNA/hAT-Charlie transposable elements (TEs), the expansion of the P450 gene family and the constitution of the cellular homeostasis system, which contributes to ecological plasticity in stress adaptation. In addition, the high transcriptional levels of perivitellin genes in the ovary and albumen gland promote the function of nutrient supply and defence ability in eggs. Furthermore, the gut metagenome also contains diverse genes for food digestion and xenobiotic degradation.

Conclusions: These findings collectively provide novel insights into the molecular mechanisms of the ecological plasticity and high invasiveness.

Keywords: golden apple snail, *Pomacea canaliculata*, genome, adaptive evolution, stress tolerance, P450, reproduction, perivitelline, metagenome

Background

The golden apple snail *Pomacea canaliculata* (family Ampullariidae, order Architaenioglossa) is a fresh water snail listed among the world's top 100 worst invasive species [1] and is considered an agricultural and quarantine pest worldwide [2]. Native to tropical and subtropical South America, *P. canaliculata* gradually spread to non-indigenous regions, such as Southeast and East Asia [3], Africa [4], North America [5], Oceania [6] and even Europe [7]. Its successful biological invasion was closely related to its polyphagous feeding habits [8],

voracious appetite [9], broad environmental adaptability [10] and rapid growth and high rate of reproduction [11]. In addition to its ecological impact, *P. canaliculata* ravages a wide range of crops, including grains, fruits and vegetables [12], causing severe economic losses each year as a result of yield loss, replanting cost and expenditures on control (https://www.cabi.org/isc/datasheet/68490). More seriously, *P. canaliculata* has been involved in the transmission of a fatal human disease, eosinophilic meningitis, which first appeared in East Asia where people frequently consume these snails [13]. During this pathophoresis, *P. canaliculata* acts as an important intermediate host of the pathogenic parasite *Angiostrongylus cantonensis*, and the range of infected regions is still expanding, creating a great challenge in terms of human health [14, 15].

Molluscs are a highly diverse group, second only to arthropods in species number [16], and their high biodiversity makes them an excellent model to address issues such as biogeography, adaptability and evolutionary processes [17]. The worldwide invasive species *P. canaliculata* provides valuable potential in these fields [18]. As a primitive circumtropical species, *P. canaliculata* possesses strong ecological plasticity with many advantages, including low-temperature resistance [19] and drought tolerance [20], which has contributed to its competitive success in resource acquisition. *P. canaliculata* has been reported to establish populations at temperatures ranging from 10 °C to 35 °C [19, 21]. Additionally, *P. canaliculata* tolerates heavy metal contamination. When living in contaminated water, the gill is enriched with a high concentration of heavy metals, and histopathological changes in the digestive tract are

detected; however, an extremely low mortality rate is observed [22]. The conspicuous colouration and neurotoxic lectin could confer a survival advantage on the eggs, defending the embryos against potential predators [23]. Moreover, an immune-neuroendocrine system can also be detected in *P. canaliculata*, as demonstrated by the existence of a specific immune memory after bacterial challenge [24, 25], broadening the study of invertebrate immunology.

The rich phenotypic and genetic diversity of molluscs makes them an excellent species group for addressing many important issues in evolution, ecology and function. However, the genomic resources on Mollusca are still insufficient compared with those of other close phyla, such as Arthropoda and Nematoda, and few molluscs can be employed as model organisms. *P. canaliculata*, however, possesses the potential to be a model organism among molluscs because of several inherent characteristics. For example, *P. canaliculata* is easy to acquire because it has a broad global distribution originating from a primarily circumtropical environment. Moreover, its high adaptability, rapid growth and efficient reproduction facilitate the cultivation of *P. canaliculata* in the laboratory.

In recent years, the genomic features of *P. canaliculata* have been increasingly studied. After the discovery of 14 pachytene bivalents in the karyotype [26], molecular markers were identified to investigate the genetic diversity of the *P. canaliculata* population, including 369 amplified fragment length polymorphism (AFLP) loci [27], 16,717 simple sequence repeats (SSR) [28, 29] and 15,412 single-nucleotide polymorphisms SNPs [30]. In addition, multiple transcriptome analyses have been

performed to investigate the adaptation, invasion and immune mechanisms of *P. canaliculata*. For instance, Sun et al. reported 128,436 unigenes based on a de novo assembly of Illumina reads [30]; transcriptome changes in response to heat stress and starving incubation were used to characterize its invasive and adaptive abilities [31, 32]; a transcriptome analysis comparing invasive *P. canaliculata* and indigenous *Cipangopaludina cathayensis* provided insights into biological invasion [29]; and 402 immune-related differentially expressed genes (DEGs) in response to lipopolysaccharide (LPyS) challenge were used to explore the mechanisms of defence against pathogens [33]. Furthermore, proteomics tools such as isobaric tags for relative and absolute quantitation (iTRAQ), and liquid chromatography-tandem mass spectrometry (LC-MS/MS) were also applied in the study of protein expression during estivation and oviposition [34,35], together providing plentiful omics- data for the functional analysis of *P. canaliculata*.

However, research at the whole-genome level in *P. canaliculata* still lags far behind that in other mollusc species due to the lack of a high-quality reference genome. Multiple draft genomes of molluscs have been published, including the genomes of the California sea hare [36], Pacific oyster [37], pearl oyster [38,39], owl limpet [40], California two-spot octopus [41], golden mussel [42], and *Biomphalaria* snails [43], greatly promoting research on mollusc genomics. In this study, we present a chromosome-level genome assembly of *P. canaliculata* with high-quality gene annotation, transcriptome data from several tissues and under various conditions, and metagenomic data from the intestinal tracts, all of which were then applied to study

the species-specific environmental adaptation characteristics, such as the cellular homeostasis system underlying strong stress and the colour and nutrient contents of the eggs. Our data will not only strengthen the understanding of the evolutionary mechanisms of molluscs and the molecular basis of biological invasion but also foster the development of approaches to control the invasion of *P. canaliculata* and provide a basis for interrupting the transmission of pathogenetic nematode parasites.

RESULTS

Complete genome assembly at the chromosome level

We generated 26.6 Gb (60.1 X) of PacBio SMRT raw reads with an average read length of 10.1 kb, and 291 Gb (652.4 X) of Illumina HiSeq paired-end reads with an average read length of 150-250 bp using DNA extracted from a single adult *P. canaliculata* (Table S1). The 24.4 Gb (55.4 X) of clean PacBio SMRT reads that passed quality filtering were assembled by smartdenovo (https://github.com/ruanjue/smartdenovo), resulting in an assembly of 1,234 raw contigs with a total length of 473.0 Mb and an N50 length of 1.0 Mb. After filtering of alternatively heterozygous contigs, the 745 resulting contigs with a total length of 440.1 Mb and an N50 length of 1.1 Mb were taken as the final contigs. Previous karyotype research has shown that the haploid *P. canaliculata* genome consists of 14 chromosomes [26]. Based on the Hi-C data, 439.5 Mb (99.9%) of final contigs were anchored and oriented into 14 large scaffolds, each corresponding to a natural chromosome (Figure 1a and Figure 1b), with the longest 45.4 Mb and the shortest

27.2 Mb. This assembly quality is much better than that of the other molluscan genomes published thus far (Table 1). In addition to the length and continuity of the assembled sequences, another important aspect for evaluating genome assembly is the ratio of genome coverage. With an estimated genome size of 446 Mb and genome heterozygosity between 1% and 2% based on the distribution of k-mer frequency [44] (Figure S1), ~98.6 % of the *P. canaliculata* genome has been assembled. To further confirm the accuracy and completeness of the assembly, we mapped the Illumina shotgun reads to the assembled reference genome. Significantly, 97% and 95% of the genome-derived and transcriptome-derived reads, respectively, could be aligned to the reference genome, suggesting no obvious bias in sequencing and assembly. Additionally, the mitochondrial genome of *P. canaliculata* was assembled as a single contig 15,707 bp in length, which has 99.9% sequence identity to the published mitochondrial genome (GenBank: KJ739609.1) (Figure S2). This high-quality reference genome provides a good foundation for gene annotation.

The protein-coding genes were predicted on the reference genome by EVM, integrating evidence from *de novo* prediction, transcriptome and homology data. In total, 21,533 gene models were predicted as the reference gene set, with coding regions spanning ~32.2 Mb (7.3 %) of the genome (Table 1 and Table S2). The distribution of CDS length in *P. canaliculata* is similar to that in closely related species (Figure 1c). Overall, 97.5% of the reference genes were supported by transcriptome data, and 98.0% of eukaryote core genes from OrthoDB (http://www.orthodb.org/) were identified in the reference gene set by BUSCO. These

results were comparable to those in other published molluscan genomes (Table 1). In functional annotation, a total of 19,815 (91.9 %) reference genes were annotated by at least one functional database. Specifically, 15,662 (72.7%), 13,769 (63.4%), 17,081 (79.3%), 18,847 (87.5%) and 17,003 (79.9%) reference genes were annotated with the eggNOG, KEGG, NR, InterPro and UniProt databases, respectively (Figure S3).

Signs of adaptive evolution in *P. canaliculata* genome

To gain insight into the evolutionary perspective of *P. canaliculata*, a phylogenetic tree was built based on 306 high-confidence single-copy orthologous genes from nine related species (*P. canaliculata*, *Lottia gigantea*, *Aplysia californica*, *Biomphalaria glabrata*, *Crassostrea gigas*, *Octopus bimaculoides*, *Pinctada fucata*, *Lingula anatina* and *Limnoperna fortunei*) by PhyML [45] and the divergence time was estimated using MCMCTree [46]. The results show that *P. canaliculata* diverged from the ancestor of *B. glabrata* and *A. californica* 372 million years ago (Mya) and from *L. gigantea* 491 Mya (Figure 2a).

Then, the molluscan orthologous genes were investigated for adaptive evolution. Utilizing pairwise protein sequence similarities, gene family clustering was conducted by orthoFinder [47]. A total of 239,541 reference genes from the nine species were clustered into 69,582 orthologous groups, among which 14,766 orthologous groups contained at least two genes each. We identified 66 orthologous groups that underwent common expansion in both *P. canaliculata* and *L. fortunei* but not the other seven species. The functions of these orthologous groups are mainly related to signal

transduction; replication and repair; translation, glycan biosynthesis and metabolism; lipid metabolism; and the endocrine, immune and nervous systems (Figure S4). These relations suggest that the gene families that underwent expansion may play important roles in adaptation to the environment as invasive species.

The high-coverage genome assembly enables a comprehensive analysis of the transposable elements (TEs), which play multiple roles in driving genome evolution in eukaryotes [48]. In total, we identified 49.6 Mb TE sequences in the assembled *P. canaliculata* genome (Table 1), including 3.4 Mb long terminal repeats (LTRs), 27.2 Mb long interspersed elements (LINEs), 17.5 Mb DNA transposons and 1.5 Mb short interspersed elements (SINEs). Next, we analysed the divergence rate of each class of TEs among the available sequenced mollusc genomes. Notably, the TE class of DNA transposons showed a specific peak at a divergence rate of ~4% divergence rate for *P. canaliculata* and *C. gigas* (Figure 2b), indicating a recent explosion of DNA transposons in these two species. We analysed the expression of 709 genes, including DNA elements restricted to the 4% peak inside the gene region, compared with that of the other genes outside the 4% peak (Figure S5). DEGs were defined here by P-values smaller than 0.05 for comparison of the treatment (heat, cold, heavy metal and air exposure) and control data. The percentages of DEGs in the 4% peak were higher than those of genes outside the peak (10.2% higher for heat, 8.6% higher for cold, 8.6% higher for heavy metal, and 7.3% higher for air exposure). Among the DEGs in the 4% peak, approximately half were up-regulated, and the other half were down-regulated. Moreover, the DEGs in the 4% peak were mainly enriched in cellular metabolic

process, response to stimulus, localization and signaling according to GO annotation. These results indicated that genes in the 4% peak were likely to be more active in the response to stimulus, promoting potential plasticity in stress adaptation. TEs are powerful facilitators of evolution that generate "evolutionary potential" to introduce small adaptive changes within a lineage, and the importance of TEs in stress responses and adaptation has been reported in numerous studies [49,50]. The recent explosion of DNA TEs in *P. canaliculata* could also play an important role in promoting the potential plasticity in stress adaptation.

Investigation of cellular homeostasis system underlying strong stress adaptation

The homeostasis system plays a crucial role in stress adaptability, providing the molecular basis for re-establishing dynamic equilibrium after challenges by various environmental stressors, including temperature, air exposure, anthropogenic pollution and pathogens [51]. In this study, we addressed three constituent parts of the cellular homeostasis system, which contributes to the successful ecological plasticity of *P. canaliculata* (Figure 3). The transcriptomes of the hemocytes after different stimuli (cold, heat, heavy metal and air exposure) were also sequenced and analysed to address the potential roles of these genes in the cellular homeostasis system.

The unfolded protein response (UPR) system is the central component of protein homeostasis [52]. Heat shock proteins (HSPs) act as molecular chaperones to maintain correct folding, and heat shock transcription factor 1 (HSF1) is responsible for the transcriptional induction of HSPs [53]. In the *P. canaliculata* genome, 13

HSP70s, 6 HSP90s, 7 HSP40s and 11 HSFs were identified (Table S3), and the expression of HSP90s and HSFs was highly induced in response to heat, cold, heavy metal and air exposure (Table S4 and Figure S6). Inositol-requiring protein 1 (IRE1), protein kinase RNA-like ER kinase (PERK), and activating transcription factor 6 (ATF6) are three mediators recruited by the endoplasmic reticulum (ER) to regulate the UPR [54]. We found putative coding genes of the three core mediators, their respective downstream transcription factors, and the corresponding recognition chaperones in the *P. canaliculata* genome (Table S3).

The xenobiotic biotransformation system helps the molluscs adapt to toxicants, especially pesticides in aquatic environments [55]. Manual annotation of this genome identified 157 cytochrome P450s (CYP450s), 15 flavin-containing monooxygenases (FMOs), 53 glutathione S-transferases (GSTs) and 105 ATP binding cassette (ABC) transporters, most of which showed up-regulated expression under stress (Table S3 and Table S4). These proteins have been shown to function in contaminant detection, conjugative modification and expulsion for xenobiotic detoxification [56-58].

The massive production of reactive oxygen species (ROS) and reactive oxygen intermediates (ROIs) induced by stress leads to many pathological conditions, and antioxidant systems protect the organism from superoxide [59]. Four main antioxidant enzyme classes, namely, superoxide dismutase (SOD), catalase (CAT), peroxidase (Prx), and glutathione peroxidase (GPX), were found in *P. canaliculata* and showed elevated global expression in response to stress (Table S3 and Table S4).

Apoptosis is a process of cell death when sensing stress and the regulation of

apoptosis contributes to the dynamic homeostasis of the internal environment. In *P. canaliculata*, we propose the existence of both intrinsic and extrinsic apoptotic signaling pathways, evidenced by the presence of homologous genes involved in both pathways. These two pathways could be activated by cytochrome C and tumour necrosis factor receptor (TNFR), respectively (Table S3). Inhibitors of apoptosis, such as XIAP, Bcl2 and Bak, are also detected and show increased expression in response to stress (Table S4), which is expected to delay the process of apoptosis and cell death in the stress response.

The expansion of the P450 gene family contribute to stress tolerance

Cytochrome P450 (CYP) enzymes are a monooxygenase family with highly diverse structures and functions that have been widely identified in all kingdoms of life [60]. P450s catalyse the reductive scission of molecular oxygen and are responsible for the synthesis and metabolism of various molecules, including drugs, hormones, antibiotics, pesticides, carcinogens and toxins [61]. The hormones they synthesize, such as glucocorticoids, mineralocorticoids, progestins, and sex hormones, are critical to stress response, growth and reproduction, and the endogenous and exogenous chemical metabolism participate in combatting toxic compounds [62].

We found that the *P. canaliculata* CYP gene family had undergone an expansion compared to that in the other molluscs. We identified 157 genes in the genome of *P. canaliculata* and 128, 102, 135, 115, 78, 52 and 94 genes in *A. californica, B. glabrata, C. gigas, L. fortunei, L. gigantea, O. bimaculoides* and *P. fucata* respectively,

using the same standard (Figure 4a). An expansive trend was also observed in comparison with other model species, such as *Homo sapiens* (57), *Mus musculus* (102), *Danio rerio* (94) and *Drosophila melanogaster* (94) [63]. Gene expansion was mainly found in the CYP2U and CYP3A sub-families, whereas fewer genes were expanded in CYP4F. In mammals, CYP2U participates in the metabolism of fatty acids to generate bioactive eicosanoid derivatives, potentially regulating the development of immune function [64]. In *P. canaliculata*, 40 genes formed the CYP2U clade, mainly expressed in the hepatopancreas (Figure 4b and Table S5_a, Table S5_b). CYP3A is a versatile enzyme that metabolizes a wide range of xenobiotics, and its production promotes the growth of various cell types [65]. The 56 CYP3A genes are comprehensively expressed in the hepatopancreas, gill and kidney (Figure 4b and Table S5_a, Table S5_b). CYP4F possesses epoxygenase activity, metabolizing fatty acids to epoxides to suppress hypertension, pain perception and inflammation [66]. Twenty genes were identified in CYP4F, and Pc06G011748, Pc06G011460, Pc06G011458, Pc06G011459, Pc04G006708, Pc04G006710 and Pc04G006707 exhibited highly induced expression levels under cold, heat, heavy metal and air exposure stress, indicating their critical roles in the stress tolerance (Figure 4b, Table S5_a and Table S5_b).

The identification of perivitellin genes and their high transcriptional levels in the ovary and albumen gland

P. canaliculata has eggs characterized by abundant nutrients, reddish or pinkish colour, aerial oviposition and neurotoxicity [23, 67] due to the perivitelline Fluid

(PVF), which fills the space between the eggshell and the embryo and consists of carbohydrates, lipids and proteins (Figure 5a). The PVF proteins in *P. canaliculata*, include three major components, PcOvo, PcPV2, and PcPV3 [68], collectively named perivitellins, which make up 90% of the total proteins, whereas most of the other dozens of low-abundance components each account for less than 1% of the total proteins [35]. The perivitellins are not only responsible for the major supply of materials and energy during embryogenesis but also provide warning pigments and deadly toxicants against predators [23, 69, 70].

We identified 28 candidate PVF genes in *P. canaliculata* by mapping each of the 59 fragmental PVF protein sequences derived from a previous proteomics study by Sun [35] to its best hit in the reference gene set of *P. canaliculata*, using BLASTP with requirements of over 85% identity and at least 50% alignment length (Table S6). Then, the functional annotation of those fragmental proteins was also transferred to our identified PVF genes. The transcriptome data show that 22 (79%) of the 28 candidate PVF genes exhibit their highest expression in the ovary and albumen gland (PVF protein synthesis factory) among all 7 tissues (Figure 5b and Table S7), confirming that most of them are genuine functional PVF genes. Six of these 28 candidate PVF genes are perivitellin genes, including two PcOvo genes, Pc09G015543 (PcOvo2) and Pc09G015548 (PcOvo3); two PcPV2 genes, Pc07G012572 (PcPV2-31) and Pc07G012571 (PcPV2-67); and two possible PcPV3 genes, Pc09G015546 and Pc09G015547. The expression levels of these 6 genes in the ovary and albumen gland are much higher than those of the other 22 candidate PVF genes.

By analysing the orthoFinder gene families that include orthologous and paralogous genes from *P. canaliculata* and 8 other sequenced mollusc species, we found that these 28 candidate PVF genes were classified into 20 multiple-gene families (⩾2 genes) and 7 single-gene families (only one gene) (Table S8). Notably, 5 of the 6 perivitellin genes were classified into single-gene families, except for Pc07G012571 (PcPV2-67), which not only has homologous genes in other mollusc species but also has three paralogous genes in *P. canaliculata* itself. However, none of these three PcPV2-67 paralogous genes in *P. canaliculata* showed higher expression in the ovary and albumen gland than in other tissues, indicating that they are likely not PVF-related genes, i.e., only Pc07G012571 plays a role in PVF. The nearly unique and single-copy nature of the 6 perivitellin genes in *P. canaliculata*, may be explained by the long evolutionary distance, over 200 Mya for *P. canaliculata* and its most closely related species, *A. californica*, as well as numerous differences in their living characteristics and egg structures. Another possible explanation is that these 6 major PVF genes may have experienced rapid evolution in their history to adapt to the changing environment.

The gut microbiome plays important roles in stress resistance and food digestion

The gut microbiome is regarded as the “second genome” of the host animal, due to the fact that gut microbiota contributes to the food digestion, immune system development, and many other processes important to the host. To investigate the relationship between the gut microbiome and the invasive lifestyle of *P. canaliculata*,

we collected gut digesta samples from 70 *P. canaliculata* snails and generated 31 Gb of high-quality metagenomic data on the Illumina HiseqX10 platform. To our knowledge, this study is the first in-depth sequencing of the snail gut microbiome. A total of 1,142,095 non-redundant genes were obtained with an average open reading frame (ORF) length of 604 bp (Table S9). The taxonomic composition analysis showed that, at the phylum level, Proteobacteria was predominant, followed by Verrucomicrobia, Bacteroidetes, Firmicutes, Spirochaetes, Actinobacteria, etc. (Table S10_a). At the genus level, the most abundant genera included *Aeromonas*, *Enterobacter*, *Desulfovibrio*, *Citrobacter*, *Comamonas*, *Klebsiella* and *Pseudomonas* (Table S10_b), most of which were also present in *Achatina fulica* [71,72].

Interestingly, some of the most abundant genera, such as *Desulfovibrio*, *Citrobacter* and *Pseudomonas*, were reported as having strong abilities to remove heavy metals by bioprecipitation and bioabsorption [73-75]. For example, the sulfur-reducing bacteria *Desulfovibrio* produce H_2S, which precipitates metals and therefore reduces the toxic effects of dissolved metals [73]. Based on the KEGG pathway database, the complete sulfate reduction metabolism pathway was identified in the *P. canaliculata* gut microbiome. We suggested that these gut microbes might help *P. canaliculata* survive the environmental stress of heavy metals in harsh conditions. In addition, a large number of genes in xenobiotic biodegradation and metabolism pathways were annotated, corresponding to 288 KEGG orthologous groups (KOs) and 21 pathways (Table S11). As many of the pathways, such as benzoate degradation, toluene degradation, xylene degradation and steroid degradation, could not be identified in the

host genome through KO analysis, we suggested that microbial detoxification abilities may contribute to the ability *P. canaliculata* to resist stresses caused by xenobiotics such as pesticides and environmental pollutants.

In digestion, the gut microbes are directly involved in the breakdown of the cellulose portion of the diet, and previous studies have isolated cellulolytic bacteria and evaluated the cellulolytic enzyme activities [76]. Our work found a broader range of carbohydrate active enzymes (CAZymes). Of the 208 annotated CAZyme families, 99 were glycoside hydrolase (GH) families (Table S12). Enzymes that could be classified as cellulases, endohemicelluloses, debranching enzymes, and oligosaccharide-degrading enzymes were all identified. These findings indicate that the gut microbiome provides assistance in digesting a broad range of food sources, enabling *P. canaliculata* to grow rapidly and adapt to an invasive lifestyle.

Conclusion

Given its environmental invasiveness, broad stress adaptability and rapid reproduction, the golden apple snail *P. canaliculata* has received a vast amount of attention worldwide. However, the underlying genetic mechanisms of these properties have not been comprehensively uncovered. The chromosome-level genome of *P. canaliculata* presented in this study sheds the first light on into the genomic basis of its ecological plasticity in response to various stressors. The major findings of this study include the recent explosion of DNA/hAT-Charlie TEs, the expansion of the P450 gene family and the constitution of the cellular homeostasis system, all of which contribute to the

plasticity of the organism in stress adaptation. Although the function of the recently originated TEs could not be confirmed, TEs are considered powerful facilitators in adaptive evolution, suggesting that their increased number plays an important role in the stress resistance of *P. canaliculata*. The UPR system, xenobiotic biotransformation system and ROS system are all major components of the cellular homeostasis system, and the P450s in particular underwent expansion with specific functions. In addition, exclusive perivitellin genes were identified in the *P. canaliculata* genome, and they are believed to contribute to the high reproductive rate and the expansion of habitats. Furthermore, the gut metagenome contains diverse genes for food digestion and xenobiotic degradation. These findings collectively provide novel insight into the molecular mechanisms of ecological plasticity and high invasiveness.

In this study, we report a fine reference genome of *P. canaliculata*, first chromosome-level Mollusca genome published. With widespread distribution, rapid growth and efficient reproduction, *P. canaliculata* possesses the potential to be a model organism of Mollusca. As its cellular complexity and conservation of pathways also make *P. canaliculata* a useful representative of Mollusca, the genome described in this study can be used to advance our understanding of the molecular mechanisms involved in various scientific questions regarding Mollusca.

Methods

Samples collection and sequencing

Adults of *P. canaliculata* were collected from a local paddy field in Shenzhen,

Guangdong province, China, and maintained in aerated freshwater at 15 ± 2 °C for a week before processing. Genomic DNA was extracted from the foot muscles of a single *P. canaliculata* for constructing PCR free Illumina 350-bp insert libraries and PacBio 20-kb insert library, and sequenced on Illumina HiSeq 2500 and PacBio SMRT platforms, respectively. The Hi-C library was prepared using the muscle tissue of another single *P. canaliculata* by following methods: Nuclear DNA was cross-linked in situ, extracted, and then digested with a restriction enzyme. The sticky ends of the digested fragments were biotinylated, diluted, and then ligated to each other randomly. Biotinylated DNA fragments were enriched and sheared again for preparing the sequencing library, which was then sequenced on a HiSeq X Ten platform (Illumina).

Seven tissues including embryos (2 days post fertilization), gill, hemocytes, hepatopancreas, kidney, ovary and albumen gland and testis from six animals were collected as parallel samples. Next, animals were cultivated in 37 °C and 10 °C for 24 hours heat and cold tolerance, in Cr^{3+}(2mg L^{-1}), Cu^{2+}(0.2mg L^{-1}) and Pb^{2+}(1mg L^{-1}) for 24 hours heavy metal tolerance, and in waterless tank for 7 days air exposure. Then the hemocytes were harvested and stored, with three replicates for each group. In final, total RNAs were extracted from the stored tissues of *P. canaliculata* materials, and then mRNAs were pulled out by beads with poly-T for constructing cDNA libraries (insert 350-bp), and sequenced on an Illumina HiSeq 2500 sequencer.

The intestinal digesta from 70 adult snails of *P. canaliculata* were collected, pooled into 6 samples and stored at −20 °C until microbial DNA was extracted. A

combination of cell lysis treatments was applied, including five freeze-thaw cycles (alternating between 65 °C and liquid nitrogen for 5 min), repeated beads-beating in ASL buffer (cat. no. 19082; Qiagen Inc.), and incubated at 95 °C for 15 min. DNA was isolated following the protocol reported protocol [77]. Paired-end libraries of metagenomic DNA were prepared with an insert size of 350 base pairs (bp) following the manufacture's protocol (cat. no. E7645L; New England Biolabs). Sequencing was performed on Illumina HiSeq X10.

Genome assembly and annotation

The Illumina raw reads were filtered by trimming the adapter sequence and low-quality regions (https://github.com/fanagislab/common_use), resulting in clean and high-quality reads with an average error rate < 0.001. For the PacBio raw data, the short subreads (< 2 kb) and low-quality (error rate > 0.2) subreads were filtered out, and only one representative subread was retained for each PacBio read. The clean PacBio reads were assembled by the software smartdenovo (https://github.com/ruanjue/smartdenovo), after which Illumina reads were aligned to the contigs by BWA-MEM (BWA, RRID:SCR_010910), and single base errors in the contigs were corrected by Pilon v1.16 (Pilon, RRID:SCR_014731) with the parameters "-fix bases, -nonpf, -minqual 20". The *P. canaliculata* genome is highly heterozygous, as illustrated by the double peaks on the distribution curve of k-mer frequency, and the current assembly algorithm tends to collapse homozygous regions and report heterozygous regions in alternative contigs. To obtain a haploid reference

contigs, we employed a whole-genome alignment (WGA) strategy with MUMmer v3.23 to recognize and selectively remove alternative heterozygous contigs, which were characterized by shorter length (less than 200 kb) and the ability of most regions (more than 50%) to be aligned to another larger contig with confident identity (higher than 80%). Next, Hi-C sequencing data were aligned to the haploid reference contigs by BWA-MEM, and then these contigs were clustered into chromosomes with LACH-ESIS (http://shendurelab.github.io/LACHESIS/).

A de novo repeat library for *P. canaliculata* was constructed by RepeatModeler v. 1.0.4 (RepeatModeler, RRID:SCR_015027; http://www.repeatmasker.org/RepeatModeler.html). TEs in the *P. canaliculata* genome were also identified by RepeatMasker v4.0.6 (RepeatMasker, RRID:SCR_012954; http://www.repeatmasker.org/) using both the Repbase library and the de novo library. Tandem repeats in the *P. canaliculata* genome were predicted using Tandem Repeats Finder v4.07b [78]. The divergence rates of TEs were calculated between the identified TE elements in the genome and their consensus sequence at the TE family level.

The gene models in the *P. canaliculata* genome were predicted by EVidence Modeler v1.1.1 [79], integrating evidence from ab initio predictions, homology-based searches and RNA-seq alignments. Then, these gene models were annotated by RNA-seq data, UniProt database and InterProScan software (InterProScan, RRID:SCR_005829) [80]. Finally, the gene models were retained if they had at least one piece of supporting evidence from the UniProt database, InterProScan domain and RNA-seq data. Gene

functional annotation was performed by aligning the protein sequences to the NCBI NR, UniProt, COG and KEGG databases with BLASTP v2.3.0+ under an E-value cutoff of 10^{-5} and choosing the best hit. Pathway analysis and functional classification were conducted based on the KEGG database [81]. InterProScan was used to assign preliminary GO terms, Pfam domains and IPR domains to the gene models.

Evolutionary analysis

Orthologous and paralogous groups were assigned from seven species (*P. canaliculata, Lottia gigantea, Aplysia californica, Biomphalaria glabrata, Crassostrea gigas, Octopus bimaculoides, Pinctada fucata, Limnoperna fortunei* and *Lingula anatina*) by OrthoFinder [47] with default parameters. Orthologous groups that contained only one gene for each species were selected to construct the phylogenetic tree. The protein sequences of each gene family were independently aligned by muscle v3.8.31 [82] and then concatenated into one super-sequence. The phylogenetic tree was constructed by maximum likelihood (ML) using PhyML v3.0 (PhyML, RRID:SCR_014629) [45] with the best-fit model (LG+I+G) estimated by ProtTest3 [83]. The Bayesian relaxed molecular clock (BRMC) approach was adopted to estimate the neutral evolutionary rate and species divergence time using the program MCMCTree, implemented in the PAML v4.9 package (PAML, RRID:SCR_014932) [46]. The tree was calibrated with the following time frames to constrain the age of the nodes between the species: minimum = 260 Ma and maximum = 290 Ma for *P. fucata* and *C. gigas* [84];

minimum = 450 Ma and maximum = 480 Ma for *A. californica* (or *B. glabrata*) and *L. gigantea* [85]. The calibration time (fossil record time) interval (550-610 Mya) of *O. bimaculoides* was adopted from previous results [86]. To identify the common expanded gene families, we compared the *P. canaliculata* and *L. fortunei* with other seven species. The gene number of orthologous group in *P. canaliculata* and *L. fortunei* were two or more times than that in all of other species, respectively. Additionally, these gene families with P-value less than 0.01 were considered as expansion by z-test.

Transcriptome data analysis

Transcriptome reads were trimmed with the same method for genomic reads (https://github.com/fanagislab/common_use), and then mapped to the reference genome of *P. canaliculata* using TopHat v. 2.1.0 (TopHat, RRID:SCR_013035) with default settings. The expression level of each reference gene in terms of FPKM was computed by cufflinks v2.2.1 (cufflinks, RRID:SCR_014597). A gene was considered to be expressed if its FPKM > 0. Differential gene expression analysis was conducted using cuffdiff v2.2.1.

Metagenome data analysis

The Illumina raw reads were filtered by trimming the adapter sequence and low-quality regions (https://github.com/fanagislab/common_use), resulting in high-quality reads with an average error rate < 0.001. Then, the reads mapped to the following genomes by BWA-MEM were filtered out

(https://github.com/fanagislab/metagenome_analysis.git)[87], to exclude the contaminated host, food, parasite, and human DNA sequences. The genomes include: the *P. canaliculata* genome, the *Brassica rapa* genome, the *Oryza sativa* genome, 2 *Angiostrongylus cantonensis* genomes, the *Caenorhabditis elegans* genome, the *Schistosoma mansoni* genome, the *Clonorchis sinensis* genome, the *Fasciola hepatica* genome, the *Danio rerio* genome, and the *human hg38* genome. Finally, short reads (length < 75 bp) and unpaired reads were excluded to form a set of clean reads.

The clean reads were assembled by metaSPAdes (v3.11.1) [88] in paired-end mode for each sample. Then, gene prediction was performed on contigs longer than 500 bp by Prodigal v2.6.3 (Prodigal, RRID:SCR_011936) [89] with the parameter "-p meta", and gene models with cds length less than 102 bp were filtered out. A non-redundant (NR) gene set (539,344 genes) was constructed using the gene models predicted from each sample by cd-hit-est (v4.6.6) [90] with the parameter "-c 0.95 -n 10 -G 0 –a S 0.9", which adopts a greedy incremental clustering algorithm and the criteria of identity > 95% and overlap > 90% of the shorter genes. Then, the clean reads were mapped onto this NR gene set by BWA-MEM with the criteria of alignment length ⩾ 50 bp and identity > 95%. The unmapped reads from all samples were assembled together, and the genes were predicted again. The newly predicted genes were combined with the previous gene set by cd-hit-est to obtain a new NR gene set (1,147,339 genes). After the taxonomic assignments to the new NR gene set, 5244 genes classified as Eukaryota but not fungi were removed, and the final NR gene set (1,142,095 genes) was obtained.

The taxonomic assignments of the final NR genes were made on the basis of DIAMOND (DIAMOND, RRID:SCR_016071) [91] protein alignment against the NCBI -NR database by CARMA3 [92]. Functional annotation was performed by aligning all the protein sequences to the KEGG [93] database (release 79) using DIAMOND and taking the best hit with the criteria of E-value < 1e-5. CAZymes were annotated with dbCAN (release 5.0) [94] using Hmmer v3.0 hmmscan (Hmmer, RRID:SCR_005305) [95] by taking the best hit with an E-value < 1e-18 and coverage > 0.35.

The clean reads from each sample were aligned against the gene catalogue (1,142,095 genes) by BWA-MEM with the criteria of alignment length ⩾ 50 bp and identity > 95%. Sequence-based gene abundance profiling was performed as previously described [96]. The taxonomic profiles of the samples were calculated by summing the gene abundance according to the taxonomic assignment result.

Abbreviations

A. californica, Aplysia californica; B. glabrata, Biomphalaria glabrata; C. gigas, Crassostrea gigas; O. bimaculoides, Octopus bimaculoides; L. anatina, Lingula anatina; L. fortunei, Limnoperna fortunei; L. gigantea, Lottia gigantea; P. canaliculata, Pomacea canaliculata; P. fucata, Pinctada fucata; Hem, hemocytes; Te, testis; Ov, ovary and albumen gland; Kn, kidney; GI, gill; Hp, hepatopancreas, Em, embryo; SSR, simple sequence repeats; mya, million years ago*; BLAST, basic local alignment search tool;* SNP, single nucleotide polymorphism; PVF, Pervitelline Fluid;

Ovo, ovorubin; AFLP, amplified fragment length polymorphism; DEGs, differentially expressed genes; LPyS, Lipopolysaccharide; iTRAQ, Isobaric Tags For Relative, Absolute Quantitation; LC-MS/MS, Liquid Chromatography-tandem Mass Spectrometry; TEs, transposable elements; LTR, long terminal repeats; LINE, long interspersed elements; SINE, short interspersed elements; UPR, Unfolded protein response; HSPs, heat shock proteins; HSF1, heat shock transcription factor 1; PERK, protein kinase RNA-like ER kinase; ATF6,activating transcription factor 6; ER, endoplasmic reticulum; CYP450s, cytochrome P450s; FMOs, flavin-containing monooxygenases; GSTs, glutathione S-transferases; ABC, ATP binding cassette; ROS, reactive oxygen species; ROI, reactive oxygen intermediates; SOD, superoxide dismutase; CAT, catalase; Prx, peroxidase; GPX, glutathione peroxidase; TNFR, tumor necrosis factor receptor; NR, non-redundant genes; ORF, open reading frame; Kos, orthologous groups; CAZymes, carbohydrate active enzymes; GH, Glycoside Hydrolase.

Availability of data and materials

Tables S1 to S12 and Figures S1 to S6 are available in the supplementary information file. The raw sequencing data has been deposited in DDBJ/EMBL/GenBank under project accession PRJNA427478, SRR6425828 for genomic Illumina_PE125 sequencing data, SRR6425829 for genomic Illumina_PE150 sequencing data, SRR6425827 for genomic PacBio sequencing data, SRR6429132~SRR6429164 for transcriptome sequencing data, and SRR6472920~SRR6472925 for gut microbiome

data. Other supporting data, including genome assemblies, annotations, phylogenetic tree files and BUSCO results, are available via the *GigaScience* repository GigaDB [97].

Authors' contributions

WF, WQ and CL conceived the study and designed the experiments. CL performed the genome sequencing and assembly, BL performed annotation and evolutionary analysis. CL performed the stress tolerance analysis, YR performed the reproduction analysis, YZ performed the metagenome analysis. HW, SL, FJ, LY, XQ provided suggestions and helped checking. CL, WF, BL, YR, YZ wrote the manuscript, and GZ helped revising the manuscript. All authors read and approved the final manuscript.

Competing interests

The authors declare that they have no competing interests.

Acknowledgements

This project is supported by the National key research and development program of China (2016YFC1200600), Shenzhen science and technology program (JCYJ20150630165133395), Fund of Key Laboratory of Shenzhen (ZDSYS20141118170111640), and The Agricultural Science and Technology Innovation Program (ASTIP) of Chinese Academy of Agricultural Sciences(CAAS) &

Elite Youth Program of Chinese Academy of Agricultural Sciences. We thank Fanghao Wan, Jue Ruan, Yutao Xiao for providing constructive suggestions to this project.

Legends of tables and figures

Tables

Table 1. Summary of assembly and annotation of mollusc genomes

**Genome feature**	***P. canaliculata***	***L. gigantea***	***A. californica***	***B. glabrata***	***C. gigas***	***O. bimaculoides***
Assembled sequences (bp)	440,071,717	359,505,668	927,310,431	916,377,450	557,735,934	23,381,887,882
Contig N50 size (bp)	1,072,857	94,165	9,817	18,978	37,218	5,982
Contig N90 size (bp)	303,904	10,180	1,626	5,132	11,109	1,606
Scaffold N50 size (bp)	31,531,291	1,870,055	917,541	48,059	401,685	475,182
Scaffold N90 size (bp)	23,662,357	74,480	207,390	817	68,181	79,088
GC content (%)	40.3	33.3	40.3	36.0	33.4	36
No. of gene models	21,533	23,824	19,909	14,224	28,402	15,814
Avg. CDS length (bp)	1,497	1,136	1,568	1,066	1,472	1,535
BUSCO (%)	98.9	98.4	98.7	72.8	99.4	98.7
Transposable elements (bp)	49,579,006	37,369,817	202,174,499	189,550,886	103,381,274	737,398,096
Tandem repeat (bp)	873,801	257,674	8,263,822	2,145,821	590,907	62,633,792

Figures

Figure 1. The genome characteristics of *P. canaliculata*. (a) Circos plot showing the genomic features**.** Track 1: 14 linkage groups of the genome; Track 2: distribution of transposon elements in chromosomes; Track 3: protein-coding genes located on chromosomes; Track 4: distribution of GC contents. **(b)** A genome-wide contact matrix from Hi-C data between each pair of the 14 chromosomes using a 100 kb window size. The colour value indicates the base 2 logarithm of the number of valid reads ($\log_2$(valid reads)). **(c)** Distribution of CDS length in six closely related species.

Figure 2. Evolutionary genomic analysis of *P. canaliculata*. (a) Phylogenetic placement of *P. canaliculata* within the dated tree of molluscs. The estimated divergence time is shown at each branching point, and *P. canaliculata* is shown in red. **(b)** Distribution of divergence rate for the class of DNA transposons in molluscs genomes. The divergence rate was calculated by comparing all TE sequences identified in the genome to the corresponding consensus sequence in each TE subfamily. The red arrow indicates that *P. canaliculata* and *C. gigas* had a recent explosion of TEs at a divergence rate of ~4%.

Figure 3. The cellular homeostasis system in *P. canaliculata*. The unfolded protein response (UPR) system includes HSPs and HSF in the heat shock response and CNX, NEF, GRP94, BIP, HSP40, ATF6, IRE1, PERK, COP2, XBP, ATF4, TRAM and Derlin in the endoplasmic reticulum unfolded protein response (UPR-ERAD). Apoptotic pathways include XIAPs, Bcl2, caspases, TNFR, and FADD. The antioxidant systems include PRX, SOD, CAT and GPX. The xenobiotic biotransformation system includes EPHX3, P450, FMO and ABC transporter. The colours of the boxes for gene families represent the degree of upregulation (FPKM-stimulus/FPKM-control) as an overall result of stress, including heat, cold, heavy metal and air exposure. Pathways and genes were obtained based on KEGG annotation.

Figure 4. The expansion of the P450 gene family in *P. canaliculata*. (a) Phylogenetic tree demonstrating orthologous and paralogous relationships of all P450 genes from eight species including *P. canaliculata*, *A. californica*, *B. glabrata*, *C.*

gigas, *L. fortunei*, *L. gigantea*, *O. bimaculoides* and *P. fucata*. P450 genes from eight species were obtained based on Pfam annotation (Interpro) with an E-value of 10^{-5}. Clades are labeled by P450 subfamily names. The tree was constructed using the maximum likelihood method in MEGA7, and the branch length scale indicates the average number of residue substitutions per site. (b) Phylogenetic tree of P450 genes in *P. canaliculata*, which is a subset of the phylogenetic tree for the 7 species, and their heat map of expression (FPKM) in seven tissues (Hem, hemocytes; Te, testis; Ov, ovary and albumen gland; Kn, kidney; Gl, gill; Hp, hepatopancreas; Em, embryo) and heat map of induced expression (FPKM-stimulus/FPKM-control) under stress (Con: control; heat; cold; Hm: heavy metal; Exp: air exposure).

Figure 5. The composition and expression of the *P. canaliculata* perivitellins in different tissues. (a) Perivitelline fluid (PVF) lies under the eggshell and surrounds the embryo. It contains carbohydrates, lipids, and proteins. The proteins are also known as perivitellins and are classified into three categories, PcOvo, PcPV2, and PcPV3. (b) The displayed expression value of PVF proteins is the base 10 logarithm of FPKM (log_{10}FPKM). The genes marked in red encode perivitellins. The 7 tissues examined are abbreviated as follows: Hem, hemocytes; Te, testis; Ov, ovary and albumen gland; Kn, kidney; Gl, gill; Hp, hepatopancreas; Em, embryo.

References

1. Lowe S, Browne M, Boudjelas S, de Poorter M. 100 of the World's Worst Invasive Alien Species: A selection fromthe Global Invasive Species Database. Auckland, New Zeland:

World Conservation Union (IUCN); 2000.

2. Ranamukhaarachchi SL, Wickramasinghe S. Golden apple snails in the world: introduction, impact, and control measures. Global advances in ecology and management of golden apple snails. 2006:133-52.

3. Naylor R. Invasions in Agriculture: Assessing the Cost of the Golden Apple Snail in Asia. Royal Swedish Academy of Sciences. 1996;25:443-8.

4. Berthold T. Vergleichende Anatomie, Phylogenie und historische Biogeographie der Ampullariidae: (Mollusca, Gastropoda). 1991.

5. Howells RG, Burlakova LE, Karatayev AY, Marfurt RK, Burks RL. Native and introduced Ampullaridae in North America: History, status, and ecology. 2006:73-112.

6. Halwart M, Bartley DM. International mechanisms for the control and responsible use of alien species in aquatic ecosystems, with special reference to the golden apple snail. Los Baños, Philippines: Philippine Rice Research Institute (PhilRice); 2006.

7. López MA, Altaba CR, Andree KB, López V. First invasion of the Apple snail Pomacea insularum in Europe. Tentacle. 2010;18:26-8.

8. Estebenet AL, Martín PR. Pomacea canaliculata (Gastropoda: Ampullariidae): life-history traits and their plasticity. Biocell 2002;26:83-9.

9. Lach L. The spread of the introduced freshwater apple snail *Pomacea canaliculata* (Lamarck) (Gastropoda Ampullariidae) on Oahu, Hawaii. Bishop Museum Occasional Papers. 1999;58:66-71.

10. Yusa Y，Sugiura N, Wada T. Predatory Potential of Freshwater Animals on an Invasive Agricultural Pest, the Apple Snail *Pomacea canaliculata* (Gastropoda: Ampullariidae), in

Southern Japan. Biol Invasions. 2006;8:137-47.

11. Lach L, Britton DK, Rundell RJ, Cowie RH. Food Preference and Reproductive Plasticity in an Invasive Freshwater Snail. Biol Invasions. 2000;2:279-88.

12. Mochida O. Spread of freshwater *Pomacea* snails (Pilidae, Mollusca) from Argentina to Asia. Micronesica. 1991;3 51-62.

13. Shan L, Zhang Y, Steinmann P, Zhou X. Emerging Angiostrongyliasis in Mainland China. Emerg Infect Dis. 2008;14:161-4.

14. Caldeira RL, Mendonca CL, Goveia CO, Lenzi HL, Graeff-TeixeiraC Lima WS, et al. First record of molluscs naturally infected with *Angiostrongylus cantonensis* (Chen, 1935) (Nematoda: Metastrongylidae) in Brazil. Memórias do Instituto Oswaldo Cruz. 2007;102:887-9.

15. McMichael AJ, Beaglehole R. The changing global context of public health. Lancet (London, England). 2000;356:495-9.

16. Chapman A. Numbers of Living Species in Australia and the World. Australian Biological Resources Study; 2009.

17. Lindberg DR, Ponder WF, Haszprunar G. The Mollusca: relationships and patterns from their first half-billion years. Oxford University Press, Oxford; 2004.

18. Hayes KA, Cowie RH, Thiengo SC. A global phylogeny of apple snails: Gondwanan origin, generic relationships, and the influence of outgroup choice (Caenogastropoda: Ampullariidae). Biol J Linn Soc Lond. 2009;98:61-76.

19. Matsukura K, Tsumuki H,Izumi Y, Wada T. Physiological response to low temperature in the freshwater apple snail, *Pomacea canaliculata* (Gastropoda: Ampullariidae). J Exp

Biol. 2009;212:2558-63.

20. Yusa Y, Wada T, Takahashi S. Effects of dormant duration, body size, self-burial and water condition on the long-term survival of the apple snail, *Pomacea canaliculata* (Gastropoda: Ampullariidae). Appl Entomol Zool. 2006;41:627-32.

21. Seuffert ME, Burela S, Martín PR. Influence of water temperature on the activity of the freshwater snail Pomacea canaliculata (Caenogastropoda: Ampullariidae) at its southernmost limit (Southern Pampas, Argentina). Journal of Thermal Biology. 2010; 35:77-84.

22. Kruatrachue M, Sumritdee C, Pokethitiyook P, Singhakaew S. Histopathological effects of contaminated sediments on golden apple snail (*Pomacea canaliculata*, Lamarck 1822). Bull Environ Contam Toxicol. 2011;86:610-4.

23. Dreon MS, Frassa MV, Ceolín M, Ituarte S, Qiu JW, Sun J, et al. Novel animal defenses against predation: a snail egg neurotoxin combining lectin and pore-forming chains that resembles plant defense and bacteria attack toxins. PLoS One. 2013;8:e63782. doi:10.1371/journal.pone.0063782.

24. Ottaviani E, Caselgrandi E, Fontanili P, Franceschi C. Evolution, immune responses and stress: studies on molluscan cells. Acta Biol Hung. 1992;43:293-8.

25. Ottaviani E, Accorsi A, Rigillo G, Malagoli D, Blom JM, Tascedda F. Epigenetic modification in neurons of the mollusc *Pomacea canaliculata* after immune challenge. Brain Res. 2013;1537:18-26.

26. Mercado Laczkó AC, Lopretto EC. Estudio cromosómico y cariotípico de p*omacea canaliculata* (Lamarck, 1801) (Gastropoda, Ampullariidae). Revista del Museo Argentino

de Ciencias Naturales "Bernardino Rivadavia" Hidrobiología. 1998;8:15-20.

27. Xu J, Han X, Li N, Yu J, Qian C, Bao Z. Analysis of genetic diversity of three geographic populations of *Pomacea canaliculata* by AFLP. Acta Ecol Sin. 2009;29:4119- 26.

28. Chen L, Xu H, Li H, Wu J, Ding H, Liu Y. Isolation and characterization of sixteen polymorphic microsatellite loci in the golden apple snail *Pomacea canaliculata*. Int J Mol Sci. 2011;12:5993-8.

29. Mu X, Hou G, Song H, Xu P, Luo D, Gu D, et al. Transcriptome analysis between invasive *Pomacea canaliculata* and indigenous *Cipangopaludina cahayensis* reveals genomic divergence and diagnostic microsatellite/SSR markers. BMC Genet. 2015;16:12.

30. Sun J, Wang M, Wang H, Zhang H, Zhang X, Thiyagarajan V, et al. De novo assembly of the transcriptome of an invasive snail and its multiple ecological applications. Mol Ecol Resour. 2012;12:1133-44.

31. Mu H, Sun J , Fang L, Luan T, Williams GA, Cheung SG, et al. Genetic Basis of Differential Heat Resistance between Two Species of Congeneric Freshwater Snails: Insights from Quantitative Proteomics and Base Substitution Rate Analysis. J Proteome Res. 2015;14:4296-308.

32. Yang L, Cheng TY, Zhao FY. Comparative profiling of hepatopancreas transcriptomes in satiated and starving *Pomacea canaliculata*. BMC Genet. 2017;18:18.

33. Xiong YM, Yan ZH, Zhang JE, Li HY. Analysis of albumen gland proteins suggests survival strategies of developing embryos of *Pomacea canaliculata*. Molluscan Res. 2017:1-6.

34. Sun J, Mu H , Zhang H, Chandramouli KH, Qian PY, Wong CK, et al. Understanding the

regulation of estivation in a freshwater snail through iTRAQ-based comparative proteomics. J Proteome res. 2013;12:5271-80.

35. Sun J, Zhang H, Wang H, Heras H, Dreon MS, Ituarte S, et al. First proteome of the egg perivitelline fluid of a freshwater gastropod with aerial oviposition. J Proteome Res. 2012;11:4240-8.

36. Aplysia Genome Project. Broad Institute. Vertebrate Biology Group. 2009. https://www.broadinstitute.org/aplysia/aplysia-genome-project

37. Zhang G, Fang X, Guo X, Li L, Luo R, Xu F, et al. The oyster genome reveals stress adaptation and complexity of shell formation. Nature. 2012;490:49-54.

38. Du X, Fan G, Jiao Y, Zhang H, Guo X, Huang R, et al. The pearl oyster Pinctada fucata martensii genome and multi-omic analyses provide insights into biomineralization. Gigascience. 2017;6:1-12.

39. Takeuchi T, Kawashima T, Koyanagi R, Gyoja F, Tanaka M, Ikuta T, et al. Draft genome of the pearl oyster *Pinctada fucata*: a platform for understanding bivalve biology. DNA Res. 2012;19:117-30.

40. Simakov O, Marletaz F, Cho SJ, Edsinger-Gonzales E, Havlak P, Hellsten U, et al. Insights into bilaterian evolution from three spiralian genomes. Nature. 2013;493:526-31.

41. Albertin CB, Simakov O, Mitros T, Wang ZY, Pungor JR, Edsinger-Gonzales E, et al. The octopus genome and the evolution of cephalopod neural and morphological novelties. Nature. 2015;524:220-4.

42. Uliano-Silva M, Dondero F, Dan Otto T, Costa I, Lima NCB, Americo JA, et al. A hybrid-hierarchical genome assembly strategy to sequence the invasive golden mussel

Limnoperna fortunei. Gigascience. 2017. doi: 10.1093/gigascience/gix128

43. Adema CM, Hillier LW, Jones CS, Loker ES, Knight M, Minx P, et al. Corrigendum: Whole genome analysis of a schistosomiasis-transmitting freshwater snail. Nat Commun. 2017;8:16153.

44. Liu B, Shi Y, Yuan J, Hu X, Zhang H, Li N, et al. Estimation of genomic characteristics by analyzing k-mer frequency in de novo genome projects. Quantitative Biology 2013:arXiv:1308.2012 [q-bio.GN].

45. Guindon S, Dufayard JF, Lefort V, Anisimova M, Hordijk W, Gascuel O. New algorithms and methods to estimate maximum-likelihood phylogenies: assessing the performance of PhyML 3.0. Syst Biol 2010;59:307-21. doi:10.1093/sysbio/syq010.

46. Yang Z. PAML 4: phylogenetic analysis by maximum likelihood. Mol Biol Evol. 2007;24:1586-91. doi:10.1093/molbev/msm088.

47. Emms DM, Kelly S. OrthoFinder: solving fundamental biases in whole genome comparisons dramatically improves orthogroup inference accuracy. Genome Biol. 2015;16:157. doi:10.1186/s13059-015-0721-2.

48. Feschotte C, Wessler SR. Mariner-like transposases are widespread and diverse in flowering plants. Proc Natl Acad Sci U S A 2002;99:280-5.

49. Hua-Van A, Le Rouzic A, Boutin TS, Filée J, Capy P. The struggle for life of the genome's selfish architects. Biol Direct. 2011;6:19.

50. Werren JH. Selfish genetic elements, genetic conflict, and evolutionary innovation. Proc Natl Acad Sci U S A. 2011;108:10863-70.

51. Chrousos GP. Stress and disorders of the stress system. Nat Rev Endocrinol.

2009;5:374-81.

52. Vabulas RM, Raychaudhuri S, Hayer-Hartl M. Protein folding in the cytoplasm and the heat shock response. Cold Spring Harbor perspectives in biology. 2010;2:a004390.

53. Chen B，Retzlaff M，Roos T，Frydman J. Cellular Strategies of Protein Quality Control. Cold Spring Harbor Perspectives in Biology. 2011;3:a004374.

54. Korennykh A and Walter P. Structural basis of the unfolded protein response. Annu Rev Cell Dev Biol. 2012;28:251-77.

55. Chambers JE and Yarbrough JD. Xenobiotic biotransformation systems in fishes. Comp Biochem Physiol C. 1976;55:77-84.

56. Mello DF, de Oliveira ES, Vieira RC, Simoes E, Trevisan R, Dafre AL, et al. Cellular and Transcriptional Responses of *Crassostrea gigas* Hemocytes Exposed in Vitro to Brevetoxin (PbTx-2) Mar Drugs. 2012;10: 583-97.

57. Boutet I, Tanguy A, Moraga D. Characterisation and expression of four mRNA sequences encoding glutathione S-transferases pi, mu, omega and sigma classes in the Pacific oyster *Crassostrea gigas* exposed to hydrocarbons and pesticides. Mar Biol 2004;146:53-64.

58. Deeley RG, Westlake C, Cole SP. Transmembrane transport of endo- and xenobiotics by mammalian ATP-binding cassette multidrug resistance proteins. Physiol Rev. 2006;86:849-99.

59. Liu C, Zhang T, Wang L, Wang M, Wang W, Jia Z, et al. The modulation of extracellular superoxide dismutase in the specifically enhanced cellular immune response against secondary challenge of Vibrio splendidus in Pacific oyster (*Crassostrea gigas*). Dev Comp Immunol. 2016;63:163-70.

60. Lamb DC, Lei L, Warrilow AG, Lepesheva GI, Mullins JG, Waterman MR, et al. The first virally encoded cytochrome p450. J Virol. 2009;83:8266-9.

61. Urlacher VB, Girhard M. Cytochrome P450 monooxygenases: an update on perspectives for synthetic application. Trends Biotechnol. 2012;30:26-36.

62. Sanderson T, van den Berg M. Topic 3.1: Interactions of xenobiotics with the steroid hormone biosynthesis pathway. Pure Appl Chem. 2003;75:1957-71.

63. Goldstone JV, McArthur AG, Kubota A, Zanette J, Parente T, Jönsson ME, et al. Identification and developmental expression of the full complement of Cytochrome P450 genes in Zebrafish. BMC Genomics. 2010;11:643.

64. Chuang SS, Helvig C, Taimi M, Ramshaw HA, Collop AH, Amad M, et al. CYP2U1, a novel human thymus- and brain-specific cytochrome P450, catalyzes omega- and (omega-1)-hydroxylation of fatty acids. J Biol Chem. 2004;279:6305-14.

65. Fleming I. The pharmacology of the cytochrome P450 epoxygenase/soluble epoxide hydrolase axis in the vasculature and cardiovascular disease. Pharmacol Rev. 2014;66:1106-40.

66. Zhang G, Kodani S, Hammock BD. Stabilized epoxygenated fatty acids regulate inflammation, pain, angiogenesis and cancer. Prog Lipid Res. 2014;53:108-23.

67. de Jong-Brink M, Boer HH, Joosse J. Mollusca. In: Adiyodi, K.G., Adiyodi, R.G. (Eds.), Reproductive Biology of invertebrates. Oogenesis oviposition and oosorption, vol. 1. John Wiley & Sons Ltd., New York, 1983; pp. 297-355.

68. Garin CF, Heras H, Pollero RJ. Lipoproteins of the egg perivitelline fluid of *Pomacea canaliculata* snails (Mollusca: Gastropoda). J Exp Zool. 1996;276:307-14.

69. Dreon MS, Schinella G, Heras H, Pollero RJ. Antioxidant defense system in the apple snail eggs, the role of ovorubin. Arch Biochem Biophys. 2004;422:1-8.

70. Dreon MS, Ituarte S, Heras H. The role of the proteinase inhibitor ovorubin in apple snail eggs resembles plant embryo defense against predation. PLoS One. 2010;5:e15059. doi:10.1371/journal.pone.0015059.

71. Cardoso AM, Cavalcante JJV, Vieira RP, Lima JL, Grieco MAB, Clementino MM, et al. Gut Bacterial Communities in the Giant Land Snail *Achatina fulica* and Their Modification by Sugarcane-Based Diet. Plos One. 2012;7 doi:ARTN e3344010.1371/journal.pone.0033440.

72. Cardoso AM, Cavalcante JJV, Cantão ME, Thompson CE, Flatschart RB, Glogauer A, et al. Metagenomic Analysis of the Microbiota from the Crop of an Invasive Snail Reveals a Rich Reservoir of Novel Genes. Plos One. 2012;7 doi:ARTN e4850510.1371/journal.pone.0048505.

73. Cabrera G, Pérez R, Gómez JM, Ábalos A, Cantero D. Toxic effects of dissolved heavy metals on Desulfovibrio vulgaris and Desulfovibrio sp strains. J Hazard Mater 2006;135:40-6. doi:10.1016/j.jhazmat.2005.11.058.

74. Finlay JA, Allan VJ, Conner A, Callow ME, Basnakova G, Macaskie LE. Phosphate release and heavy metal accumulation by biofilm-immobilized and chemically-coupled cells of a Citrobacter sp. pre-grown in continuous culture. Biotechnol Bioeng. 1999;63:87-97.

75. Valls M, de Lorenzo V, Gonzalez-Duarte R, Atrian S. Engineering outer-membrane proteins in *Pseudomonas putida* for enhanced heavy-metal bioadsorption. J Inorg

Biochem. 2000;79:219-23.

76. Pinheiro GL, Correa RF, Cunha RS, Cardoso AM, Chaia C, Clementino MM, et al. Isolation of aerobic cultivable cellulolytic bacteria from different regions of the gastrointestinal tract of giant land snail *Achatina fulica*. Front Microbiol. 2015;6 doi:Artn 86010.3389/Fmicb.2015.00860.

77. Zoetendal EG, Heilig HG, Klaassens ES, Booijink CC, Kleerebezem M, Smidt H, et al. Isolation of DNA from bacterial samples of the human gastrointestinal tract. Nature protocols 2006, 1(2): 870-873.

78. Benson G. Tandem repeats finder: a program to analyze DNA sequences. Nucleic Acids Res. 1999;27:573-80.

79. Haas BJ, Salzberg SL, Zhu W, Pertea M, Allen JE, Orvis J, et al. Automated eukaryotic gene structure annotation using EVidenceModeler and the Program to Assemble Spliced Alignments. Genome Biol. 2008;9:R7.

80. Quevillon E, Silventoinen V, Pillai S, Harte N, Mulder N, Apweiler R, et al. InterProScan: protein domains identifier. Nucleic Acids Res. 2005;33:W116-20.

81. Kanehisa M, Goto S, Sato Y, Furumichi M, Tanabe M. KEGG for integration and interpretation of large-scale molecular data sets. Nucleic Acids Res. 2012;40:D109-D14.

82. Edgar RC. MUSCLE: multiple sequence alignment with high accuracy and high throughput. Nucleic Acids Res. 2004;32:1792-7.

83. Darriba D, Taboada GL, Doallo R, Posada D. ProtTest 3: fast selection of best-fit models of protein evolution. Bioinformatics. 2011;27:1164-5. doi:10.1093/bioinformatics/btr088.

84. Sun J, Zhang Y, Xu T, Zhang Y, Mu H, Zhang Y, et al. Adaptation to deep-sea

chemosynthetic environments as revealed by mussel genomes. Nature Ecology & Evolution. 2017; 1: 121.

85. Benton MJ, Donoghue PCJ, Asher RJ. in The Timetree of Life:Calibrating and Constraining Molecular Clocks (eds Hedges, S. B. & Kumar, S.)35–86 (Oxford Univ. Press, 2009.

86. Zapata F, Wilson NG, Howison M, Andrade SC, Jörger KM, Schrödl M, et al. Phylogenomic analyses of deep gastropod relationships reject Orthogastropoda. Proc Biol Sci. 2014;281(1794):20141739. doi: 10.1098/rspb.2014.1739.

87. Li H and Durbin R. Fast and accurate short read alignment with Burrows-Wheeler transform. Bioinformatics. 2009;25:1754-60.

88. Nurk S, Meleshko D, Korobeynikov A, Pevzner PA. metaSPAdes: a new versatile metagenomic assembler. Genome Res. 2017;27:824-34.

89. Hyatt D, LoCascio PF, Hauser LJ, Uberbacher EC. Gene and translation initiation site prediction in metagenomic sequences. Bioinformatics. 2012;28:2223-30.

90. Fu L, Niu B, Zhu Z, Wu S, Li W. CD-HIT: accelerated for clustering the next-generation sequencing data. Bioinformatics. 2012;28:3150-2.

91. Buchfink B, Chao X, Huson DH. Fast and sensitive protein alignment using DIAMOND. Nat Methods. 2015;12:59-60.

92. Gerlach W and Stoye J. Taxonomic classification of metagenomic shotgun sequences with CARMA3. Nucleic Acids Res. 2011;39 doi:Artn E9110.1093/Nar/Gkr225.

93. Kanehisa M, Goto S, Kawashima S, Okuno Y, Hattori M. The KEGG resource for deciphering the genome. Nucleic Acids Res. 2004;32:D277-80.

94. Yin Y, Mao X, Yang J, Chen X, Mao F, Xu Y. dbCAN: a web resource for automated carbohydrate-active enzyme annotation. Nucleic Acids Res. 2012;40:W445-51.

95. Eddy SR. Accelerated Profile HMM Searches. Plos Comput Biol. 2011;7 doi:ARTN e100219510.1371/journal.pcbi.1002195.

96. Qin JJ, Li YR, Cai ZM, Li SH, Zhu JF, Zhang F, et al. A metagenome-wide association study of gut microbiota in type 2 diabetes. Nature. 2012;490:55-60.

97. Liu C, Zhang Y, Ren Y, Wang H, Li S, Jiang F, et al. Supporting data for "The genome of the golden apple snail Pomacea canaliculata provides insight into stress tolerance and invasive adaptation". GigaScience Database. 2018. http://dx.doi.org/10.5524/100485

Figure

Click here to access/download;Figure;Fig.1.pdf

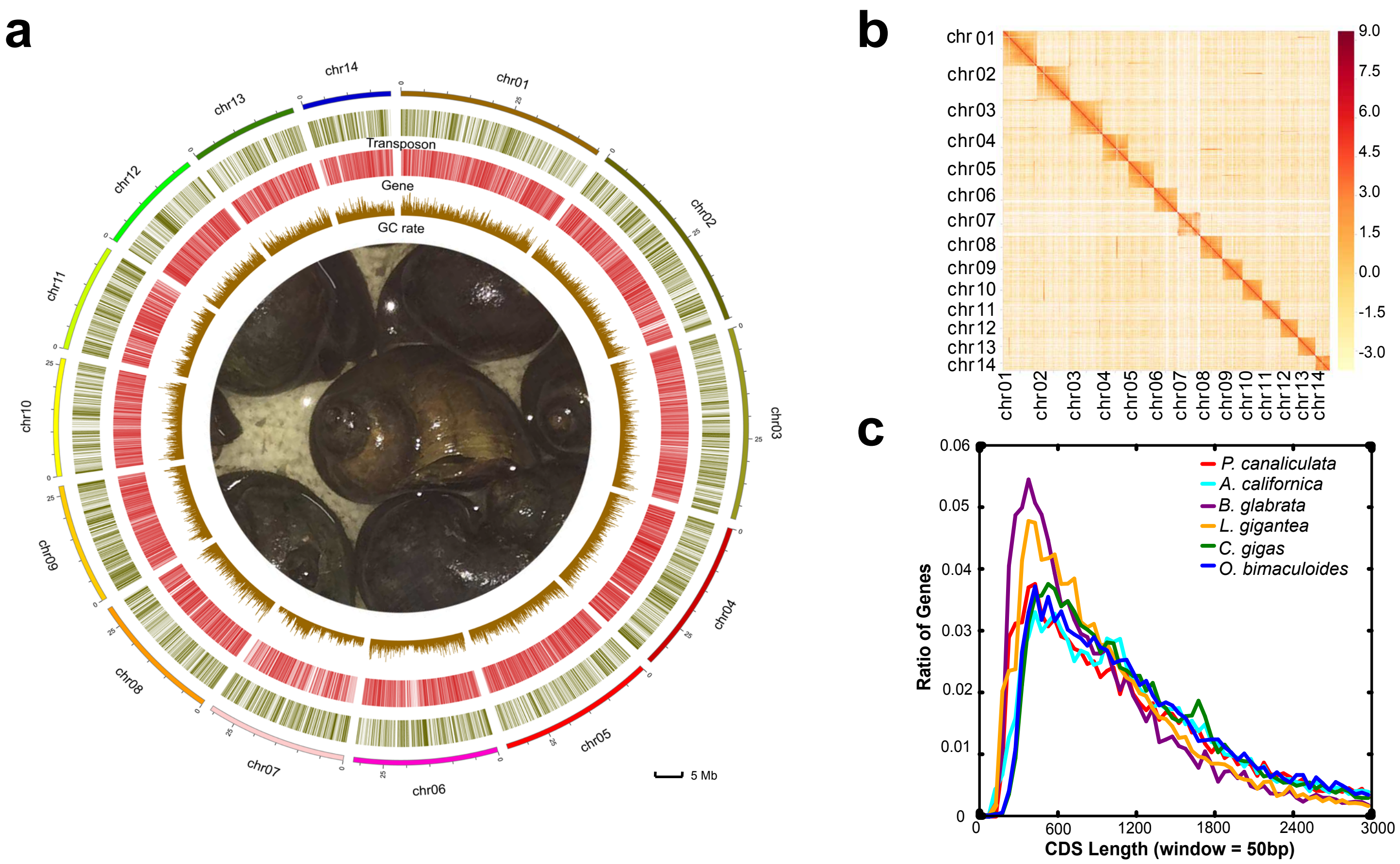

Figure

Click here to access/download;Figure;Fig.2.pdf

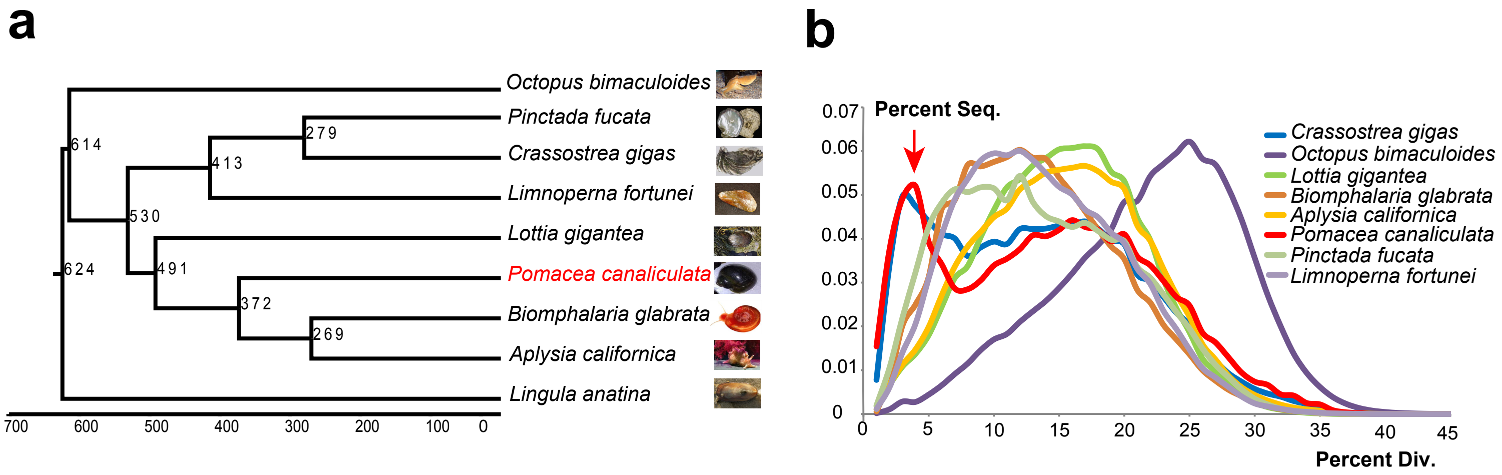

Figure

Click here to access/download;Figure;Fig.3.pdf

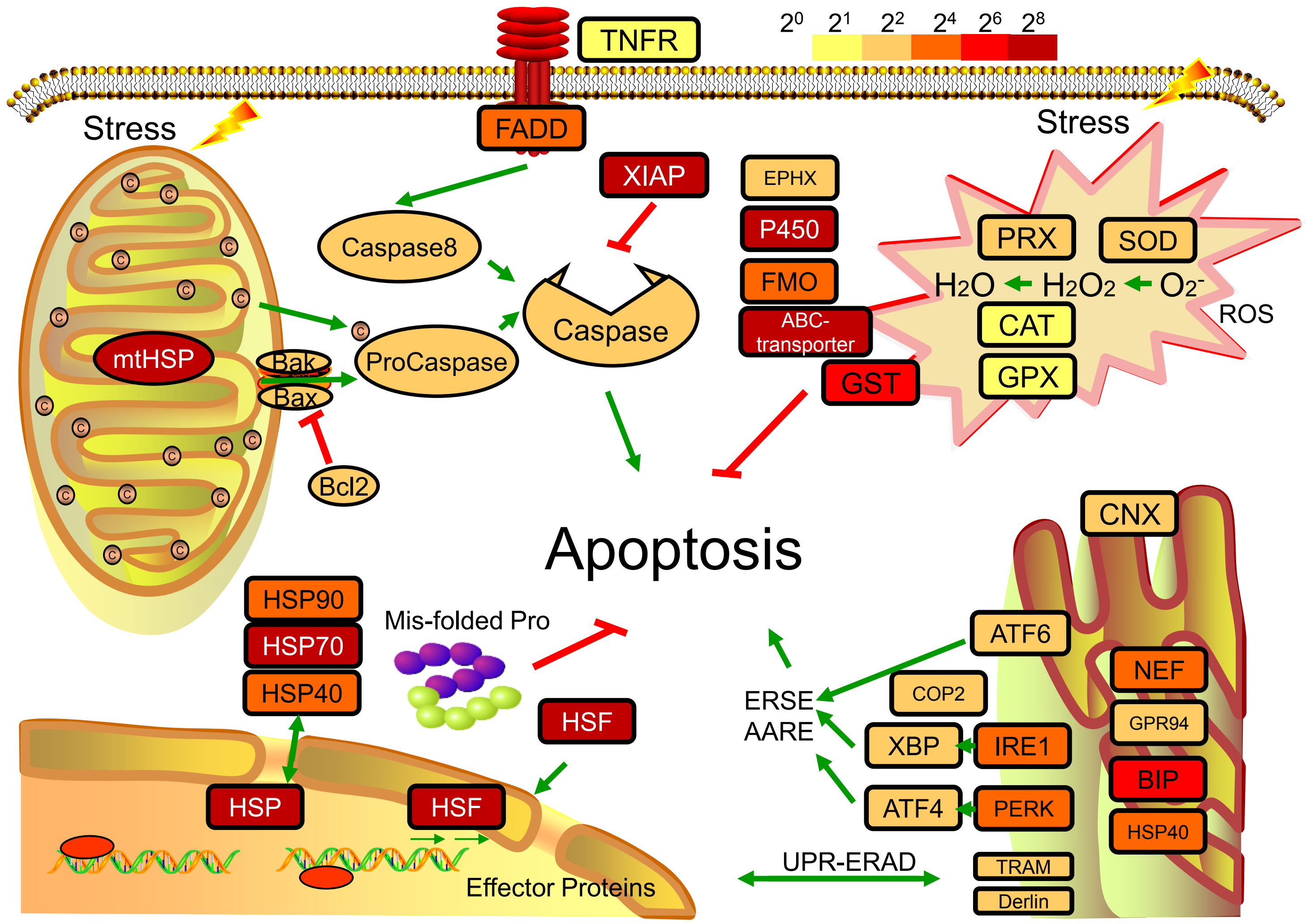

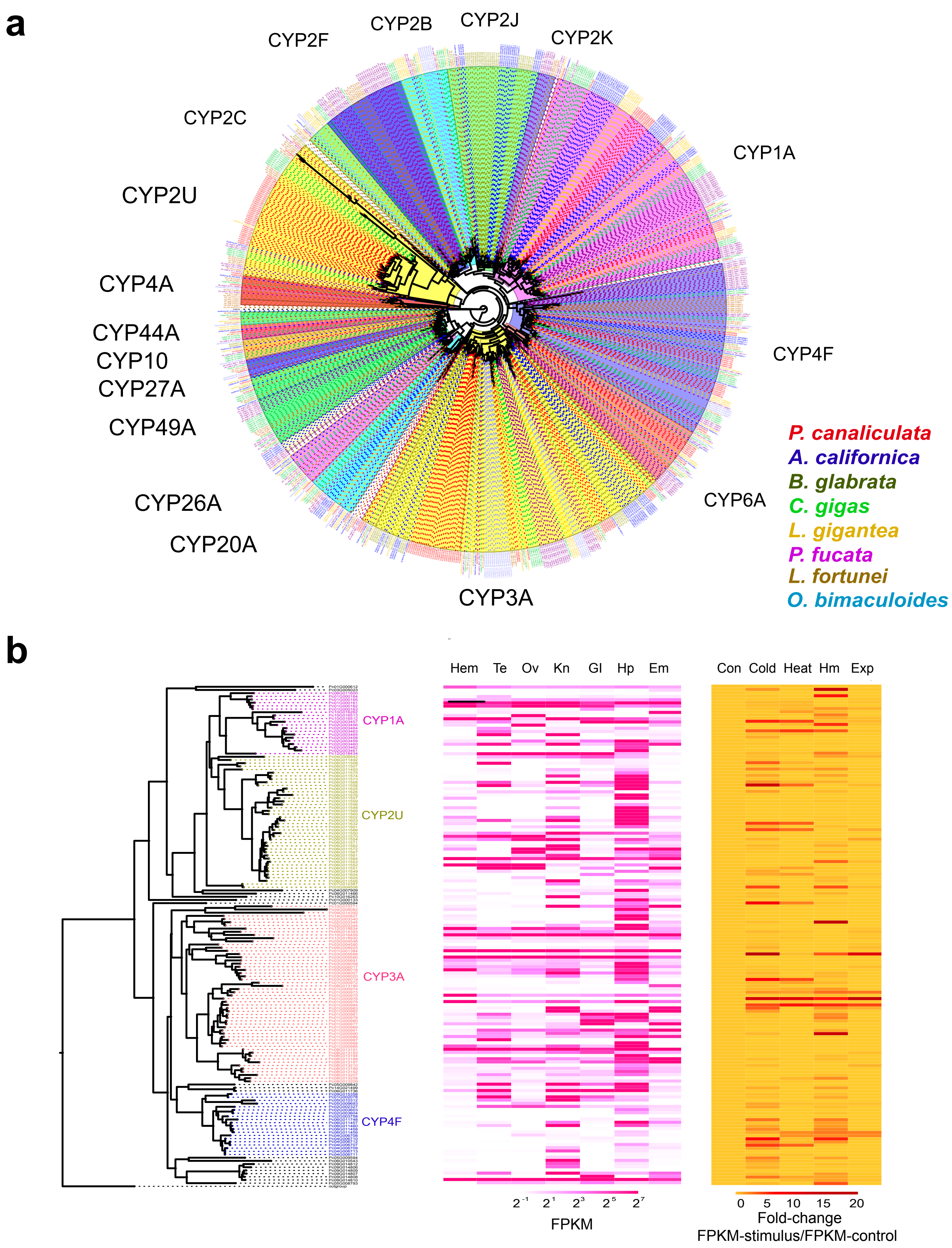
a
CYP2F
CYP2B
CYP2J
CYP2K
CYP2C
CYP1A
CYP2U
CYP4A
CYP44A
CYP10
CYP27A
CYP4F
CYP49A
P. canaliculata
A. californica
B. glabrata
C. gigas
L. gigantea
P. fucata
L. fortunei
O. bimaculoides
CYP26A
CYP6A
CYP20A
CYP3A
b
CYP1A
CYP2U
CYP3A
CYP4F
Hem
Te
Ov
Kn
Gl
Hp
Em
Con
Cold
Heat
Hm
Exp
2^-1 2^1 2^3 2^5 2^7
FPKM
0 5 10 15 20
Fold-change
FPKM-stimulus/FPKM-control

Figure
Click here to access/download;Figure;Fig.5.pdf

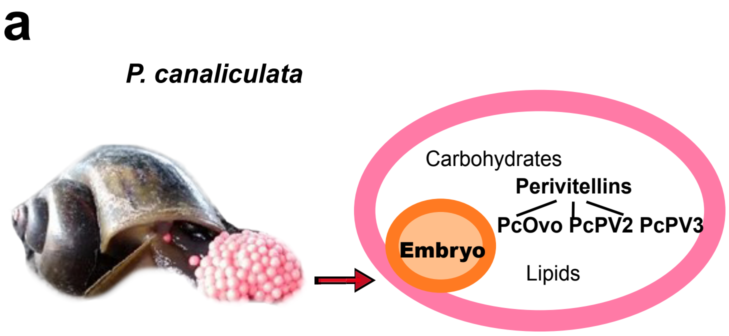

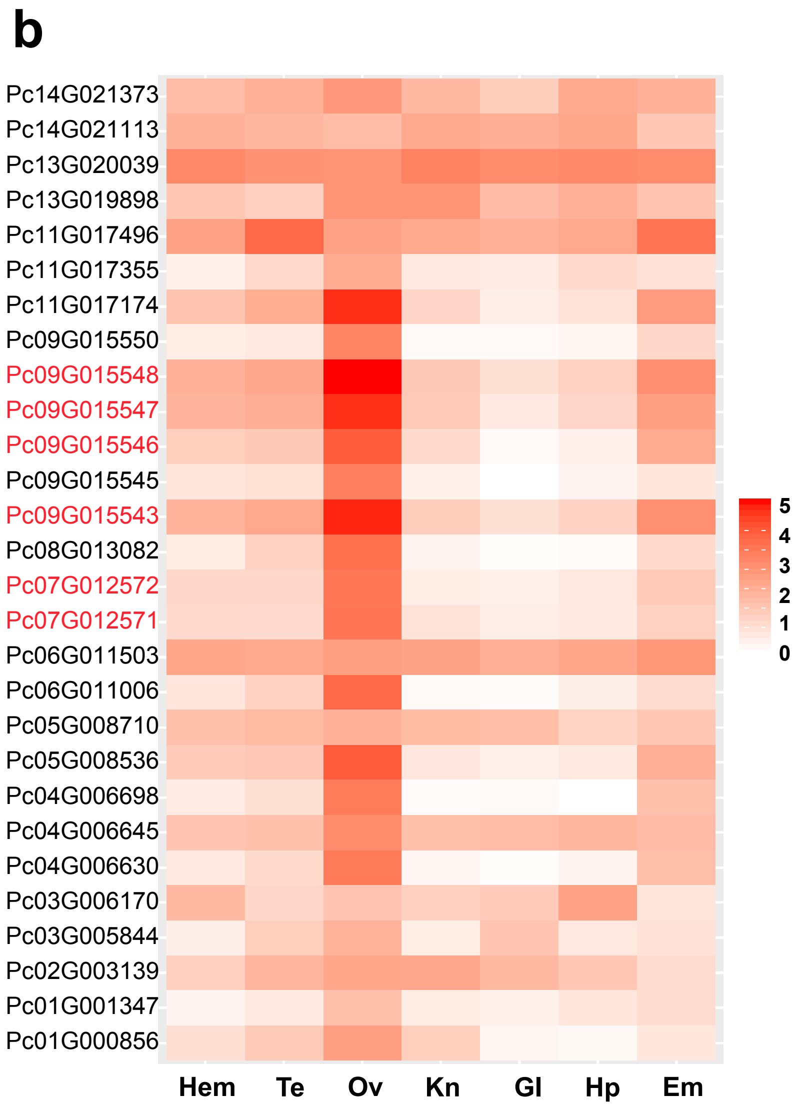

Click here to access/download
Supplementary Material
Supplemental_Information-final.doc
