# Supplementary material for: The genome of the golden apple snail Pomacea canaliculata provides insight into stress tolerance and invasive adaptation
Source: Gigascience. 2018 Aug 9;7(9):giy101. doi: 10.1093/gigascience/giy101 (PMC6129957; doi:10.1093/gigascience/giy101)
Supplement: Supplemental Tables and Figures [file giy101_supplemental_tables_and_figures.doc]

**Supplementary information**

**Supplementary tables**

Table S1. Statistics of sequencing data for various types of samples

| **Sample type** | **Sequencing Platform** | **Data Size (reads)** | **Data Size (base pairs)** |
| --- | --- | --- | --- |
| Genome | PacBio RsII | 3,607,008 | 26,623,591,565 |
|  | Hiseq2500 PE250 | 200,494,956 | 50,123,739,000 |
|  | HiseqX10  PE150 | 1,615,413,302 | 242,292,786,582 |
|  | Hi-C | 479,946,024 | 71,991,903,600 |
| Transcriptome | HiseqX10  PE150 | 1,409,408,426 | 211,411,263,900 |
| Metagenome | HiseqX10  PE150 | 623,168,338 | 93,475,250,700 |

The table is the platforms and data size used in this study for genome, transcriptome

and gut metagenome.

**Table S2. Statistics of gene predictions from different software in *P. canaliculata***

| **Statistics** | **AUGUSTUS** | **SNAP** | **GeneWise**  **(Protein1)** | **GeneWise**  **(Protein2)** | **GeneWise**  **(Protein3)** | **RNA-seq** | **Final** |
| --- | --- | --- | --- | --- | --- | --- | --- |
| Total Gene | 37,671 | 46,316 | 22,012 | 16,018 | 25,076 | 36,375 | 21,533 |
| Avg. CDS Length | 1,073 | 712 | 880 | 627 | 711 | 1,784 | 1,497 |
| Avg. exon number | 5.3 | 4.8 | 4.8 | 3.2 | 4.1 | 5.1 | 8.3 |
| Number of Exon | 200,806 | 220,514 | 105,233 | 52,017 | 101,822 | 182,739 | 179,026 |
| Avg. intron Length | 778 | 1768 | 941 | 939 | 927 | 1,092 | 968 |
| Avg. Exon Length | 201 | 149 | 184 | 193 | 175 | 355 | 180 |
| Single Exon Gene | 0.345 | 0.223 | 0.369 | 0.476 | 0.44 | 0.433 | 0.07 |

Protein1: *Aplysia Californica* gene set.

Protein2: *Biomphalaria glabrata* gene set.

Protein3: *Lottia giganta* gene set.

**Table S3. Genes in the cellular homeostasis system of *P. canaliculata***

| **Gene name** | **Number** | **GeneID** |
| --- | --- | --- |
| HSP70 | 13 | Pc01G000685,Pc03G005699,Pc04G007116,Pc03G005561,Pc03G004953,Pc05G009984,Pc11G017690,Pc07G012339,Pc09G014667,Pc02G004216,Pc01G000630,Pc05G010224, Pc01G000722 |
| HSP90 | 5 | Pc06G011498,Pc03G006457,Pc03G006072,Pc08G013797,Pc09G014481, |
| HSP40 | 7 | Pc05G008731,Pc05G008732,Pc10G016590,Pc01G001246,Pc06G011590,Pc10G016950,Pc10G016948 |
| HSF | 11 | Pc03G005218,Pc06G011716,Pc06G011583,Pc06G011582,Pc06G011581,Pc06G011580,Pc06G011579,Pc11G017106,Pc03G004397,Pc03G004395,Pc03G005789 |
| IRE1 | 2 | Pc12G018294,Pc03G006042 |
| PERK | 6 | Pc12G019029,Pc12G019028,Pc12G018952,Pc05G009350,Pc08G013924,Pc06G010846 |
| ATF6 | 1 | Pc11G017357 |
| P450 | 157 | Pc01G000133,Pc03G005023,Pc01G000612,Pc10G016263,Pc06G011466,Pc04G007909,Pc03G004506,Pc07G012387,Pc04G006842,Pc06G011492,Pc06G011508,Pc06G011507,Pc06G011493,Pc06G011575,Pc06G011574,Pc06G011573,Pc06G011568,Pc06G011558,Pc06G011555,Pc06G011605,Pc06G011604,Pc06G011549,Pc06G011551,Pc06G011552,Pc06G011553,Pc06G011564,Pc06G011561,Pc06G011567,Pc06G011572,Pc06G011562,Pc06G011571,Pc06G011554,Pc06G011570,Pc06G011550,Pc06G011566,Pc06G011601,Pc06G011632,Pc06G011603,Pc06G011602,Pc06G011565,Pc06G011569,Pc06G011548,Pc06G011556,Pc06G011559,Pc06G011557,Pc06G011576,Pc06G011626,Pc06G011625,Pc06G011600,Pc01G000164,Pc01G000165,Pc01G000161,Pc01G000162,Pc01G000163,Pc12G018434,Pc10G016262,Pc10G016513,Pc10G016512,Pc02G003457,Pc02G003456,Pc02G003464,Pc02G003463,Pc02G003465,Pc02G003458,Pc02G003459,Pc02G003460,Pc02G003462,Pc02G003461,Pc01G000594,Pc05G008793,Pc09G014810,Pc09G014808,Pc09G014807,Pc09G014809,Pc09G014806,Pc09G014812,Pc06G010543,Pc05G009594,Pc05G009842,Pc14G021499,Pc06G011736,Pc09G015358,Pc01G002078,Pc05G010312,Pc05G009693,Pc02G002327,Pc02G003603,Pc02G003604,Pc02G003758,Pc06G011748,Pc06G011457,Pc06G011460,Pc06G011458,Pc06G011459,Pc04G006708,Pc04G006710,Pc04G006712,Pc04G006707,Pc04G006709,Pc04G006713,Pc04G006711,Pc14G020827,Pc02G003340,Pc02G003345,Pc02G003344,Pc12G018834,Pc09G014393,Pc10G016459,Pc12G018930,Pc03G004548,Pc03G004580,Pc05G008925,Pc01G001384,Pc03G005688,Pc03G005690,Pc03G005691,Pc03G006058,Pc03G006017,Pc03G006018,Pc03G006057,Pc03G006020,Pc03G006019,Pc08G013191,Pc08G013193,Pc08G013194,Pc08G013188,Pc08G013187,Pc08G013210,Pc08G013189,Pc08G013192,Pc08G013207,Pc08G013208,Pc08G013209,Pc01G000985,Pc01G000988,Pc01G000987,Pc01G000986,Pc01G000990,Pc01G000991,Pc01G000989,Pc01G000977,Pc01G000980,Pc01G000978,Pc01G000981,Pc01G000982,Pc01G000983,Pc01G000984,Pc01G000979,Pc01G000976,Pc01G000975,Pc01G000973,Pc01G000974,Pc08G013190,Pc01G000972,Pc09G014392,Pc04G008082,Pc10G015771 |
| FMO | 15 | Pc04G006633,Pc14G021406,Pc09G015269,Pc01G000799,Pc01G000800,Pc11G017964,Pc11G017965,Pc11G017966,Pc11G017967,Pc12G018482,Pc14G021226,Pc14G021227,Pc14G021228,Pc14G021229,Pc14G021230 |
| GST | 50 | Pc01G000438,Pc01G000437,Pc01G000436,Pc13G020510,Pc03G005920,Pc11G017311,Pc12G018377,Pc11G018042,Pc09G015570,Pc09G015571,Pc09G015572,Pc09G015573,Pc12G018846,Pc01G001757,Pc05G009263,Pc06G010512,Pc04G006914,Pc08G013158,Pc08G013159,Pc08G013059,Pc02G003858,Pc02G003862,Pc04G007231,Pc04G008024,Pc06G010648,Pc03G005442,Pc08G013235,Pc04G008044,Pc01G001131,Pc11G017204,Pc11G017203,Pc11G017202,Pc11G017201,Pc11G017200,Pc11G017194,Pc11G017193,Pc11G017192,Pc09G015055,Pc09G015056,Pc10G016310,Pc06G010551,Pc02G003840,Pc02G004337,Pc09G014654,Pc05G008848,Pc06G010765,Pc04G007275,Pc12G018592,Pc02G004189 ,Pc05G009392 |
| ABC-transporters | 102 | Pc07G012684,Pc07G012687,Pc07G012688,Pc13G020363,Pc01G002031,Pc01G000190,Pc10G015979,Pc05G008613,Pc02G003977,Pc14G021284,Pc10G016587,Pc10G016640,Pc14G020675,Pc04G006993,Pc08G013637,Pc08G013636,Pc08G013635,Pc08G013537,Pc01G000191,Pc04G006554,Pc02G002982,Pc07G011891,Pc02G003180,Pc01G000958,Pc01G000957,Pc04G008429,Pc06G010426,Pc08G014031,Pc09G015528,Pc09G015527,Pc08G013151,Pc08G013152,Pc01G000296,Pc01G000297,Pc01G000298,Pc01G000299,Pc01G000300,Pc01G000301,Pc06G011714,Pc07G012443,Pc07G012441,Pc07G012440,Pc07G012439,Pc07G012438,Pc07G012437,Pc07G012436,Pc04G008113,Pc07G012793,Pc07G012651,Pc07G012650,Pc07G012649,Pc07G012648,Pc07G012647,Pc07G012646,Pc07G012645,Pc07G012643,Pc07G012642,Pc07G012641,Pc07G012640,Pc07G012639,Pc07G012638,Pc07G012637,Pc10G016221,Pc07G012469,Pc07G012468,Pc06G010652,Pc01G000816,Pc11G017125,Pc07G011859,Pc07G011858,Pc05G009414,Pc04G008212,Pc06G010316,Pc07G012335,Pc02G004057,Pc07G012719,Pc07G012718,Pc07G012717,Pc03G005144,Pc14G020843,Pc09G014692,Pc01G000353,Pc06G010619,Pc02G002523,Pc09G014433,Pc01G001676,Pc08G013294,Pc03G005809,Pc05G010176,Pc05G010173,Pc08G014287,Pc07G012033,Pc09G014860,Pc11G017346,Pc07G011879,Pc09G015320,Pc09G015321,Pc09G015322,Pc09G015323,Pc03G006015,Pc07G012636,Pc02G004035 |
| SOD | 4 | Pc05G008710,Pc06G011503,Pc09G014348,Pc06G011532 |
| CAT | 2 | Pc06G011145,Pc06G011176 |
| Prx | 3 | Pc08G014010,Pc08G014295,Pc10G016424 |
| GPX | 1 | Pc12G019035 |
| TNFR | 1 | Pc01G000745 |
| XIAP | 33 | Pc05G009169,Pc05G009170,Pc06G010924,Pc06G011799,Pc14G021350,Pc06G010406,Pc06G010407,Pc06G010425,Pc12G018654,Pc06G011578,Pc05G010309,Pc06G011129,Pc06G011135,Pc06G010689,Pc01G001603,Pc01G001602,Pc01G001601,Pc08G014173,Pc08G014172,Pc06G010596,Pc06G011178,Pc05G010200,Pc06G010889,Pc06G010888,Pc06G010887,Pc06G010885,Pc01G000639,Pc06G011225,Pc06G011226,Pc06G011229,Pc06G011230,Pc06G011231,Pc01G001589 |
| Bcl2 | 12 | Pc14G020631,Pc10G016458,Pc06G011372,Pc03G004903,Pc06G010466,Pc05G009982,Pc01G000737,Pc07G012068,Pc04G007973,Pc06G010777,Pc13G020050,Pc13G020081 |
| Bak | 2 | Pc06G011372,Pc13G020050 |

The table is the numbers and the gene IDs of the apoptosis genes.

**Table S4. The fold change** of apoptosis genes after different stimulus

|  | **COLD** | **HEAT** | **H-metal** | **Exposure** | **Total** |
| --- | --- | --- | --- | --- | --- |
| HSP70 | 6.459673 | 181.9473 | 49.71692 | 5.195767 | 243.3197 |
| HSP90 | 1.499631 | 14.10652 | 2.487652 | 0.926448 | 19.02025 |
| HSP40 | 6.969939 | 17.156 | 7.565144 | 6.958513 | 38.6496 |
| NEF | 4.914569 | 6.948091 | 3.114141 | 2.8772 | 17.854 |
| HSF | 36.00595 | 515.4244 | 112.9141 | 8.873658 | 673.2182 |
| TRAM | 1.121503 | 1.426366 | 0.720108 | 0.905824 | 4.1738 |
| Derlin | 1.944414 | 2.257947 | 1.709969 | 1.555357 | 7.467687 |
| PERK | 7.756796 | 7.94896 | 6.158262 | 7.754422 | 29.61844 |
| ATF4 | 1.703561 | 2.078309 | 1.744228 | 1.20433 | 6.730428 |
| ATF6 | 1.495915 | 1.711876 | 2.316532 | 1.443403 | 6.967726 |
| COP2 | 1.815957 | 1.898648 | 2.227554 | 2.284535 | 8.226694 |
| IRE1 | 10.35886 | 6.903043 | 1.759535 | 1.458844 | 20.48028 |
| XBP | 1.081782 | 1.497176 | 1.362271 | 0.922054 | 4.863283 |
| TRAF2 | 1.637009 | 3.225344 | 1.782821 | 2.524386 | 9.16956 |
| CNX | 1.080857 | 1.463232 | 0.728304 | 0.840008 | 4.112401 |
| NEF | 6.422634 | 10.29429 | 5.097661 | 5.17375 | 26.98833 |
| BIP | 3.11576 | 12.11487 | 58.02281 | 2.900762 | 76.1542 |
| GRP94 | 2.430843 | 6.033127 | 2.838138 | 1.379022 | 12.68113 |
| CAT | 1.368814 | 0.857907 | 0.703665 | 0.974536 | 3.904921 |
| SOD | 4.283926 | 2.674067 | 3.8362 | 4.020261 | 14.81445 |
| PRDX5 | 1.715124 | 1.978137 | 1.187737 | 1.572169 | 6.453167 |
| PRDX1 | 1.223071 | 0.920822 | 0.965228 | 0.424879 | 3.534001 |
| EPHX2 | 4.20375 | 2.062985 | 1.078835 | 0.492791 | 7.83836 |
| GPX | 1.042735 | 1 | 1.088609 | 1 | 4.131344 |
| GST | 22.86347 | 21.12611 | 16.31219 | 13.3775 | 73.67928 |
| cytochrome P450 | 235.1966 | 135.3783 | 154.0874 | 103.4709 | 628.1333 |
| FMO | 13.23473 | 9.543805 | 18.29613 | 5.649185 | 46.72384 |
| ABC-transportor | 873.7374 | 1347.352 | 925.6876 | 404.2391 | 3551.017 |
| TNF | 0.918361 | 1 | 0.999205 | 0.190756 | 3.108322 |
| FADD | 3.808588 | 7.811742 | 4.615306 | 6.641982 | 22.87762 |
| CASP8 | 2.288753 | 3.335247 | 1.506481 | 2.852247 | 9.982727 |
| CASP-pro | 5.013838 | 5.789056 | 4.363902 | 5.579801 | 20.7466 |
| CASP9 | 7.4627 | 5.107884 | 4.974776 | 5.668726 | 23.21409 |
| BAX | 1.086828 | 1.316722 | 0.964272 | 0.997401 | 4.365223 |
| XIAP | 69.00893 | 78.02759 | 118.7249 | 30.21887 | 295.9803 |
| BCL-XL | 1.333067 | 1.962047 | 2.83444 | 1.580384 | 7.709937 |
| BAK | 0.943673 | 1.764712 | 0.876813 | 1.786907 | 5.372105 |

The table is the apoptosis genes expression (fold change against the control group) in hemocytes after cold, heat, heavy metal and air exposure.

**Table S5**_a. The FPKM of P450 genes in different tissues

| **Gene_id** | **Aver_Hem** | **Bar_Hem** | **Aver_Te** | **Bar_Te** | **Aver_Ov** | **Bar_Ov** | **Aver_Kn** | **Bar_Kn** | **Aver_Gl** | **Bar_Gl** | **Aver_Hp** | **Bar_Hp** | **Aver_Em** | **Bar_Em** |
| --- | --- | --- | --- | --- | --- | --- | --- | --- | --- | --- | --- | --- | --- | --- |
| Pc01G000133 | 16.64 | 2.30 | 11.91 | 1.88 | 9.03 | 1.58 | 9.64 | 1.66 | 9.93 | 1.68 | 11.31 | 1.83 | 8.70 | 1.56 |
| Pc03G005023 | 0.36 | 0.39 | 0.28 | 0.33 | 0.00 | 0.00 | 0.99 | 0.87 | 1.64 | 1.37 | 0.49 | 0.44 | 4.69 | 2.38 |
| Pc01G000612 | 0.00 | 0.00 | 0.00 | 0.00 | 0.25 | 0.28 | 0.00 | 0.00 | 0.00 | 0.00 | 0.30 | 0.32 | 0.00 | 0.00 |
| Pc10G016263 | 0.00 | 0.00 | 7.21 | 3.70 | 0.00 | 0.00 | 1.70 | 1.50 | 3.17 | 2.48 | 1.74 | 1.44 | 2.62 | 1.95 |
| Pc06G011466 | 8.83 | 1.58 | 7.08 | 1.38 | 9.55 | 1.64 | 10.25 | 1.71 | 8.75 | 1.57 | 8.46 | 1.54 | 12.99 | 1.97 |
| Pc04G007909 | 30.93 | 3.91 | 22.03 | 3.15 | 34.57 | 4.20 | 27.06 | 3.58 | 19.91 | 2.96 | 45.05 | 5.04 | 54.57 | 5.78 |
| Pc03G004506 | 55.02 | 6.72 | 19.28 | 3.56 | 40.08 | 5.50 | 60.58 | 7.18 | 22.05 | 3.77 | 11.54 | 2.65 | 14.03 | 2.97 |
| Pc07G012387 | 0.68 | 0.58 | 2.51 | 1.14 | 1.41 | 0.90 | 0.82 | 0.68 | 10.90 | 2.55 | 0.41 | 0.38 | 1.84 | 1.07 |
| Pc04G006842 | 1.00 | 0.69 | 3.16 | 1.25 | 2.53 | 1.11 | 2.08 | 0.99 | 1.15 | 0.72 | 0.20 | 0.19 | 23.98 | 3.75 |
| Pc06G011492 | 5.92 | 4.87 | 0.00 | 0.00 | 60.22 | 16.97 | 1.70 | 1.48 | 0.00 | 0.00 | 0.74 | 0.82 | 2.63 | 1.84 |
| Pc06G011508 | 503.81 | 36.46 | 24.47 | 4.99 | 5.04 | 2.15 | 45.49 | 7.09 | 21.20 | 4.54 | 16.85 | 4.01 | 6.76 | 2.49 |
| Pc06G011507 | 5.28 | 1.89 | 12.50 | 3.00 | 3.94 | 1.66 | 2.46 | 1.29 | 41.85 | 5.89 | 72.48 | 8.50 | 15.98 | 3.43 |
| Pc06G011493 | 4.39 | 1.74 | 0.70 | 0.59 | 62.10 | 7.79 | 1.11 | 0.87 | 0.74 | 0.63 | 1.45 | 1.00 | 7.09 | 2.25 |
| Pc06G011575 | 0.70 | 0.64 | 11.01 | 3.27 | 0.00 | 0.00 | 3.31 | 1.79 | 2.38 | 1.51 | 9.09 | 2.97 | 0.78 | 0.69 |
| Pc06G011574 | 0.33 | 0.35 | 14.80 | 4.07 | 4.96 | 2.31 | 3.08 | 1.83 | 4.18 | 2.04 | 2.17 | 1.53 | 1.84 | 1.42 |
| Pc06G011573 | 0.41 | 0.35 | 48.97 | 5.63 | 0.80 | 0.61 | 29.06 | 3.96 | 0.41 | 0.35 | 2.50 | 1.02 | 3.03 | 1.13 |
| Pc06G011568 | 0.00 | 0.00 | 0.00 | 0.00 | 0.76 | 0.80 | 0.67 | 0.76 | 0.00 | 0.00 | 0.00 | 0.00 | 2.01 | 1.65 |
| Pc06G011558 | 2.84 | 1.59 | 3.08 | 1.70 | 13.80 | 3.56 | 6.44 | 2.37 | 13.34 | 3.42 | 52.24 | 7.57 | 8.20 | 2.71 |
| Pc06G011555 | 8.00 | 4.03 | 23.05 | 6.84 | 3.74 | 2.87 | 116.83 | 17.00 | 5.90 | 3.38 | 19.89 | 6.24 | 21.90 | 6.70 |
| Pc06G011605 | 0.59 | 0.60 | 0.00 | 0.00 | 0.00 | 0.00 | 0.00 | 0.00 | 0.00 | 0.00 | 19.41 | 5.64 | 1.04 | 0.96 |
| Pc06G011604 | 0.91 | 0.88 | 0.70 | 0.70 | 0.91 | 0.81 | 2.15 | 1.92 | 1.85 | 1.80 | 31.42 | 9.52 | 0.00 | 0.00 |
| Pc06G011549 | 3.89 | 2.67 | 43.22 | 9.25 | 39.16 | 8.71 | 44.85 | 9.45 | 98.73 | 14.84 | 9.31 | 4.15 | 2.21 | 1.67 |
| Pc06G011551 | 1.03 | 0.98 | 7.51 | 4.23 | 8.76 | 4.59 | 13.35 | 5.66 | 16.71 | 6.29 | 8.16 | 4.36 | 0.00 | 0.00 |
| Pc06G011552 | 0.34 | 0.39 | 2.62 | 2.13 | 0.39 | 0.43 | 0.41 | 0.43 | 3.40 | 2.29 | 1.94 | 1.49 | 0.88 | 0.76 |
| Pc06G011553 | 0.42 | 0.44 | 30.79 | 7.09 | 0.51 | 0.51 | 1.80 | 1.52 | 3.29 | 2.20 | 2.25 | 1.84 | 1.22 | 1.06 |
| Pc06G011564 | 0.38 | 0.45 | 0.00 | 0.00 | 0.00 | 0.00 | 0.50 | 0.51 | 0.36 | 0.45 | 3.14 | 2.48 | 0.53 | 0.53 |
| Pc06G011561 | 0.47 | 0.44 | 1.96 | 1.50 | 0.29 | 0.33 | 7.42 | 2.98 | 2.48 | 1.75 | 7.34 | 2.97 | 0.94 | 0.83 |
| Pc06G011567 | 0.50 | 0.51 | 1.38 | 1.16 | 0.00 | 0.00 | 3.76 | 2.79 | 0.57 | 0.55 | 13.62 | 5.11 | 0.51 | 0.52 |
| Pc06G011572 | 1.11 | 1.09 | 0.39 | 0.46 | 0.00 | 0.00 | 21.71 | 6.54 | 1.40 | 1.31 | 23.91 | 6.80 | 0.00 | 0.00 |
| Pc06G011562 | 0.98 | 0.89 | 0.95 | 0.90 | 0.76 | 0.73 | 1.23 | 1.07 | 1.53 | 1.29 | 40.57 | 7.68 | 0.62 | 0.59 |
| Pc06G011571 | 6.36 | 5.33 | 17.81 | 9.29 | 2.91 | 2.40 | 221.92 | 34.76 | 15.19 | 8.49 | 33.03 | 12.09 | 6.73 | 3.89 |
| Pc06G011554 | 0.75 | 0.83 | 68.14 | 18.19 | 4.98 | 4.33 | 1.91 | 1.79 | 9.66 | 6.64 | 43.43 | 14.11 | 1.13 | 1.04 |
| Pc06G011570 | 3.05 | 2.98 | 0.80 | 0.87 | 0.90 | 0.91 | 4.05 | 3.02 | 0.00 | 0.00 | 87.76 | 20.53 | 0.00 | 0.00 |
| Pc06G011550 | 0.80 | 0.74 | 5.83 | 3.50 | 5.71 | 3.46 | 11.48 | 4.93 | 8.04 | 4.12 | 0.72 | 0.69 | 1.83 | 1.52 |
| Pc06G011566 | 1.88 | 1.72 | 1.43 | 1.27 | 0.00 | 0.00 | 1.88 | 1.72 | 2.90 | 2.62 | 40.64 | 12.64 | 0.00 | 0.00 |
| Pc06G011601 | 8.09 | 6.16 | 0.00 | 0.00 | 0.00 | 0.00 | 5.73 | 4.83 | 0.00 | 0.00 | 299.64 | 40.55 | 1.95 | 1.80 |
| Pc06G011632 | 0.76 | 0.76 | 0.00 | 0.00 | 0.00 | 0.00 | 0.65 | 0.72 | 0.00 | 0.00 | 2.27 | 1.96 | 0.82 | 0.79 |
| Pc06G011603 | 0.61 | 0.64 | 0.44 | 0.52 | 0.00 | 0.00 | 0.00 | 0.00 | 0.00 | 0.00 | 10.35 | 4.65 | 0.00 | 0.00 |
| Pc06G011602 | 6.95 | 4.90 | 0.63 | 0.71 | 0.00 | 0.00 | 8.07 | 5.26 | 0.00 | 0.00 | 237.09 | 32.06 | 3.56 | 2.76 |
| Pc06G011565 | 1.79 | 1.53 | 0.69 | 0.64 | 0.60 | 0.60 | 0.61 | 0.60 | 0.00 | 0.00 | 46.43 | 9.54 | 0.91 | 0.87 |
| Pc06G011569 | 0.00 | 0.00 | 0.00 | 0.00 | 0.00 | 0.00 | 1.91 | 1.86 | 0.00 | 0.00 | 77.77 | 26.14 | 0.00 | 0.00 |
| Pc06G011548 | 9.92 | 3.31 | 0.58 | 0.57 | 0.36 | 0.35 | 15.96 | 4.28 | 0.66 | 0.63 | 271.90 | 23.21 | 6.65 | 2.69 |
| Pc06G011556 | 4.00 | 1.05 | 18.51 | 2.55 | 4.52 | 1.13 | 70.76 | 5.93 | 3.20 | 0.93 | 49.56 | 5.02 | 3.45 | 0.99 |
| Pc06G011559 | 5.18 | 1.98 | 0.73 | 0.64 | 0.21 | 0.24 | 1.32 | 1.04 | 0.28 | 0.27 | 94.05 | 10.64 | 0.66 | 0.58 |
| Pc06G011557 | 1.27 | 0.65 | 0.89 | 0.57 | 4.97 | 1.33 | 13.09 | 2.27 | 2.56 | 0.95 | 16.03 | 2.57 | 1.45 | 0.71 |
| Pc06G011576 | 0.00 | 0.00 | 0.00 | 0.00 | 0.83 | 0.77 | 0.00 | 0.00 | 0.00 | 0.00 | 0.00 | 0.00 | 1.36 | 1.29 |
| Pc06G011626 | 5.87 | 2.42 | 13.04 | 3.70 | 7.12 | 2.70 | 58.34 | 8.51 | 17.65 | 4.31 | 16.15 | 4.12 | 5.80 | 2.41 |
| Pc06G011625 | 20.40 | 3.31 | 5212.41 | 238.70 | 39.95 | 5.03 | 6.74 | 1.79 | 2.16 | 0.99 | 2.71 | 1.12 | 10.44 | 2.27 |
| Pc06G011600 | 9.08 | 2.22 | 12.80 | 2.68 | 22.94 | 3.76 | 50.92 | 6.18 | 3.86 | 1.42 | 18.80 | 3.36 | 7.47 | 2.01 |
| Pc01G000164 | 0.58 | 0.51 | 0.58 | 0.50 | 1.28 | 0.93 | 3.24 | 1.47 | 0.15 | 0.19 | 11.33 | 2.77 | 3.37 | 1.46 |
| Pc01G000165 | 3.66 | 1.04 | 0.43 | 0.35 | 2.20 | 0.80 | 374.32 | 19.36 | 1.36 | 0.63 | 0.75 | 0.47 | 5.39 | 1.28 |
| Pc01G000161 | 9.86 | 2.31 | 0.64 | 0.53 | 108.91 | 10.28 | 253.96 | 18.72 | 2.31 | 1.08 | 107.09 | 10.19 | 113.56 | 10.32 |
| Pc01G000162 | 0.68 | 0.56 | 0.76 | 0.62 | 25.09 | 3.98 | 4.68 | 1.60 | 2.00 | 1.02 | 12.20 | 2.66 | 15.46 | 3.03 |
| Pc01G000163 | 0.76 | 0.63 | 0.46 | 0.40 | 6.89 | 1.95 | 21.26 | 3.63 | 0.53 | 0.45 | 8.01 | 2.13 | 17.25 | 3.21 |
| Pc12G018434 | 36.46 | 4.97 | 23.37 | 3.79 | 20.18 | 3.47 | 27.82 | 4.22 | 22.84 | 3.79 | 19.28 | 3.41 | 44.60 | 5.67 |
| Pc10G016262 | 0.27 | 0.28 | 6.61 | 2.24 | 0.31 | 0.29 | 0.23 | 0.25 | 2.78 | 1.43 | 0.68 | 0.59 | 1.53 | 1.07 |
| Pc10G016513 | 51.24 | 5.81 | 28.14 | 3.95 | 397.30 | 21.53 | 5.19 | 1.51 | 2.03 | 0.91 | 11.41 | 2.30 | 5.93 | 1.64 |
| Pc10G016512 | 0.91 | 0.74 | 41.91 | 5.60 | 26.27 | 4.16 | 0.95 | 0.75 | 10.82 | 2.47 | 0.75 | 0.63 | 30.98 | 4.62 |
| Pc02G003457 | 1.24 | 0.57 | 5.04 | 1.20 | 1.70 | 0.67 | 1.67 | 0.68 | 0.99 | 0.51 | 14.46 | 2.18 | 6.74 | 1.40 |
| Pc02G003456 | 0.21 | 0.20 | 0.63 | 0.53 | 0.47 | 0.41 | 0.48 | 0.42 | 12.60 | 2.62 | 0.57 | 0.48 | 1.88 | 0.98 |
| Pc02G003464 | 0.00 | 0.00 | 0.00 | 0.00 | 6.09 | 2.52 | 0.00 | 0.00 | 0.24 | 0.30 | 0.37 | 0.35 | 0.71 | 0.66 |
| Pc02G003463 | 3.13 | 1.25 | 0.00 | 0.00 | 0.97 | 0.70 | 69.03 | 7.59 | 0.92 | 0.69 | 14.74 | 2.89 | 6.27 | 1.81 |
| Pc02G003465 | 0.48 | 0.43 | 0.00 | 0.00 | 1.08 | 0.81 | 12.73 | 2.77 | 0.21 | 0.21 | 9.48 | 2.37 | 10.44 | 2.48 |
| Pc02G003458 | 0.14 | 0.14 | 0.10 | 0.10 | 0.00 | 0.00 | 0.21 | 0.19 | 1.63 | 0.64 | 0.47 | 0.34 | 4.67 | 1.11 |
| Pc02G003459 | 2.50 | 0.83 | 0.25 | 0.22 | 0.24 | 0.21 | 3.87 | 1.06 | 3.01 | 0.92 | 83.57 | 6.80 | 1.72 | 0.70 |
| Pc02G003460 | 0.34 | 0.32 | 2.43 | 1.06 | 0.40 | 0.35 | 2.12 | 0.99 | 6.59 | 1.79 | 6.77 | 1.83 | 0.90 | 0.68 |
| Pc02G003462 | 2.24 | 0.76 | 0.14 | 0.14 | 1.04 | 0.52 | 57.40 | 5.00 | 0.68 | 0.41 | 7.02 | 1.40 | 3.48 | 0.96 |
| Pc02G003461 | 1.45 | 0.96 | 0.15 | 0.18 | 0.00 | 0.00 | 0.59 | 0.51 | 0.00 | 0.00 | 37.32 | 5.39 | 7.54 | 2.19 |
| Pc01G000594 | 0.68 | 0.48 | 0.79 | 0.52 | 0.19 | 0.18 | 1.56 | 0.72 | 0.75 | 0.51 | 13.49 | 2.26 | 1.22 | 0.64 |
| Pc05G008793 | 2.35 | 1.08 | 0.70 | 0.58 | 1.88 | 0.95 | 52.93 | 6.25 | 10.37 | 2.30 | 15.93 | 2.97 | 34.45 | 4.72 |
| Pc09G014810 | 4.50 | 1.54 | 0.36 | 0.34 | 0.55 | 0.47 | 1.21 | 0.82 | 0.15 | 0.17 | 183.92 | 14.46 | 2.68 | 1.20 |
| Pc09G014808 | 0.41 | 0.40 | 0.00 | 0.00 | 0.00 | 0.00 | 0.28 | 0.28 | 0.00 | 0.00 | 12.17 | 3.13 | 0.23 | 0.24 |
| Pc09G014807 | 2.77 | 1.05 | 0.25 | 0.24 | 0.35 | 0.31 | 0.46 | 0.39 | 0.31 | 0.28 | 82.27 | 8.17 | 3.52 | 1.17 |
| Pc09G014809 | 1.99 | 1.05 | 0.14 | 0.16 | 0.37 | 0.35 | 0.51 | 0.45 | 0.13 | 0.17 | 73.63 | 8.12 | 1.54 | 0.92 |
| Pc09G014806 | 0.53 | 0.46 | 0.89 | 0.71 | 2.81 | 1.24 | 0.69 | 0.58 | 4.98 | 1.68 | 2.00 | 1.09 | 6.63 | 1.97 |
| Pc09G014812 | 4.44 | 1.56 | 0.37 | 0.35 | 0.81 | 0.66 | 8.82 | 2.24 | 0.00 | 0.00 | 185.77 | 14.68 | 4.49 | 1.57 |
| Pc06G010543 | 31.09 | 5.27 | 11.20 | 3.02 | 22.30 | 4.37 | 20.13 | 4.11 | 7.98 | 2.50 | 52.30 | 7.24 | 8.59 | 2.61 |
| Pc05G009594 | 6.62 | 2.36 | 8.44 | 2.66 | 6.55 | 2.37 | 10.15 | 2.96 | 12.35 | 3.28 | 5.37 | 2.10 | 9.56 | 2.86 |
| Pc05G009842 | 28.90 | 4.46 | 42.28 | 5.67 | 28.71 | 4.42 | 34.25 | 4.95 | 28.37 | 4.42 | 98.18 | 10.11 | 96.64 | 9.96 |
| Pc14G021499 | 4.15 | 2.18 | 7.61 | 2.89 | 3.82 | 2.06 | 4.55 | 2.25 | 5.29 | 2.43 | 4.01 | 2.09 | 12.79 | 3.77 |
| Pc06G011736 | 2.11 | 1.54 | 0.85 | 0.75 | 2.96 | 1.82 | 3.22 | 1.84 | 2.30 | 1.66 | 4.23 | 2.10 | 2.02 | 1.54 |
| Pc09G015358 | 0.88 | 0.85 | 0.00 | 0.00 | 0.32 | 0.37 | 0.30 | 0.35 | 0.42 | 0.41 | 1.83 | 1.54 | 0.00 | 0.00 |
| Pc01G002078 | 5.50 | 1.80 | 1.26 | 0.85 | 0.41 | 0.39 | 2.22 | 1.16 | 2.45 | 1.22 | 6.06 | 1.90 | 0.67 | 0.56 |
| Pc05G010312 | 27.51 | 4.50 | 32.57 | 4.99 | 40.18 | 5.64 | 32.19 | 4.93 | 20.19 | 3.77 | 26.91 | 4.44 | 26.97 | 4.45 |
| Pc05G009693 | 0.24 | 0.29 | 1.62 | 1.33 | 1.71 | 1.33 | 5.05 | 2.27 | 0.27 | 0.29 | 6.04 | 2.47 | 1.74 | 1.35 |
| Pc02G002327 | 92.25 | 9.55 | 30.86 | 4.58 | 27.54 | 4.26 | 34.19 | 4.86 | 57.68 | 6.84 | 27.72 | 4.31 | 14.88 | 2.99 |
| Pc02G003603 | 10.72 | 2.71 | 8.00 | 2.31 | 4.88 | 1.78 | 7.21 | 2.21 | 5.88 | 1.98 | 8.38 | 2.38 | 3.77 | 1.55 |
| Pc02G003604 | 6.78 | 2.09 | 3.01 | 1.39 | 5.52 | 1.91 | 8.25 | 2.33 | 2.18 | 1.20 | 103.18 | 10.81 | 4.72 | 1.78 |
| Pc02G003758 | 9.84 | 2.61 | 8.11 | 2.37 | 8.66 | 2.46 | 8.12 | 2.34 | 5.49 | 1.93 | 29.50 | 4.83 | 9.70 | 2.61 |
| Pc06G011748 | 53.21 | 8.36 | 5.90 | 2.57 | 12.63 | 3.77 | 18.51 | 4.63 | 1.34 | 1.12 | 21.46 | 4.99 | 2.73 | 1.71 |
| Pc06G011457 | 2.97 | 1.23 | 7.46 | 2.02 | 11.52 | 2.52 | 91.67 | 9.33 | 12.17 | 2.58 | 34.79 | 4.81 | 7.20 | 1.98 |
| Pc06G011460 | 1.79 | 0.98 | 1.70 | 0.95 | 2.65 | 1.15 | 12.80 | 2.66 | 1.71 | 0.93 | 17.28 | 3.16 | 2.08 | 1.03 |
| Pc06G011458 | 0.93 | 0.72 | 0.19 | 0.19 | 0.22 | 0.20 | 1.52 | 0.92 | 0.82 | 0.65 | 20.40 | 3.54 | 0.60 | 0.50 |
| Pc06G011459 | 0.75 | 0.63 | 0.29 | 0.28 | 0.18 | 0.18 | 2.94 | 1.24 | 0.83 | 0.66 | 12.58 | 2.68 | 1.27 | 0.79 |
| Pc04G006708 | 0.94 | 0.78 | 0.74 | 0.63 | 0.25 | 0.25 | 0.00 | 0.00 | 0.63 | 0.57 | 0.18 | 0.22 | 18.54 | 3.87 |
| Pc04G006710 | 2.89 | 1.36 | 0.87 | 0.73 | 1.16 | 0.89 | 0.39 | 0.38 | 0.88 | 0.73 | 1.15 | 0.90 | 3.36 | 1.45 |
| Pc04G006712 | 0.61 | 0.54 | 0.00 | 0.00 | 0.00 | 0.00 | 0.17 | 0.20 | 0.15 | 0.19 | 0.30 | 0.28 | 1.92 | 1.12 |
| Pc04G006707 | 37.29 | 5.71 | 9.45 | 2.70 | 7.64 | 2.37 | 8.30 | 2.51 | 9.57 | 2.70 | 14.80 | 3.38 | 10.94 | 2.92 |
| Pc04G006709 | 0.98 | 1.04 | 1.24 | 1.17 | 1.09 | 1.09 | 0.00 | 0.00 | 0.00 | 0.00 | 1.06 | 1.09 | 1.39 | 1.25 |
| Pc04G006713 | 40.08 | 5.39 | 8.47 | 2.20 | 10.65 | 2.49 | 17.62 | 3.30 | 10.88 | 2.56 | 141.36 | 11.97 | 14.25 | 2.92 |
| Pc04G006711 | 0.46 | 0.53 | 0.00 | 0.00 | 0.67 | 0.63 | 0.00 | 0.00 | 0.39 | 0.49 | 3.02 | 2.52 | 0.00 | 0.00 |
| Pc14G020827 | 0.94 | 0.68 | 2.12 | 1.06 | 0.62 | 0.51 | 80.30 | 8.46 | 0.43 | 0.39 | 0.47 | 0.41 | 23.88 | 3.80 |
| Pc02G003340 | 1.46 | 0.95 | 0.34 | 0.33 | 0.45 | 0.41 | 126.14 | 12.04 | 5.39 | 1.83 | 3.11 | 1.39 | 142.82 | 12.46 |
| Pc02G003345 | 3.03 | 1.30 | 16.20 | 3.16 | 2.87 | 1.26 | 5.78 | 1.80 | 71.71 | 7.92 | 57.98 | 6.86 | 2.67 | 1.21 |
| Pc02G003344 | 1.56 | 0.83 | 7.83 | 1.92 | 2.62 | 1.08 | 0.81 | 0.59 | 16.15 | 2.80 | 45.48 | 5.42 | 0.85 | 0.65 |
| Pc12G018834 | 1.85 | 1.07 | 3.02 | 1.38 | 1.97 | 1.10 | 1.96 | 1.08 | 24.52 | 4.01 | 19.93 | 3.63 | 7.13 | 2.10 |
| Pc09G014393 | 8.19 | 3.20 | 8.55 | 3.27 | 2.19 | 1.68 | 5.54 | 2.67 | 31.41 | 6.48 | 3.06 | 1.93 | 24.77 | 5.74 |
| Pc10G016459 | 0.47 | 0.43 | 0.53 | 0.48 | 0.98 | 0.77 | 5.93 | 1.92 | 0.36 | 0.35 | 0.00 | 0.00 | 2.90 | 1.38 |
| Pc12G018930 | 1.97 | 0.80 | 16.65 | 2.55 | 15.02 | 2.38 | 14.05 | 2.29 | 20.91 | 2.91 | 17.27 | 2.60 | 33.52 | 3.98 |
| Pc03G004548 | 1.63 | 1.02 | 11.04 | 2.70 | 3.79 | 1.54 | 16.73 | 3.39 | 7.84 | 2.22 | 15.44 | 3.23 | 4.38 | 1.65 |
| Pc03G004580 | 11.30 | 2.66 | 10.48 | 2.54 | 3.62 | 1.45 | 6.30 | 1.94 | 6.44 | 1.97 | 32.73 | 4.87 | 22.03 | 3.87 |
| Pc05G008925 | 1.42 | 0.86 | 130.64 | 11.02 | 1.28 | 0.81 | 0.58 | 0.49 | 0.58 | 0.48 | 3.57 | 1.34 | 0.95 | 0.73 |
| Pc01G001384 | 1.76 | 0.98 | 0.00 | 0.00 | 0.00 | 0.00 | 0.39 | 0.37 | 0.00 | 0.00 | 29.18 | 4.47 | 0.83 | 0.68 |
| Pc03G005688 | 16.98 | 3.86 | 9.22 | 2.81 | 5.83 | 2.19 | 16.68 | 3.83 | 6.33 | 2.25 | 68.56 | 8.85 | 0.00 | 0.00 |
| Pc03G005690 | 37.39 | 5.88 | 25.84 | 4.74 | 23.53 | 4.49 | 37.88 | 5.92 | 21.18 | 4.21 | 176.69 | 15.98 | 50.65 | 7.12 |
| Pc03G005691 | 8.19 | 1.97 | 9.63 | 2.16 | 6.80 | 1.78 | 14.26 | 2.68 | 18.97 | 3.15 | 8.33 | 2.00 | 8.26 | 1.99 |
| Pc03G006058 | 0.84 | 0.68 | 0.59 | 0.50 | 0.00 | 0.00 | 0.16 | 0.17 | 1.33 | 0.83 | 29.03 | 4.28 | 0.27 | 0.27 |
| Pc03G006017 | 5.59 | 1.86 | 19.38 | 3.63 | 8.57 | 2.33 | 11.11 | 2.68 | 6.87 | 2.09 | 21.76 | 3.89 | 40.73 | 5.65 |
| Pc03G006018 | 4.89 | 1.88 | 17.16 | 3.66 | 17.55 | 3.67 | 4.36 | 1.78 | 16.13 | 3.55 | 21.37 | 4.17 | 48.19 | 6.70 |
| Pc03G006057 | 1.39 | 0.69 | 1.86 | 0.81 | 0.94 | 0.57 | 0.75 | 0.51 | 0.71 | 0.49 | 11.52 | 2.11 | 4.32 | 1.24 |
| Pc03G006020 | 2.87 | 1.52 | 1.67 | 1.15 | 7.75 | 2.48 | 6.77 | 2.34 | 2.18 | 1.33 | 13.93 | 3.39 | 6.14 | 2.24 |
| Pc03G006019 | 2.59 | 1.67 | 2.76 | 1.76 | 2.20 | 1.54 | 3.90 | 2.02 | 6.02 | 2.59 | 18.79 | 4.59 | 6.28 | 2.63 |
| Pc08G013191 | 2.00 | 0.67 | 10.76 | 1.72 | 1.14 | 0.52 | 7.90 | 1.43 | 6.16 | 1.24 | 14.67 | 2.06 | 3.13 | 0.87 |
| Pc08G013193 | 1.45 | 0.88 | 3.40 | 1.35 | 1.16 | 0.77 | 2.29 | 1.10 | 2.47 | 1.12 | 0.98 | 0.72 | 0.33 | 0.30 |
| Pc08G013194 | 2.40 | 0.60 | 3.48 | 0.74 | 3.90 | 0.79 | 3.85 | 0.78 | 3.52 | 0.74 | 5.23 | 0.93 | 7.26 | 1.13 |
| Pc08G013188 | 2.85 | 1.36 | 24.45 | 4.25 | 1.93 | 1.12 | 27.67 | 4.54 | 14.85 | 3.17 | 34.71 | 5.23 | 1.27 | 0.94 |
| Pc08G013187 | 0.89 | 0.51 | 4.67 | 1.21 | 0.54 | 0.40 | 5.15 | 1.26 | 2.54 | 0.87 | 22.60 | 3.00 | 4.34 | 1.17 |
| Pc08G013210 | 7.75 | 1.55 | 111.02 | 8.58 | 2.58 | 0.87 | 259.32 | 14.13 | 102.23 | 7.98 | 26.03 | 3.23 | 3.40 | 0.99 |
| Pc08G013189 | 0.33 | 0.28 | 9.04 | 1.70 | 0.66 | 0.44 | 3.03 | 0.94 | 2.66 | 0.88 | 1.87 | 0.74 | 1.20 | 0.59 |
| Pc08G013192 | 1.66 | 1.38 | 35.55 | 7.13 | 1.83 | 1.53 | 28.24 | 6.29 | 16.26 | 4.72 | 6.40 | 2.88 | 2.97 | 2.05 |
| Pc08G013207 | 1.05 | 0.73 | 12.48 | 2.54 | 3.39 | 1.27 | 43.92 | 5.43 | 9.82 | 2.23 | 7.27 | 1.89 | 0.68 | 0.56 |
| Pc08G013208 | 0.28 | 0.27 | 1.04 | 0.68 | 8.38 | 1.97 | 2.22 | 0.99 | 0.28 | 0.27 | 0.32 | 0.30 | 0.30 | 0.28 |
| Pc08G013209 | 1.64 | 1.10 | 16.68 | 3.57 | 18.63 | 3.78 | 16.00 | 3.46 | 4.91 | 1.85 | 6.77 | 2.20 | 4.44 | 1.78 |
| Pc01G000985 | 0.79 | 0.68 | 0.21 | 0.23 | 0.00 | 0.00 | 0.00 | 0.00 | 0.18 | 0.21 | 13.22 | 3.14 | 3.60 | 1.57 |
| Pc01G000988 | 0.46 | 0.45 | 0.00 | 0.00 | 0.00 | 0.00 | 0.00 | 0.00 | 0.00 | 0.00 | 8.59 | 2.68 | 2.23 | 1.33 |
| Pc01G000987 | 0.00 | 0.00 | 0.00 | 0.00 | 0.19 | 0.22 | 0.18 | 0.22 | 0.00 | 0.00 | 0.00 | 0.00 | 0.00 | 0.00 |
| Pc01G000986 | 0.27 | 0.27 | 0.62 | 0.56 | 0.00 | 0.00 | 0.26 | 0.26 | 1.99 | 1.23 | 0.85 | 0.72 | 4.36 | 1.81 |
| Pc01G000990 | 1.35 | 1.13 | 1.87 | 1.46 | 0.00 | 0.00 | 13.82 | 3.88 | 1.19 | 1.03 | 25.38 | 5.42 | 11.74 | 3.63 |
| Pc01G000991 | 0.95 | 0.65 | 6.21 | 1.71 | 0.41 | 0.36 | 27.69 | 3.95 | 0.51 | 0.43 | 18.28 | 3.09 | 3.64 | 1.29 |
| Pc01G000989 | 0.23 | 0.28 | 0.89 | 0.78 | 0.25 | 0.28 | 0.00 | 0.00 | 0.90 | 0.78 | 0.00 | 0.00 | 1.68 | 1.28 |
| Pc01G000977 | 0.42 | 0.42 | 0.00 | 0.00 | 1.68 | 1.40 | 1.26 | 1.07 | 0.51 | 0.47 | 5.48 | 2.60 | 0.37 | 0.38 |
| Pc01G000980 | 0.36 | 0.34 | 0.32 | 0.33 | 4.51 | 2.01 | 1.60 | 1.23 | 0.62 | 0.57 | 4.98 | 2.15 | 0.89 | 0.75 |
| Pc01G000978 | 0.19 | 0.23 | 0.54 | 0.51 | 0.00 | 0.00 | 1.39 | 1.08 | 2.08 | 1.32 | 1.01 | 0.84 | 0.25 | 0.26 |
| Pc01G000981 | 0.16 | 0.15 | 0.58 | 0.48 | 0.17 | 0.16 | 0.57 | 0.47 | 1.21 | 0.68 | 1.42 | 0.75 | 0.38 | 0.33 |
| Pc01G000982 | 0.46 | 0.49 | 0.00 | 0.00 | 0.00 | 0.00 | 1.48 | 1.27 | 0.66 | 0.62 | 9.65 | 3.86 | 2.33 | 1.86 |
| Pc01G000983 | 0.23 | 0.25 | 0.18 | 0.22 | 0.28 | 0.27 | 6.88 | 2.25 | 0.22 | 0.23 | 3.79 | 1.70 | 3.24 | 1.58 |
| Pc01G000984 | 1.08 | 0.89 | 0.76 | 0.64 | 0.93 | 0.77 | 17.73 | 3.77 | 0.56 | 0.49 | 3.54 | 1.65 | 1.17 | 0.91 |
| Pc01G000979 | 0.00 | 0.00 | 0.00 | 0.00 | 0.31 | 0.29 | 1.09 | 0.91 | 0.35 | 0.33 | 1.70 | 1.17 | 1.02 | 0.84 |
| Pc01G000976 | 0.30 | 0.27 | 0.19 | 0.19 | 0.00 | 0.00 | 4.99 | 1.36 | 0.39 | 0.34 | 1.49 | 0.74 | 1.07 | 0.62 |
| Pc01G000975 | 1.07 | 0.88 | 0.18 | 0.22 | 0.19 | 0.22 | 27.82 | 4.83 | 0.41 | 0.40 | 8.49 | 2.53 | 0.70 | 0.59 |
| Pc01G000973 | 0.30 | 0.27 | 0.52 | 0.43 | 0.40 | 0.34 | 17.27 | 2.71 | 0.08 | 0.11 | 3.04 | 1.05 | 0.81 | 0.55 |
| Pc01G000974 | 0.55 | 0.52 | 0.00 | 0.00 | 0.00 | 0.00 | 15.30 | 3.53 | 0.33 | 0.31 | 2.34 | 1.32 | 0.63 | 0.55 |
| Pc08G013190 | 0.00 | 0.00 | 0.00 | 0.00 | 0.00 | 0.00 | 0.00 | 0.00 | 0.00 | 0.00 | 0.00 | 0.00 | 0.00 | 0.00 |
| Pc01G000972 | 2.27 | 1.09 | 15.47 | 2.98 | 1.20 | 0.79 | 33.04 | 4.65 | 9.17 | 2.23 | 7.92 | 2.08 | 15.13 | 2.93 |
| Pc09G014392 | 0.36 | 0.39 | 1.63 | 1.34 | 0.33 | 0.38 | 0.31 | 0.36 | 49.90 | 7.87 | 0.31 | 0.37 | 12.05 | 3.87 |
| Pc04G008082 | 48.83 | 5.44 | 37.28 | 4.52 | 25.94 | 3.56 | 26.32 | 3.59 | 42.21 | 4.91 | 60.94 | 6.39 | 20.98 | 3.13 |
| Pc10G015771 | 0.50 | 0.58 | 7.69 | 4.40 | 8.36 | 4.56 | 4.74 | 3.40 | 12.73 | 5.66 | 5.48 | 3.86 | 8.25 | 4.55 |

The table is the P450 genes expression (FPKM) in seven tissues (Hem, hemocyte; Te, testis; Ov, Ovary and albumen gland; Kn, kidney; Gl, gill; Hp, hepatopancreas; Em, Embryo; Aver, the average FPKM for three replicates; Bar, the e-value for three replicates).

**Table S5**_b. The FPKM of P450 genes after different stimulus

| **Gene_id** | **FC-COLD** | **Bar-COLD** | **FC-HEAT** | **Bar-HEAT** | **FC-Hmetal** | **Bar-Hmetal** | **FC-EXP** | **Bar-EXP** |
| --- | --- | --- | --- | --- | --- | --- | --- | --- |
| Pc01G000133 | 1.03 | 0.40 | 1.18 | 0.31 | 0.97 | 0.47 | 1.15 | 0.32 |
| Pc03G005023 | 0.08 | 0.09 | 0.02 | 0.02 | 0.71 | 0.47 | 0.03 | 0.03 |
| Pc01G000612 | 0.00 | 0.00 | 0.00 | 0.00 | 0.00 | 0.00 | 0.00 | 0.00 |
| Pc10G016263 | 0.04 | 0.05 | 0.02 | 0.03 | 0.36 | 0.34 | 0.00 | 0.00 |
| Pc06G011466 | 0.76 | 0.31 | 0.58 | 0.17 | 0.42 | 0.22 | 0.73 | 0.22 |
| Pc04G007909 | 3.68 | 1.35 | 3.04 | 0.76 | 2.05 | 0.97 | 2.71 | 0.72 |
| Pc03G004506 | 6.45 | 2.36 | 5.45 | 1.36 | 4.16 | 1.95 | 3.94 | 1.06 |
| Pc07G012387 | 0.07 | 0.07 | 0.07 | 0.05 | 0.20 | 0.15 | 0.10 | 0.07 |
| Pc04G006842 | 0.09 | 0.08 | 0.19 | 0.09 | 0.14 | 0.11 | 0.05 | 0.04 |
| Pc06G011492 | 0.59 | 0.59 | 0.24 | 0.18 | 2.29 | 1.61 | 0.37 | 0.33 |
| Pc06G011508 | 44.93 | 16.11 | 19.84 | 4.84 | 78.54 | 35.84 | 49.78 | 12.69 |
| Pc06G011507 | 3.49 | 1.35 | 2.24 | 0.62 | 0.61 | 0.37 | 0.63 | 0.24 |
| Pc06G011493 | 0.29 | 0.18 | 0.46 | 0.18 | 2.84 | 1.38 | 0.38 | 0.17 |
| Pc06G011575 | 0.04 | 0.04 | 0.00 | 0.00 | 0.03 | 0.04 | 0.00 | 0.00 |
| Pc06G011574 | 0.14 | 0.13 | 0.14 | 0.10 | 0.14 | 0.15 | 0.03 | 0.02 |
| Pc06G011573 | 0.05 | 0.05 | 0.04 | 0.03 | 0.02 | 0.03 | 0.02 | 0.02 |
| Pc06G011568 | 0.09 | 0.09 | 0.00 | 0.00 | 0.00 | 0.00 | 0.00 | 0.00 |
| Pc06G011558 | 0.07 | 0.07 | 0.15 | 0.09 | 0.16 | 0.15 | 0.06 | 0.05 |
| Pc06G011555 | 0.82 | 0.52 | 0.16 | 0.12 | 0.74 | 0.57 | 0.21 | 0.18 |
| Pc06G011605 | 0.04 | 0.04 | 0.00 | 0.00 | 0.00 | 0.00 | 0.00 | 0.00 |
| Pc06G011604 | 0.05 | 0.06 | 0.00 | 0.00 | 0.05 | 0.06 | 0.00 | 0.00 |
| Pc06G011549 | 0.54 | 0.38 | 0.31 | 0.21 | 1.52 | 0.94 | 0.39 | 0.26 |
| Pc06G011551 | 0.13 | 0.15 | 0.03 | 0.03 | 0.26 | 0.29 | 0.16 | 0.14 |
| Pc06G011552 | 0.04 | 0.04 | 0.00 | 0.00 | 0.00 | 0.00 | 0.00 | 0.00 |
| Pc06G011553 | 0.17 | 0.17 | 0.04 | 0.04 | 0.04 | 0.04 | 0.00 | 0.00 |
| Pc06G011564 | 0.03 | 0.04 | 0.00 | 0.00 | 0.03 | 0.04 | 0.00 | 0.00 |
| Pc06G011561 | 0.17 | 0.15 | 0.08 | 0.07 | 0.14 | 0.16 | 0.04 | 0.04 |
| Pc06G011567 | 0.00 | 0.00 | 0.00 | 0.00 | 0.00 | 0.00 | 0.00 | 0.00 |
| Pc06G011572 | 0.22 | 0.22 | 0.03 | 0.03 | 0.23 | 0.25 | 0.06 | 0.06 |
| Pc06G011562 | 0.09 | 0.09 | 0.00 | 0.00 | 0.00 | 0.00 | 0.02 | 0.02 |
| Pc06G011571 | 1.85 | 1.25 | 0.67 | 0.49 | 1.05 | 0.96 | 0.21 | 0.21 |
| Pc06G011554 | 1.06 | 0.85 | 0.23 | 0.18 | 0.00 | 0.00 | 0.08 | 0.07 |
| Pc06G011570 | 0.10 | 0.10 | 0.12 | 0.11 | 0.00 | 0.00 | 0.00 | 0.00 |
| Pc06G011550 | 0.20 | 0.20 | 0.04 | 0.04 | 0.12 | 0.14 | 0.00 | 0.00 |
| Pc06G011566 | 0.15 | 0.16 | 0.04 | 0.04 | 0.07 | 0.08 | 0.00 | 0.00 |
| Pc06G011601 | 0.33 | 0.35 | 0.10 | 0.09 | 0.00 | 0.00 | 0.17 | 0.15 |
| Pc06G011632 | 0.05 | 0.06 | 0.00 | 0.00 | 0.07 | 0.08 | 0.00 | 0.00 |
| Pc06G011603 | 0.04 | 0.05 | 0.00 | 0.00 | 0.00 | 0.00 | 0.00 | 0.00 |
| Pc06G011602 | 0.28 | 0.29 | 0.09 | 0.08 | 0.05 | 0.07 | 0.06 | 0.06 |
| Pc06G011565 | 0.03 | 0.04 | 0.00 | 0.00 | 0.00 | 0.00 | 0.00 | 0.00 |
| Pc06G011569 | 0.00 | 0.00 | 0.00 | 0.00 | 0.00 | 0.00 | 0.00 | 0.00 |
| Pc06G011548 | 0.38 | 0.26 | 0.11 | 0.09 | 0.90 | 0.55 | 0.08 | 0.07 |
| Pc06G011556 | 0.32 | 0.15 | 0.05 | 0.03 | 0.36 | 0.20 | 0.06 | 0.04 |
| Pc06G011559 | 2.93 | 1.17 | 1.86 | 0.54 | 0.20 | 0.16 | 0.03 | 0.03 |
| Pc06G011557 | 0.22 | 0.12 | 0.04 | 0.03 | 0.16 | 0.11 | 0.03 | 0.03 |
| Pc06G011576 | 0.23 | 0.24 | 0.14 | 0.12 | 0.00 | 0.00 | 0.07 | 0.07 |
| Pc06G011626 | 0.30 | 0.21 | 0.07 | 0.06 | 0.33 | 0.26 | 0.14 | 0.10 |
| Pc06G011625 | 0.06 | 0.05 | 0.04 | 0.03 | 0.08 | 0.07 | 0.04 | 0.03 |
| Pc06G011600 | 1.36 | 0.56 | 0.36 | 0.14 | 0.76 | 0.41 | 1.60 | 0.47 |
| Pc01G000164 | 0.02 | 0.02 | 0.02 | 0.02 | 0.06 | 0.07 | 0.01 | 0.01 |
| Pc01G000165 | 0.45 | 0.20 | 0.09 | 0.05 | 0.59 | 0.31 | 0.10 | 0.05 |
| Pc01G000161 | 0.95 | 0.42 | 0.30 | 0.12 | 0.63 | 0.35 | 0.19 | 0.10 |
| Pc01G000162 | 0.06 | 0.06 | 0.03 | 0.03 | 0.10 | 0.10 | 0.02 | 0.02 |
| Pc01G000163 | 0.03 | 0.04 | 0.02 | 0.02 | 0.04 | 0.05 | 0.01 | 0.01 |
| Pc12G018434 | 1.50 | 0.61 | 0.84 | 0.27 | 0.83 | 0.44 | 1.46 | 0.44 |
| Pc10G016262 | 0.00 | 0.00 | 0.00 | 0.00 | 0.11 | 0.11 | 0.03 | 0.02 |
| Pc10G016513 | 1.29 | 0.53 | 1.86 | 0.50 | 1.04 | 0.53 | 0.95 | 0.30 |
| Pc10G016512 | 0.11 | 0.09 | 0.17 | 0.08 | 0.09 | 0.08 | 0.04 | 0.04 |
| Pc02G003457 | 0.24 | 0.12 | 0.04 | 0.03 | 0.11 | 0.08 | 0.02 | 0.02 |
| Pc02G003456 | 0.05 | 0.05 | 0.04 | 0.04 | 0.02 | 0.03 | 0.02 | 0.02 |
| Pc02G003464 | 0.02 | 0.02 | 0.00 | 0.00 | 0.07 | 0.07 | 0.00 | 0.00 |
| Pc02G003463 | 0.20 | 0.13 | 0.19 | 0.09 | 0.43 | 0.26 | 0.21 | 0.10 |
| Pc02G003465 | 0.04 | 0.04 | 0.01 | 0.01 | 0.03 | 0.04 | 0.02 | 0.02 |
| Pc02G003458 | 0.09 | 0.06 | 0.01 | 0.01 | 0.09 | 0.06 | 0.01 | 0.01 |
| Pc02G003459 | 0.04 | 0.04 | 0.00 | 0.00 | 0.09 | 0.07 | 0.01 | 0.01 |
| Pc02G003460 | 0.04 | 0.04 | 0.01 | 0.01 | 0.05 | 0.05 | 0.00 | 0.00 |
| Pc02G003462 | 0.26 | 0.13 | 0.02 | 0.02 | 0.12 | 0.08 | 0.09 | 0.05 |
| Pc02G003461 | 0.06 | 0.06 | 0.01 | 0.01 | 0.10 | 0.10 | 0.01 | 0.01 |
| Pc01G000594 | 0.70 | 0.30 | 0.20 | 0.08 | 0.07 | 0.06 | 0.01 | 0.01 |
| Pc05G008793 | 0.15 | 0.10 | 0.05 | 0.04 | 0.03 | 0.04 | 0.00 | 0.00 |
| Pc09G014810 | 0.04 | 0.04 | 0.03 | 0.02 | 0.00 | 0.00 | 0.00 | 0.00 |
| Pc09G014808 | 0.03 | 0.03 | 0.00 | 0.00 | 0.00 | 0.00 | 0.00 | 0.00 |
| Pc09G014807 | 0.20 | 0.12 | 0.04 | 0.03 | 0.10 | 0.08 | 0.09 | 0.06 |
| Pc09G014809 | 0.03 | 0.03 | 0.00 | 0.00 | 0.00 | 0.00 | 0.00 | 0.00 |
| Pc09G014806 | 0.06 | 0.06 | 0.02 | 0.02 | 1.31 | 0.67 | 0.02 | 0.02 |
| Pc09G014812 | 0.21 | 0.13 | 0.11 | 0.06 | 0.00 | 0.00 | 0.16 | 0.09 |
| Pc06G010543 | 1.28 | 0.57 | 0.75 | 0.27 | 1.28 | 0.69 | 2.59 | 0.76 |
| Pc05G009594 | 0.91 | 0.44 | 0.51 | 0.21 | 0.51 | 0.33 | 0.75 | 0.29 |
| Pc05G009842 | 2.48 | 0.97 | 1.60 | 0.45 | 1.58 | 0.79 | 2.23 | 0.64 |
| Pc14G021499 | 0.40 | 0.26 | 0.33 | 0.17 | 0.27 | 0.23 | 0.39 | 0.20 |
| Pc06G011736 | 0.23 | 0.18 | 0.04 | 0.04 | 0.09 | 0.10 | 0.12 | 0.10 |
| Pc09G015358 | 0.09 | 0.09 | 0.00 | 0.00 | 0.05 | 0.06 | 0.06 | 0.06 |
| Pc01G002078 | 0.89 | 0.40 | 0.13 | 0.08 | 0.32 | 0.21 | 0.62 | 0.23 |
| Pc05G010312 | 2.40 | 0.95 | 1.60 | 0.46 | 1.99 | 0.98 | 2.16 | 0.63 |
| Pc05G009693 | 0.65 | 0.36 | 0.02 | 0.02 | 0.11 | 0.12 | 0.28 | 0.16 |
| Pc02G002327 | 6.07 | 2.22 | 1.24 | 0.36 | 4.03 | 1.88 | 5.39 | 1.40 |
| Pc02G003603 | 0.48 | 0.25 | 0.37 | 0.15 | 0.41 | 0.26 | 0.75 | 0.27 |
| Pc02G003604 | 0.35 | 0.20 | 0.29 | 0.13 | 0.33 | 0.22 | 0.41 | 0.17 |
| Pc02G003758 | 0.96 | 0.44 | 0.45 | 0.18 | 0.56 | 0.34 | 1.02 | 0.35 |
| Pc06G011748 | 5.17 | 2.02 | 3.42 | 0.96 | 2.38 | 1.23 | 2.70 | 0.84 |
| Pc06G011457 | 0.44 | 0.22 | 0.10 | 0.06 | 0.42 | 0.26 | 0.11 | 0.07 |
| Pc06G011460 | 0.20 | 0.13 | 0.10 | 0.06 | 0.26 | 0.17 | 0.11 | 0.07 |
| Pc06G011458 | 0.90 | 0.40 | 0.46 | 0.17 | 0.09 | 0.08 | 0.01 | 0.01 |
| Pc06G011459 | 0.11 | 0.09 | 0.13 | 0.07 | 0.11 | 0.10 | 0.02 | 0.02 |
| Pc04G006708 | 0.12 | 0.10 | 0.02 | 0.02 | 0.03 | 0.04 | 0.06 | 0.05 |
| Pc04G006710 | 0.41 | 0.22 | 0.39 | 0.16 | 0.74 | 0.42 | 0.14 | 0.08 |
| Pc04G006712 | 0.17 | 0.13 | 0.12 | 0.07 | 0.28 | 0.20 | 0.22 | 0.12 |
| Pc04G006707 | 2.58 | 1.03 | 1.49 | 0.45 | 4.00 | 1.89 | 2.28 | 0.67 |
| Pc04G006709 | 1.18 | 1.11 | 0.80 | 0.67 | 0.60 | 0.69 | 1.46 | 1.01 |
| Pc04G006713 | 2.51 | 0.97 | 3.27 | 0.84 | 2.39 | 1.15 | 2.92 | 0.80 |
| Pc04G006711 | 0.22 | 0.22 | 0.24 | 0.19 | 0.28 | 0.30 | 0.20 | 0.17 |
| Pc14G020827 | 0.51 | 0.26 | 0.03 | 0.03 | 0.28 | 0.19 | 0.15 | 0.08 |
| Pc02G003340 | 0.37 | 0.21 | 0.08 | 0.06 | 0.19 | 0.15 | 0.04 | 0.04 |
| Pc02G003345 | 0.29 | 0.17 | 0.03 | 0.03 | 0.91 | 0.49 | 0.04 | 0.03 |
| Pc02G003344 | 0.17 | 0.11 | 0.01 | 0.01 | 0.55 | 0.31 | 0.02 | 0.02 |
| Pc12G018834 | 0.17 | 0.12 | 0.11 | 0.07 | 0.33 | 0.22 | 0.14 | 0.08 |
| Pc09G014393 | 0.74 | 0.43 | 0.17 | 0.12 | 0.17 | 0.18 | 0.50 | 0.25 |
| Pc10G016459 | 0.03 | 0.03 | 0.01 | 0.01 | 0.00 | 0.00 | 0.00 | 0.00 |
| Pc12G018930 | 0.13 | 0.08 | 0.07 | 0.04 | 0.40 | 0.22 | 0.10 | 0.05 |
| Pc03G004548 | 0.23 | 0.15 | 0.04 | 0.04 | 2.86 | 1.37 | 0.02 | 0.02 |
| Pc03G004580 | 0.61 | 0.30 | 0.34 | 0.14 | 0.45 | 0.27 | 0.60 | 0.22 |
| Pc05G008925 | 0.04 | 0.04 | 0.02 | 0.02 | 0.08 | 0.07 | 0.04 | 0.03 |
| Pc01G001384 | 0.06 | 0.06 | 0.04 | 0.03 | 0.11 | 0.10 | 0.10 | 0.07 |
| Pc03G005688 | 1.36 | 0.61 | 0.56 | 0.22 | 1.17 | 0.64 | 1.02 | 0.36 |
| Pc03G005690 | 3.68 | 1.35 | 3.04 | 0.76 | 2.05 | 0.97 | 2.71 | 0.72 |
| Pc03G005691 | 3.16 | 1.24 | 1.73 | 0.51 | 2.46 | 1.22 | 2.49 | 0.73 |
| Pc03G006058 | 0.59 | 0.28 | 0.23 | 0.10 | 0.43 | 0.25 | 0.84 | 0.27 |
| Pc03G006017 | 0.05 | 0.05 | 0.01 | 0.01 | 0.11 | 0.10 | 0.02 | 0.02 |
| Pc03G006018 | 0.68 | 0.33 | 0.29 | 0.13 | 0.70 | 0.40 | 0.40 | 0.17 |
| Pc03G006057 | 0.26 | 0.17 | 0.02 | 0.02 | 0.16 | 0.13 | 0.20 | 0.11 |
| Pc03G006020 | 0.19 | 0.11 | 0.05 | 0.04 | 0.09 | 0.07 | 0.19 | 0.09 |
| Pc03G006019 | 0.19 | 0.14 | 0.04 | 0.04 | 0.08 | 0.09 | 0.12 | 0.09 |
| Pc08G013191 | 0.07 | 0.07 | 0.05 | 0.05 | 0.11 | 0.12 | 0.02 | 0.02 |
| Pc08G013193 | 0.13 | 0.07 | 0.03 | 0.02 | 0.23 | 0.14 | 0.04 | 0.03 |
| Pc08G013194 | 0.09 | 0.08 | 0.16 | 0.08 | 0.46 | 0.28 | 0.10 | 0.07 |
| Pc08G013188 | 0.24 | 0.11 | 0.15 | 0.05 | 0.18 | 0.10 | 0.14 | 0.05 |
| Pc08G013187 | 0.22 | 0.15 | 0.04 | 0.03 | 0.18 | 0.15 | 0.06 | 0.05 |
| Pc08G013210 | 0.16 | 0.09 | 0.02 | 0.02 | 0.21 | 0.13 | 0.03 | 0.02 |
| Pc08G013189 | 0.62 | 0.26 | 0.03 | 0.02 | 0.61 | 0.32 | 0.05 | 0.03 |
| Pc08G013192 | 0.04 | 0.03 | 0.01 | 0.01 | 0.04 | 0.04 | 0.02 | 0.02 |
| Pc08G013207 | 0.18 | 0.17 | 0.04 | 0.04 | 0.33 | 0.28 | 0.07 | 0.07 |
| Pc08G013208 | 0.04 | 0.04 | 0.00 | 0.00 | 0.07 | 0.07 | 0.03 | 0.02 |
| Pc08G013209 | 0.05 | 0.05 | 0.02 | 0.02 | 0.03 | 0.03 | 0.02 | 0.02 |
| Pc01G000985 | 0.36 | 0.21 | 0.10 | 0.07 | 0.20 | 0.16 | 0.03 | 0.03 |
| Pc01G000988 | 0.04 | 0.04 | 0.00 | 0.00 | 0.00 | 0.00 | 0.00 | 0.00 |
| Pc01G000987 | 0.00 | 0.00 | 0.00 | 0.00 | 0.05 | 0.05 | 0.00 | 0.00 |
| Pc01G000986 | 0.00 | 0.00 | 0.00 | 0.00 | 0.03 | 0.04 | 0.00 | 0.00 |
| Pc01G000990 | 0.09 | 0.09 | 0.01 | 0.01 | 0.09 | 0.09 | 0.00 | 0.00 |
| Pc01G000991 | 0.14 | 0.13 | 0.04 | 0.04 | 0.34 | 0.26 | 0.02 | 0.02 |
| Pc01G000989 | 0.19 | 0.12 | 0.03 | 0.02 | 0.25 | 0.17 | 0.05 | 0.04 |
| Pc01G000977 | 0.06 | 0.07 | 0.00 | 0.00 | 0.07 | 0.08 | 0.00 | 0.00 |
| Pc01G000980 | 0.07 | 0.07 | 0.09 | 0.07 | 0.14 | 0.15 | 0.11 | 0.09 |
| Pc01G000978 | 0.02 | 0.03 | 0.09 | 0.07 | 0.12 | 0.12 | 0.12 | 0.09 |
| Pc01G000981 | 0.16 | 0.13 | 0.01 | 0.01 | 0.14 | 0.13 | 0.01 | 0.02 |
| Pc01G000982 | 0.06 | 0.05 | 0.02 | 0.02 | 0.05 | 0.05 | 0.01 | 0.01 |
| Pc01G000983 | 0.25 | 0.22 | 0.14 | 0.12 | 0.04 | 0.05 | 0.00 | 0.00 |
| Pc01G000984 | 0.04 | 0.04 | 0.00 | 0.00 | 0.02 | 0.02 | 0.00 | 0.00 |
| Pc01G000979 | 0.04 | 0.05 | 0.02 | 0.02 | 0.06 | 0.06 | 0.04 | 0.04 |
| Pc01G000976 | 0.05 | 0.05 | 0.04 | 0.03 | 0.04 | 0.04 | 0.01 | 0.02 |
| Pc01G000975 | 0.11 | 0.07 | 0.00 | 0.00 | 0.06 | 0.06 | 0.02 | 0.02 |
| Pc01G000973 | 0.24 | 0.16 | 0.07 | 0.06 | 0.04 | 0.05 | 0.05 | 0.05 |
| Pc01G000974 | 0.04 | 0.04 | 0.01 | 0.01 | 0.03 | 0.04 | 0.02 | 0.02 |
| Pc08G013190 | 0.08 | 0.08 | 0.01 | 0.01 | 0.04 | 0.05 | 0.02 | 0.02 |
| Pc01G000972 | 0.00 | 0.00 | 0.00 | 0.00 | 0.00 | 0.00 | 0.00 | 0.00 |
| Pc09G014392 | 0.26 | 0.16 | 0.11 | 0.07 | 0.54 | 0.31 | 0.17 | 0.09 |
| Pc04G008082 | 0.12 | 0.12 | 0.05 | 0.04 | 0.06 | 0.08 | 0.03 | 0.03 |
| Pc10G015771 | 3.57 | 1.31 | 4.50 | 1.11 | 9.85 | 4.45 | 4.55 | 1.18 |

The table is the P450 genes expression (FPKM) in hemocytes after cold, heat, heavy metal and air exposure (FC- indicates the fold change against the control group; Bar- indicates the e-value for three replicates).

**Table S6** **The blast results between 59 referenced PVF genes and *P.* *canaliculata*** proteome

| **Q-id** | **Q-len** | **S-id** | **S-len** | **Identity** | **Alignment-len** | **E-value** | **Bit-score** |
| --- | --- | --- | --- | --- | --- | --- | --- |
| SSH2 | 203 | Pc09G015547 | 203 | 98.03 | 203 | 1.00E-148 | 410 |
| SSH20 | 146 | Pc09G015548 | 602 | 96.8 | 125 | 1.00E-80 | 248 |
| SSH4 | 226 | Pc09G015543 | 210 | 97.14 | 210 | 2.00E-138 | 385 |
| SSH8 | 324 | Pc09G015548 | 602 | 99.48 | 194 | 6.00E-136 | 398 |
| SSH208 | 565 | Pc07G012571 | 614 | 91.37 | 614 | 0 | 1135 |
| SSH95 | 203 | Pc09G015546 | 128 | 97.66 | 128 | 2.00E-92 | 265 |
| SSH115 | 286 | Pc07G012572 | 288 | 96.15 | 286 | 0 | 564 |
| SSH36 | 168 | Pc11G017174 | 208 | 98.21 | 168 | 1.00E-118 | 333 |
| SSH122 | 30 | Pc09G015546 | 128 | 86.67 | 30 | 2.00E-13 | 57.8 |
| SSH218 | 186 | Pc05G008536 | 313 | 99.46 | 186 | 6.00E-124 | 351 |
| Pc109422 | 211 | Pc09G015550 | 347 | 98.1 | 211 | 5.00E-154 | 430 |
| Pc66440 | 61 | Pc03G005844 | 811 | 93.22 | 59 | 2.00E-29 | 107 |
| Pc123838 | 544 | Pc04G006645 | 810 | 97.41 | 540 | 0 | 1084 |
| Pc54251 | 55 | Pc08G013082 | 199 | 90.91 | 55 | 5.00E-31 | 105 |
| Pc99555 | 104 | Pc02G003139 | 550 | 100 | 104 | 1.00E-68 | 214 |
| Pc75576 | 73 | Pc09G015545 | 273 | 86.3 | 73 | 2.00E-40 | 132 |
| Pc52282 | 44 | Pc06G011006 | 430 | 95.45 | 44 | 6.00E-24 | 90.9 |
| Pc94087 | 124 | Pc04G006630 | 212 | 97.58 | 124 | 1.00E-86 | 250 |
| Pc49292 | 51 | Pc02G003139 | 550 | 100 | 51 | 7.00E-32 | 113 |
| Pc47034 | 52 | Pc05G008710 | 1014 | 94.12 | 51 | 2.00E-26 | 98.6 |
| Pc52841 | 34 | Pc05G008536 | 313 | 100 | 34 | 1.00E-17 | 72 |
| Pc59410 | 60 | Pc05G008710 | 1014 | 98.33 | 60 | 1.00E-32 | 116 |
| Pc65629 | 66 | Pc04G006698 | 196 | 100 | 65 | 3.00E-42 | 135 |
| Pc29496 | 100 | Pc08G013082 | 199 | 98 | 100 | 6.00E-69 | 204 |
| Pc112854 | 144 | Pc03G006170 | 771 | 99.23 | 130 | 2.00E-87 | 270 |
| Pc86328 | 85 | Pc13G020039 | 245 | 100 | 85 | 4.00E-56 | 172 |
| Pc86328 | 85 | Pc13G020039 | 245 | 100 | 85 | 4.00E-56 | 172 |
| Pc86328 | 85 | Pc13G020039 | 245 | 100 | 54 | 1.00E-32 | 112 |
| Pc86328 | 85 | Pc13G020038 | 832 | 100 | 85 | 1.00E-53 | 177 |
| Pc117840 | 125 | Pc13G019898 | 488 | 100 | 108 | 1.00E-72 | 224 |
| Pc119142 | 190 | Pc01G000856 | 182 | 97.66 | 128 | 3.00E-91 | 264 |
| Pc124918 | 301 | Pc02G003139 | 550 | 98.01 | 301 | 0 | 616 |
| Pc17674 | 71 | Pc11G017496 | 376 | 100 | 69 | 5.00E-46 | 149 |
| Pc17674 | 71 | Pc10G017025 | 376 | 95.65 | 69 | 9.00E-45 | 146 |
| Pc17674 | 71 | Pc10G017026 | 357 | 94.2 | 69 | 2.00E-43 | 142 |
| Pc101185 | 100 | Pc06G011503 | 157 | 99 | 100 | 4.00E-66 | 195 |
| Pc100845 | 105 | Pc03G004959 | 209 | 92.41 | 79 | 9.00E-52 | 161 |
| Pc106229 | 119 | Pc14G021373 | 459 | 99.16 | 119 | 1.00E-81 | 246 |
| Pc120959 | 212 | Pc01G001347 | 733 | 95.75 | 212 | 2.00E-136 | 398 |
| Pc122290 | 204 | Pc14G021113 | 362 | 98.53 | 204 | 2.00E-146 | 410 |
| Pc34343 | 316 | Pc11G017355 | 342 | 96.85 | 317 | 0 | 569 |
| Pc379 | 55 | Pc03G005844 | 811 | 87.27 | 55 | 2.00E-27 | 101 |

This table showed the results of 28 identified perivitellins genes, and the letter “Q” means “query”, the letter “S” means “Subject”, the abbreviation “len” means “length”.

**Table S7 The expression of perivitellin** genes in different tissues

| **GeneID** | **Hem_FPKM** | **Te_FPKM** | **Ov_FPKM** | **Kn_FPKM** | **Gl_FPKM** | **Dg_FPKM** | **De_FPKM** |
| --- | --- | --- | --- | --- | --- | --- | --- |
| Pc05G008710 | 50.6505 | 73.9476 | 123.641 | 68.4106 | 60.3573 | 13.9174 | 33.7886 |
| Pc06G011503 | 268.994 | 208.897 | 399.342 | 305.148 | 149.429 | 272.83 | 633.25 |
| Pc11G017496 | 321.835 | 7998.86 | 344.979 | 212.885 | 140.032 | 227.048 | 4259.44 |
| Pc04G006630 | 3.2907 | 8.8188 | 3124.56 | 0.725011 | 0.227392 | 1.0569 | 55.9542 |
| Pc04G006645 | 36.755 | 50.338 | 1160.61 | 50.3862 | 63.3439 | 91.7954 | 71.0555 |
| Pc14G021373 | 61.6859 | 130.038 | 594.427 | 81.9439 | 22.3613 | 206.914 | 127.509 |
| Pc02G003139 | 18.3761 | 99.8081 | 257.824 | 245.039 | 84.362 | 33.2195 | 8.16275 |
| Pc04G006698 | 2.58936 | 6.09549 | 2900.47 | 0.254928 | 0.353371 | 0 | 49.4454 |
| Pc09G015550 | 2.19885 | 3.00122 | 1777.38 | 0.351332 | 0.350677 | 0.920501 | 11.909 |
| Pc09G015548 | 135.962 | 238.416 | 171316.8 | 28.4109 | 5.95733 | 17.0749 | 1014.57 |
| Pc09G015547 | 103.842 | 157.346 | 79148.4 | 25.9818 | 2.92489 | 12.6931 | 374.299 |
| Pc09G015546 | 19.8961 | 28.3157 | 13440.1 | 8.99876 | 0.360408 | 1.48432 | 186.663 |
| Pc09G015545 | 4.17551 | 5.70941 | 2507.98 | 1.37584 | 0 | 0.975327 | 3.53133 |
| Pc09G015543 | 114.704 | 221.441 | 104141 | 22.3918 | 6.3234 | 15.1163 | 961.974 |
| Pc01G001347 | 0.975601 | 2.83692 | 54.1582 | 2.37386 | 1.42079 | 3.75353 | 8.31885 |
| Pc06G011006 | 4.29889 | 16.4752 | 8033.13 | 0.419774 | 0.257958 | 1.78467 | 8.29356 |
| Pc08G013082 | 2.39888 | 15.0859 | 5405.04 | 1.05548 | 0.092849 | 0.299574 | 8.73006 |
| Pc03G006170 | 81.5837 | 11.9024 | 36.6163 | 19.0818 | 23.5102 | 347.788 | 4.19592 |
| Pc14G021113 | 122.156 | 93.0183 | 65.0926 | 210.685 | 165.762 | 249.378 | 31.8317 |
| Pc01G000856 | 6.76562 | 25.8172 | 394.865 | 19.9319 | 0.836135 | 0.507798 | 3.62914 |
| Pc13G019898 | 31.8245 | 19.574 | 718.243 | 753.052 | 71.5682 | 137.04 | 39.9517 |
| Pc07G012572 | 10.6242 | 10.9193 | 3869.55 | 2.11173 | 1.56952 | 3.37519 | 27.5187 |
| Pc07G012571 | 8.7773 | 8.54894 | 4008.62 | 5.09299 | 1.92125 | 2.75519 | 17.8094 |
| Pc11G017174 | 40.1837 | 172.062 | 82750.2 | 12.865 | 2.03394 | 4.86915 | 500.846 |
| Pc03G005844 | 1.73498 | 21.4939 | 113.799 | 2.14216 | 38.4248 | 3.39789 | 5.29838 |
| Pc13G020039 | 1352.32 | 859.231 | 788.268 | 2051.09 | 1213.66 | 1359.22 | 1174.15 |
| Pc05G008536 | 23.2216 | 30.2095 | 14976.1 | 3.51156 | 1.58388 | 2.89328 | 152.526 |
| Pc11G017355 | 1.34879 | 9.95113 | 187.884 | 3.29445 | 2.53664 | 9.0034 | 5.47014 |

The table is the PVF genes expression (FPKM) in seven tissues (Hem, hemocyte; Te, testis; Ov, Ovary and albumen gland; Kn, kidney; Gl, gill; Hp, hepatopancreas; Em, Embryo).

**Table** S8 The OrthoFinder results of PVF genes among 9 species

|  | **A.cal** | **B.gla** | **C.gig** | **L.for** | **L.gig** | **L.ing** | **O.bim** | **P.can** | **P.inc** | **PVF genes** |
| --- | --- | --- | --- | --- | --- | --- | --- | --- | --- | --- |
| OG0012609 | 0 | 0 | 0 | 0 | 0 | 2 | 0 | 1 | 0 | Pc01G000856 |
| OG0000225 | 8 | 4 | 7 | 9 | 9 | 13 | 0 | 16 | 4 | Pc01G001347 |
| OG0000004 | 5 | 0 | 281 | 157 | 3 | 27 | 1 | 40 | 265 | Pc11G017174 |
| OG0008809 | 0 | 0 | 1 | 3 | 1 | 0 | 0 | 1 | 2 | Pc11G017355 |
| OG0000149 | 9 | 7 | 17 | 16 | 6 | 8 | 4 | 6 | 16 | Pc11G017496 |
| OG0000224 | 7 | 1 | 9 | 5 | 8 | 21 | 1 | 15 | 3 | Pc13G019898 |
| OG0000408 | 9 | 2 | 4 | 7 | 9 | 6 | 4 | 2 | 2 | Pc13G020039 |
| OG0001456 | 2 | 0 | 3 | 4 | 1 | 4 | 1 | 1 | 3 | Pc14G021113 |
| OG0000764 | 2 | 2 | 3 | 3 | 3 | 7 | 4 | 3 | 2 | Pc14G021373 |
| OG0001598 | 1 | 3 | 1 | 0 | 2 | 2 | 4 | 2 | 2 | Pc02G003139 |
| OG0000071 | 10 | 7 | 12 | 38 | 21 | 17 | 9 | 23 | 12 | Pc03G006170,Pc03G005844 |
| OG0001515 | 1 | 0 | 3 | 0 | 1 | 2 | 0 | 5 | 7 | Pc04G006630 |
| OG0006366 | 1 | 0 | 1 | 0 | 1 | 2 | 1 | 1 | 1 | Pc04G006645 |
| OG0022140 | 0 | 0 | 0 | 0 | 0 | 0 | 0 | 1 | 0 | Pc04G006698 |
| OG0001026 | 2 | 1 | 0 | 0 | 1 | 2 | 1 | 17 | 0 | Pc05G008536 |
| OG0002921 | 1 | 0 | 2 | 0 | 1 | 1 | 1 | 2 | 4 | Pc05G008710 |
| OG0000052 | 3 | 11 | 28 | 37 | 13 | 63 | 6 | 21 | 20 | Pc06G011006 |
| OG0002118 | 2 | 1 | 1 | 2 | 4 | 1 | 1 | 1 | 2 | Pc06G011503 |
| OG0007349 | 1 | 0 | 0 | 3 | 0 | 0 | 0 | 4 | 1 | Pc07G012571 |
| OG0023690 | 0 | 0 | 0 | 0 | 0 | 0 | 0 | 1 | 0 | Pc07G012572 |
| OG0000716 | 4 | 2 | 6 | 0 | 4 | 4 | 4 | 3 | 3 | Pc08G013082 |
| OG0024492 | 0 | 0 | 0 | 0 | 0 | 0 | 0 | 1 | 0 | Pc09G015543 |
| OG0024494 | 0 | 0 | 0 | 0 | 0 | 0 | 0 | 1 | 0 | Pc09G015545 |
| OG0024495 | 0 | 0 | 0 | 0 | 0 | 0 | 0 | 1 | 0 | Pc09G015546 |
| OG0024496 | 0 | 0 | 0 | 0 | 0 | 0 | 0 | 1 | 0 | Pc09G015547 |
| OG0024497 | 0 | 0 | 0 | 0 | 0 | 0 | 0 | 1 | 0 | Pc09G015548 |
| OG0000130 | 12 | 1 | 15 | 14 | 7 | 12 | 7 | 7 | 22 | Pc09G015550 |

This table showed the OrthoFinder results from *P.* *canaliculata* and 8 sequenced mollusc species, and these 28 candidate PVF genes were classified into 20 multiple-gene families (>= 2 genes) and 7 single-gene families (only one gene). The meaning of the abbreviations are: *A. cal, Aplysia californica; B. gla, Biomphalaria glabrata; C. gig, Crassostrea gigas; O. bim, Octopus bimaculoides; L. ana, Lingula anatina; L. for, Limnoperna fortune; L. gig, Lottia giganta; P. can, Pomacea* *canaliculata; P. fuc, Pinctada fucata.*

**Table S9. Description of the assembly data from the *P. canaliculata* gut samples**

|  | **Assembly data** |
| --- | --- |
| Raw data | 93.48 Gb |
| High quality data | 31.46 Gb |
| Assembled contigs length (> 500 bp) | 1.69 Gb |
| Contig N50 | 1.59 kb |
| ORFs number | 1,142,095 |
| Average ORF length | 604 bp |

The intestinal digesta from 70 adult snails of *P. canaliculata* were pooled into 6 samples for microbial DNA extraction and sequencing (Illumina HiSeq X10). Raw reads with adaptor sequences, low quality sequences, and contaminated DNA sequences were filtered before assembly by metaSPAdes (v3.11.1). Genes were predicted by Prodigal (v2.6.3) on contigs longer than 500 bp. ORF denotes open reading frame.

**Table S10_a. Taxonomic profile at phylum level of 6 *P. canaliculata*** gut samples

| **Phylum** | **Mean** | **SD** |
| --- | --- | --- |
| Acidobacteria | 2.15E-04 | 2.36E-05 |
| Actinobacteria | 6.67E-03 | 9.46E-04 |
| Bacteroidetes | 3.08E-02 | 5.31E-03 |
| Cyanobacteria | 1.03E-03 | 8.90E-05 |
| Firmicutes | 2.44E-02 | 2.19E-03 |
| Lentisphaerae | 1.72E-04 | 2.38E-05 |
| Others | 5.85E-04 | 1.60E-05 |
| Planctomycetes | 1.45E-03 | 9.16E-05 |
| Proteobacteria | 7.03E-01 | 3.83E-03 |
| Spirochaetes | 2.37E-02 | 1.04E-03 |
| Synergistetes | 1.76E-03 | 2.48E-04 |
| Tenericutes | 2.04E-03 | 1.44E-04 |
| Unaligned | 6.60E-02 | 9.02E-04 |
| Unclassified | 1.02E-01 | 4.26E-03 |
| Verrucomicrobia | 3.61E-02 | 4.62E-03 |

“Mean” and “SD” denote the mean and standard deviation of the relative abundance of each phylum in 6 gut microbiota samples. The taxonomic assignments of gut microbial genes (1,142,095 genes) were made by CARMA3. The relative abundance of a phylum in a sample was calculated by summing the relative abundance of the respective genes belonging to that phylum in that sample. “Unaligned” denotes genes that have no reference sequence in the NCBI-NR database. “Unclassified” denotes genes that could not unambiguously be assigned to a phylum.

**Table S10_b. Taxonomic profile at genus level of 6 *P. canaliculata*** gut samples

| **Genus** | **Mean** | **SD** |
| --- | --- | --- |
| *Acetobacterium* | 1.97E-04 | 1.45E-05 |
| *Achromobacter* | 8.73E-04 | 4.53E-05 |
| *Acidovorax* | 1.16E-02 | 1.01E-03 |
| *Acinetobacter* | 2.84E-03 | 4.81E-04 |
| *Aeromonas* | 9.36E-02 | 1.39E-03 |
| *Agrobacterium* | 3.48E-04 | 2.23E-05 |
| *Anaeroarcus* | 3.02E-04 | 7.42E-06 |
| *Anaeromusa* | 3.30E-04 | 7.85E-06 |
| *Atlantibacter* | 1.08E-03 | 1.66E-05 |
| *Azonexus* | 4.05E-04 | 2.80E-05 |
| *Azospira* | 2.98E-03 | 9.72E-05 |
| *Bacteroides* | 5.90E-03 | 1.85E-03 |
| *Bilophila* | 5.55E-04 | 2.65E-05 |
| *Brachymonas* | 6.93E-04 | 5.54E-05 |
| *Brevundimonas* | 2.67E-04 | 1.91E-05 |
| *Chryseobacterium* | 1.73E-04 | 2.19E-05 |
| *Citrobacter* | 4.04E-02 | 9.52E-04 |
| *Clostridium* | 1.13E-04 | 5.90E-06 |
| *Comamonas* | 2.74E-02 | 2.03E-03 |
| *Coraliomargarita* | 1.54E-03 | 2.31E-04 |
| *Cronobacter* | 2.38E-04 | 1.45E-05 |
| *Cyanobium* | 1.78E-04 | 2.20E-05 |
| *Dechloromonas* | 4.52E-04 | 2.51E-05 |
| *Delftia* | 2.26E-03 | 2.90E-04 |
| *Desulfomicrobium* | 1.09E-04 | 3.40E-06 |
| *Desulfovibrio* | 7.17E-02 | 2.64E-03 |
| *Dickeya* | 2.27E-04 | 1.14E-05 |
| *Dysgonomonas* | 1.75E-04 | 6.19E-06 |
| *Enterobacter* | 8.25E-02 | 2.89E-03 |
| *Escherichia* | 1.77E-03 | 6.20E-05 |
| *Glutamicibacter* | 1.45E-04 | 2.17E-05 |
| *Hafnia* | 7.36E-04 | 7.68E-05 |
| *Herbaspirillum* | 6.32E-04 | 5.53E-05 |
| *Klebsiella* | 2.52E-02 | 5.97E-04 |
| *Kluyvera* | 7.24E-03 | 4.90E-04 |
| *Kosakonia* | 3.83E-03 | 2.18E-04 |
| *Lactobacillus* | 7.70E-04 | 4.22E-05 |
| *Lactococcus* | 1.79E-03 | 4.77E-04 |
| *Leclercia* | 4.71E-03 | 9.09E-05 |
| *Leuconostoc* | 2.21E-04 | 2.33E-05 |
| *Magnetospirillum* | 1.26E-02 | 1.84E-04 |
| *Massilia* | 2.26E-04 | 4.01E-05 |
| *Microbacterium* | 2.63E-03 | 4.28E-04 |
| *Mitsuokella* | 2.90E-04 | 2.26E-05 |
| *Mumia* | 1.94E-04 | 8.20E-06 |
| *Mycobacterium* | 6.25E-04 | 1.08E-04 |
| *Mycoplasma* | 5.07E-04 | 6.14E-05 |
| Others | 3.40E-03 | 2.77E-05 |
| *Pantoea* | 1.38E-02 | 1.62E-03 |
| *Parabacteroides* | 1.23E-04 | 3.47E-05 |
| *Paracoccus* | 7.31E-04 | 3.20E-05 |
| *Phascolarctobacterium* | 1.20E-04 | 1.69E-05 |
| *Pleomorphomonas* | 1.08E-03 | 8.18E-05 |
| *Prevotella* | 8.46E-04 | 1.06E-04 |
| *Pseudomonas* | 2.24E-02 | 1.63E-03 |
| *Psychrobacter* | 1.23E-02 | 3.26E-03 |
| *Rahnella* | 8.99E-04 | 2.84E-05 |
| *Ralstonia* | 1.83E-04 | 4.24E-06 |
| *Raoultella* | 2.12E-03 | 6.18E-05 |
| *Rhizobium* | 3.97E-04 | 1.92E-05 |
| *Rhodopseudomonas* | 6.26E-04 | 8.44E-06 |
| *Salmonella* | 7.36E-04 | 3.77E-05 |
| *Selenomonas* | 5.83E-03 | 5.20E-04 |
| *Serratia* | 7.59E-04 | 4.57E-05 |
| *Shewanella* | 1.79E-02 | 2.40E-03 |
| *Shigella* | 1.77E-04 | 1.42E-05 |
| *Sphaerochaeta* | 1.46E-02 | 7.97E-04 |
| *Sphingobacterium* | 1.16E-04 | 1.37E-05 |
| *Stenotrophomonas* | 5.63E-03 | 3.22E-04 |
| *Synechococcus* | 2.12E-04 | 2.47E-05 |
| *Tolumonas* | 1.17E-04 | 4.71E-06 |
| *Treponema* | 4.40E-03 | 3.47E-04 |
| *Uliginosibacterium* | 2.48E-04 | 2.19E-05 |
| Unaligned | 6.60E-02 | 9.02E-04 |
| Unclassified | 4.10E-01 | 4.88E-03 |
| *Verrucomicrobium* | 1.14E-04 | 1.80E-05 |
| *Vibrio* | 1.69E-04 | 1.47E-05 |
| *Xanthomonas* | 1.28E-04 | 9.54E-06 |

“Mean” and “SD” denote the mean and standard deviation of the relative abundance of each genus in 6 gut microbiota samples. The taxonomic assignments of gut microbial genes (1,142,095 genes) were made by CARMA3. The relative abundance of a genus in a sample was calculated by summing the relative abundance of the respective genes belonging to that genus in that sample. “Unaligned” denotes genes that have no reference sequence in the NCBI-NR database. “Unclassified” denotes genes that could not unambiguously be assigned to a genus.

**Table S11. Putative genes and KOs in xenobiotics biodegradation and metabolism pathways in *P. canaliculata*** gut mirobiome

| **Pathway** | **Gene number** | **KO number** | **KO** |
| --- | --- | --- | --- |
| Aminobenzoate degradation | 2886 | 56 | K00141,K00493,K00680,K01034,K01035,K01045,K01077,K01078,K01093,K01101,K01113,K01426,K01501,K01512,K01576,K01692,K01721,K01781,K03380,K03788,K03862,K03863,K04099,K04100,K04101,K04105,K04107,K04108,K04109,K04110,K05599,K05600,K07824,K08295,K09461,K09474,K10215,K10219,K10221,K11311,K14333,K14338,K14578,K14581,K15054,K15055,K15056,K15057,K15060,K15064,K15066,K16319,K16320,K18541,K19709,K20458, |
| Atrazine degradation | 762 | 10 | K01428,K01429,K01430,K01457,K01941,K03382,K03383,K06035,K14048,K19837, |
| Benzoate degradation | 5489 | 78 | K00074,K00217,K00252,K00446,K00448,K00449,K00481,K00626,K00632,K01031,K01032,K01055,K01075,K01607,K01615,K01617,K01666,K01692,K01782,K01821,K01825,K01856,K01857,K02554,K03381,K03464,K04073,K04098,K04100,K04101,K04105,K04107,K04108,K04109,K04110,K04112,K04113,K04114,K04115,K04116,K04117,K04118,K05549,K05550,K05783,K05784,K07104,K07516,K07535,K07536,K07537,K07538,K07539,K07823,K07824,K10216,K10217,K10218,K10219,K10220,K10221,K10621,K13767,K14333,K14727,K16242,K16243,K16244,K16245,K16246,K16249,K16514,K16515,K18364,K18365,K18366,K19065,K20458, |
| Bisphenol degradation | 16 | 3 | K01045,K05913,K14520, |
| Caprolactam degradation | 1430 | 17 | K00002,K00496,K01053,K01453,K01692,K01782,K01825,K03379,K06446,K14519,K14731,K17754,K18199,K19960,K19961,K19962,K19963, |
| Chloroalkane and chloroalkene degradation | 2348 | 19 | K00001,K00114,K00121,K00128,K00148,K00531,K01560,K01561,K01563,K02586,K02588,K02591,K04072,K08726,K13953,K13954,K14421,K14422,K17067, |
| Chlorocyclohexane and chlorobenzene degradation | 940 | 23 | K00217,K00446,K00462,K01061,K01560,K01561,K01563,K01856,K03380,K03381,K03391,K04098,K06912,K07104,K10676,K15237,K15238,K16242,K16243,K16244,K16245,K16246,K16249, |
| Dioxin degradation | 502 | 13 | K00462,K00480,K01617,K01666,K01821,K02554,K04073,K10222,K14600,K15750,K18364,K18365,K18366, |
| Drug metabolism - cytochrome P450 | 2177 | 11 | K00001,K00121,K00129,K00157,K00274,K00485,K00699,K00799,K04097,K13299,K13953, |
| Drug metabolism - other enzymes | 1993 | 16 | K00088,K00106,K00207,K00569,K00699,K00757,K00758,K00760,K00857,K00876,K01195,K01431,K01464,K01489,K01951,K13421, |
| Ethylbenzene degradation | 402 | 8 | K00632,K00680,K14578,K14581,K14748,K14749,K14750,K18092, |
| Fluorobenzoate degradation | 797 | 11 | K00217,K01061,K01721,K01856,K03381,K03391,K05549,K05550,K05783,K05784,K08686, |
| Furfural degradation | 36 | 7 | K16873,K16874,K16876,K16877,K16878,K16879,K16880, |
| Metabolism of xenobiotics by cytochrome P450 | 2198 | 13 | K00001,K00078,K00079,K00121,K00129,K00699,K00799,K01253,K04097,K11822,K13299,K13953,K15303, |
| Naphthalene degradation | 1480 | 13 | K00001,K00121,K00480,K04072,K13953,K13954,K14578,K14581,K14584,K14586,K18242,K18243,K19958, |
| Nitrotoluene degradation | 1209 | 18 | K00169,K00170,K00171,K00172,K00196,K00198,K03518,K03519,K03520,K06281,K06282,K10678,K10679,K10680,K11180,K11181,K14578,K14581, |
| Polycyclic aromatic hydrocarbon degradation | 298 | 20 | K00448,K00449,K00480,K04100,K04101,K04102,K11943,K11947,K14578,K14581,K14600,K14604,K18067,K18068,K18069,K18074,K18075,K18076,K18077,K19065, |
| Steroid degradation | 514 | 15 | K01822,K03333,K05296,K05898,K15981,K15982,K15983,K16045,K16046,K16047,K16048,K16049,K16050,K16051,K18687, |
| Styrene degradation | 1108 | 16 | K00146,K00446,K00451,K01026,K01039,K01040,K01426,K01501,K01502,K01555,K01721,K01800,K07104,K10216,K14481,K16171, |
| Toluene degradation | 711 | 23 | K00055,K00141,K00217,K01061,K01856,K03380,K03381,K05797,K07544,K07546,K07550,K15760,K15762,K15764,K15765,K16242,K16243,K16244,K16245,K16246,K16249,K20200,K20218, |
| Xylene degradation | 826 | 22 | K00055,K00141,K00446,K01617,K01666,K01821,K02554,K04073,K05549,K05550,K05783,K05784,K07104,K10216,K10217,K10617,K10619,K10621,K16303,K18364,K18365,K18366, |

Functional annotation of the gut microbial genes (1,142,095 genes) was based on the KEGG database (release 79). The summary of genes and KOs in xenobiotics biodegradation and metabolism pathways was shown.

**Table S12. Putative CAZymes identified in *P. canaliculata*** gut mirobiome

| **Class** | **Gene number** | **Family number** | **Family** |
| --- | --- | --- | --- |
| Auxiliary Activities (AAs) | 336 | 6 | AA10,AA12,AA3,AA4,AA6,AA7, |
| Carbohydrate-Binding Modules (CBMs) | 255 | 26 | CBM13,CBM2,CBM20,CBM22,CBM27,CBM32,CBM34,CBM35,CBM38,CBM4,CBM41,CBM44,CBM47,CBM48,CBM5,CBM51,CBM54,CBM57,CBM6,CBM61,CBM67,CBM73,CBM74,CBM77,CBM8,CBM9, |
| Carbohydrate Esterases (CEs) | 1633 | 16 | CE1,CE10,CE11,CE12,CE13,CE14,CE15,CE16,CE2,CE3,CE4,CE5,CE6,CE7,CE8,CE9, |
| Glycoside Hydrolases (GHs) | 7619 | 99 | GH1,GH10,GH100,GH101,GH102,GH103,GH104,GH105,GH106,GH108,GH109,GH110,GH111,GH112,GH113,GH114,GH115,GH116,GH117,GH119,GH12,GH120,GH123,GH125,GH126,GH127,GH128,GH129,GH13,GH130,GH133,GH135,GH14,GH15,GH16,GH17,GH18,GH19,GH2,GH20,GH23,GH24,GH25,GH26,GH27,GH28,GH29,GH3,GH30,GH31,GH32,GH33,GH35,GH36,GH37,GH38,GH39,GH4,GH42,GH43,GH44,GH46,GH47,GH5,GH50,GH51,GH52,GH53,GH57,GH59,GH6,GH62,GH63,GH64,GH65,GH67,GH71,GH73,GH74,GH77,GH78,GH79,GH8,GH81,GH84,GH85,GH87,GH88,GH89,GH9,GH90,GH91,GH92,GH93,GH94,GH95,GH97,GH98,GH99, |
| GlycosylTransferases (GTs) | 4815 | 45 | GT1,GT10,GT11,GT14,GT17,GT19,GT2,GT20,GT21,GT23,GT25,GT26,GT27,GT28,GT3,GT30,GT31,GT32,GT35,GT39,GT4,GT41,GT5,GT51,GT53,GT56,GT6,GT62,GT66,GT7,GT70,GT73,GT75,GT76,GT8,GT81,GT83,GT84,GT85,GT87,GT89,GT9,GT90,GT94,GT99, |
| Polysaccharide Lyases (PLs) | 449 | 16 | PL1,PL10,PL11,PL12,PL14,PL15,PL17,PL2,PL22,PL3,PL4,PL5,PL6,PL7,PL8,PL9, |

The gut microbial genes (1,142,095 genes) were annotated with dbCAN (release 5.0) to predict the carbohydrate active enzymes (CAZymes).

**Supplementary figures**


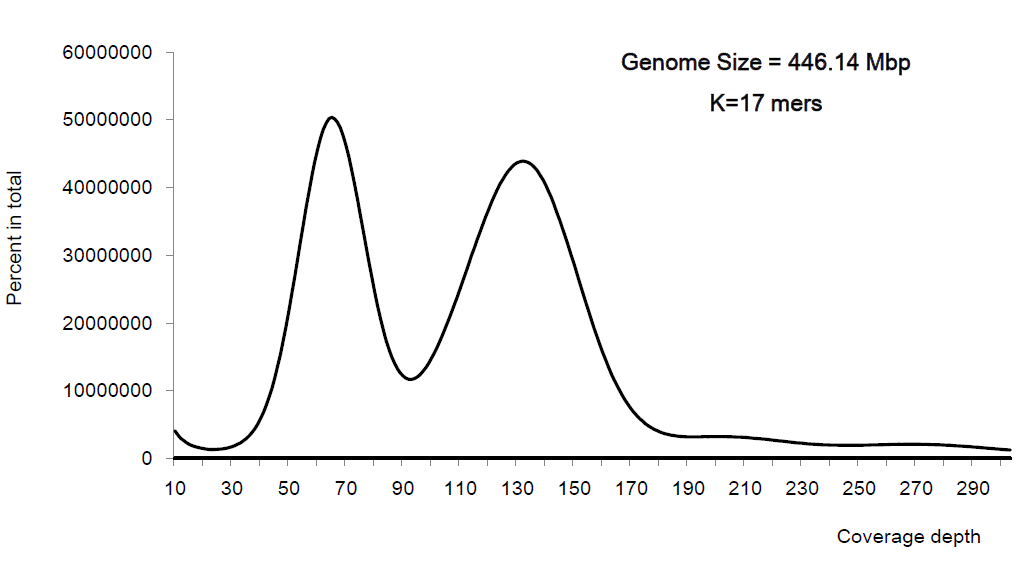


**Figure S1. Distribution of k-mer frequency and genome size estimation.** To address the heterozygosity and genome size, k-mer frequency was analyzed based on Illumina reads. High heterozygosity was illustrated by the double peaks on the distribution curve of k-mer frequency

**Figure S2.** **Mitochondria assembly vs NCBI mitochondria.** The pairwise alignment of assembled mitochondria genome and the *P. canaliculata* mitochondria from NCBI (GenBank: KJ739609.1) based on BLASTN program. The identity was indicated by synteny blocks, and the mismatched sequences were indicated in red.

**Figure S3. Gene function annotation shared by NR, KEGG, GO, eggNOG.** Gene functional annotation was performed by aligning the protein sequences to NCBI NR, UniProt, COG and KEGG databases with BLASTP v2.3.0+ under E-value cutoff of 10^-5^. The pathway analysis and functional classification were conducted based on KEGG database. InterProScan was used to assign preliminary GO terms, Pfam domains and IPR domains to the gene models.


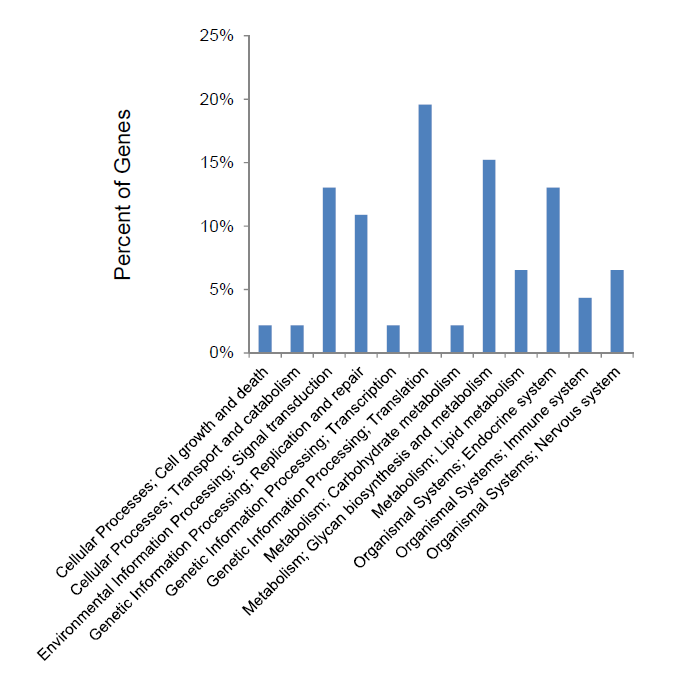


**Figure S4.** **KEGG annotation of common expansion gene families both in *P. canaliculata* and *L. fortunei*.** Based on orthologous groups in closed-related species, we identified the common expansion orthologous groups both in *P. canaliculata* and *L. fortunei*.


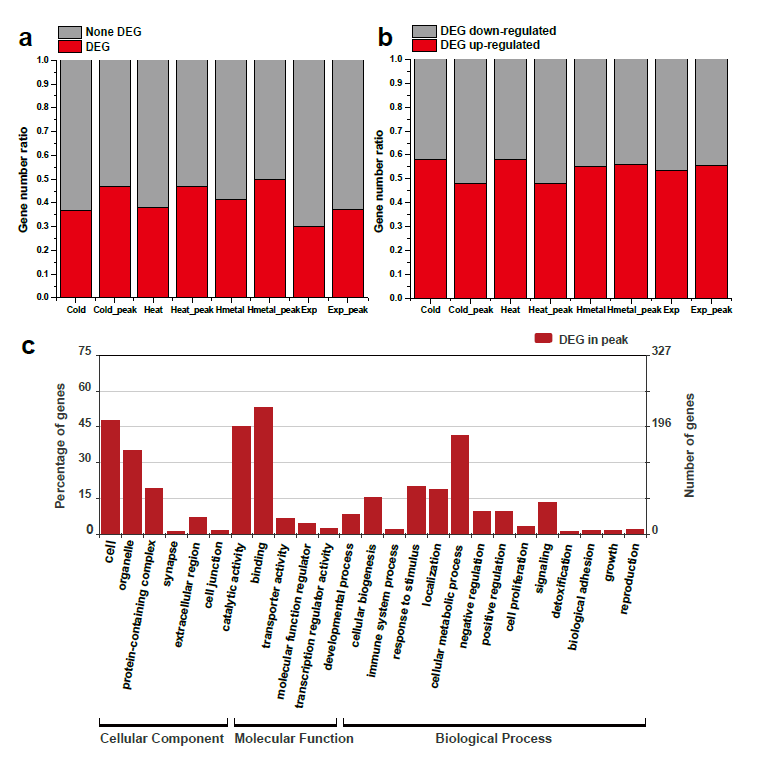


**Figure S5. Expression and function analysis of genes with DNA TEs within the ~4% peak.** a. the gene number percent of “DEG/None DEG” of genes with DNA TEs within the ~4% peak compare with the others. b. the gene number percent of “up-regulated DEG/down-regulated DEG” of genes with DNA TEs within the ~4% peak compare with the others. c. gene ontology annotation of DEGs with DNA TEs within the ~4% peak.


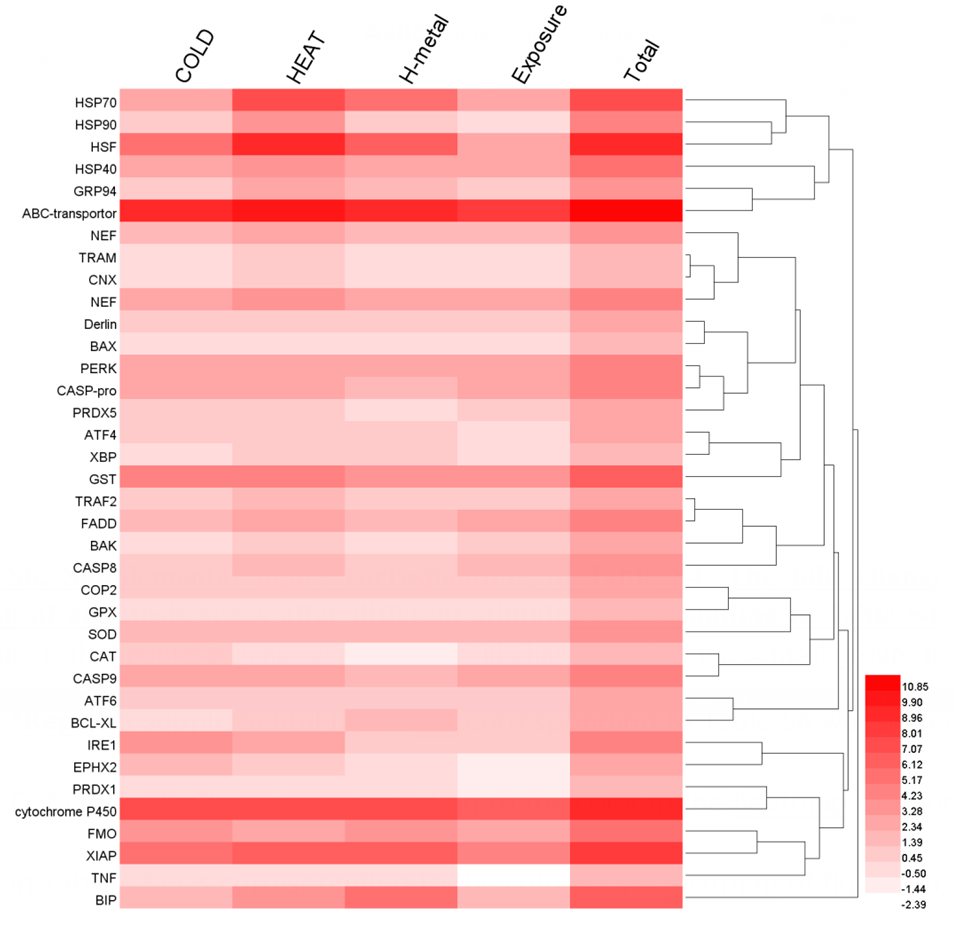


**Figure S6. Supplemental figure corresponding to Table S4. The fold change heatmap of apoptosis genes after different stimulus corresponding to Table S4.** The figure is the apoptosis genes expression (fold change against the control group) in hemocytes after cold, heat, heavy metal and air exposure. The scale in the right indicating the FPKM.
