# Supplementary material for: The genome of the golden apple snail Pomacea canaliculata provides insight into stress tolerance and invasive adaptation
Source: Gigascience. 2018 Aug 9;7(9):giy101. doi: 10.1093/gigascience/giy101 (PMC6129957; doi:10.1093/gigascience/giy101)
Supplement: Response_to_Reviewer_Comments_Original_Submission.pdf [file giy101_response_to_reviewer_comments_original_submission.pdf]

Author's Response To Reviewer Comments

Close

GIGA-D-18-00030
The genome of golden apple snail Pomacea canaliculata provides insight into stress tolerance and invasive adaptation
Conghui Liu; Bo Liu; Yuwei Ren; Yan Zhang; Hengchao Wang; Shuqu Li; Fan Jiang; Lijuan Yin; Guojie Zhang; Wanqiang Qian; Wei Fan
GigaScience

Dear Dr Fan,

Your manuscript "The genome of golden apple snail Pomacea canaliculata provides insight into stress tolerance and invasive adaptation" (GIGA-D-18-00030) has been assessed by our reviewers. Although it is of interest, we are unable to consider it for publication in its current form. The reviewers have raised a number of points which we believe would improve the manuscript and may allow a revised version to be published in GigaScience.
Reply: we have made revisions according to the reviewer's suggestions.

Their reports, together with any other comments, are below. Please also take a moment to check our website at https://giga.editorialmanager.com/ for any additional comments that were saved as attachments.

Please consider including more recent data on other molluscs in your analyses - see the report of reviewer 3 below.
Reply: We have added a new mollusc species "golden mussel Limnoperna fortunei", and replaced the data for "pearl oyster Pinctada fucata" with the latest version.

Please carefully revise the manuscript for language use and grammar, ideally with the help of a native speaker. Please note the attached file of one of the reviewers (available via Editorial Manager), which contains some suggestions for improvements
Reply: We have revised the language and grammar, and asked a native speaker for polishing. We also adopted the suggestions from the attached file of one of the reviewers.

Author roles: I note that you indicate four "equally contributing" first authors. Please note that we cannot indicate more than three "equally contributing" co-first authors, and please be aware that shared first authorship is reserved for exceptional cases where the contribution of two or three authors is indeed exactly equal.
Reply: We have reduced the co-first authors to three.

If you are able to fully address these points, we would encourage you to submit a revised manuscript to GigaScience. Once you have made the necessary corrections, please submit online at:

https://giga.editorialmanager.com/

Reply: After fully addressed all the points, we re-submitted the manuscript to GigaScience.

If you have forgotten your username or password please use the "Send Login Details" link

to get your login information. For security reasons, your password will be reset.

Please include a point-by-point within the 'Response to Reviewers' box in the submission system. Please ensure you describe additional experiments that were carried out and include a detailed rebuttal of any criticisms or requested revisions that you disagreed with. Please also ensure that your revised manuscript conforms to the journal style, which can be found in the Instructions for Authors on the journal homepage.

The due date for submitting the revised version of your article is 21 Jun 2018.

I look forward to receiving your revised manuscript soon.

Best wishes,

Hans Zauner
GigaScience
www.gigasciencejournal.com

Reviewer reports:

Reviewer #1: In their manuscript Liu et al. reported the genome sequence of the golden apple snail Pomacea canaliculata. They constructed chromosomal-level genome assembly using HiSeq, PacBio, and Hi-C sequencing technologies. They also tested differential gene expression under various environmental stress, showing many genes are responded to maintain homeostasis. In addition, they sequenced gut metagenome of the snail for the first time, implying that microorganisms contribute to digestion and resistance to xenobiotics of the host animal.

I think the massive data provides fundamental information to understand the biology of the animal as well as molluscs, therefore the study is valuable to be published in the journal GigaScience after some corrections.

Overall, the methods are appropriate, but description and interpretation of the results look not sufficient in some points as shown below.

P. 5, lines 94-96
"such as Califonia sea hare, Pacific oyster, Pearl oyster,..."
should be "such as the Califonia sea hare, the Pacific oyster, the pearl oyster,..."
There are many mistakes like this. I won't mention all of them. Please consult professional English editor before submitting the revision.
Reply: We have corrected this mistake in the new submitted manuscript.

P. 7, lines 148-150
"genes from seven related species..."
In fact eight species including Pinctada fucata were analyzed in figures 2a and 4a. Takeuchi et al.(2016, Zoological Letters, 2:3) and Luo et al.(2015, Nature Communications, 6, 8301) should be referred for P. fucata and Lingula anatina genome data, respectively.
Reply: We have corrected the species number. Because a new species is added into analysis, now the total species number is nine. The reference paper of the new species “Uliano-Silva M, Dondero F, Dan Otto T, Costa I, Lima NCB, Americo JA, et al. A hybrid-hierarchical

genome assembly strategy to sequence the invasive golden mussel Limnoperna fortunei. Gigascience. 2017. doi: 10.1093/gigascience/gix128. ” were also added at line 104 in the new submitted manuscript.

In addition, please carefully correct scientific names in Abbreviations and figures.
"Lottia gigantean" should be "Lottia giganta"
"Aplysia california" should be "Aplysia californica."
"Lingula anatine" should be "Lingula anatina"
Reply: we have corrected all the mistakes on scientific names in Abbreviations and figures.

P. 9 178-179
From the results I could not understand how the idea that the "DNA/hAT-Charlie TEs... promote the potential plasticity in the stress adaptation" came. This hypothesis can be tested using the present RNA-seq data, by checking whether the TEs are up-regulated under the stresses.
Reply: Transposons can insert into any genomic regions, which may change the gene regulations, or modify the gene structure thus form new functions. If a genome has high transposon activity, then it has high ability to adapt to the changing environment, so the recent explosion of DNA TEs may benefit the fast evolution of P. canaliculata in the recent history. There were several previous studies (Hua-Van A, Le Rouzic A, Boutin TS, Filée J, Capy P. The struggle for life of the genome's selfish architects. Biol Direct. 2011;6:19; Werren JH. Selfish genetic elements, genetic conflict, and evolutionary innovation. Proc Natl Acad Sci U S A. 2011;108:10863-70) on this issue that provides evidences that TEs can introduce small adaptive changes for a species.
Using the RNA-seq data to resolve this question is good idea. In our understanding, TEs can’t be transcribed and translated as an independent element, except for some low and random transcriptions which are likely to be no functions. So we analyzed the expression of 709 genes including DNA elements that restricted to the 4% peak inside the gene region, compared with the other genes that outside the 4% peak. Differentially expressed genes (DEG) were defined here by P-value smaller than 0.05 for comparison of treatments (heat, cold, heavy metal and air exposure) and control data. The percent of DEGs in the 4% peak were higher than those of genes outside the peak (10.2% higher for heat, 8.6% higher for cold, 8.6% higher for heavy metal, and 7.3% higher for air exposure). Among the DEGs in the 4% peak, about half are up-regulated and the other half are down-regulated. Moreover, the DEGs in the 4% peak were mainly enriched in cellular metabolic process, response to stimulus, localization and signaling by GO annotation. These results indicated that genes in the 4% peak were likely to be more active in the response of stimulus, promoting the potential plasticity in the stress adaptation.
The figure and related context was added in the new manuscript.

P.11 lines 232-236
The authors claimed that the P. canaliculata CYP gene family expanded compare to other molluscs. But the gene expansion of CYP looks common among molluscs. The number of the gene in P. canaliculata didn't significantly stand out from other molluscs (for example P. canaliculata has 157 genes and the Pacific oyster has 135). A molecular phylogeny in Fig 4a shows that lineage-specific gene expansion of CYP occurs not only in P. canaliculata but also other molluscs.
Reply: We appreciate the reviewer’s comments. We claimed CYP450 family as expansion

for two reasons. 1) Although the gene number of CYP450 in P. canaliculata was close to C.gigas (135) and A. californica (128), the gene ratio of “CYP450 genes/total genes in genome” was distinct, namely P. canaliculata (157/21553,~0.0073), C.gigas (135/46748, ~0.0029) and A. californica (128/27591, ~0.0046). 2) The expansion was more obvious in special subfamily, such as CYP3A (P.c 56, A.c 24, Bg 21, C.g 12, L.g 10, P.f 23 and O.b 20) and CYP 2U (P.c 42 A.c 5, Bg 2, C.g 0, L.g 8, P.f 0 and O.b 2) (figure 4). However, we weaken the mood when refer to expansion and withdraw the adjective “great”.

P. 17 lines 346-354
"The rich phenotypic... in laboratory."
These sentences should be move to Introduction.
Reply: We have moved "The rich phenotypic... in the laboratory." into introduction part between line 71 and 80.

P. 18 lines 380-381
"total messenger RNAs"
Total RNA or messenger RNA?
Reply: Here we mean “messenger RNA”. The sentence was revised to be “In final, total RNAs were extracted from the stored tissues of P. canaliculata materials, and then mRNAs were pulled out by beads with poly-T for constructing cDNA libraries.”

P. 21 line 445
Please cite the literature of "previous results."
Reply: We have added the literature “Sun J, Zhang Y, Xu T, Zhang Y, Mu H, Zhang Y, et al. Adaptation to deep-sea chemosynthetic environments as revealed by mussel genomes. Nature Ecology & Evolution. 2017.1(5), 121; Benton MJ, Donoghue, PCJ, Asher RJ. in The Timetree of Life:Calibrating and Constraining Molecular Clocks (eds Hedges, S. B. & Kumar, S.)35–86 Oxford Univ. Press, 2009; Zapata F, Wilson NG, Howison M, Andrade SC, Jörger KM, Schrödl M, Goetz FE, Giribet G, Dunn CW. Phylogenomic analyses of deep gastropod relationships reject Orthogastropoda. Proc Biol Sci. 2014 281:20141739. doi: 10.1098/rspb.2014.1739” in revised version.

Figure 2
The title "Evolutionary genomic analysis between P. canaliculata and other molluscs" is not appropriate because Lingula is a brachiopod.
Reply: The title of Figure 2 is changed to “Evolutionary genomic analysis of P. canaliculata”, because our focus is the species of P. canaliculata, other species were used for comparison.

Figure 4
Method for molecular phylogeny construction of CYP genes should be described.
Reply: The method was described in Figure 4 legend “The tree was constructed using the maximum likelihood method in MEGA7, and the branch length scale indicates the average number of residue substitutions per site”.

Figure S1
Which K-mer size used?

Reply: here we used 17-mer, the K-mer size is 17.

Table S4, S5, and S7
It is not reader-friendly to show the huge data in a table. I couldn't recognize what is the message of the data. Why not visualize the data in a heat map like Fig4b.
Reply: A supplemental figure S6 corresponding to Table S4 was added.
Data in Table S5 was corresponding to the color in the heatmap of figure 4b, Data in Table S7 was corresponding to the color in the heatmap of figure 5b, so there is no need to add other heat map figures.

Table S9
What "Mean" and "SD" indicate? E-value of blast results? Please describe.
Reply: "Mean" and "SD" indicate the mean and standard deviation of relative abundance of a phylum or a genus from the 6 gut microbiota samples. We also added a note under the Table.

Reviewer 2: - [the reviewer has no specific comments to the authors at this point, but recommends careful improvements of language and grammar]
Reply: We have improved the language and grammar, and polished the text by native speakers.

Reviewer #3: This manuscript presents a high-quality genome assembly for the snail P. canaiculata. Such genome and further analysis presented will contribute deeply for future studies of the molecular evolution and adaptation of molluscs, as well as to the study of the molecular mechanisms leading to - or involved with - invasive species success. I also point out the relevance of a first qualitative description of a high-depth gut microbiome for a snail. For such reasons, I recommend the publication of this manuscript. Nevertheless, I would like to recommend some essential revision prior publication.

First, the English has to be revised. I'll give a few examples bellow, and authors will find major marks in purple concerning specifically the need of English revision in the revised pdf attached. However, the entire manuscript would benefit from a native English speaker revision.
Reply: we have revised the descriptions highlighted in the attached pdf, and also asked a native speaker to help polish the language.

Examples of sentences needing English revision:

Lines 50-51: "causing severe economic loss each year as a result of yield loss, replanting cost and the funds of control." - rephrasing necessary.
Reply: This sentence is modified to “causing severe economic losses each year as a result of yield loss, replanting cost and expenditures on control.”

Line 52: "More seriously, P. canaliculata has involved in the transmission of a human fatal disease."
Reply: This sentence is modified to “More seriously, P. canaliculata has been involved in the transmission of a fatal human disease,”

Line 57: "causing great challenge to human health"

- rephrasing advised.
Reply: This sentence is modified to “creating a great challenge in terms of human health.”

Line 58: "Molluscs is …" - English correction necessary.
Reply: This sentence is modified to “Molluscs are a highly diverse group, second only to arthropods in species number, and their high biodiversity makes them an excellent model to address issues such as biogeography, adaptability and evolutionary processes.”

Lines 92-94: "However, researches at whole genome level in P. canaliculata still lags far behind other mollusks species, due to the lack of a high-quality reference genome. By far, multiple draft…" - rewriting necessary.
Reply: This sentence is modified to “However, research at the whole-genome level in P. canaliculata still lags far behind that in other mollusc species due to the lack of a high-quality reference genome. Multiple draft genomes of molluscs have been published, including the genomes of the California sea hare, Pacific oyster, pearl oyster, owl limpet, California two-spot octopus, golden mussel, and Biomphalaria snails, greatly promoting research on mollusc genomics.”

Line 263: "was" should be "were".
Reply: Corrected in the new manuscript.

Data and analysis related comments:

Lines 36-37: The description of the genome and the several molecular expression data are great contributions for the further understanding of molluscan and invasive biology. Nevertheless, we should avoid direct jumps to conclusions such as in lines 36 and 37, as the results in the manuscript don't present tools or direct ways to prevent invasions or pathogen transmission. I advise the withdraw of such sentence.
Reply: We agree to the suggestion, and have removed that sentence “Our results not only strengthen the understanding of molluscs genomics and biological invasion, but also benefit preventing the invasion of apple snail and transmission of pathogenetic parasites.”

Line 47: I would rephrase the sentence here in line 47. Even though the biology of the species may positively influence its invasive capacity, such characteristics are not exclusive of invasive mollusks. For that reason, I would exclude the "was due to" (line 47) which implies causality.
Reply: In the revised manuscript, we rephrased “was due to” to be “is closely related to”.

Line 63: Please present and refer to the lower temperature the species can establish populations in.
Reply: We added a sentence “P. canaliculata has been reported to establish populations at temperatures ranged from 10 °C to 35 °C” in the new manuscript, as well as two reference papers (Seuffert ME, Burela S, Martín PR. Influence of water temperature on the activity of the freshwater snail Pomacea canaliculata (Caenogastropoda: Ampullariidae) at its southernmost limit (Southern Pampas, Argentina). Journal of Thermal Biology. 2010; 35:77-84; Matsukura K, Tsumuki H,Izumi Y, Wada T. Physiological response to low temperature in the freshwater apple snail, Pomacea canaliculata (Gastropoda: Ampullariidae). J Exp Biol. 2009;212:2558-63).

Line 95: I would cite here also the draft genome of the invasive Limnoperna fortunei mussel.
Reply: The golden mussel "Limnoperna fortunei" and the related article were added in the new manuscript.

Line 95: There is a new version of the Pearl oyster published. If analysis were performed with data cited in line 95, I would advise for updating the analysis with proteins from the new genome (Du X, Fan G, Jiao Y et al. The pearl oyster Pinctada fucata martensii genome and multi-omic analyses provide insights into biomineralization. Gigascience 2017;6(8):1-12).
Reply: We have replaced the proteins data of Pinctada fucata to the latest version, and updated all the analysis in the new manuscript.

Line 100-101: Rephrasing is necessary as cellular homeostasis, color and nutrient of the eggs are not species-specific invasive characteristics.
Reply: We revised "invasive characters" to "environmental adaptation characteristics".

Line 104-105: same argument as for lines 36-37. Some rephrasing starting from "interrupt transmission…" is necessary.
Reply: We agree with the suggestion, and weakened the mood. The sentence is modified to "and provide a basis for interrupting the transmission of pathogenetic nematode parasites".

Table S1: Table S1 would benefit of having 2 columns: one with (i) number of reads generated and (ii) total bp produced for each library, instead of having a column 'Data size' (and what G bp means?).
Reply: We have made 2 columns in Table S1 according to the suggestions. One column refers to number of sequenced reads, the other column refers to number of sequenced bases.

Line 122: The ratio of genome coverage by reads used as input in the assembly? Rephrase it together with the sentences in lines 126-127, please.
Reply: In this sentence "another important aspect for evaluating genome assembly is the ratio of genome coverage." (between line 132 and 133), we want to explain that the ratio of assembly coverage is important. In P. canaliculata, the genome size of 446 Mb was estimated by the distribution of k-mer frequency. In this assembly genome, ~98.6 % sequence has been assembled.
In the sentence "we mapped the Illumina shotgun reads to the assembled reference genome. Significantly, 97% and 95% of the genome-derived and transcriptome-derived reads, respectively, could be aligned to the reference genome," (between line 136 and 137), we want to confirm the accuracy and no obvious bias for sequencing and assembly.

Line 123-124 and line 403: Please estimate and present the levels of heterozygosity using the illumina reads.
Reply: We used K-mer with K-size 17 to estimate the genome heterozygosity based on algorithm from reference (Liu B, et al. Quantitative Biology 2013:arXiv:1308.2012 [q-bio.GN]). The estimated heterozygosity of P. canaliculata range from 1% to 2%. In addition, we also used FIndError (Gnerre S et al., 2011) in the Allpath-LG package to estimate the heterozygosity, the result is 1.75%, consistent with the first method.
We have added it in revised manuscript. "With an estimated genome size of 446 Mb and genome heterozygosity between 1% and 2% based on the distribution of k-mer frequency."

Line 415-416: "Then, the protein-coding sequences were mapped by RNA-seq data." - please explain this sentence.
Reply: To determine whether the predicted genes are expressed or not, we used the transcriptome data to map to the CDS of genes. The gene models were retained if they had at least one supporting evidence from UniProt database, InterProScan domain and RNA-seq data.
To be more clear, we have revised this sentence in the new manuscript: “Then, these gene models were annotated by RNA-seq data, UniProt database and InterProScan software”.

Line 163: Withdraw "and so on".
Reply： "and so on" is removed.

Lines 146-163: To start understanding if the genome composition itself - and not only regulation of gene expression - can play a major role in the success of invasive species, I would advise to compare gene family expansions and contractions between the genomes of two invasive mollusks, which is now possible once the draft genome of L. fortunei is available (GigaScience doi: 10.1093/gigascience/gix128.). Further discussion about the presence - or lack thereof - of common expansions and contractions of gene families would be a great contribution. Such gene families could be further investigated for their roles in the expression of phenotypes related to invasive ecology and behaviour. I would strongly suggest for a comparative analysis of P. canaliculata and L. fortunei protein sets leading to a new Figure S4 and brief discussion on the findings.
Reply: We agree with the reviewer’s comments. In the revised version, we added the genome data of L. fortune to re-construct the orthoFinder ortholog and paralog gene families. Then, we identified the common expanded gene families both in P. canaliculata and L. fortune. The functions of these gene families are mainly enriched in signal transduction, replication and repair, Translation, glycan biosynthesis and metabolism, Lipid metabolism, endocrine, immune and nervous system. And we have revised the results in Figure S4.

Line 171-172: "interestingly, only the results of DNA transposons showed a unique peak at ~4% divergence rate for P. canaliculata and C. gigas" - rewrite this sentence.
Reply: We rephrased it as “Notably, the TE class of DNA transposons showed a specific peak at a divergence rate of ~4% divergence rate for P. canaliculata and C. gigas”.

Line 249: Please indicate how many and which genes were highly induced to facilitate further investigation by other groups in the future.
Reply: Gene IDs“Pc06G011748, Pc06G011460, Pc06G011458, Pc06G011459, Pc04G006708, Pc04G006710 and Pc04G006707”were added.

Line 254 -257: This direct link between phenotype and molecular characteristics cannot be supported by your data. Please rephrase it.
Reply: We revised these sentences in the new manuscript:
“P. canaliculata has eggs characterized by abundant nutrients, reddish or pinkish colour, aerial oviposition and neurotoxicity due to the perivitelline Fluid (PVF), which fills the space between the eggshell and the embryo and consists of carbohydrates, lipids and proteins (Figure 5a).”

Line 264- 269: Please clarify what was performed here. In any case, blast alone is not the

best tool to predict orthology. I would use RBBH methods.
Reply: In the revised manuscript, we used the reported 59 PVF protein fragments as query to identify the PVF genes from P. canaliculata reference genes by blastp (e-value 10-5), and further used the requirements of more than 85% sequence identity and over half alignment length for the query to get 36 best hits, corresponding to 28 candidate P. canaliculata PVF genes, and then confirmed 6 perivitellin genes which encode the subunits of PcOvo, PcPV2, and PcPV3, according to their high RNA expression in ovary and albumen gland tissue.
As OrthoFinder could analyze the orthology and paralogy of more than two species at the same time, we utilized the OrthoFinder results to investigate the ortholog and paralog relationships of these P. canaliculata PVF genes compared with other 8 sequenced mollusc species. Notably, 5 of the 6 perivitellin genes fall into single-gene families. In other words, it is hard to detect any homologs for most of these perivitellin genes in other sequenced mollusc species. One reason may be that the divergence time is too long (>200 Mya), another reason may be that these major PVF genes may have experienced fast evolution in the history, in order to adapt to the changing environment. At last, we used the RBBH method (reciprocal best hit) to identify ortholog genes between each species pair, and the result is consistent with that of OrthoFinder.

Line 327: Conclusion and discussion? At this point, only conclusions should be stated. Please eliminate sentences from 346 to 359. [condense and add into introduction]
Reply: We have condensed this paragraph, and moved it to the introduction part between line 376 and line 382. The revised paragraph is:
"In this study, we report a fine reference genome of P. canaliculata, first chromosome-level Mollusca genome published. With its easy acquisition, rapid growth and efficient reproduction, P. canaliculata possesses the potential to be a model organism of Mollusca. As its the cellular complexity and conservation of pathways also make P. canaliculata a useful representative of Mollusca, the genome described in this study can be used to advance our understanding of the molecular mechanisms involved in various scientific questions regarding Mollusca."

Line 395: Please indicate software used for trimming.
Reply: We used an in-house software for trimming (clean_dapter, clean_lowqual, filter_unpaired_reads.pl), which is freely available at Github " https://github.com/fanagislab/common_use".

Line 424-431: I would state the masking before stating the gene prediction. Rewrite. Reply: We have moved the repeat paragraph before the gene prediction paragraph.

Line 448: Any trimming performed for the transcriptome?
Reply: Yes, we use the same method. This sentence is revised to:
"Transcriptome reads were trimmed with the same method for genomic reads (https://github.com/fanagislab/common_use), and then mapped to the reference genome of P. canaliculata using TopHat (v. 2.1.0) with default settings".

Line 591: Please make available a supplementary material with the IDs of all sequences presented in Figure 4b. Please explain the scale in the heat maps of figure 4b.
Reply: The IDs are listed in Supplemental table S5.
We explain the scale meaning in the legend of figure 4b. The scale for the left heat map

represent FPKM value, showing by gradually changing colors; The scale for the right heat map represent fold change (FPKM-stimulus/FPKM-control), showing by gradually changing colors. To be more clear, we also add two marks “FPKM” and “Fold-change” alongside the scale on the figure.

--

Please also take a moment to check our website at https://giga.editorialmanager.com/l.asp?i=38889&l=8DS0D5CA for any additional comments that were saved as attachments. Please note that as GigaScience has a policy of open peer review, you will be able to see the names of the reviewers.

Close
