# Supplementary material for: The genome of the golden apple snail Pomacea canaliculata provides insight into stress tolerance and invasive adaptation
Source: Gigascience. 2018 Aug 9;7(9):giy101. doi: 10.1093/gigascience/giy101 (PMC6129957; doi:10.1093/gigascience/giy101)
Supplement: Response_to_Reviewer_Comments_Revision_1.pdf [file giy101_response_to_reviewer_comments_revision_1.pdf]

Author's Response To Reviewer Comments

Close

Reviewer reports:
Reviewer #1: Still there are some typos in the revised manuscript.
For example, in line 181 "Pintada" must be "Pinctada".
Please carefully check the manuscript again before submission.
Reply: We have revised all the typos in the manuscript. We revised “Lottia giganta” to “Lottia gigantea” in line 161, “Pintada fucata” to “Pinctada fucata” in line 162, “giganta” to “gigantea” in line 166, “orthfinder” to “orthoFinder” in line 169, “L. fortune” to “L. fortunei” in line 172, “L. giganta” to “L. gigantea” in line 259, “Lottia giganta” to “Lottia gigantea” in line 462, “Pintada fucata” to “Pinctada fucata” in line 463, “giganta” to “gigantea” in line 476, “L. fortune” to “L. fortunei” in line 543, “L. giganta, Lottia giganta” to “L. gigantea, Lottia gigantea” in line 543, “L. giganta” to “L. gigantea” in Table 1, “L. giganta” to “L. gigantea” in the legend of Figure 4.

Reviewer #3: Dear authors,

Thank you for providing a revised version of the manuscript and for addressing my suggestions. I think this manuscript will be a great contribution for the genomic studies of mollusks and invasive species. I, however, still have a few comments.

1-) The written English is much improved, but there are still a few persistent mistakes. Such as "L. giganta" where it should be 'L. gigantea' and the same with "L. fortune" which is actually 'L. fortunei'.
Reply: We have revised “L. giganta” to “L. gigantea”, and “L. fortune” to “L. fortunei” in the manuscript. We revised “Lottia giganta” to “Lottia gigantea” in line 161, “giganta” to “gigantea” in line 166, “L. fortune” to “L. fortunei” in line 172, “L. giganta” to “L. gigantea” in line 259, “Lottia giganta” to “Lottia gigantea” in line 462, “giganta” to “gigantea” in line 476, “L. fortune” to “L. fortunei” in line 543, “L. giganta, Lottia giganta” to “L. gigantea, Lottia gigantea” in line 543, “L. giganta” to “L. gigantea” in Table 1, “L. giganta” to “L. gigantea” in the legend of Figure 4.

I've attached again a manuscript with some purple highlights of critical pieces of text that should be revised. For example, the sentence between lines 479-484 is too long and non-technical. The same for "With its easy acquisition" in line 377. The improvement of such sentences would greatly benefit the manuscript readers.
Reply: (1) The long and non-technical sentence between lines 479-484 “Raw reads were cleaned to exclude adapter sequences, low-quality sequences, and contaminated DNA. The adapter sequence was identified and trimmed from the reads by an ungapped dynamic programming algorithm; the low-quality part (head or tail) of the reads was trimmed off to ensure that the average error rate of the remaining reads was lower than 0.001; the reads that were mapped to contaminated DNA by BWA-MEM were filtered out…” has been revised to short sentences, with the non-technical description removed and the applied in-house program cited:
“The Illumina raw reads were filtered by trimming the adapter sequence and low-quality regions (https://github.com/fanagislab/common_use), resulting in high-quality reads with an average error rate < 0.001. Then, the reads mapped to the following genomes by BWA-MEM were filtered out (https://github.com/fanagislab/metagenome_analysis.git), to exclude

the contaminated host, food, parasite, and human DNA sequences …"
(2) The "With its easy acquisition" in line 377 has been revised to "With wide distribution", and the whole sentence became: "With wide distribution, rapid growth and efficient reproduction, P. canaliculata possesses the potential to be a model organism of Mollusca."

(3) The "orthologue groups" in line 170 has been revised to "orthologous groups".

(4) The "maintains" in line 238 has been revised to "contributes to", and the whole sentence became: "Apoptosis is a process of cell death when sensing stress, and the regulation of apoptosis contributes to the dynamic homeostasis of the internal environment."

(5) The sentence between lines 319-322 "The gut microbiome is well known as the second genome of animals and plays important roles in food digestion, immune defence, and other processes that are essential to the animal host. To investigate whether the gut microbiome influences the invasive lifestyle" has been improved to:
"The gut microbiome is regarded as the "second genome" of the host animal, due to the fact that the gut microbiota contributes to the food digestion, immune system development, and many other processes important to the host. To investigate the relationship between the gut microbiome and the invasive lifestyle of P. canaliculata."

Also, the final subtopic should not be "Conclusion and Discussion", at that point, I would say, its time to just conclude.
Reply: We have deleted "and Discussion" in the subtopic.

In the results sections, however, many paragraphs start with a discussion of the literature instead of presenting the results: I would advice to revise those, present results first in the paragraphs and then discuss them. Again, coherence benefit readers a great deal.
Reply: Yes, we agree that results should be presented in front of discussion. To make it easier to understand for the readers, the sentences in the head of these paragraphs are brief background information, not discussion on the results. Real discussions are put after the results, in the end of the paragraphs.

2-) The amount of data generated is one of the strongest points of the work presented. And specially because of that, a great deal of analysis can be performed. For example, as you have 60x coverage of PacBio data for the snail, I would suggest running the Falcon and Falcon-Unzip pipeline to actually phase the genome: separate the haplotypes, instead of trying to merge or just through away the variation, as described in lines 424-432. The high heterozygosity described for the species actually helps in the phasing of haplotypes: there are several manuscripts describing methods to do so. I would run FALCON and FALCON-unzip, then I would polish with Illumina and try filling gaps with it in the different haplotypes and then would use the Hi-C data. I know its a great deal of analysis and highly experimental, so I'll leave it as a suggestion. But I would be interested in having a supplementary material with the imperfect alternate contigs generated by the phasing. This is the kind of information that were almost impossible to obtain with the generation of short reads, but now the long-reads technologies allow us to phase some long genome portions, and this is a very valuable information to some of us. With that, we can start understanding how much variation there are - and what are their evolutionary implications - in coding and non-coding regions within a genome.

Reply: Assembly the two haplotype chromosomes with long-reads is a very good suggestion, and we agree that the phased chromosomal sequences have greater value than the current mosaic reference genome sequence. In fact, we have run both SMARTdenovo and Falcon/Falcon-unzip, and polished by Pilon with illumina reads. The biggest difference of SMARTdenovo from Falcon is that SMRTdenovo does not need to correct sequencing errors in the first step, but instead perform an overlap-layout-consensus algorithm directly. With algorithms improved in many aspects, SMARTdenovo can achieve good assembly results with moderate sequencing coverage (50 X), in contrast, Falcon usually needs higher sequencing coverage（100 X) to get a good assembly. In this study, using the 60 X apple snail Pacbio data, SMARTdenovo generates contigs with N50 length over 1 megabases, which is 4 times of that of Falcon/Falcon-unzip (240 Kb).

The comparison between SMARTdenovo and Falcon/Falcon-unzip assemblies showed that contigs assembled by SMARTdenovo had the assembly size of 473.04 Mb, N50 size of 1010.40 Kb and N90 size of 172.34 Kb; primary contigs assembled by Falcon had the assembly size of 475.28 Mb, N50 size of 241.14 Kb and N90 size of 54.29 Kb; alternate contigs assembled by Falcon had the assembly size of 54.10 Mb, N50 size of 43.88 Kb and N90 size of 22.68 Kb; primary contigs assembled by Falcon-unzip had the assembly size of 474.23 Mb, N50 size of 246.62 Kb and N90 size of 58.36 Kb; haplotigs assembled by Falcon-unzip had the assembly size of 173.15 Mb, N50 size of 48.98 Kb and N90 size of 17.44 Kb.

Considering that Hi-C contains extremely long-range linkage information, the larger contig length is an import factor for the success application of Hi-C data for scaffolding. Therefore, we adopted the SMARTdenovo contigs and then applied Hi-C to get a chromosomal-scale scaffold sequence.
To make the phasing information available to the public, we also uploaded the SMARTdenovo alternate sequences excluded from the reference haploid genome sequence, as well as the Falcon-unzip assembly of the apple snail, to the GigaDB and our institution's ftp-site, respectively.

3-) About the expansions found between the snail and L. fortunei, could you please describe the methodology used to consider genes expanded in these two groups?
Was this done in a comparative manner with other species? Which ones? What was the criteria to consider gene families expanded?
Reply: we added the sentence at method part "To identify the common expanded gene families, we compared the P. canaliculata and L. fortunei with other seven species. The gene number of orthologous group in P. canaliculata and L. fortunei were two or more times than that in all of other species, respectively. Additionally, these gene families with P-value less than 0.01 were considered as expansion by z-test."

3a-) Have you identified CPYs expanded in both invasive species? I would suggest that L. fortunei should be included in figure 4.
Reply: We have identified the CYP genes in the L. fortunei in the revised manuscript, which were included in Figure 4a. There were 115 CYP genes found in L. fortunei, with no obvious expansion.

Close
