# Supplementary material for: The genome of the golden apple snail Pomacea canaliculata provides insight into stress tolerance and invasive adaptation
Source: Gigascience. 2018 Aug 9;7(9):giy101. doi: 10.1093/gigascience/giy101 (PMC6129957; doi:10.1093/gigascience/giy101)
Supplement: Reviewer_1_Report_(Original_Submission) -- Takeshi Takeuchi, PhD — 3/12/2018 Reviewed [file giy101_reviewer_1_report_(original_submission).pdf]

Reviewer Report

Title: **The genome of golden apple snail Pomacea canaliculata provides insight into stress tolerance and invasive adaptation**

Version: **Original Submission** **Date:** 3/12/2018

Reviewer name: **Takeshi Takeuchi**

Reviewer Comments to Author:

In their manuscript Liu et al. reported the genome sequence of the golden apple snail Pomacea canaliculata. They constructed chromosomal-level genome assembly using HiSeq, PacBio, and Hi-C sequencing technologies. They also tested differential gene expression under various environmental stress, showing many genes are responded to maintain homeostasis. In addition, they sequenced gut metagenome of the snail for the first time, implying that microorganisms contribute to digestion and resistance to xenobiotics of the host animal.

I think the massive data provides fundamental information to understand the biology of the animal as well as molluscs, therefore the study is valuable to be published in the journal GigaScience after some corrections.

Overall, the methods are appropriate, but description and interpretation of the results look not sufficient in some points as shown below.

P. 5, lines 94-96
"such as Califonia sea hare, Pacific oyster, Pearl oyster,..."
should be "such as the Califonia sea hare, the Pacific oyster, the pearl oyster,..."
There are many mistakes like this. I won't mention all of them. Please consult professional English editor before submitting the revision.

P. 7, lines 148-150
"genes from seven related species..."
In fact eight species including Pinctada fucata were analyzed in figures 2a and 4a. Takeuchi et al.(2016, Zoological Letters, 2:3) and Luo et al.(2015, Nature Communications, 6, 8301) should be referred for P. fucata and Lingula anatina genome data, respectively.

In addition, please carefully correct scientific names in Abbreviations and figures.
"Lottia gigantean" should be "Lottia giganta"
"Aplysia california" should be "Aplysia californica."
"Lingula anatine" should be "Lingula anatina"

P. 9 178-179
From the results I could not understand how the idea that the "DNA/hAT-Charlie TEs... promote the potential plasticity in the stress adaptation" came. This hypothesis can be tested using the present RNA-seq data, by checking whether the TEs are up-regulated under the stresses.

P.11 lines 232-236
The authors claimed that the P. canaliculata CYP gene family expanded compare to other molluscs. But the gene expansion of CYP looks common among molluscs. The number of the gene in P. canaliculata didn't significantly stand out from other molluscs (for example P. canaliculata has 157 genes and the Pacific oyster has 135). A molecular phylogeny in Fig 4a shows that lineage-specific gene expansion of CYP occurs not only in P. canaliculata but also other molluscs.

P. 17 lines 346-354
"The rich phenotypic... in laboratory."
These sentences should be move to Introduction.

P. 18 lines 380-381
"total messenger RNAs"
Total RNA or messenger RNA?

P. 21 line 445
Please cite the literature of "previous results."

Figure 2
The title "Evolutionary genomic analysis between P. canaliculata and other molluscs" is not appropriate because Lingula is a brachiopod.

Figure 4
Method for molecular phylogeny construction of CYP genes should be described.

Figure S1
Which K-mer size used?

Table S4, S5, and S7
It is not reader-friendly to show the huge data in a table. I couldn't recognize what is the message of the data. Why not visualize the data in a heat map like Fig4b.

Table S9
What "Mean" and "SD" indicate? E-value of blast results? Please describe.

Methods

Are the methods appropriate to the aims of the study, are they well described, and are necessary controls included? Yes

Conclusions

Are the conclusions adequately supported by the data shown? Yes

Reporting Standards

Does the manuscript adhere to the journal's guidelines on minimum standards of reporting? Yes

Choose an item.

Statistics

Are you able to assess all statistics in the manuscript, including the appropriateness of statistical tests used? There are no statistics in the manuscript.

Quality of Written English

Please indicate the quality of language in the manuscript: Not suitable for publication unless extensively edited

Declaration of Competing Interests

Please complete a declaration of competing interests, considering the following questions:

- Have you in the past five years received reimbursements, fees, funding, or salary from an organisation that may in any way gain or lose financially from the publication of this manuscript, either now or in the future?
- Do you hold any stocks or shares in an organisation that may in any way gain or lose financially from the publication of this manuscript, either now or in the future?
- Do you hold or are you currently applying for any patents relating to the content of the manuscript?
- Have you received reimbursements, fees, funding, or salary from an organization that holds or has applied for patents relating to the content of the manuscript?
- Do you have any other financial competing interests?
- Do you have any non-financial competing interests in relation to this paper?

If you can answer no to all of the above, write 'I declare that I have no competing interests' below. If your reply is yes to any, please give details below.

I declare that I have no competing interests.

I agree to the open peer review policy of the journal. I understand that my name will be included on my report to the authors and, if the manuscript is accepted for publication, my named report including any attachments I upload will be posted on the website along with the authors' responses. I agree for my report to be made available under an Open Access Creative Commons CC-BY license (http://creativecommons.org/licenses/by/4.0/). I understand that any comments which I do not wish to be included in my named report can be included as confidential comments to the editors, which will not be published.

I agree to the open peer review policy of the journal

To further support our reviewers, we have joined with Publons, where you can gain additional credit to further highlight your hard work (see: https://publons.com/journal/530/gigascience). On publication of this paper, your review will be automatically added to Publons, you can then choose whether or not to claim your Publons credit. I understand this statement.

Yes Choose an item.
