# Supplementary material for: The genome of the golden apple snail Pomacea canaliculata provides insight into stress tolerance and invasive adaptation
Source: Gigascience. 2018 Aug 9;7(9):giy101. doi: 10.1093/gigascience/giy101 (PMC6129957; doi:10.1093/gigascience/giy101)
Supplement: Reviewer_3_Report_(Original_Submission) -- Marcela Uliano da Silva, PhD — 03/20/2018 Reviewed [file giy101_reviewer_3_report_(original_submission).pdf]

Reviewer Report

Title: **The genome of golden apple snail Pomacea canaliculata provides insight into stress tolerance and invasive adaptation**

Version: **Original Submission** **Date:** 3/20/2018

Reviewer name: **Marcela Uliano da Silva,**

Reviewer Comments to Author:

This manuscript presents a high-quality genome assembly for the snail P. canaiculata. Such genome and further analysis presented will contribute deeply for future studies of the molecular evolution and adaptation of molluscs, as well as to the study of the molecular mechanisms leading to - or involved with - invasive species success. I also point out the relevance of a first qualitative description of a high-depth gut microbiome for a snail. For such reasons, I recommend the publication of this manuscript. Nevertheless, I would like to recommend some essential revision prior publication.

First, the English has to be revised. I'll give a few examples bellow, and authors will find major marks in purple concerning specifically the need of English revision in the revised pdf attached. However, the entire manuscript would benefit from a native English speaker revision.

Examples of sentences needing English revision:

Lines 50-51: "causing severe economic loss each year as a result of yield loss, replanting cost and the funds of control." - rephrasing necessary.
Line 52: "More seriously, P. canaliculata has involved in the transmission of a human fatal disease."
Line 57: "causing great challenge to human health" - rephrasing advised.
Line 58: "Molluscs is …" - English correction necessary.
Lines 92-94: " However, researches at whole genome level in P. canaliculata still lags far behind other mollusks species, due to the lack of a high-quality reference genome. By far, multiple draft…" - rewriting necessary.
Line 263: "was" should be "were".

Data and analysis related comments:

Lines 36-37: The description of the genome and the several molecular expression data are great contributions for the further understanding of molluscan and invasive biology. Nevertheless, we should avoid direct jumps to conclusions such as in lines 36 and 37, as the results in the manuscript don't present tools or direct ways to prevent invasions or pathogen transmission. I advise the withdraw of such sentence.

Line 47: I would rephrase the sentence here in line 47. Even though the biology of the species may positively influence its invasive capacity, such characteristics are not exclusive of invasive mollusks. For that reason, I would exclude the "was due to" (line 47) which implies causality.

Line 63: Please present and refer to the lower temperature the species can establish populations in.

Line 95: I would cite here also the draft genome of the invasive Limnoperna fortunei mussel.

Line 95: There is a new version of the Pearl oyster published. If analysis were performed with data cited in line 95, I would advise for updating the analysis with proteins from the new genome (Du X, Fan G, Jiao Y et al. The pearl oyster Pinctada fucata martensii genome and multi-omic analyses provide insights into biomineralization. Gigascience 2017;6(8):1-12).

Line 100-101: Rephrasing is necessary as cellular homeostasis, color and nutrient of the eggs are not species-specific invasive characteristics.

Line 104-105: same argument as for lines 36-37. Some rephrasing starting from "interrupt transmission…" is necessary.

Table S1: Table S1 would benefit of having 2 columns: one with (i) number of reads generated and (ii) total bp produced for each library, instead of having a column 'Data size' (and what G bp means?).

Line 122: The ratio of genome coverage by reads used as input in the assembly? Rephrase it together with the sentences in lines 126-127, please.

Line 123-124 and line 403: Please estimate and present the levels of heterozygosity using the illumina reads.

Line 415-416: "Then, the protein-coding sequences were mapped by RNA-seq data." - please explain this sentence.

Line 163: Withdraw "and so on".

Lines 146-163: To start understanding if the genome composition itself - and not only regulation of gene expression - can play a major role in the success of invasive species, I would advise to compare gene family expansions and contractions between the genomes of two invasive mollusks, which is now possible once the draft genome of L. fortunei is available (GigaScience doi: 10.1093/gigascience/gix128.). Further discussion about the presence - or lack thereof - of common expansions and contractions of gene families would be a great contribution. Such gene families could be further investigated for their roles in the expression of phenotypes related to invasive ecology and behaviour. I would strongly suggest for a comparative analysis of P. canaliculata and L. fortunei protein sets leading to a new Figure S4 and brief discussion on the findings.

Line 171-172: "interestingly, only the results of DNA transposons showed a unique peak at ~4% divergence rate for P. canaliculata and C. gigas" - rewrite this sentence.

Line 249: Please indicate how many and which genes were highly induced to facilitate further investigation by other groups in the future.

Line 254 -257: This direct link between phenotype and molecular characteristics cannot be supported by your data. Please rephrase it.

Line 264- 269: Please clarify what was performed here. In any case, blast alone is not the best tool to predict orthology. I would use RBBH methods.

Line 327: Conclusion and discussion? At this point, only conclusions should be stated. Please eliminate sentences from 346 to 359.

Line 395: Please indicate software used for trimming.

Line 424-431: I would state the masking before stating the gene prediction. Rewrite.

Line 448: Any trimming performed for the transcriptome?

Line 591: Please make available a supplementary material with the IDs of all sequences presented in Figure 4b. Please explain the scale in the heat maps of figure 4b.

Methods

Are the methods appropriate to the aims of the study, are they well described, and are necessary controls included? Yes

Conclusions

Are the conclusions adequately supported by the data shown? Yes

Reporting Standards

Does the manuscript adhere to the journal's guidelines on minimum standards of reporting? Yes

Choose an item.

Statistics

Are you able to assess all statistics in the manuscript, including the appropriateness of statistical tests used? Yes, and I have assessed the statistics in my report.

Quality of Written English

Please indicate the quality of language in the manuscript: Needs some language corrections before being published

Declaration of Competing Interests

Please complete a declaration of competing interests, considering the following questions:

- Have you in the past five years received reimbursements, fees, funding, or salary from an organisation that may in any way gain or lose financially from the publication of this manuscript, either now or in the future?
- Do you hold any stocks or shares in an organisation that may in any way gain or lose financially from the publication of this manuscript, either now or in the future?
- Do you hold or are you currently applying for any patents relating to the content of the manuscript?
- Have you received reimbursements, fees, funding, or salary from an organization that holds or has applied for patents relating to the content of the manuscript?
- Do you have any other financial competing interests?
- Do you have any non-financial competing interests in relation to this paper?

If you can answer no to all of the above, write 'I declare that I have no competing interests' below. If your reply is yes to any, please give details below.

I declare that I have no competing interests.

I agree to the open peer review policy of the journal. I understand that my name will be included on my report to the authors and, if the manuscript is accepted for publication, my named report including any attachments I upload will be posted on the website along with the authors' responses. I agree for my report to be made available under an Open Access Creative Commons CC-BY license (http://creativecommons.org/licenses/by/4.0/). I understand that any comments which I do not wish to be included in my named report can be included as confidential comments to the editors, which will not be published.

I agree to the open peer review policy of the journal

To further support our reviewers, we have joined with Publons, where you can gain additional credit to further highlight your hard work (see: https://publons.com/journal/530/gigascience). On publication of this paper, your review will be automatically added to Publons, you can then choose whether or not to claim your Publons credit. I understand this statement.

Yes Choose an item.
