# Supplementary material for: The genome of the golden apple snail Pomacea canaliculata provides insight into stress tolerance and invasive adaptation
Source: Gigascience. 2018 Aug 9;7(9):giy101. doi: 10.1093/gigascience/giy101 (PMC6129957; doi:10.1093/gigascience/giy101)
Supplement: Reviewer_3_Report_(Revision_1) -- Marcela Uliano da Silva, PhD — 05/31/218 Reviewed [file giy101_reviewer_3_report_(revision_1).pdf]

Reviewer Report

Title: The genome of golden apple snail Pomacea canaliculata provides insight into stress tolerance and invasive adaptation

Version: Revision 1 Date: 5/31/2018

Reviewer name: Marcela Uliano da Silva

Reviewer Comments to Author:

Dear authors,

Thank you for providing a revised version of the manuscript and for addressing my suggestions. I think this manuscript will be a great contribution for the genomic studies of mollusks and invasive species. I, however, still have a few comments.

1-) The written English is much improved, but there are still a few persistent mistakes. Such as "L. giganta" where it should be 'L. gigantea' and the same with "L. fortune" which is actually 'L. fortunei'. I've attached again a manuscript with some purple highlights of critical pieces of text that should be revised. For example, the sentence between lines 479-484 is too long and non-technical. The same for "With its easy acquisition" in line 377. The improvement of such sentences would greatly benefit the manuscript readers. Also, the final subtopic should not be "Conclusion and Discussion", at that point, I would say, its time to just conclude. In the results sections, however, many paragraphs start with a discussion of the literature instead of presenting the results: I would advice to revise those, present results first in the paragraphs and then discuss them. Again, coherence benefit readers a great deal.

2-) The amount of data generated is one of the strongest points of the work presented. And specially because of that, a great deal of analysis can be performed. For example, as you have 60x coverage of PacBio data for the snail, I would suggest running the Falcon and Falcon-Unzip pipeline to actually phase the genome: separate the haplotypes, instead of trying to merge or just through away the variation, as described in lines 424-432. The high heterozygosity described for the species actually helps in the phasing of haplotypes: there are several manuscripts describing methods to do so. I would run FALCON and FALCON-unzip, then I would polish with Illumina and try filling gaps with it in the different haplotypes and then would use the Hi-C data. I know its a great deal of analysis and highly experimental, so I'll leave it as a suggestion. But I would be interested in having a supplementary material with the imperfect alternate contigs generated by the phasing. This is the kind of information that were almost impossible to obtain with the generation of short reads, but now the long-reads technologies allow us to phase some long genome portions, and this is a very valuable information to some of us. With that, we can start understanding how much variation there are - and what are their evolutionary implications - in coding and non-coding regions within a genome.

3-) About the expansions found between the snail and L. fortunei, could you please describe the methodology used to consider genes expanded in these two groups? Was this done in a comparative manner with other species? Which ones? What was the criteria to consider gene families expanded?
3a-) Have you identified CPYs expanded in both invasive species? I would suggest that L. fortunei should be included in figure 4.

Methods

Are the methods appropriate to the aims of the study, are they well described, and are necessary controls included? Choose an item.

Conclusions

Are the conclusions adequately supported by the data shown? Choose an item.

Reporting Standards

Does the manuscript adhere to the journal's guidelines on minimum standards of reporting? Choose an item.

Choose an item.

Statistics

Are you able to assess all statistics in the manuscript, including the appropriateness of statistical tests used? Choose an item.

Quality of Written English

Please indicate the quality of language in the manuscript: Choose an item.

Declaration of Competing Interests

Please complete a declaration of competing interests, considering the following questions:

- Have you in the past five years received reimbursements, fees, funding, or salary from an organisation that may in any way gain or lose financially from the publication of this manuscript, either now or in the future?
- Do you hold any stocks or shares in an organisation that may in any way gain or lose financially from the publication of this manuscript, either now or in the future?
- Do you hold or are you currently applying for any patents relating to the content of the manuscript?
- Have you received reimbursements, fees, funding, or salary from an organization that holds or has applied for patents relating to the content of the manuscript?
- Do you have any other financial competing interests?
- Do you have any non-financial competing interests in relation to this paper?

If you can answer no to all of the above, write 'I declare that I have no competing interests' below. If your reply is yes to any, please give details below.

I declare that I have no competing interests

I agree to the open peer review policy of the journal. I understand that my name will be included on my report to the authors and, if the manuscript is accepted for publication, my named report including any attachments I upload will be posted on the website along with the authors' responses. I agree for my

report to be made available under an Open Access Creative Commons CC-BY license (http://creativecommons.org/licenses/by/4.0/). I understand that any comments which I do not wish to be included in my named report can be included as confidential comments to the editors, which will not be published.

I agree to the open peer review policy of the journal

To further support our reviewers, we have joined with Publons, where you can gain additional credit to further highlight your hard work (see: https://publons.com/journal/530/gigascience). On publication of this paper, your review will be automatically added to Publons, you can then choose whether or not to claim your Publons credit. I understand this statement.

Yes Choose an item.
